# Integrating point-of-care diabetes detection with lifestyle counselling in community settings: outcomes from Western Sydney, Australia

**DOI:** 10.1186/s12913-024-11335-y

**Published:** 2024-08-13

**Authors:** Jaybee Serrano, Gideon Meyerowitz-Katz, Janine Dawson, Aruni Ratnayake, Sumathy Ravi, Helen Dick, Sian Bramwell, Mark Scott, Rajini Jayaballa, Glen Maberly

**Affiliations:** 1 Western Sydney Diabetes (WSD), Western Sydney Local Health District (WSLHD), Sydney, Australia; 2 School of Public Health, University of Sydney, Sydney, Australia; 3 School of Nursing, University of Wollongong, Wollongong, Australia; 4 School of Medicine, Macquarie University, Sydney, Australia; 5 Novo Nordisk Australia, Sydney, Australia

**Keywords:** Type 2 diabetes, Pre-diabetes, Detection, Diabetes education, Prevention, Community engagement, Point-of-care testing, Western Sydney

## Abstract

**Introduction:**

Early detection and prevention of type 2 diabetes and its complications are global health priorities. Optimal outcomes depend on individual awareness and proactive self-management of health risks. This study evaluates the effectiveness of a community-based diabetes detection and intervention program in a high-risk area in western Sydney, Australia.

**Research design and methods:**

We collaborated with the Workers Lifestyle Group, Tamil Association Arts and Culture Association, and the National Aboriginal and Islanders Day Observance Committee to implement our program. Participants underwent HbA1C testing via point-of-care blood spot testing. They received personalized feedback, education on diabetes management, and were offered opportunities to enrol in lifestyle modification programs. Participants identified with pre-diabetes (HbA1C 5.7–6.4%) or diabetes (HbA1C > 6.4%) were advised to consult their General Practitioners (GPs). A follow-up questionnaire was distributed 3–8 months post-intervention to evaluate the programs usefulness and relevance and lifestyle changes implemented by the participants.

**Results:**

Over eight months, 510 individuals participated. Of these, 19% had an HbA1C > 6.4%, and 38% had levels between 5.7 and 6.4%. Among those with diabetes, HbA1C levels ranged as follows: 56% <7%; 20% 7-7.9%; 18% 8-8.9%; and 5% >9%. Post intervention survey indicated that the program was well-received, with 62.5% of responses reporting lifestyle changes and 36.3% seeking further advice from their local healthcare providers.

**Conclusion:**

The study demonstrates a significant prevalence of pre-diabetes and diabetes in the community, similar to findings from larger-scale hospital and general practice studies. Point-of-care testing combined with personalized education effectively motivated participants toward healthier lifestyle choices and medical consultations. The paper discusses the scalability of this approach for broader population.

**Supplementary Information:**

The online version contains supplementary material available at 10.1186/s12913-024-11335-y.

## Key messages

***What is already known in this topic***

• There is a high burden of prediabetes and diabetes in Western Sydney that has a significant impact in the health system and economy

• Early detection of prediabetes and diabetes can prevent future complications

***What does this study adds***

• A community detection and individualised education can motivate behaviour change. This initiative was a feasibility study which demonstrates a proactive approach in detecting diabetes in the community more broadly, especially to those harder to reach communities

***How this study might affect research, practice or policy***

• Combining real-time HbA1c results allowed meaningful discussions that acted as catalyst for behavioural change. Additionally, promoting locally available health programs and resources and linking the participants to their primary care provider can boost positive outcomes to an individual and the community at large

• The challenge is to make this affordable and scalable, innovative approaches using artificial intelligence may also assist

**Supplementary Information:**

The online version contains supplementary material available at 10.1186/s12913-024-11335-y.

## Introduction

Globally, the escalating prevalence of pre-diabetes and diabetes represents a significant public health challenge, with recent diabetes prevalence estimates indicating that more than 400 million people are affected worldwide [1, 2]. This situation underscores the critical need for early detection and intervention [3], as timely diagnosis and management have been consistently shown to mitigate the progression of these conditions and reduce the burden of associated complications [4, 5].

In Western Sydney, a densely populated region characterized by cultural and socioeconomic diversity, the prevalence of diabetes is particularly alarming, with estimates suggesting that 13% of adults are affected [6]. This figure is notably higher than the national average, indicating a region-specific health crisis [7].

Historical data from Emergency Departments and General Practices in Western Sydney have highlighted a significant yet often undetected burden of pre-diabetes and diabetes [8, 9]. These findings point to the necessity of expanding our diabetes detection and prevention strategies beyond traditional healthcare settings.

In response to this need, our study introduces the Western Sydney Changing Diabetes program, a community-focused initiative aimed at improving the detection and management of diabetes in the local area. This pilot program, a collaborative effort involving Western Sydney Diabetes, the Workers Lifestyle Group, Novo Nordisk Pty Ltd, and other community groups, represents a shift from clinical to community-based diabetes detection and management. The program’s design enables direct engagement with the community, combining point-of-care testing with individualized advice and an education package, thereby linking participants back to their local healthcare providers.

This approach not only facilitates the early detection of prediabetes and type 2 diabetes but also empowers individuals with the knowledge and resources to make informed health decisions. By moving into community settings, we aim to address the gaps in diabetes care identified in clinical environments and extend our reach to underserved populations, thus enhancing the overall efficacy of diabetes management strategies [10, 11]. This is in part based on detection efforts that are becoming increasingly common globally, utilizing point-of-care testing to identify key high-risk populations in the community as this provides easy access in the local area, quick testing and results on the spot in a more relaxed setting [12].

## Methods

### Study design

This study represents a review of the data collected in the Western Sydney Changing Diabetes detection program. We collected basic demographic information from participants, including age, self-reported ethnicity, previous diabetes/prediabetes diagnoses, and identification as Aboriginal or Torres Strait Islander. The program targeted three distinct populations for its detection efforts: the Workers Lifestyle Group, attendees at the Tamil Arts and Culture Annual Festival, and visitors during the National Aboriginal and Islanders Day Observance Committee week. In all these platforms, we implemented the same basic testing methodology, as described below.

### Participant recruitment and ethics

Participants were recruited through on-site advertising and messaging from the participating organizations. The program was approved through the Western Sydney Diabetes initiative, and the data review was approved by the Western Sydney Local Health District (WSLHD) ethics committee. All participants provided informed consent, including consent for testing methodology, minimal demographic information, email contact for follow-up, information on their nominated General Practitioner (GP), and details on the planned use of the data.

### Target populations

1. **Workers Lifestyle Group**: A hospitality and lifestyle organization in Western Sydney which comprises 55,000 members across three sites, with testing conducted at the Blacktown Worker’s Club. The clientele primarily consisted of individuals from western Sydney, the majority of whom are over 60 years old and from culturally diverse backgrounds. The detection program was promoted through various channels, including social media and newsletters. Initially conducted in a remote club room, the program later moved to more visible locations, primarily at the Blacktown Worker’s Club location.
2. **Tamil Arts and Culture Annual Festival**: This cultural event, organized by community leaders, attracted approximately 1,500 attendees. A highly visible booth for the HbA1c testing was set up to raise community awareness and engagement.
3. **National Aboriginal and Islanders Day Observance Committee Week Event**: To address the high diabetes rates in Aboriginal and Torres Strait Islander communities, a Point-of-Care HbA1c testing station was set up at the WSLHD tent. Testing was conducted over three hours. The Good Tucker App [13], a culturally appropriate healthy eating application for Aboriginal people in Australia, was part of healthy lifestyle options package.

These populations were chosen as they are highly susceptible to diabetes due to socioeconomic and demographic factors, and these specific events were selected through collaboration with the local community. This sample was not intended to be representative of the entire population of Western Sydney.

### Process and testing

Participants provided informed consent, read a brief information sheet, and provided basic demographic information before proceeding to the testing area. A registered nurse collected a small blood sample, which was analysed using the Abbott Afinion machine with standard testing cartridges. This highly sensitive tool has demonstrated good clinical agreement in previous evaluations with traditional testing methods for HbA1c [14]. This procedure was conducted with trained nursing staff who had experience with the point-of-testing machines. During a three-hour timeframe, the team processed an average of 40 participants using two Abbott’s Afinion™ 2 Analysers for point-of-care HbA1c testing. Test results were communicated verbally and in writing, and participants falling within the prediabetes or diabetes range received corresponding educational materials.

### Based on the test results, all participants received education from a diabetes nurse educator and were directed to one of four pathways


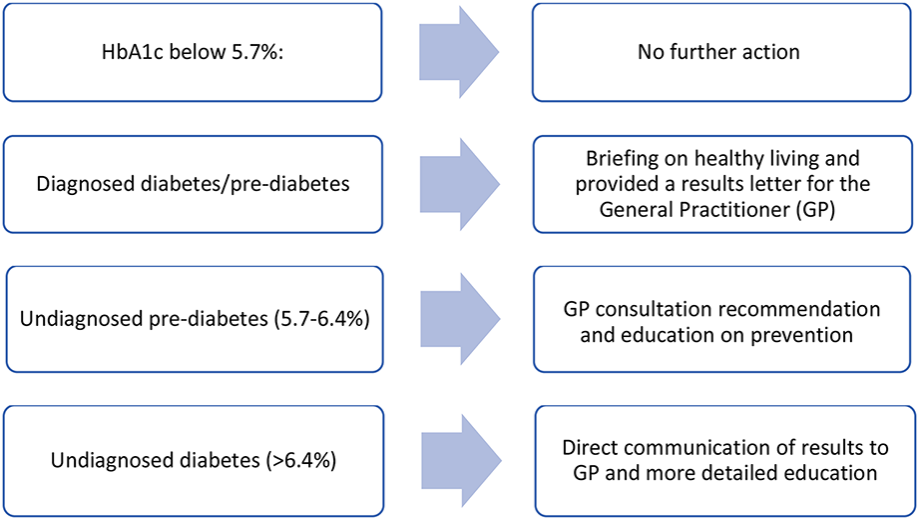


Participants without a GP were provided with a list of local practitioners. All participants received a local lifestyle education pack, with additional referrals for diabetes patients (optometrist for eye checks, podiatrist for foot reviews). Inclusion criteria were not specific, as testing was offered to all interested adults seeking to know their HbA1c levels.

### Collateral education pack

A comprehensive, localized educational pack was developed for this project. These materials are available see supplementary appendix and online on the WSD website [15]. The pack included information about local low-cost or free healthy living options, such as outdoor and living options, a diabetes-specific cookbook, details about local free exercise classes, cooking classes, and other activities, along with a range of other information for patients.

### Statistics

As this was a pragmatic pilot with no specific predetermined hypothesis, we did not conduct a sample size calculation. Patient characteristics and responses were summarized descriptively and analysed qualitatively. As there was no formal hypothesis, we did not conduct any hypothesis testing statistically. Data was descriptively analysed in Stata 15; graphs were created in Stata or Excel. Missing data (i.e. participants not giving a country of origin) was excluded from our descriptive analysis.

## Results

The detection work commenced in February 2023, following an initial trial run in December 2022, and data collection continued until the end of July 2023. During this period, a total of 510 people were tested at three different events: 24 individuals at NAIDOC week, 59 at the Tamil community event, and 427 at the Club, including 189 tested during Diabetes Week. The results for these demographics are presented in Table 1, categorized by test results.

**Table 1:** Demographic distribution by test results

	Normal (*n* = 221)	Prediabetes (*n* = 194)	Diabetes (*n* = 95)
**Age** (years)	56.8 (19.2)	66.0 (13.5)	67.9 (12.8)
**Ethnic Origin**:			
Aboriginal/Torres Strait Islands	21 (9.5%)	5 (2.6%)	3 (3.2%)
European/Caucasian	105 (47.5%)	74 (38.1%)	30 (31.6%)
Asian	27 (12.2%)	47 (24.2%)	25 (26.3%)
Pacific Islands	-	5 (2.6%)	1 (1.1%)
Indian/Subcontinent	40 (18.1%)	49 (25.3%)	28 (29.5%)
Africa	7 (3.2%)	1 (0.5%)	-
Middle East	11 (5.0%)	9 (4.6%)	5 (5.3%)
South America	1 (0.5%)	2 (1.0%)	-
**History of Diabetes**:			
No History	187 (84.6%)	104 (53.6%)	10 (10.5%)
Pre-Diabetes	22 (10.0%)	52 (26.8%)	11 (11.6%)
Diabetes	5 (2.3%)	38 (19.6%)	74 (77.9%)

### Distribution of HbA1c results for the total sample *n* = 510, using American Diabetes Association (ADA) criteria for prediabetes of 5.7–6.4%

**Fig. 1:**
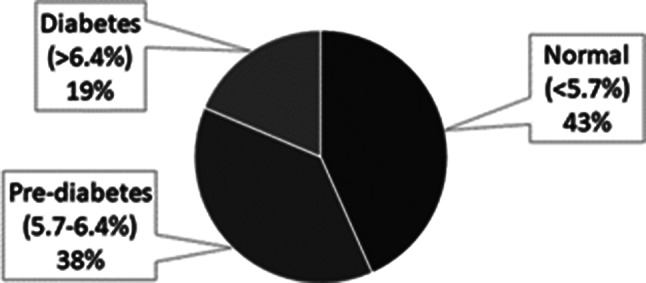
The distribution of HbA_1c_ results from the project (ADA criteria)

Of the individuals tested, 19% had HbA1c levels consistent with diabetes, and 38% with prediabetes (Fig. 1).

Additionally, some participants were already aware of their diabetes or prediabetes status. A total of 146 individuals either tested positive for diabetes in our program or had previously been diagnosed. Among these, 82 (56%) had well-controlled HbA1c levels under 7%, while 64 (44%) had suboptimal diabetes control (Table 2).

**Table 2:** Distribution of HbA1c results by prior history of diabetes/prediabetes

HbA1c (%)	Number	Percentage
< 7%	82	56%
7-7.9%	29	20%
8-8.9%	27	18%
9%+	8	5%
Total	146	100%

This included a total of 38 people who had diabetes but tested within the prediabetes range.

**Fig. 2:**
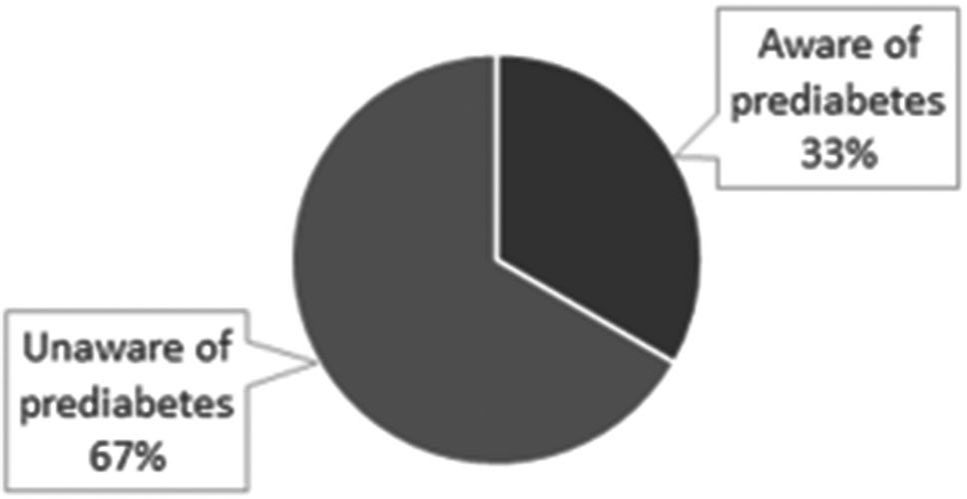
The proportion of people with HbA_1c_ 5.7–6.4% by prior knowledge of prediabetes (ADA criteria)

While most individuals with diabetes were aware of their condition, we identified 21 out of 146 (14%) individuals who were newly diagnosed. However, many participants with prediabetes were unaware of their condition. Among the 156 individuals testing within the prediabetes range without a previous diagnosis of diabetes, only 52 out of 156 (33%) had been informed of their prediabetes status by a healthcare provider and majority 104 (67%) were unaware (Fig. 2).

Within the prediabetes range, we also observed variability. Two definitions of prediabetes are currently in use, with different criteria. The previous tables have utilized the American Diabetes Association definition of HbA1c 5.7–6.4%, but we could also apply the European Association for the Study of Diabetes definition, which is HbA1c 6-6.4%. Among individuals with prediabetes, 94 (60%) had HbA1c levels of 5.7–5.9%, while 62 (40%) fell within the 6.0-6.4% range.

### Results of survey

After completing the detection program, we conducted an evaluation survey developed for this study. Out of 506 participants, 398 provided contact information and agreed to be surveyed. 3 basic questions were asked in the survey for which we have received 81 responses.

1. Did you find the diabetes detection useful?


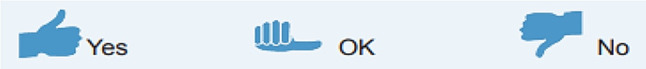


Participants feedback highlighted the convenience and effectiveness of the testing procedure, as well as the compassionate care provided by the healthcare team.

2. Did you find the conversations, booklets and information sheets provided to you useful?


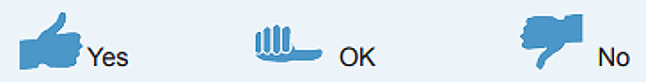


Participants feedback included the resources were useful and new, especially relevant, and locally available.

3. Based on the result provided to you, what actions did you take?

**Fig. 3:**
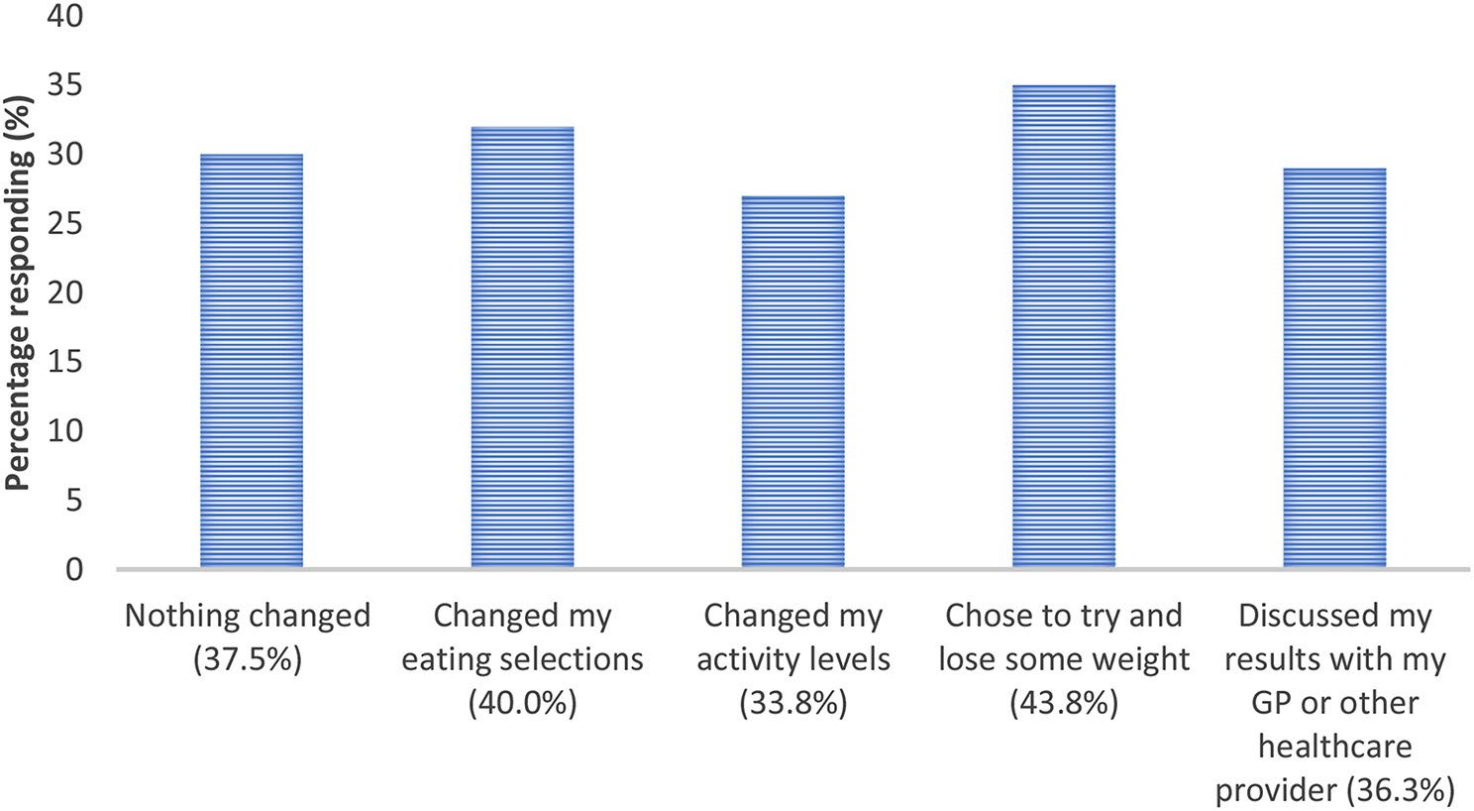
Actions reported for participants in the testing program (N=81)

Participants feedback emphasized the motivation derived from observing the results and implementing lifestyle adjustments.

## Discussion

In this detection program, we have demonstrated the possibility and feasibility of conducting diabetes and prediabetes detection in local communities [16]. This pilot program has shown that, even with limited staffing and minimal additional resources, collaborating with local community organizations can effectively engage vulnerable populations in diabetes detection using point-of-care testing [17]. This approach is invaluable for reaching underserved populations who often face barriers to accessing traditional healthcare services and may find utility in lower-resource settings where point-of-care testing can enhance detection efforts. Moreover, we have found that testing of this nature is well-received by the community, with participants reporting its usefulness and a willingness to change their behaviour based on test results.

Our pilot detection program highlights the potential of community-based approaches in high-risk areas. Although limited in scope, the detection rates in this study closely mirror those found in hospital emergency departments and general practices within the same community, suggesting a consistent pattern of diabetes prevalence [8]. This underscores the high burden of disease in this chronically underserved, low socioeconomic status area of Australia and emphasizes the need for detection efforts in local communities alongside traditional healthcare settings. However, there are challenges associated with this testing program. While point-of-care testing is more cost-effective than traditional HbA1c testing, the setup of this project required a significant number of staff to run the community testing station and provide education to participants [3]. This mirrors similar efforts and highlights that certain demographic segments of the population may yield unreliable results with point-of-care testing modalities [3]. Scalability may be a concern for larger groups, depending on the availability of staffing in broader detection efforts.

Scalability could potentially be addressed through the application of AI and machine learning techniques for diabetes education in community settings by addressing health literacy needs [16, 17]. A trained Large Language Model might be able to provide basic diabetes education for participants, reducing the requirement to have diabetes educators spending a large part of their time discussing options with participants. There are several educational tools utilizing LLMs that are being developed in Australia by groups such as Diabetes Australia that might be applicable to this testing program.

The program’s visibility, particularly at the club foyer and cultural festival booths, played a vital role in participant engagement, as did the development of local materials focusing on providing free and low-cost exercise and weight management options for people in Western Sydney. Building relationships with local groups such as the Workers Lifestyle Group and the Tamil community in Western Sydney was crucial for the success of this pragmatic, localized effort.

Many participants were unaware of their prediabetes status, and some unaware of diabetes. Undiagnosed diabetes is a well-researched and understood issue, with data from Australia indicating that a large minority of those with diabetes are living undiagnosed [18]. However, undiagnosed prediabetes is less well-understood, due to limitations in screening for the condition. Our results indicate that screening for prediabetes may be necessary at a broader scale in communities with high rates of diabetes to assist with early intervention to prevent diabetes long-term.

The advancement of point-of-care testing, exemplified by the Abbott Afinion device, has been instrumental in enabling rapid, sensitive results and immediate education based on HbA1c findings for individuals with prediabetes and diabetes [3]. This contrasts with other detection efforts in Australia that rely on traditional blood tests, necessitating additional personnel for follow-up once results are available [9].

It is essential to recognize that this type of detection effort extends beyond the individuals seen at the booth on the day. Screening programs for type 2 diabetes may have broader societal benefits [19], drawing in traditionally uninvolved participants, such as the families of individuals with diabetes and prediabetes. This allows for a much wider reach than the number of tested individuals might suggest, ultimately impacting entire communities that may otherwise have limited contact with the healthcare system and limited understanding of their long-term risk.

Detection of diabetes in underserved communities is of paramount importance. Large, randomized trials have demonstrated that early detection of prediabetes and diabetes can lead to actions that reduce the long-term risk of developing complications [20]. Many communities, particularly those from culturally and linguistically diverse backgrounds, face a significantly higher risk of diabetes due to a range of factors affecting their physical health [21]. Our approach offers a novel and innovative way to engage with traditionally unrecognized high-risk groups and initiate conversations about reducing their long-term health risks.

### Limitations

This was a pilot program conducted in a community. Thus, there are many sources of potential bias that may have impacted the study’s results. The sample size and methodology mean that we cannot draw conclusions about the population of Western Sydney, nor the entire communities from which our participants were drawn. We have not collected long-term data looking at the impact of the intervention on individuals, nor any objective data such as HbA1c results at follow-up demonstrating clinical benefits. In addition, we did not recruit a control group. This was a limited pilot initiative that aimed to improve testing and provide outreach to high-risk communities, but further clinical trials are necessary to address questions of effectiveness and generalizability.

## Conclusion

In conclusion, this project demonstrates the feasibility of an integrated collaboration to improve diabetes detection efforts in local communities [16, 17]. It serves as a foundation for broader programs that involve large segments of the population. Continuous refinement and innovation are essential for enhancing the program’s efficiency and scalability, ultimately benefiting the wider community.

### Electronic supplementary material

Below is the link to the electronic supplementary material.

Supplementary Material 1

Supplementary Material 2

Supplementary Material 3

Supplementary Material 4

Supplementary Material 5

Supplementary Material 6

Supplementary Material 7

## Data Availability

Data is not available for this study, as it covers potentially reidentifiable information from participants. If individuals wish to request data for this study, please contact author JS.
