# Supplementary material for: Integrating point-of-care diabetes detection with lifestyle counselling in community settings: outcomes from Western Sydney, Australia
Source: BMC Health Serv Res. 2024 Aug 13;24:926. doi: 10.1186/s12913-024-11335-y (PMC11323375; doi:10.1186/s12913-024-11335-y)

**Thank you for attending the Western Sydney Diabetes Detection Program**

We want to hear from you, as your feedback is extremely valuable

Your response will not be identified

Please take a minute to answer a few questions

1. Did you find the diabetes detection program useful for you?


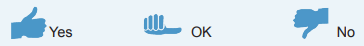


Tell us in a few words what you thought about the experience?

1. Did you find the conversations, booklets and information sheets provided useful?


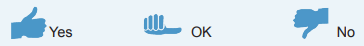


Tell us in a few words what you thought about the information given?

1. Based on the result provided to you, what actions did you take?

- Nothing changed
- Changed my eating selections
- Changed my activity levels
- Chose to try and lose some weight
- Discussed my results with my GP or other healthcare provider?

Tell us in a few words what you did differently?

Thank you for taking time to answer this survey!


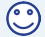

Supplement: Supplementary file 1 — Supplementary Material 1 [file 12913_2024_11335_MOESM1_ESM.docx]
