# Supplementary material for: Integrating point-of-care diabetes detection with lifestyle counselling in community settings: outcomes from Western Sydney, Australia
Source: BMC Health Serv Res. 2024 Aug 13;24:926. doi: 10.1186/s12913-024-11335-y (PMC11323375; doi:10.1186/s12913-024-11335-y)
Supplement: Supplementary file 2 — Supplementary Material 2 [file 12913_2024_11335_MOESM2_ESM.pdf]

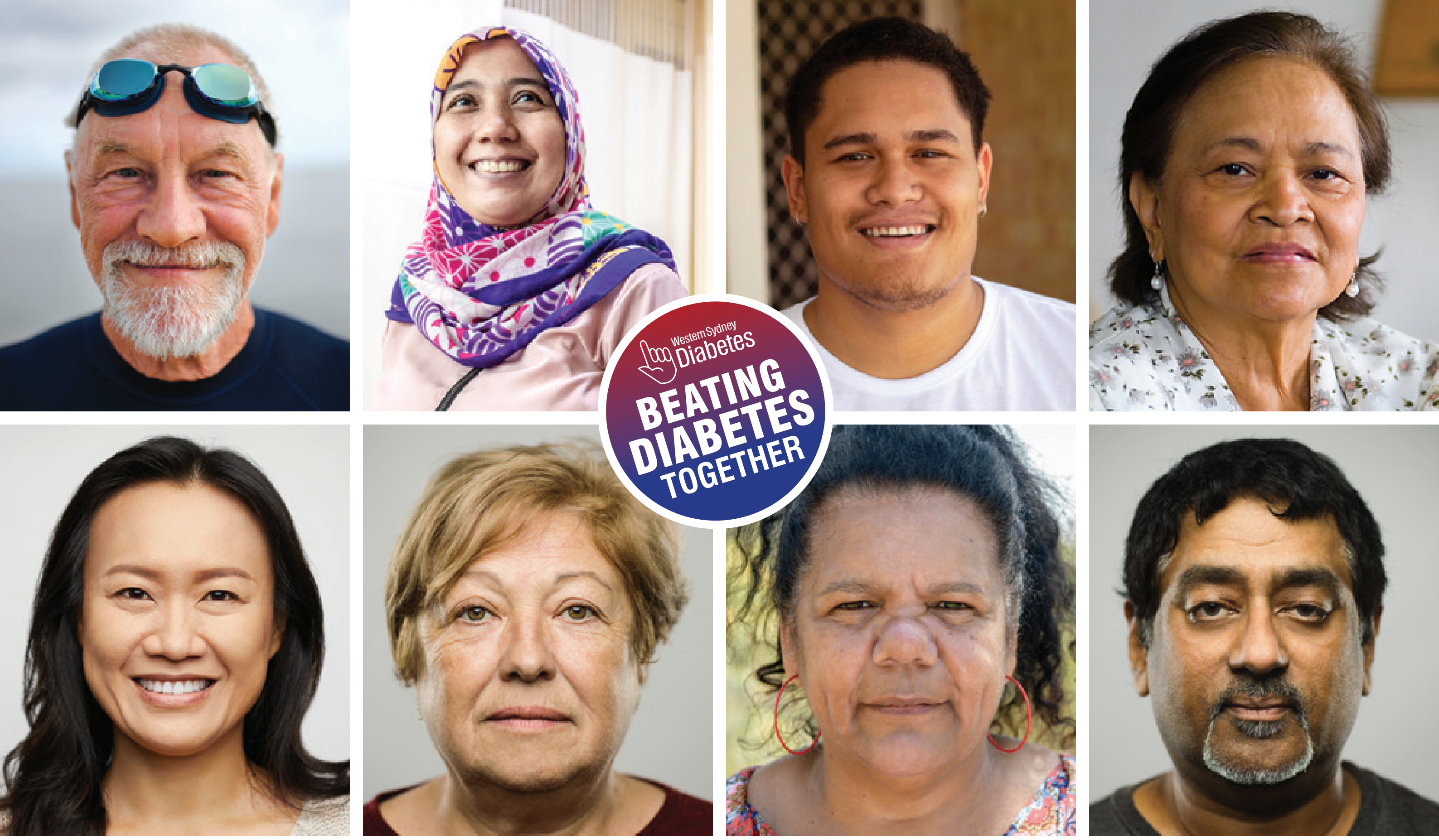

Western Sydney Diabetes

ARE YOU AT RISK OF DIABETES?

Is this a member of your family?

Western Sydney is a diabetes hotspot, with rates of diabetes more than double that of Sydney's northern beaches and eastern suburbs.

50% OF PEOPLE IN WESTERN SYDNEY ARE AT RISK OR HAVE TYPE 2 DIABETES

1 IN 2 PEOPLE DON'T KNOW THEY HAVE TYPE 2 DIABETES

Should you have the HbA1c test?

Yes! Especially if you are an adult in western Sydney with one or more of these risk factors:

- **A family history of diabetes**
- **Have a Pacific Island, Indian subcontinent, Chinese, South East Asian, Middle Eastern, African, Aboriginal or Torres Strait Island background**
- **Had diabetes during a pregnancy**
- **Are overweight or obese**
- **Don't do any regular physical activity.**

A blood test can tell you if you are at risk of developing Type 2 diabetes. This is covered by Medicare and can be done once a year – ask your GP.

phn WESTERN SYDNEY
An Australian Government Initiative

pwc

diabetes australia

NSW GOVERNMENT

The HbA1c Test and YOU

Western Sydney Diabetes
BEATING DIABETES TOGETHER

What is the HbA1c test?

- HbA1c stands for glycated haemoglobin and is one of the ways we find out how much sugar is in your blood.
- It is measured from a single blood sample and does not need any special preparation before testing.
- This test tells us what your average sugar levels are over the last 3 months.

Why should I have the test?

The amount of sugar in your blood tells us if you have diabetes or pre-diabetes. Early diagnosis and treatment can help you to reverse pre-diabetes and avoid complications if you have diabetes.

How do I get tested?

Your doctor can send you to get a HbA1c test.

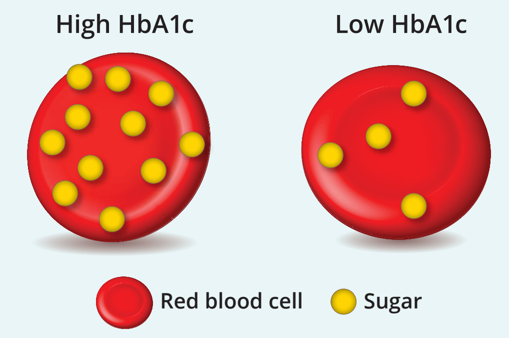

Is there a cost?

Medicare covers one HbA1c test each year to check for diabetes. **There is no cost.**

What does the test result tell me?

NO DIABETES
HbA1c =
less than 5.7%

PRE-DIABETES
HbA1c =
5.7 to 6.4%

DIABETES
HbA1c =
6.5% or above

Great news!

Keep choosing healthy food options, check your meal portion sizes and be physically active on most days to stay in the No Diabetes range.

What next?

If you lose weight and become physically active for 30 minutes on most days, you may be able to return to the No Diabetes range.

What next?

You will need to have a second blood test to confirm you have diabetes. If you have diabetes, talk to your doctor about a care plan. Your doctor can also register you with the National Diabetes Services Scheme (NDSS).

Talk to your doctor about the HbA1c test

Visit www.westernsydneydiabetes.com.au
