# Supplementary material for: Integrating point-of-care diabetes detection with lifestyle counselling in community settings: outcomes from Western Sydney, Australia
Source: BMC Health Serv Res. 2024 Aug 13;24:926. doi: 10.1186/s12913-024-11335-y (PMC11323375; doi:10.1186/s12913-024-11335-y)
Supplement: Supplementary file 3 — Supplementary Material 3 [file 12913_2024_11335_MOESM3_ESM.pdf]

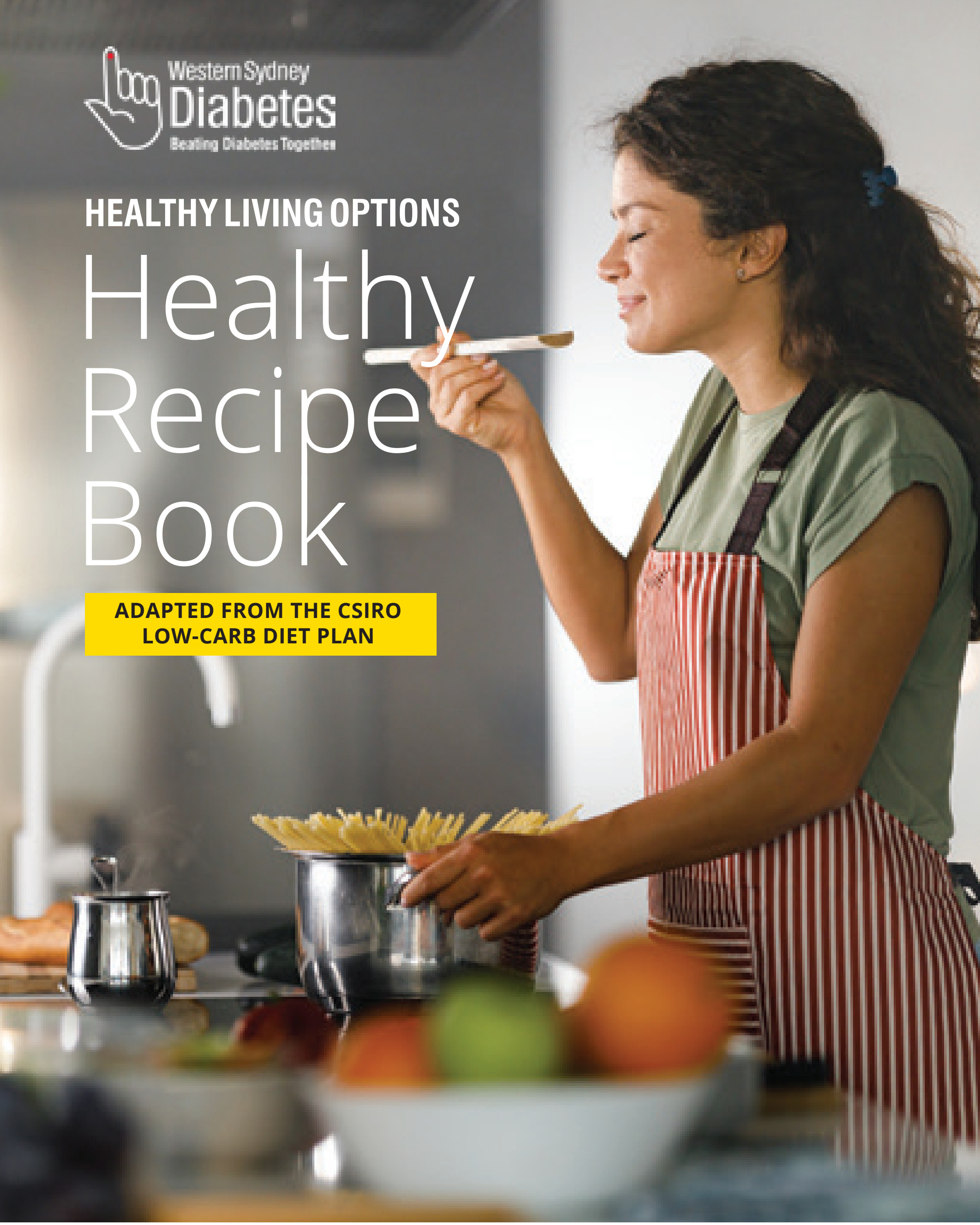

Western Sydney Diabetes
Beating Diabetes Together

HEALTHY LIVING OPTIONS

# Healthy Recipe Book

**ADAPTED FROM THE CSIRO LOW-CARB DIET PLAN**

phn WESTERN SYDNEY
An Australian Government Initiative

pwc

diabetes australia

NSW GOVERNMENT

# About this booklet

Improving the overall health and wellbeing of the people of western Sydney is the aim of Western Sydney Diabetes and our Alliance partners. We want to provide our residents with simple and practical ways to help keep themselves, and their families, healthy.

Our diet and weight play an important role in helping us remain healthy to enjoy the fun things in life. But with all the information and confusing messaging about food and ingredients, it's often difficult to confidently make good food choices. To help Australians improve their diets, the CSIRO has used the latest scientific evidence to develop and test the CSIRO Low-Carb diet.

The CSIRO Low-Carb diet is a prescriptive, nutritionally complete diet and lifestyle plan, which research has shown to be highly effective in achieving sustained long-term weight loss, as well as reducing the risk of chronic diseases such as heart disease and diabetes.

This diet has already helped many Australians to better manage their diabetes, often with less medication, and in some cases achieve type 2 diabetes remission. It is recommended to follow the lifestyle plan with support from your doctor and health care team, especially if you have diabetes as your medication requirements may change as your diabetes control improves.

We wanted to give you a 'taster' of what the diet is all about and tempt you to find out more with a small selection of the delicious recipes you'll find in the CSIRO Low-Carb Diabetes Diet and Lifestyle Solution books. Please visit our website for more information on healthy living options: www.westernsydneydiabetes.com.au

**Janine Dawson**

Primary Prevention Manager
Western Sydney Diabetes

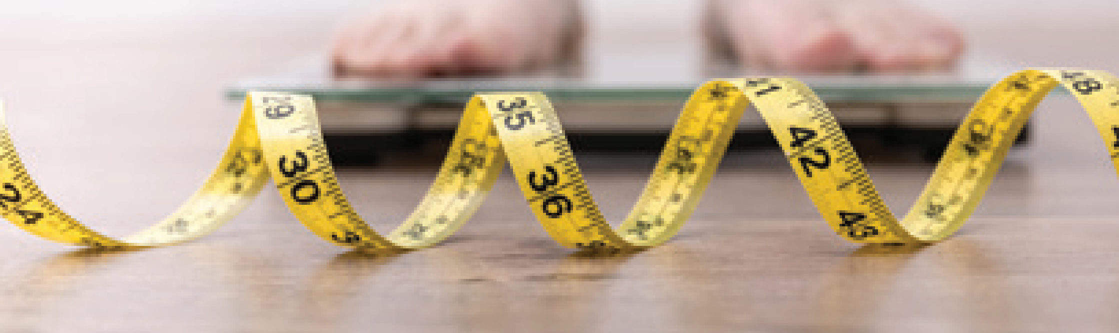

# Contents

**About this booklet ..... 2**

What the diet is about 4
Research results 5
Why & how the diet works 6
How to get started 8

**Recipes – Low Carb Diet Easy 100 ..... 10**

Beef and mushroom bolognese with broccoli 10
Beef nachos bowl with quick guac 12
Chicken with lemony butter beans and garlicky greens 14
Fetta, spinach and sun-dried tomato breakfast muffins 16
Fish with artichoke, cherry tomatoes and thyme 18
Prawn curry with cauliflower rice 20
Tomato-braised lamb shanks with crushed cauliflower 22

**Recipes – Low-Carb Diabetes Diet & Lifestyle Solution ..... 24**

Asparagus and salmon spaghetti 24
Beef fillet steaks with korma vegetables 26
Chargrilled chicken and mushrooms with avocado lentils 28
Ginger chicken and bok choy bake 30
Greek roasted lamb cutlets and veggies 32
Rosemary potato and chicken bake 34
Tandoori salmon tray bake 36

**The CSIRO books – full versions ..... 38**

# What the diet is about

The CSIRO Low-Carb diet is a prescriptive, nutritionally complete eating and lifestyle plan, which is lower in carbohydrates and higher in proportions of protein and healthy (unsaturated) fats – combined with a tailored home-based exercise program.

Clinical research has shown this diet and lifestyle approach is highly effective in achieving sustained long-term weight loss and compared to a traditional higher carbohydrate, low fat diet is more effective in controlling blood sugar levels, reducing risk factors for heart disease and reducing the requirements of diabetes medication.

In fact, the CSIRO Low-Carb Diabetes Diet & Lifestyle Solution targets and markedly reduces all the modifiable risk factors for diabetes and heart disease, all at the same time, in one simple solution.

The CSIRO Low-Carb Diabetes Diet and Lifestyle Solution is a plan designed to help individuals with pre-diabetes and type 2 diabetes to understand the condition and take practical steps to comprehensively improve their health and wellbeing through a complete diet and exercise solution that have been scientifically developed and proven.

If you are going to change your lifestyle in any way, you should do this in close consultation with your healthcare team. Scientific research has shown that a low-carb diet is very effective in improving blood glucose control and reducing diabetes medication requirements. This is why it's so important to involve your doctors so that they can monitor and adjust your medication requirements as necessary to ensure it remains at the correct level. Taking too much medication can cause your blood glucose levels to fall too low, which can be quite dangerous.

# Research results

Results of a clinical trial showed that after following this diet for 2 years the average person with type 2 diabetes had achieved:

- a sustained weight loss of 7% (7kg);
- a reduction in blood pressure, fasting blood glucose and HbA1c levels;
- a reduction in blood levels of low-density lipoprotein – LDL (bad) cholesterol levels;
- improved mood and quality of life, mental function and performance.

In addition, the research showed that the CSIRO Low-Carb diet, when compared with the traditional high-carbohydrate, low-fat diet resulted in:

- a more stable blood glucose profile during the day, thereby lowering the risk of complications associated with diabetes;
- a greater reduction in the requirements for diabetes medication;
- improved blood cholesterol levels by reducing triglyceride levels and increased high-density lipoprotein – HDL (good) cholesterol levels.

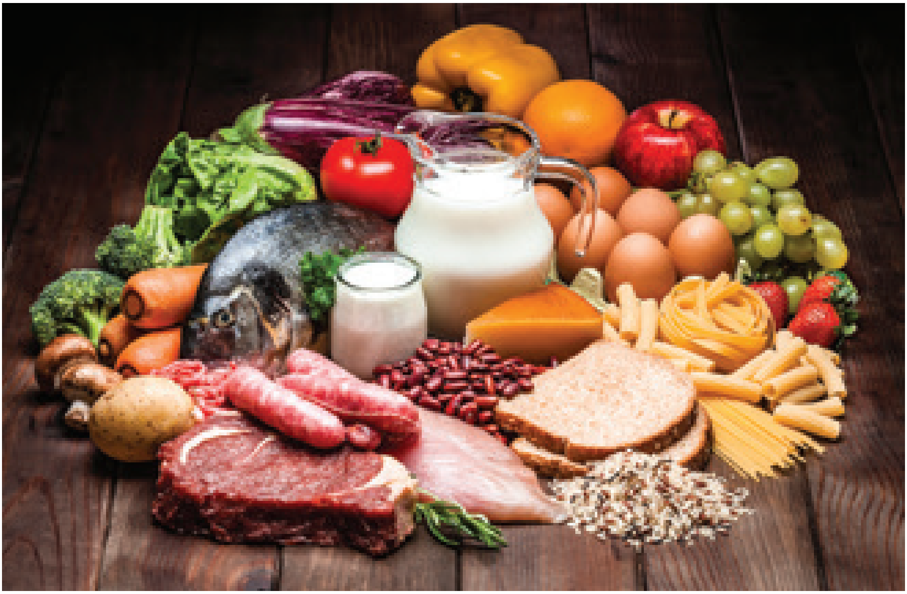

# Why & how the diet works

The CSIRO Low-Carb Diet works so well because it is based on the latest scientific evidence and nutrition principles of healthy eating that uses a select combination of everyday whole foods proven to provide numerous health benefits including weight and blood glucose control, metabolic health, and wellbeing to maximise the health benefits that can be gained – particularly for people with pre- diabetes and type 2 diabetes. The diet does this by:

- Focusing on good-quality carbohydrates (low-glycaemic index (GI) foods and high in fibre) and limiting the total amount of high carbohydrate foods. Eating foods with higher amounts of carbohydrate – such as breads, cereals, rice, pasta, potatoes, many fruits, and food high in sugar can cause the blood glucose levels to rise rapidly that increases the risk of type 2 diabetes and heart disease. By simply reducing the total amount of carbohydrate rich foods in your diet and meals can help to reduce your blood glucose levels and risk for diabetes.

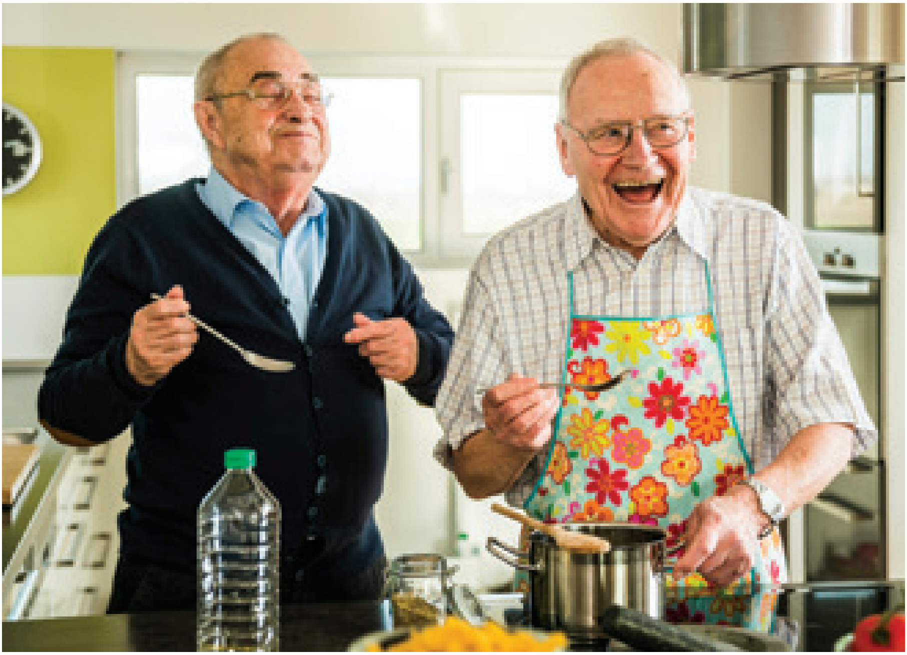

- Increasing the amount of 'healthy' unsaturated fats. Research shows that instead of eating a low-fat diet, eating high amounts of healthy 'unsaturated' fats found in food such as nuts, seeds, oils (olive, canola, sunflower, peanut, and sesame), avocados, olives, and oily fish such as salmon and tuna improves heart health and reduces risk factors for heart disease. Having higher amounts of healthy fats in meals also helps to further reduce and blunt the blood glucose rise of any carbohydrate in the meal.
- Increasing the amount of quality dietary protein. This offers several benefits for weight control and managing type 2 diabetes. Eating higher amount of quality protein in your diet can:
  - Improve body composition by maintaining higher amounts of lean muscle tissue, particularly when combined with exercise training. This will maintain a higher metabolic rate making it easier to maintain a lower body weight;
  - Burns more energy from processing and digesting food, also making it easier to maintain a healthy body weight;
  - Help to suppress appetite and the amount of food we eat, as dietary protein make you feel full, again making it easier to maintain a healthier and lower body weight;
  - Helps to further reduce and blunt the rise in blood glucose levels from carbohydrate in the meal.
- Remaining nutritionally complete and providing adequate amounts of essential vitamins, minerals, dietary fibre and trace elements because the CSIRO Low-Carb diet includes foods from all major food groups.
- Focusing on eating low-energy, nutrient dense whole foods and limiting the intake of high-energy nutrient poor discretionary food which is the foundation of all healthy eating plans.

# How to get started

**Step 1** – Once you have your "CSIRO Low-Carb Diabetes Diet and Lifestyle Solution Book", the next step is to become familiar with what is in the book. Follow the next few steps to get started.

**Tip**: Page 82-83 (Diabetes Diet & Lifestyle Solution Book) will provide a quick visual guide of a day on the diet, use this to see the types of everyday foods you will eat as part of the plan.

**Step 2** – Calculate your daily energy (kilojoule) needs to personalise your diet plan. Where do I find this information? See Diabetes Solution book page 74-76. (Diabetes & Lifestyle Solution Book).

**Tip**: The plan provides 4 energy level options and will help to achieve a nutritionally balanced eating plan. If you have a BMI > 25, selecting an energy level to help you achieve weight loss is desired. See page 26 (Diabetes Lifestyle Solution Book) to learn how to calculate BMI.

**Step 3** – Identify the energy level of the CSIRO Low Carb Diet that will meet your needs. This will tell you how many units of each food unit you will need to eat each day to achieve your energy and nutrient requirements Where do I find the information? See book page 71 (Diabetes Solution Book).

**Tip**: The book provides 4 energy levels and will help you to achieve a nutritionally balanced eating plan.

**Step 4** – To choose the foods and amounts right for you, read about the food groups and food units. Here you can see what types of foods you will be eating. But more importantly, how much food you will need to make up 1 food unit and how many of these units you will need each day.

Eating the correct number of food units for your energy level means that you will following the eating plan correctly. It is that simple!!! Where do I find the information? See table on Page 71, 79-81. (Diabetes Diet & Lifestyle Solution Book).

**Tip**: Review pages 79-81 "your daily food guide'. Pages 82-83 provide a visual guide of 'one day on a diet' to help you pull all the information together. If you need more help you can speak to your GP about how to get dietary support including a referral to an Accredited Practising Dietitian.

**Step 5** – Each recipe shows how many food units of each food group are in one serve of the recipe. These are here for you to easily track your units across the day. Keeping track of your daily food units is a simple way to make sure your are getting a balanced dietary intake. Where do I find the information? Top of each recipe next to the recipe title.

**Tip**: This eating plan helps you to achieve between 50 to 70g of carbohydrates a day. By planning your daily or weekly menus using the recipes provided, you will be able to easily reach your required food units allowance.

**Step 6** – Become familiar with the CSIRO exercise program to commence your exercise plan. *If you have not exercised before, speak with your GP for a Referral to an Accredited Exercise Physiologist who will assess your readiness to commence and guide you through the exercises in the book. Where do I find the information? See book page 266-279. (Diabetes Diet & Lifestyle Solution Book).

**Tip**: Start at a level of exercise that you're comfortable with increasing your exercise intensity and duration as you start to feel fitter and more confident with the types of exercise you are doing. The exercise intensity rating (page 268) is a useful guide.

**Step 7** – Monitor your progress and success using the information provided in the book in consultation with your health care team.

Another way to self-monitor and keep yourself on track is to use food and exercise diaries. Visit the website below to find more information including weekly shopping lists for following the meal plans in your book, or weekly food checklists and an exercise training diary. www.csiro.au/lcd

**Tip**: Through your journey seek support from your health care team including your GP or practising dietitian or exercise physiologist to assist you to achieve the principles of the book and lifestyle plan.

**For full details refer to the Diet & Lifestyle Solution Book**

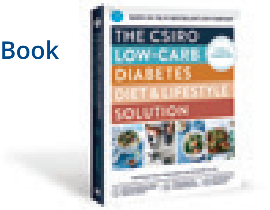

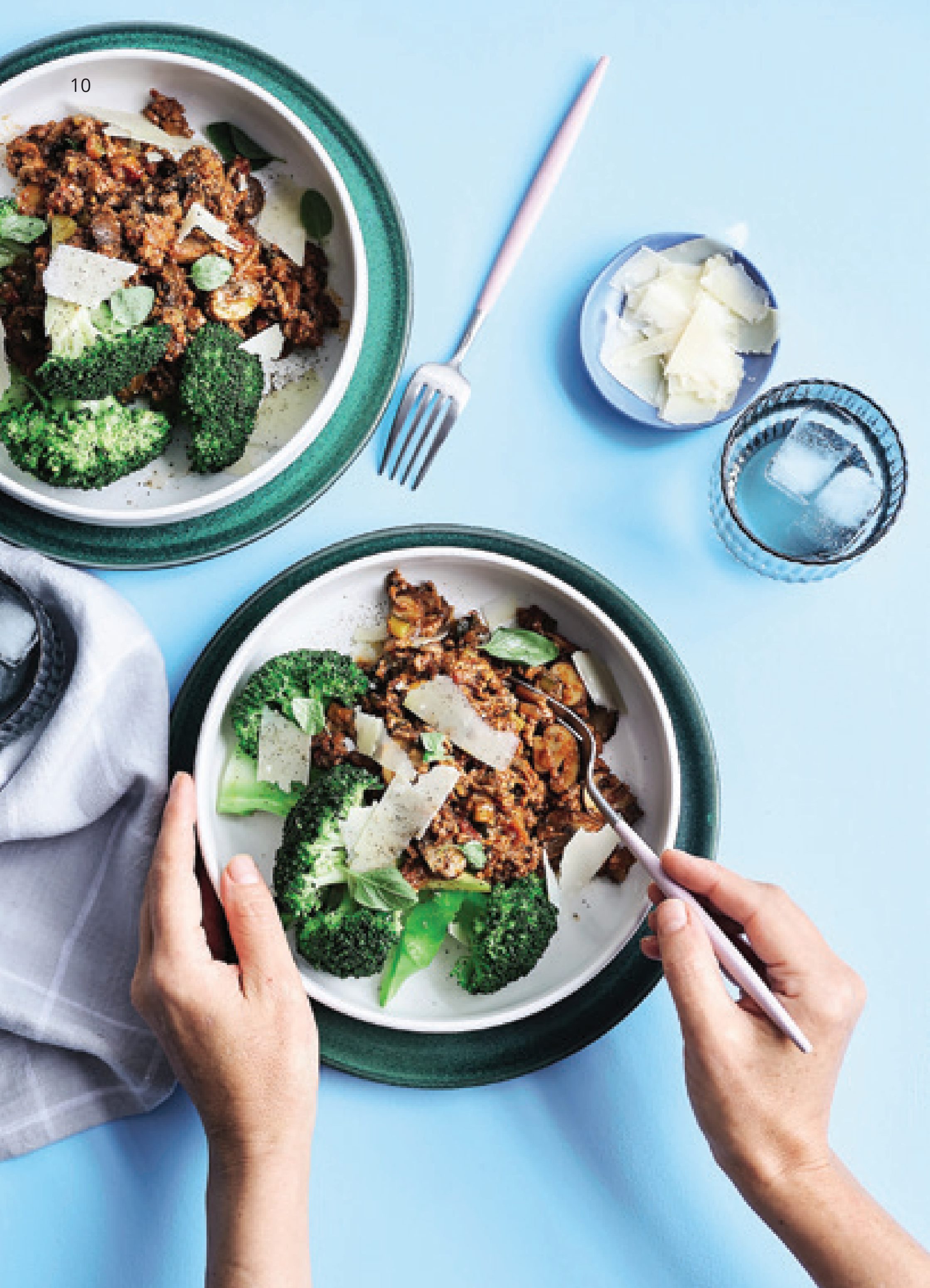

| UNITS PER SERVE: | |
|---|---|
| Breads, Cereals, Legumes, Starchy Veg | 0 |
| Dairy | 1 |
| Lean Meat, Fish, Poultry, Eggs, Tofu | 1.5 |
| Low–Moderate Carbohydrate Veg | 3 |
| Healthy Fats | 2 |

# Beef and Mushroom Bolognese with Broccoli

CARBS 9g PER SERVE

**SERVES: 4 | PREPARATION: 10 MINUTES | COOKING: 60 MINUTES**

1 tablespoon extra virgin olive oil
1 onion, finely diced
1 zucchini, finely diced
600 g lean beef mince
300 g sliced Swiss brown mushrooms
5 cloves garlic, crushed
½ bunch oregano, leaves picked (or 2 teaspoons dried oregano)
3 tablespoons tomato paste (no added sugar or salt)
2 tablespoons red wine vinegar
1 cup (250 ml) salt-reduced chicken stock (see notes)
400 g tinned diced tomatoes (no added sugar or salt)
3 heads broccoli, stems and florets chopped into 2 cm pieces separately
60 g parmesan, shaved

1. Place half the olive oil in a large, deep frying pan over medium–high heat and cook the onion and zucchini, stirring, for 5 minutes, until translucent and soft. Add the beef mince and cook, breaking it up with a wooden spoon, for 3–4 minutes until mostly browned. Add the remaining olive oil, the mushrooms, garlic and oregano and cook, stirring, for 3–5 minutes until the mushrooms are wilted and the excess liquid has evaporated.
2. Add the tomato paste to the pan and stir it through for 1 minute until it sticks to the bottom of the pan. Add the red wine vinegar and cook for 1 minute, using a spatula to scrape up any bits caught on the bottom of the pan. Add the chicken stock and diced tomatoes and bring to the boil. Reduce the heat to low–medium and cook, partially covered, for 30–40 minutes, or until thickened. Add ½ cup (125 ml) of water at a time, as needed, to keep the sauce from sticking to the bottom of the pan and thickening too much.
3. Once the bolognese has 10 minutes to go, bring a saucepan of water to the boil. Add the broccoli stems and cook for 3 minutes, then add the florets and cook for a further 2 minutes, until softened. Drain.
4. Divide the broccoli and bolognese between four bowls and serve with the parmesan and some freshly ground black pepper.

## Notes

Commercial chicken stock is usually very high in sodium. We recommend shopping for 'chicken broth', which is a similar product you can find in the refrigerator section of supermarkets and grocers. Broth is sold in small batches and contains less sodium. Check the label to ensure your stock contains less than 50 mg sodium per 100 g. Alternatively, if you'd like to make your own homemade stock, see the recipe on page 131.

To freeze, divide the bolognese and broccoli into individual portions and freeze in airtight containers for up to 3 months.

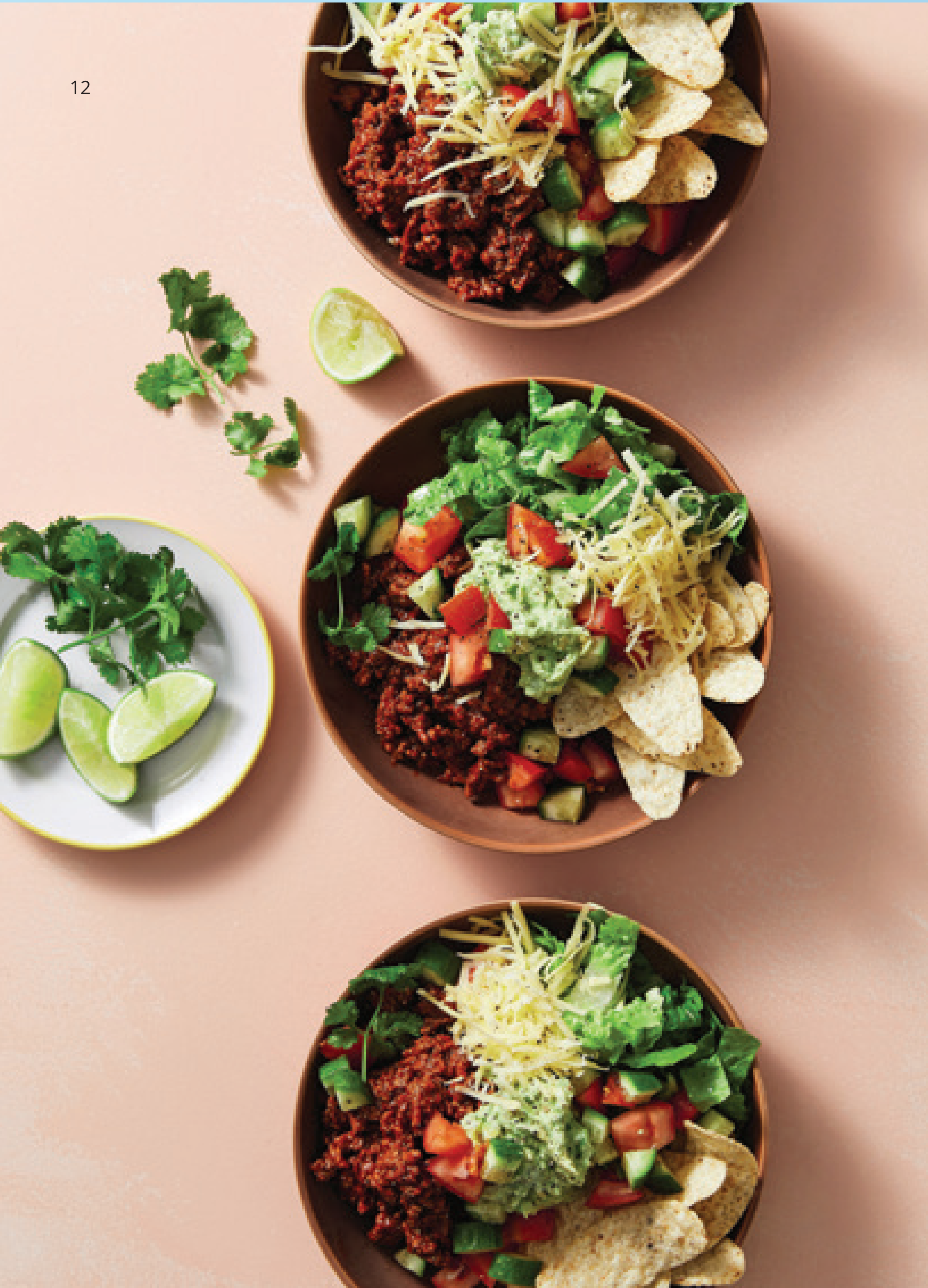

| UNITS PER SERVE: | |
|---|---|
| Breads, Cereals, Legumes, Starchy Veg | 1 |
| Dairy | 1 |
| Lean Meat, Fish, Poultry, Eggs, Tofu | 1.5 |
| Low–Moderate Carbohydrate Veg | 2 |
| Healthy Fats | 2.5 |

# Beef Nachos Bowl with Quick Guac

CARBS 20g PER SERVE

SERVES: 4 | PREPARATION: 10 MINUTES | COOKING: 10 MINUTES

2 teaspoons extra virgin olive oil
600 g lean beef mince
1 tablespoon smoked paprika
2 teaspoons ground cumin
2 teaspoons ground coriander
3 cloves garlic, crushed
1 tablespoon tomato paste (no added sugar or salt)

## QUICK GUAC

1 avocado
1 tablespoon low-fat natural yoghurt
Juice of 1 lime
½ cup coriander leaves, finely chopped

## TO SERVE

2 tomatoes, diced
2 baby cos lettuces, shredded
1 Lebanese cucumber, diced
100 g unsalted corn chips
80 g grated cheddar
Coriander sprigs

1. Heat the olive oil in a large frying pan over medium–high heat. Add the beef mince and cook, breaking it up with a wooden spoon, for 4–5 minutes or until brown and cooked through. Add the paprika, cumin, ground coriander and garlic and stir through for 1 minute or until fragrant. Add the tomato paste and ⅓ cup (80 ml) water and stir for 1–2 minutes until well combined. Set aside.
2. To make the quick guac, mash the avocado in a bowl with a fork. Add the yoghurt, lime juice and chopped coriander and stir until combined.
3. Divide the beef between four bowls and serve with the tomato, lettuce, cucumber, corn chips, cheese, corainder sprigs and a dollop of guac.

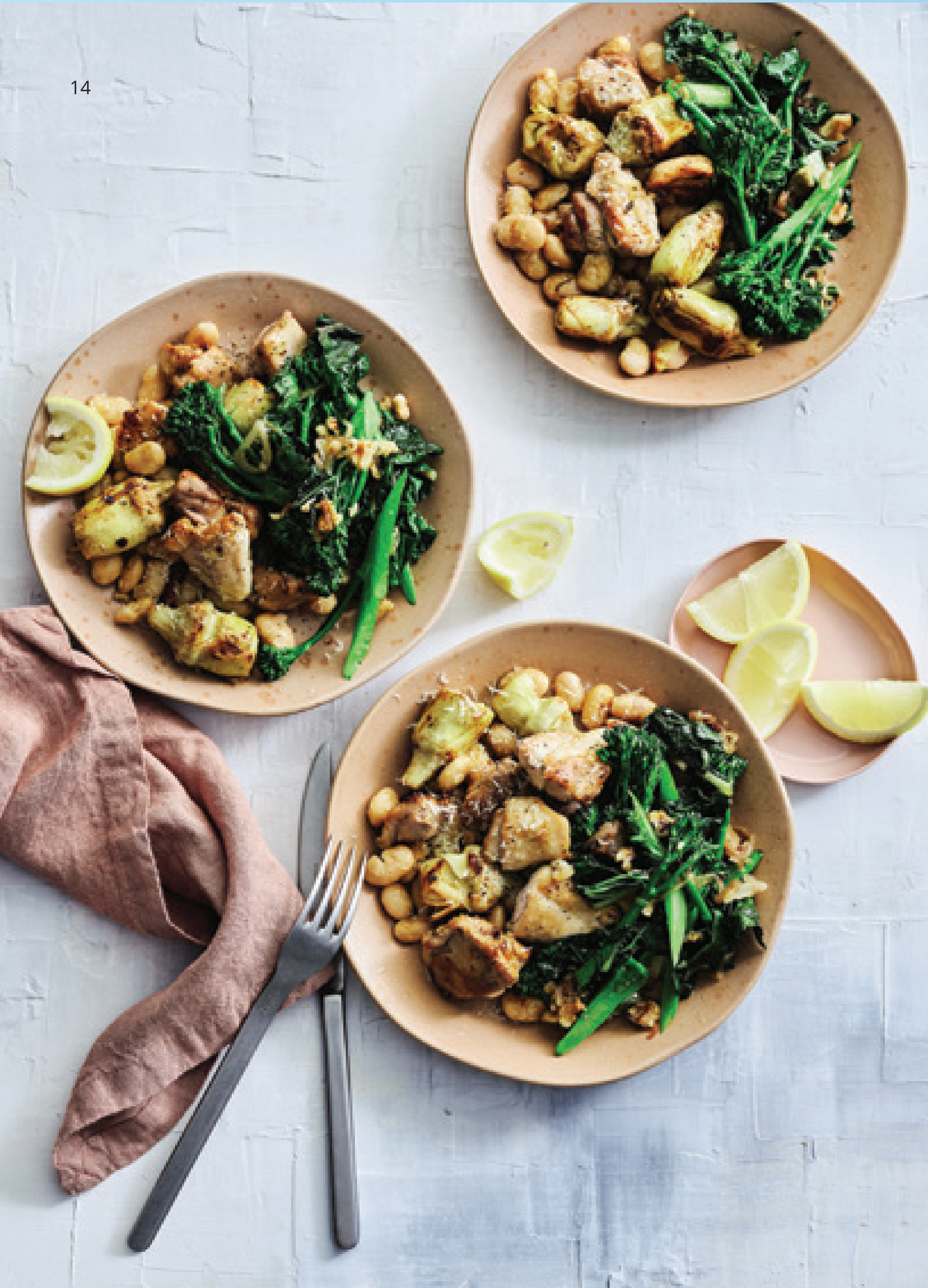

| UNITS PER SERVE: | |
|---|---|
| Breads, Cereals, Legumes, Starchy Veg | 1 |
| Dairy | 0.5 |
| Lean Meat, Fish, Poultry, Eggs, Tofu | 1 |
| Low–Moderate Carbohydrate Veg | 3 |
| Healthy Fats | 3 |

# Chicken with Lemony Butter Beans and Garlicky Greens

CARBS 20g PER SERVE

**SERVES: 4 | PREPARATION: 15 MINUTES | COOKING: 15 MINUTES**

2 tablespoons extra virgin olive oil
400 g chicken thigh fillets, visible fat removed, chopped into 1 cm pieces
200 g tinned butter beans, drained and rinsed
300 g tinned artichoke hearts, drained and rinsed
Juice of 1 lemon
40 g parmesan, shaved

## GARLICKY GREENS

2 teaspoons extra virgin olive oil
4 golden shallots, thinly sliced
2 cloves garlic, thinly sliced
2 tablespoons crumbled walnuts
1 bunch broccolini, stems sliced in half lengthways
½ cup (125 ml) salt-reduced chicken stock (see note)
600 g kale or silverbeet, stalks removed and leaves roughly chopped

1. Heat 1 teaspoon of the olive oil in a large frying pan over medium–high heat. Add the chicken and cook for 4 minutes – not tossing, but leaving the chicken to brown on the bottom of the pan – then flip and cook for another 3 minutes, until browned and cooked through. Add the butter beans, artichoke, remaining olive oil and lemon juice and toss just until warmed through. Transfer everything to a plate and sprinkle with the parmesan and a pinch of freshly ground black pepper.
2. To make the garlicky greens, add the olive oil to the same frying pan over medium–high heat. Add the shallots and garlic and cook for 3 minutes until golden. Add the walnuts and continue cooking for 2–3 minutes until nicely browned and crisp. Transfer to a bowl and set aside.
3. Place the broccolini and stock in the same frying pan, cover and cook for 3–4 minutes until the broccolini is almost tender. Add the kale or silverbeet and cook, tossing everything together, for 2 minutes until very wilted. Transfer to a bowl and top with the crispy shallot, garlic and walnut mixture.
4. Divide the chicken, butter beans, artichoke and garlicky greens between four bowls and serve.

## Note

Commercial chicken stock is usually very high in sodium. We recommend shopping for 'chicken broth', which is a similar product you can find in the refrigerator section of supermarkets and grocers. Broth is sold in small batches and contains less sodium. Check the label to ensure your stock contains less than 50 mg sodium per 100 g. Alternatively, if you'd like to make your own homemade stock, see the recipe on page 131.

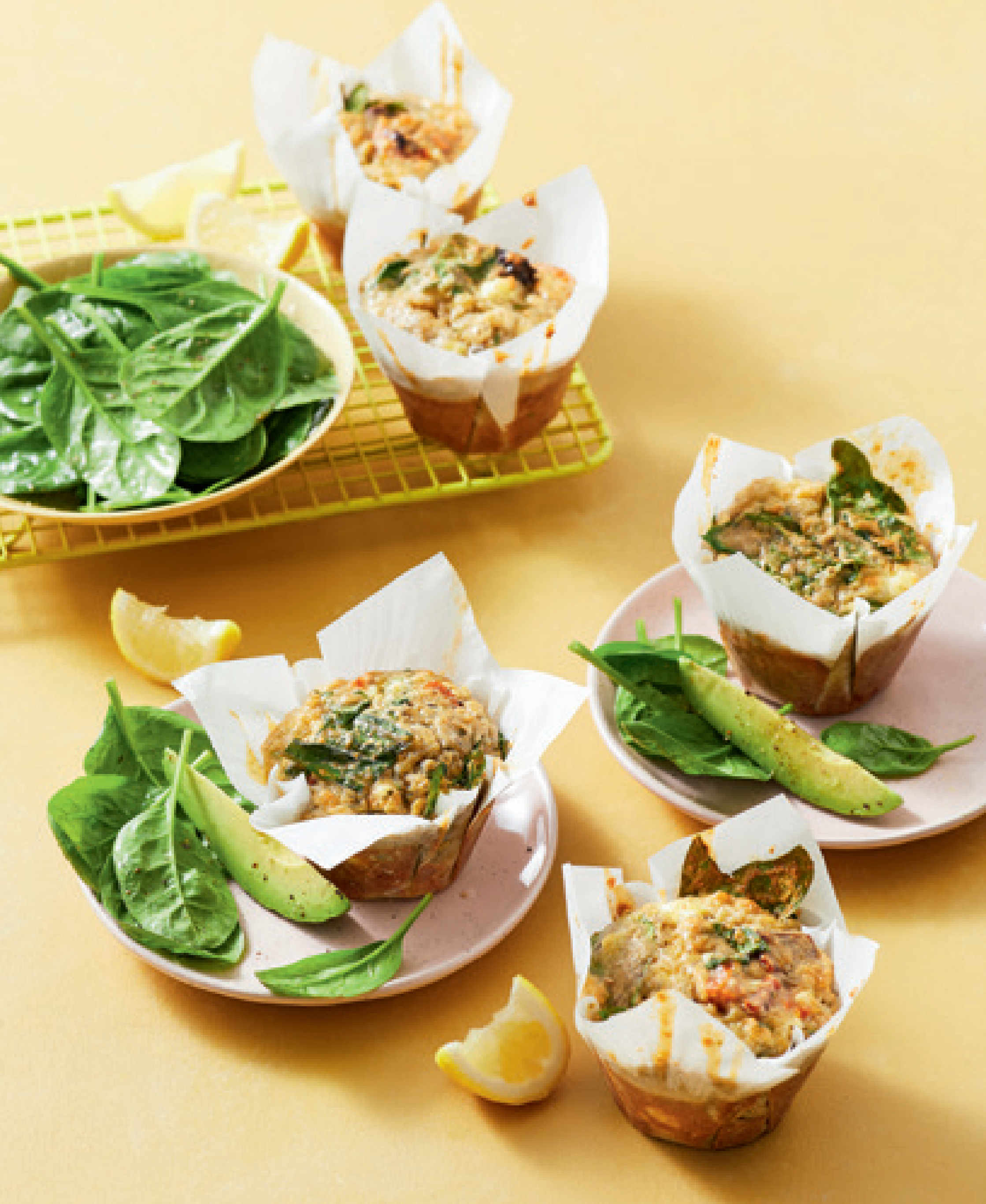

| UNITS PER SERVE: | |
|---|---|
| Breads, Cereals, Legumes, Starchy Veg | 0.5 |
| Dairy | 0.5 |
| Lean Meat, Fish, Poultry, Eggs, Tofu | 0.5 |
| Low–Moderate Carbohydrate Veg | 0.5 |
| Healthy Fats | 2 |

# Feta, Spinach and Sundried Tomato Breakfast Muffins

CARBS 12g PER SERVE

SERVES: 6 | PREPARATION: 10 MINUTES | COOKING: 20 MINUTES

6 × 55 g free-range eggs
¼ cup (60 ml) full-fat milk
40 g reduced-fat firm feta
½ cup (70 g) sundried tomatoes, roughly chopped
1 tablespoon oil-free pesto
1 cup baby spinach leaves, roughly chopped
½ cup (60 g) wholemeal self-raising flour

### TO SERVE

½ teaspoon Nuttelex or canola margarine
½ cup baby spinach leaves
1 × 20 g slice avocado
Lemon wedges

1. Preheat the oven to 200°C (180°C fan-forced) and line 6 holes of a muffin tin with patty pans.
2. In a large bowl, whisk the eggs and milk. Add the remaining ingredients and ¼ teaspoon freshly ground black pepper and mix until well combined.
3. Pour the batter into 6 holes of the muffin tin. Bake in the oven for 20 minutes until the egg has cooked through and the muffins are set.
4. Remove from the oven and allow to cool in the tin. Divide the muffins into ziplock bags or place in an airtight container to store.
5. To serve, warm one muffin, split it open and spread with Nuttelex or margarine. Serve with the baby spinach, avocado and a squeeze of lemon juice.

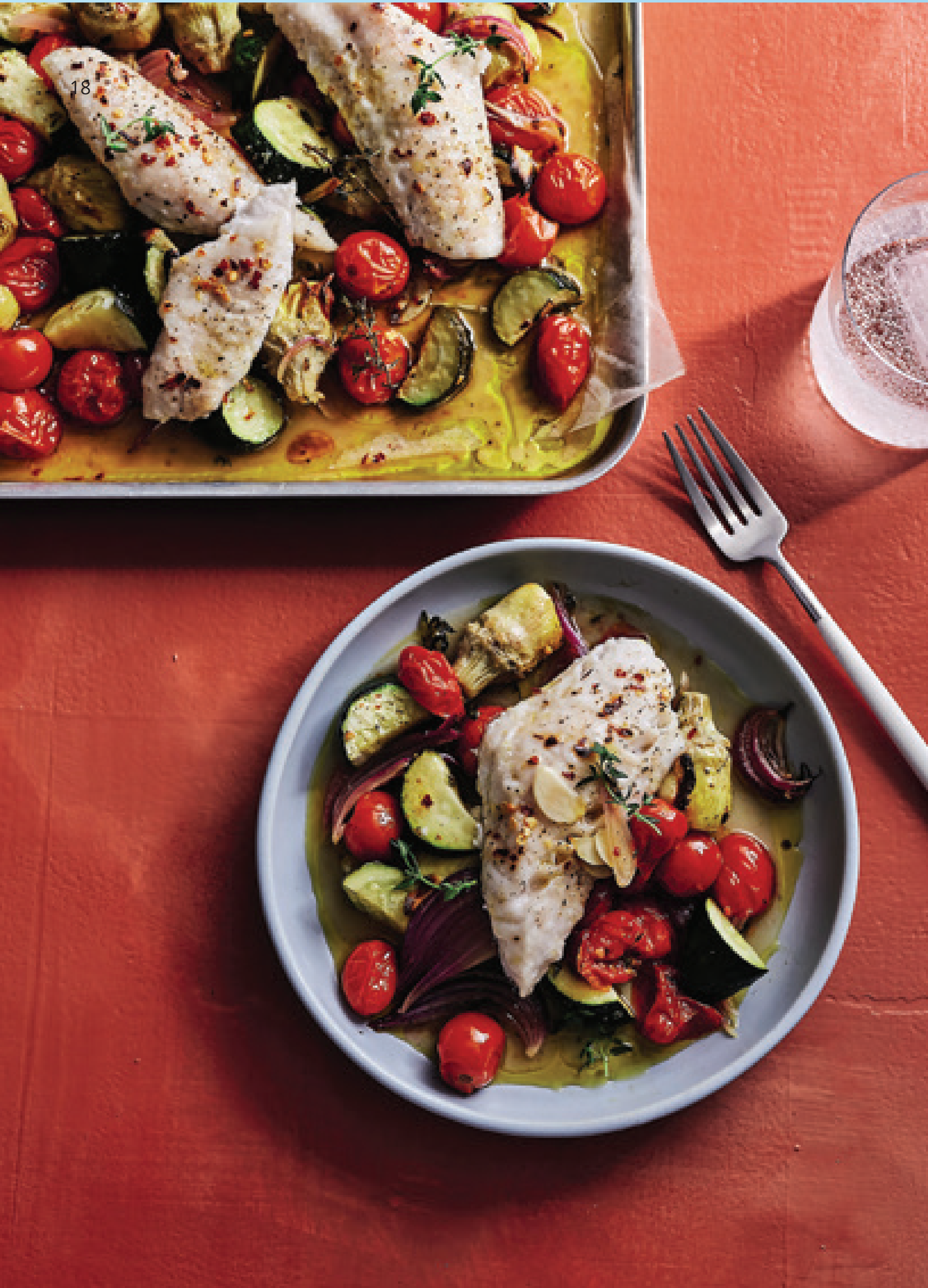

| UNITS PER SERVE: | |
|---|---|
| Breads, Cereals, Legumes, Starchy Veg | 0 |
| Dairy | 0 |
| Lean Meat, Fish, Poultry, Eggs, Tofu | 1.5 |
| Low–Moderate Carbohydrate Veg | 3 |
| Healthy Fats | 3 |

# Fish with Artichoke, Cherry Tomatoes and Thyme

CARBS 16 g PER SERVE

SERVES: 4 | PREPARATION: 10 MINUTES | COOKING: 30 MINUTES

3 tablespoons extra virgin olive oil
Pinch dried chilli flakes (optional)
5 cloves garlic, thinly sliced
1 small red onion, thinly sliced
5 sprigs thyme, leaves picked, plus extra thyme leaves to serve
450 g drained artichoke hearts
750 g cherry tomatoes
2 zucchini, thinly sliced
600 g skinless, boneless firm white fish, such as snapper, barramundi or flathead (see note on page 174 regarding sodium)
2 tablespoons white wine vinegar

1. Preheat the oven to 200°C (180°C fan-forced).
2. Divide the olive oil between two medium-sized baking dishes (or one very large dish) and place in the oven for 10 minutes to heat the oil.
3. Carefully remove the baking dishes from the oven and divide the chilli flakes, garlic, onion and thyme leaves between the two dishes, tossing the ingredients together, allowing them to sizzle in the hot oil. Divide the artichoke, cherry tomatoes and zucchini between the two dishes, tossing to coat them in the oil.
4. Return the dishes to the oven and roast for 20 minutes. Carefully remove the dishes from the oven again, place the fish fillets on top and drizzle with the vinegar. Roast for a final 10 minutes until the fish is cooked through and the vegetables are very softened.
5. Divide the roasted vegetables and fish between four plates. Spoon over any sauce pooled at the bottom of the casserole dishes, sprinkle with freshly ground black pepper and the extra thyme leaves and serve.

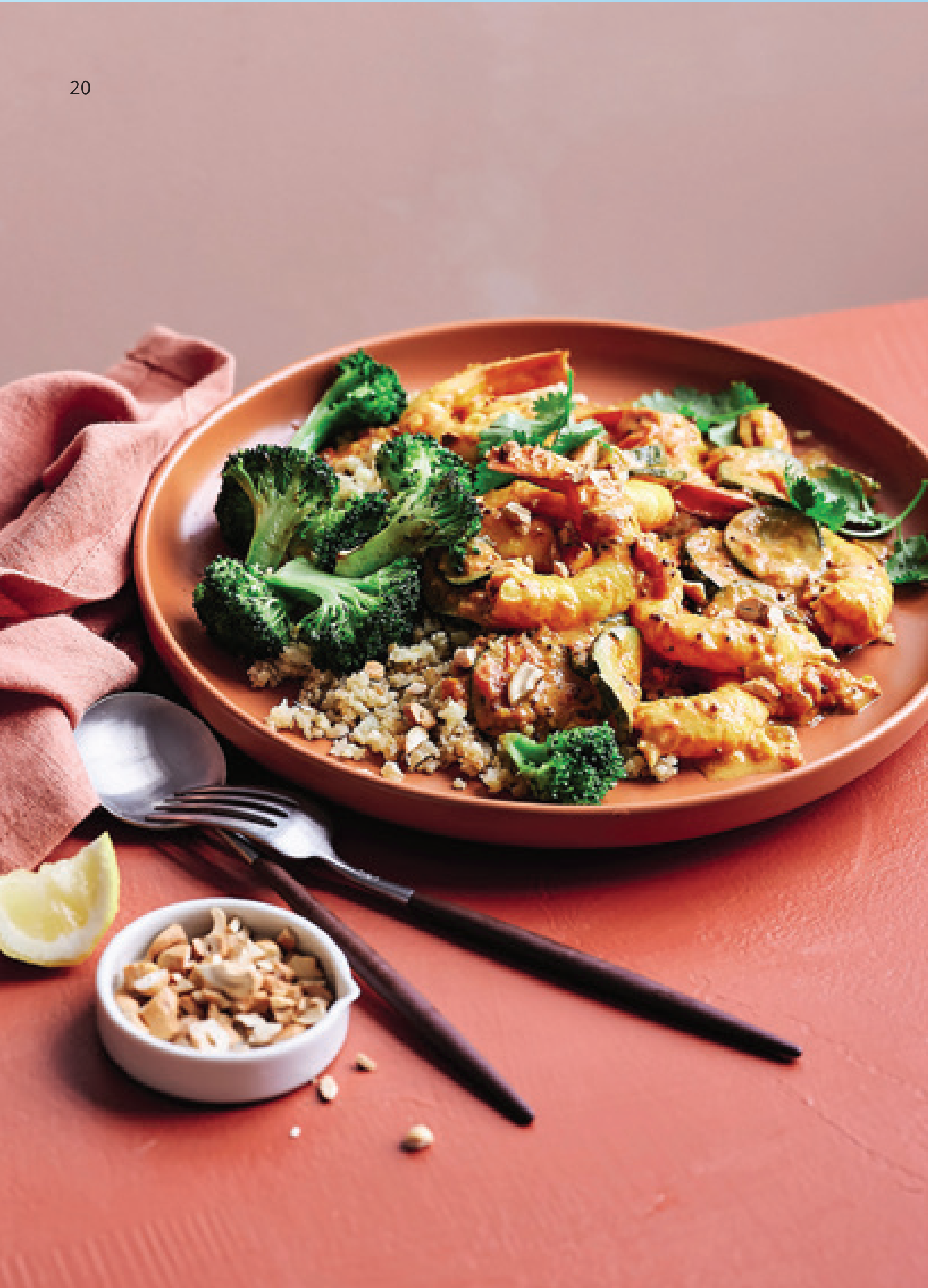

| UNITS PER SERVE: | |
|---|---|
| Breads, Cereals, Legumes, Starchy Veg | 0 |
| Dairy | 1 |
| Lean Meat, Fish, Poultry, Eggs, Tofu | 1.5 |
| Low–Moderate Carbohydrate Veg | 3.5 |
| Healthy Fats | 2 |

# Prawn Curry with Cauliflower Rice

CARBS 18 g PER SERVE

SERVES: 4 | PREPARATION: 10 MINUTES | COOKING: 25 MINUTES

2 heads broccoli, stems and florets chopped
1 tablespoon extra virgin olive oil
1 onion, finely diced
1 zucchini, thinly sliced
1 long red chilli, thinly sliced
3 cloves garlic, sliced
2 cm piece of ginger, finely grated
2 teaspoons curry powder
3 teaspoons brown mustard seeds
1 teaspoon ground turmeric
400 g tinned diced tomatoes
600 g cooked prawns, peeled and deveined, tails intact (see notes, and also note on page 174 regarding sodium)
1 cup (260 g) natural yoghurt
Juice of 1 lemon

### TO SERVE

450 g cauliflower rice, heated according to the packet instructions (see notes)
2 tablespoons chopped roasted unsalted cashews
½ cup coriander leaves

1. Bring a large saucepan of water to the boil and cook the broccoli for 5 minutes until tender. Drain and set aside.
2. Heat the olive oil in a large, deep frying pan over medium heat. Add the onion and zucchini and cook, stirring occasionally, for 6–7 minutes until very softened. Add the chilli, garlic, ginger, curry powder, mustard seeds, turmeric and ¼ teaspoon freshly ground black pepper and cook, stirring, for a further 1–2 minutes until fragrant.
3. Add the diced tomatoes, 2 tablespoons water and the prawns, tossing everything together. (There won't be a lot of sauce here, so place a lid on the frying pan to help the prawns steam to cook through.) Cook, covered, for 2–3 minutes until the prawns are cooked through, then stir the yoghurt and lemon juice through.
4. Divide the cauliflower rice four between bowls, spoon the prawn curry over and add the cooked broccoli. Garnish with the cashews and coriander leaves and serve.

## Notes

You can use frozen raw prawns if you prefer. But don't add the water to the frying pan as the frozen prawns will release quite a bit of moisture. You may need to simmer the sauce for an extra 2 minutes to reduce any excess liquid in your curry before serving.

You can find cauliflower rice in the fresh produce section at your local supermarket. If unavailable, use the frozen variety instead.

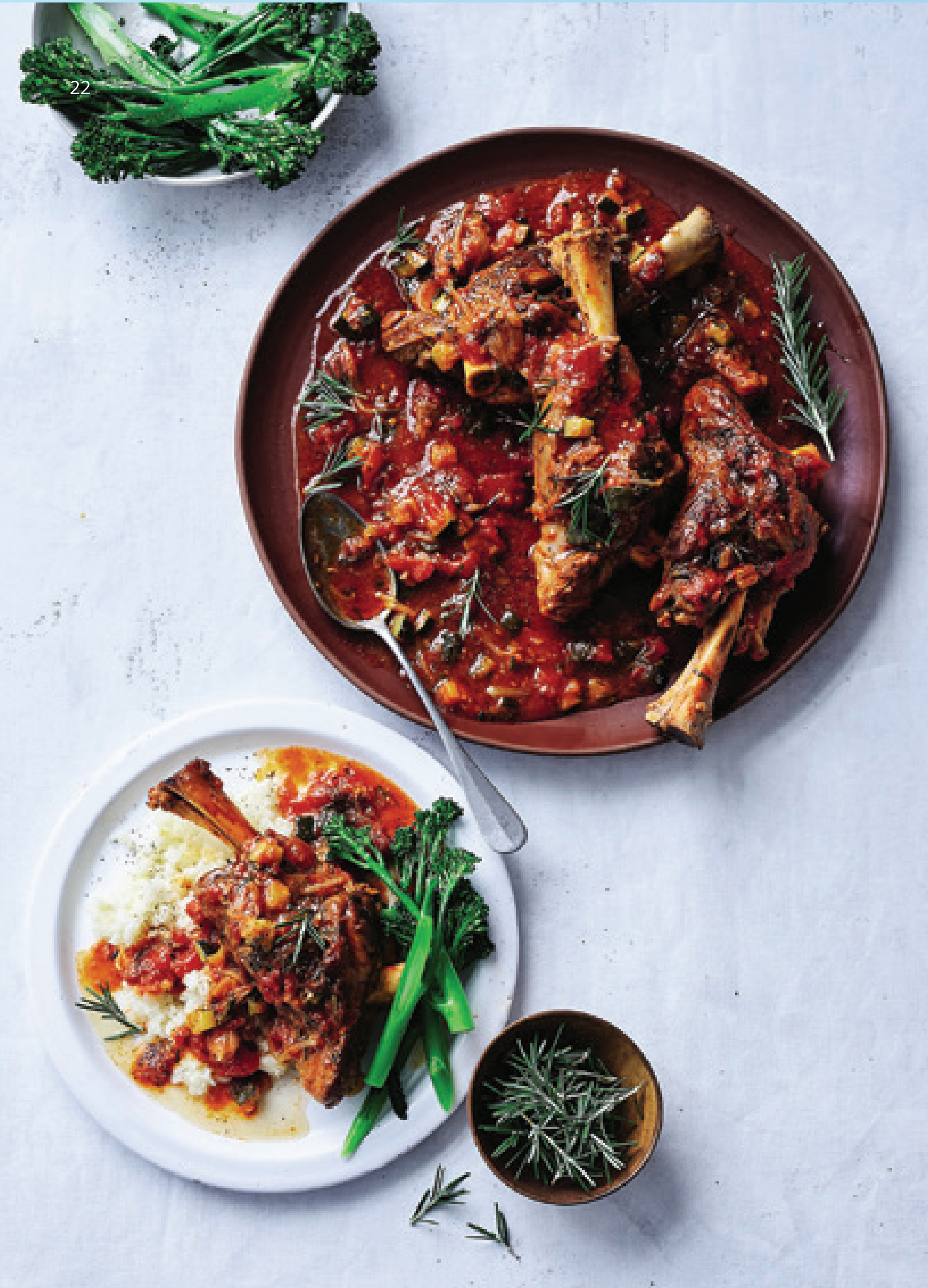

| UNITS PER SERVE: | |
|---|---|
| Breads, Cereals, Legumes, Starchy Veg | 0 |
| Dairy | 0.5 |
| Lean Meat, Fish, Poultry, Eggs, Tofu | 1.5 |
| Low–Moderate Carbohydrate Veg | 3 |
| Healthy Fats | 2 |

# Tomato-braised Lamb Shanks with Crushed Cauliflower

CARBS 14g PER SERVE

**SERVES: 4 | PREPARATION: 15 MINUTES | COOKING: 2.5–3.5 HOURS**

1½ tablespoons extra virgin olive oil
4 large fully trimmed lamb shanks (1.8 kg) (150 g meat per serve)
1 onion, thinly sliced
1 zucchini, finely diced
4 cloves garlic, crushed
4 sprigs thyme, leaves picked
2 sprigs rosemary, leaves picked and chopped
2 tablespoons tomato paste (no added sugar or salt)
2 tablespoons red wine vinegar
400 g tinned whole peeled tomatoes (no added sugar or salt)
2 cups (500 ml) salt-reduced chicken stock (see note)
2 bunches broccolini, trimmed

## CRUSHED CAULIFLOWER

½ head cauliflower, chopped into 3 cm chunks
30 g parmesan, finely grated
2 teaspoons extra virgin olive oil

1. Preheat the oven to 200°C (180°C fan-forced).
2. Heat the olive oil in a large heavy-based casserole dish over medium–high heat. Add the lamb shanks in batches, and cook, turning, for 4–5 minutes until browned. Transfer to a plate and set aside.
3. To the same casserole dish, add the onion, zucchini, garlic, thyme and rosemary and cook for 3–4 minutes until softened. Add the tomato paste and stir it through until the vegetables are well coated. Add the vinegar and use a spatula to scrape up any bits from the bottom of the pan. Cook for 1 minute until the vinegar evaporates.
4. Break up the whole peeled tomatoes using your hands (or a knife) and add them to the casserole dish, along with the stock and 1 cup (250 ml) water. Return the lamb shanks to the dish, bring to the boil, then turn off the heat and place the lid on. Bake in the oven for 2–3 hours, or until the lamb is very tender.
5. When the lamb has 20 minutes cooking time left, make the crushed cauliflower. Place the cauliflower in a large saucepan over high heat and cover with water. Bring to the boil and cook for 10 minutes until the cauliflower is mostly tender. Add the broccolini and continue boiling for another 3 minutes, until the cauliflower is tender and the broccolini is bright green.
6. Pick out the broccolini with tongs and set aside, then drain the cauliflower and return it to the saucepan with the parmesan, olive oil and ¼ teaspoon freshly ground black pepper. Use a fork or potato masher to lightly crush the cauliflower.
7. Divide the lamb shanks, broccolini and crushed cauliflower between four bowls and spoon over the tomato sauce to serve.

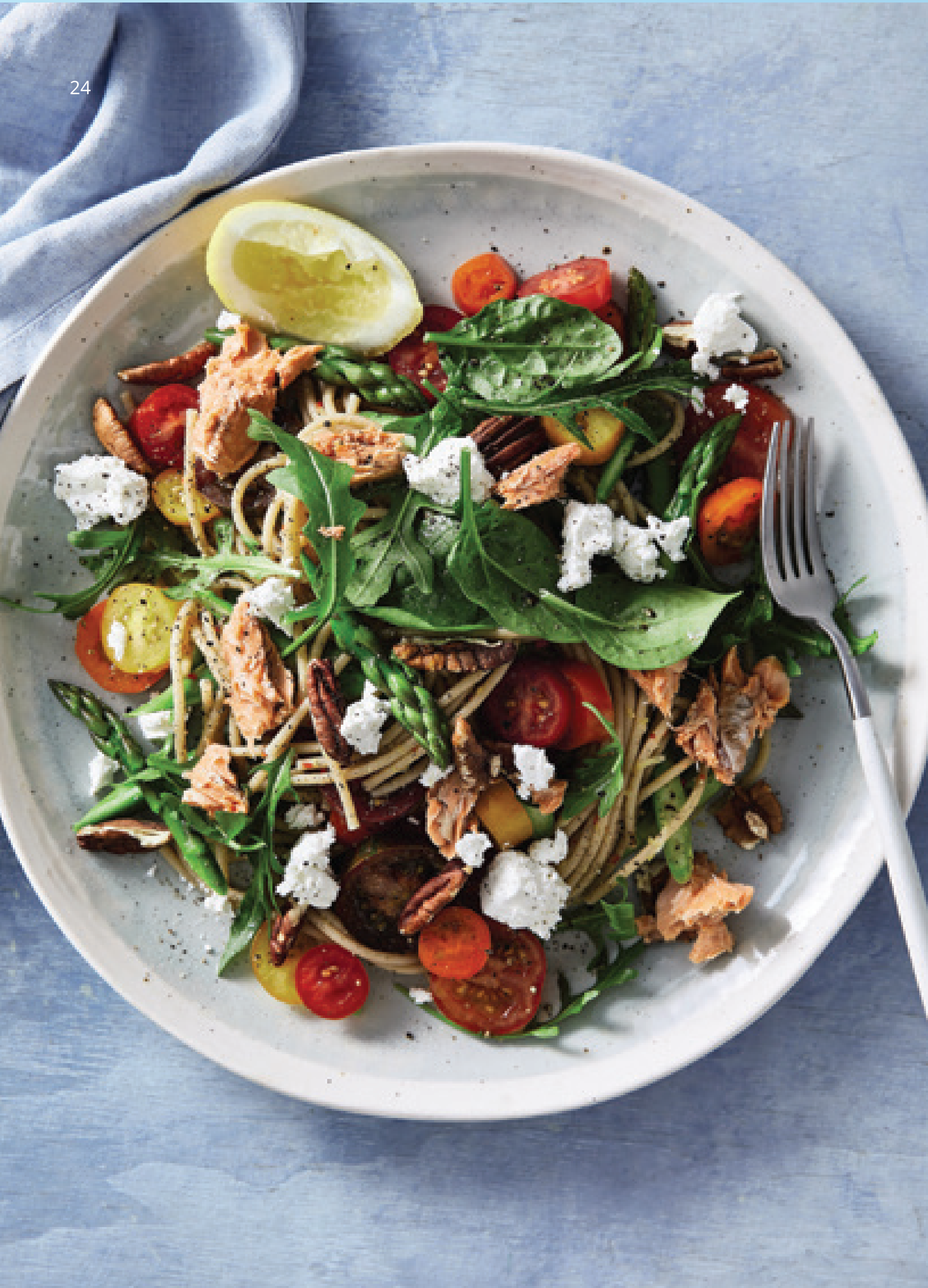

# Asparagus and salmon spaghetti

GRAMS CARB 25 PER SERVE

Serves 4 Preparation: 20 minutes
Cooking: 5 minutes Difficulty: Low

120 g dried wholemeal spaghetti, broken into thirds
4 bunches asparagus, trimmed and cut diagonally into 4 cm lengths
400 g drained tinned red salmon, flaked (see note)
120 g baby spinach and rocket leaf mix
200 g mixed baby tomatoes, sliced into rounds
1 long red chilli, seeded and finely chopped
Finely grated zest and juice of 1 lemon
80 g Danish feta, crumbled
160 g toasted pecans, chopped

Cook the pasta in a saucepan of boiling water over high heat for 4 minutes. Add the asparagus and cook for a further 1 minute or until the pasta is al dente and the asparagus is tender. Drain well and transfer to a large bowl.

Immediately add the salmon, leaf mix, tomato, chilli, lemon zest and juice to the bowl and toss to combine.

Divide the pasta mixture among bowls, sprinkle with the feta and pecans and serve warm.

**Note:** You will require 1 x 415 g tin of red salmon for this recipe. Store the leftover salmon in an airtight container in the fridge for up to 3 days.

## UNITS PER SERVE

| BREADS, CEREALS, LEGUMES, STARCHY VEGETABLES | DAIRY | LEAN MEAT, FISH, POULTRY, EGGS, TOFU | LOW-CARB VEGETABLES | MODERATE-CARB VEGETABLES | HEALTHY FATS |
|---|---|---|---|---|---|
| 1 | 1 | 1 | 2 | 0 | 2 |

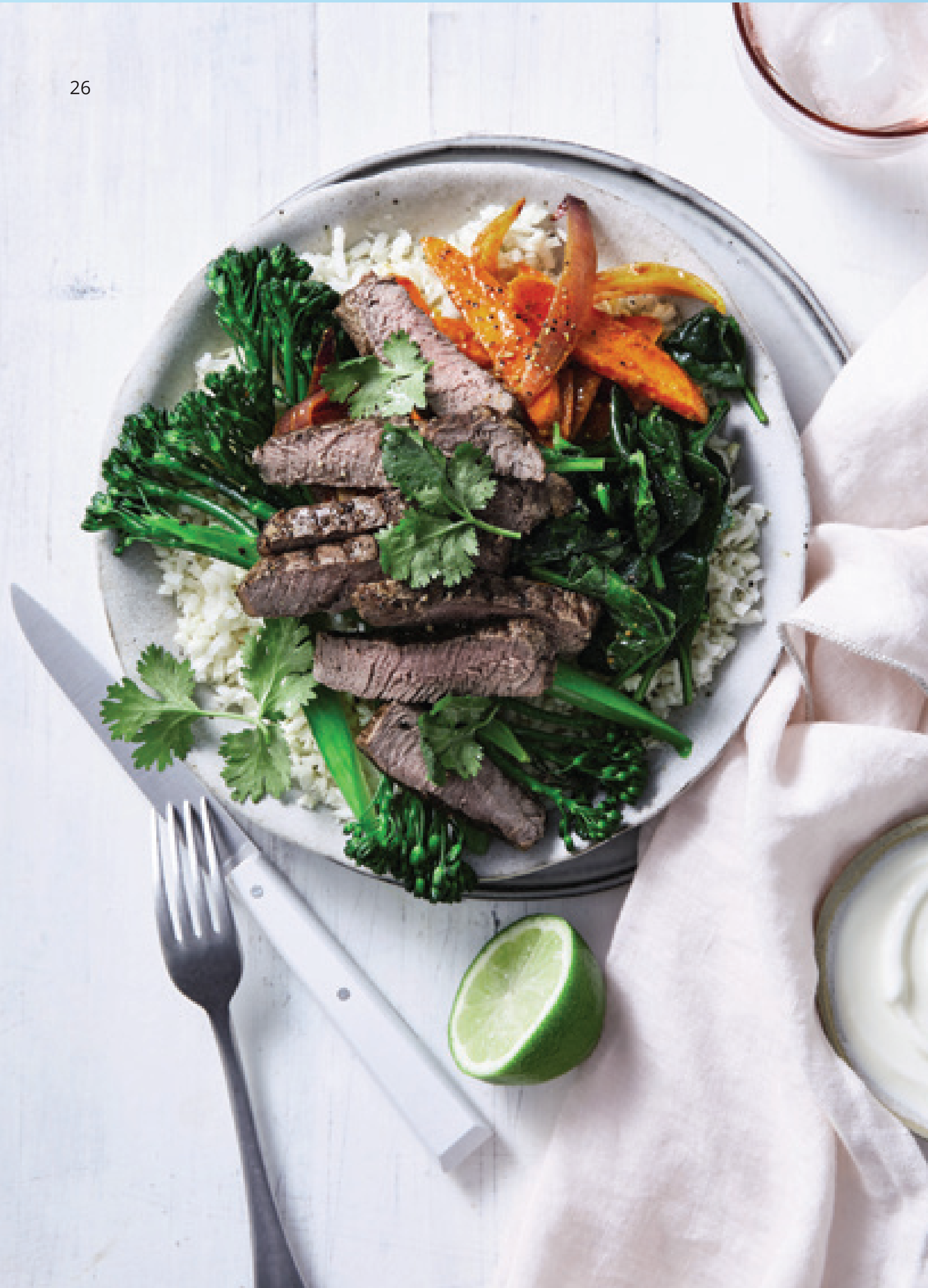

# Beef fillet steaks with korma vegetables

GRAMS CARB 13 PER SERVE

Serves 4 · Preparation: 25 minutes, plus 3 minutes resting time
Cooking: 10 minutes · Difficulty: Low

- 2 tablespoons korma curry paste
- 1 red onion, cut into thin wedges
- 1 carrot, halved lengthways, thinly sliced diagonally
- 3 bunches broccolini, trimmed
- ½ cup (125 ml) salt-reduced beef stock
- 150 g baby spinach leaves
- 100 g Greek-style yoghurt
- 4 x 150 g lean beef fillet steaks
- 500 g fresh cauliflower rice (see note)
- ½ cup coriander leaves

Heat the korma paste in a large deep non-stick frying pan over high heat. Add the onion and carrot and cook, stirring occasionally, for 3 minutes or until starting to soften. Reduce the heat to medium. Add the broccolini and stock and simmer, stirring occasionally, for 5 minutes or until almost tender. Add the spinach and stir until just wilted. Remove the pan from the heat, stir through the yoghurt and season with freshly ground black pepper.

Meanwhile, heat a chargrill pan over high heat. Season the steaks with freshly ground black pepper, then add to the pan and cook for 3–4 minutes each side for medium or until cooked to your liking. Transfer to serving plates, cover loosely with foil and leave to rest for 3 minutes before slicing.

Heat the cauliflower rice according to the packet instructions.

Divide the cauliflower rice among serving plates and top with the korma vegetables and steak. Sprinkle with the coriander and serve.

**Note:** You can find fresh cauliflower rice in the fresh produce section at your local supermarket. If unavailable, use the frozen variety instead.

**UNITS PER SERVE**

| BREADS, CEREALS, LEGUMES, STARCHY VEGETABLES | DAIRY | LEAN MEAT, FISH, POULTRY, EGGS, TOFU | LOW-CARB VEGETABLES | MODERATE-CARB VEGETABLES | HEALTHY FATS |
|---|---|---|---|---|---|
| 0 | >0.5 | 1.5 | 2 | 1 | 2 |

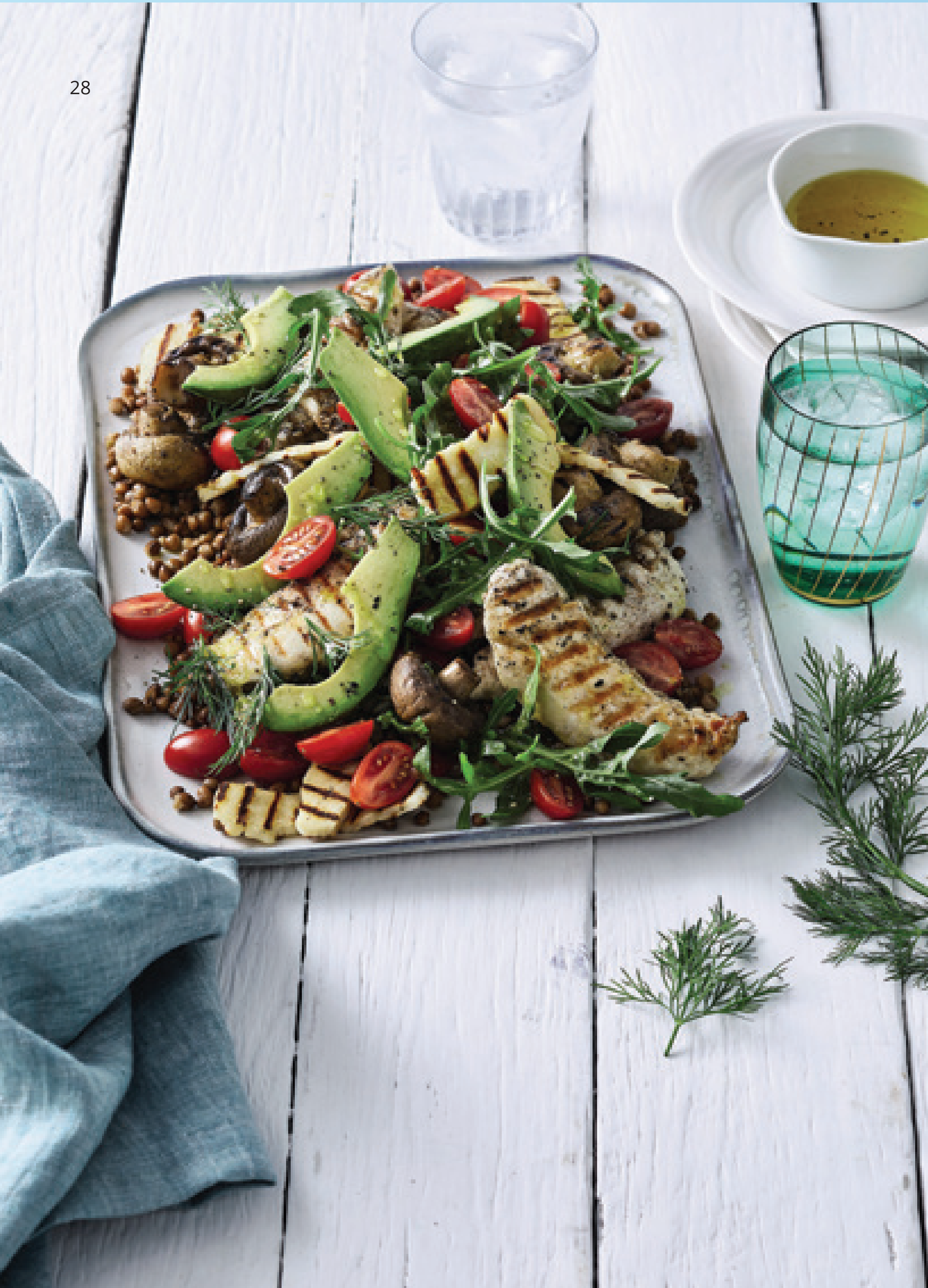

# Chargrilled chicken *and* mushrooms *with* avocado lentils

GRAMS CARB **20** PER SERVE

Serves 4 Preparation: 20 minutes
Cooking: 10 minutes Difficulty: Low

- 600 g cup mushrooms, wiped clean
- 400 g lean chicken tenderloins
- 80 g haloumi, sliced
- 360 g drained, rinsed tinned lentils (see note)
- 250 g cherry tomatoes, halved
- 100 g baby rocket leaves
- ¼ cup dill fronds
- finely grated zest and juice of 1 large lemon
- 1 tablespoon extra virgin olive oil
- 160 g avocado, sliced

Preheat a large chargrill pan over high heat. Season the mushrooms and chicken with freshly ground black pepper, add to the pan and cook, turning occasionally, for 6–8 minutes or until tender and golden. Chargrill the haloumi for 30 seconds each side until warmed through and light golden.

Meanwhile, combine all the remaining ingredients, except the avocado, in a large bowl. Season with freshly ground black pepper.

Divide the lentil mixture among plates and top with the avocado, mushrooms, chicken and haloumi.

**Note:** You will require 2 x 400 g tins of lentils for this recipe. Store leftover lentils in an airtight container in the fridge for up to 3 days.

**UNITS PER SERVE**

| BREADS, CEREALS, LEGUMES, STARCHY VEGETABLES | DAIRY | LEAN MEAT, FISH, POULTRY, EGGS, TOFU | LOW-CARB VEGETABLES | MODERATE-CARB VEGETABLES | HEALTHY FATS |
|---|---|---|---|---|---|
| 1 | 1 | 1 | 2 | 0 | 3 |

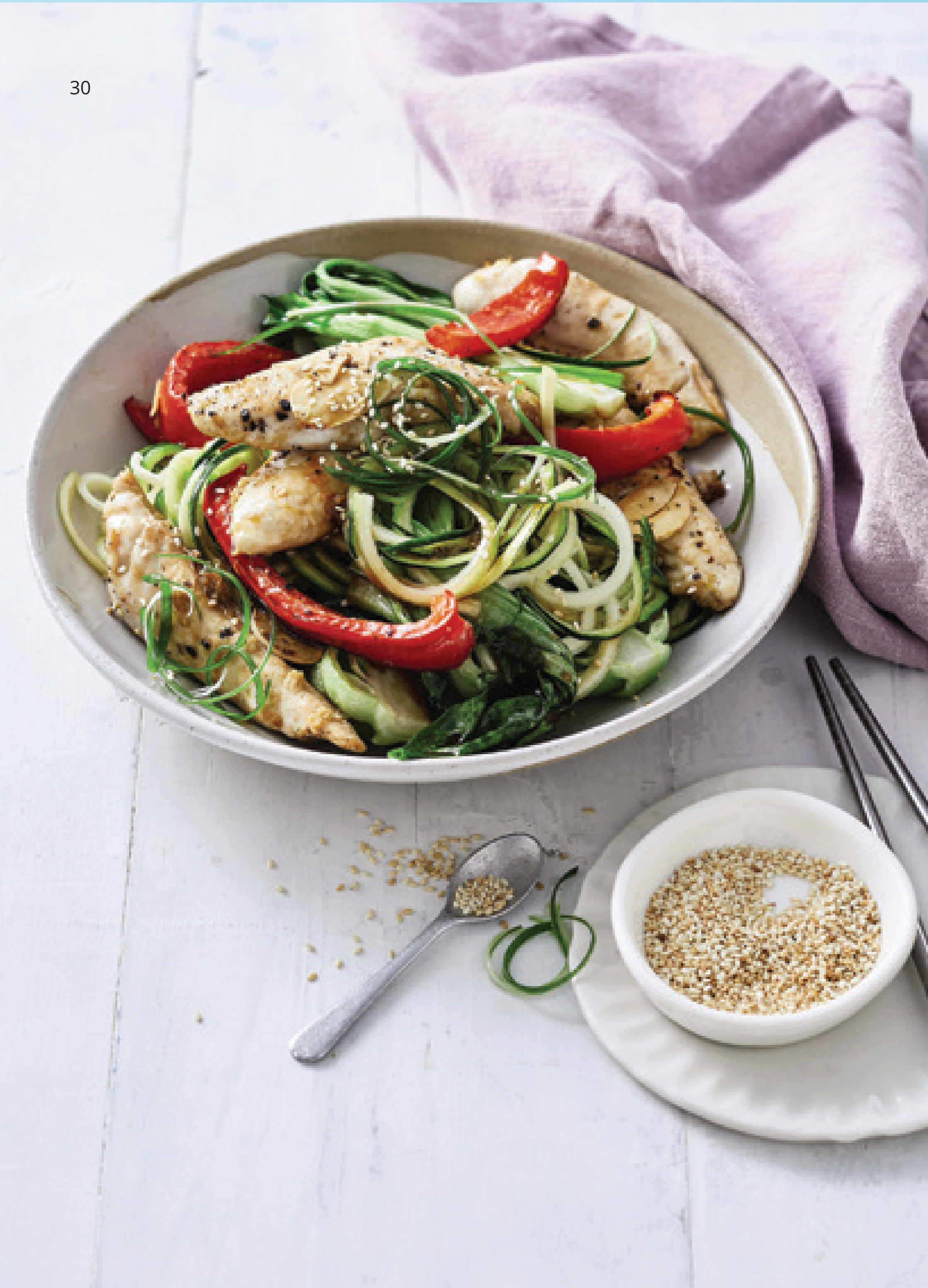

# Ginger chicken *and* bok choy bake

GRAMS CARB 7 PER SERVE

Serves 4 Preparation: 25 minutes
Cooking: 25 minutes Difficulty: Low

**2 bunches (6 pieces) baby bok choy, halved lengthways**
**1 red capsicum, seeded and sliced**
**2 cloves garlic, sliced**
**7 cm piece ginger, finely grated**
**¼ cup (60 ml) salt-reduced soy sauce**
**½ cup (125 ml) salt-reduced chicken stock**
**600 g lean chicken tenderloins**
**500 g fresh zucchini noodles (see note)**
**2 spring onions, thinly sliced**
**2 tablespoons toasted sesame seeds**

Preheat the oven to 200°C (180°C fan-forced).

Place the bok choy, capsicum, garlic, ginger, soy sauce, stock and chicken in a roasting tin and toss well to combine and coat. Bake, tossing occasionally, for 20–25 minutes or until cooked and golden.

Meanwhile, heat the zucchini noodles according to the packet instructions.

Divide the zucchini noodles among large bowls and top with the chicken mixture. Sprinkle with the spring onion and sesame seeds and serve.

**Note:** You'll find fresh zucchini noodles in the fresh produce section at the supermarket. If unavailable, you can simply cut 4 zucchini into thin matchsticks and lightly steam.

UNITS PER SERVE

| BREADS, CEREALS, LEGUMES, STARCHY VEGETABLES | DAIRY | LEAN MEAT, FISH, POULTRY, EGGS, TOFU | LOW-CARB VEGETABLES | MODERATE-CARB VEGETABLES | HEALTHY FATS |
|---|---|---|---|---|---|
| 0 | 0 | 1.5 | 2 | 2 | 2 |

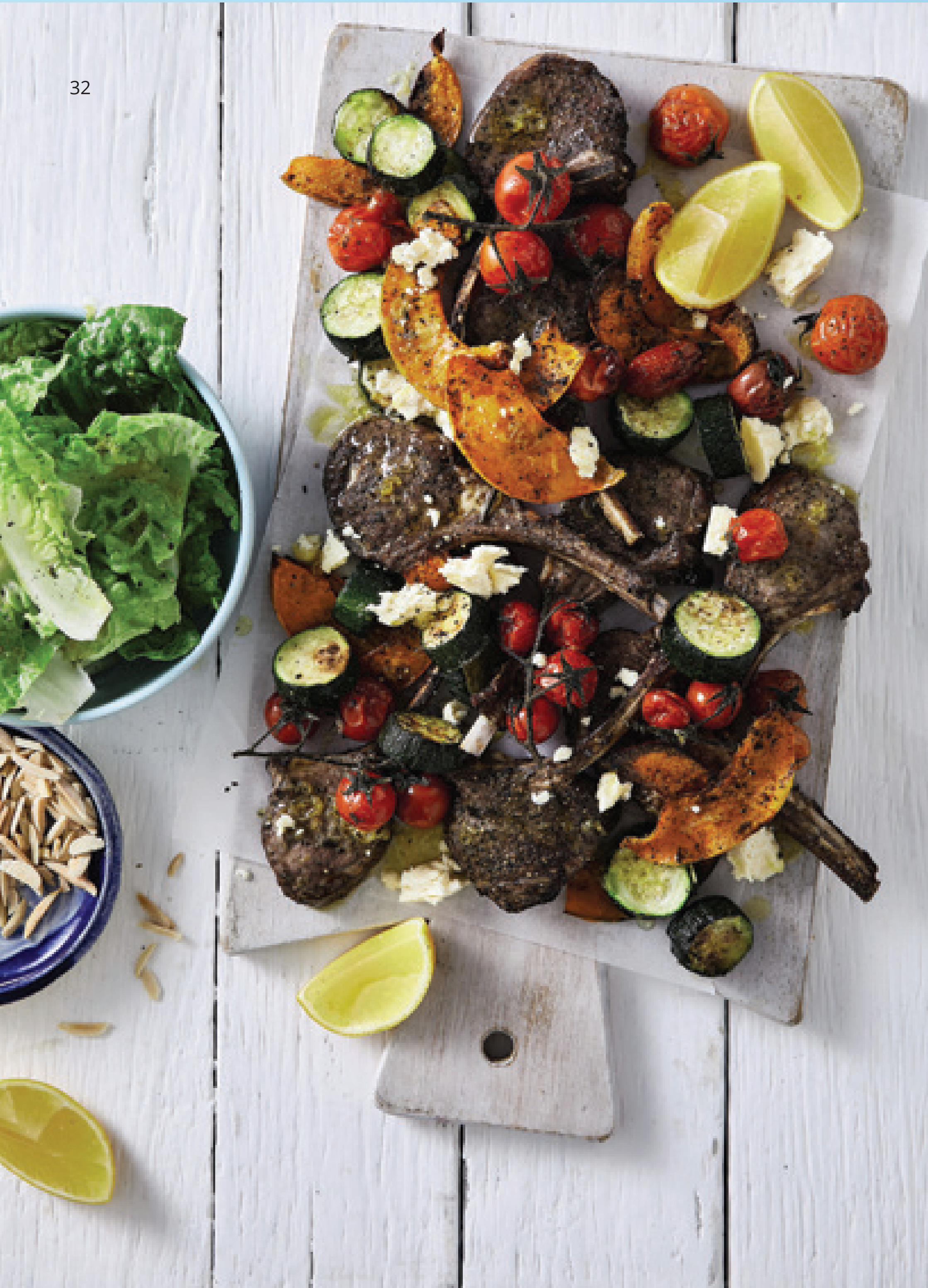

# Greek roasted lamb cutlets *and* veggies

GRAMS CARB 8 PER SERVE

Serves 4 · Preparation: 25 minutes, plus 5 minutes resting time
Cooking: 25 minutes · Difficulty: Low

**150 g peeled, seeded pumpkin, thinly sliced**
**4 zucchini, thickly sliced into rounds**
**500 g cherry tomatoes**
**600 g lean French-trimmed lamb cutlets**
**2 tablespoons lamb dried herbs (see note)**
**2 tablespoons extra virgin olive oil**
**Finely grated zest and juice of 1 large lemon, plus extra lemon wedges to serve**
**1 cos lettuce, roughly chopped**
**80 g Greek feta, crumbled**
**80 g toasted slivered almonds**

Preheat the oven to 200°C (180°C fan-forced) and line a large baking tray with baking paper.

Place the pumpkin, zucchini, tomatoes, lamb cutlets, dried herbs and 1 tablespoon oil on the prepared tray, tossing to combine and coat. Spread out in a single layer and roast for 20–25 minutes or until cooked and golden. Remove from the oven and rest for 5 minutes.

Meanwhile, whisk the lemon zest and juice with the remaining oil in a large bowl. Add the lettuce, feta and almonds and toss to combine.

Divide the lamb cutlets and vegetable mixture among plates and serve with the salad and lemon wedges.

Note: The lamb dried herbs seasoning mix is a combination of dried rosemary, garlic, thyme, oregano, mint, marjoram and basil and is readily available in the spice aisle at the supermarket. If unavailable, you can either make your own blend of all the herbs listed above or simply use 2 teaspoons each of dried rosemary, dried thyme, dried oregano and dried mint.

## UNITS PER SERVE

| BREADS, CEREALS, LEGUMES, STARCHY VEGETABLES | DAIRY | LEAN MEAT, FISH, POULTRY, EGGS, TOFU | LOW-CARB VEGETABLES | MODERATE-CARB VEGETABLES | HEALTHY FATS |
|---|---|---|---|---|---|
| 0 | 1 | 1.5 | 2 | 0.5 | 4 |

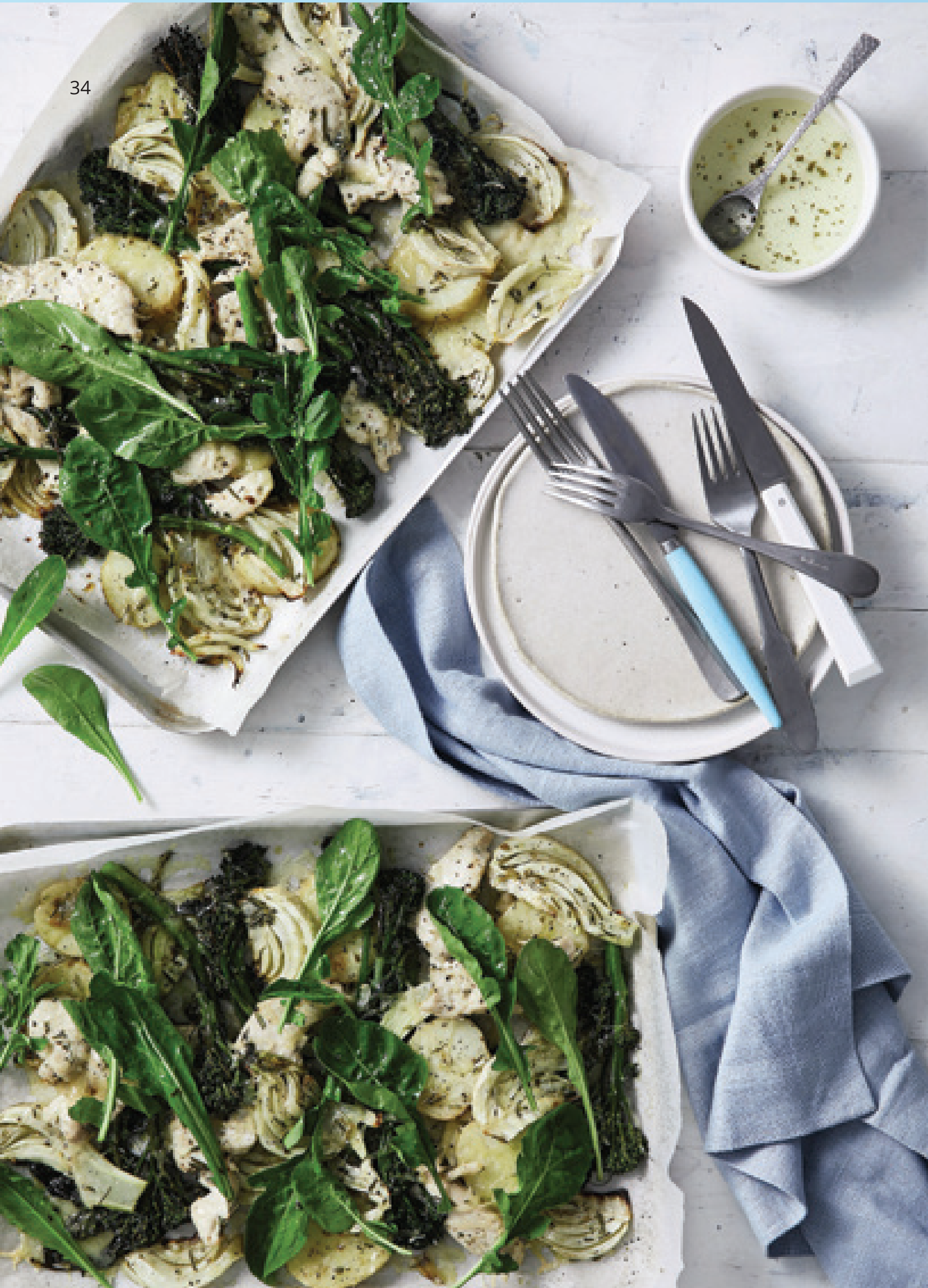

# Rosemary potato *and* chicken bake

GRAMS CARB 15 PER SERVE

Serves 4 · Preparation: 20 minutes
Cooking: 40 minutes · Difficulty: Low

- 400 g washed potatoes, very thinly sliced into rounds
- 400 g lean chicken breast stir-fry strips
- ¼ cup rosemary leaves
- 2 tablespoons olive oil
- 2 cloves garlic, crushed
- 2 bulbs baby fennel, cored and thinly sliced lengthways
- 2 bunches broccolini, trimmed
- 80 g Gruyere cheese, finely grated
- 2 bunches rocket, trimmed
- ¼ cup (60 ml) oil-free Italian dressing

Preheat the oven to 220°C (200°C fan-forced).

Place the potato, chicken, rosemary, oil, garlic, fennel and broccolini in two roasting tins and toss to combine and coat well. Season with freshly ground black pepper. Bake, turning occasionally, for 25–30 minutes or until cooked and golden. Sprinkle with the cheese, then bake for a further 10 minutes or until the cheese has melted.

Remove the tins from the oven, add the rocket and dressing and toss to combine. Serve straight from the tins at the table.

## UNITS PER SERVE

| BREADS, CEREALS, LEGUMES, STARCHY VEGETABLES | DAIRY | LEAN MEAT, FISH, POULTRY, EGGS, TOFU | LOW-CARB VEGETABLES | MODERATE-CARB VEGETABLES | HEALTHY FATS |
|---|---|---|---|---|---|
| 1 | 1 | 1 | 2 | 1 | 2 |

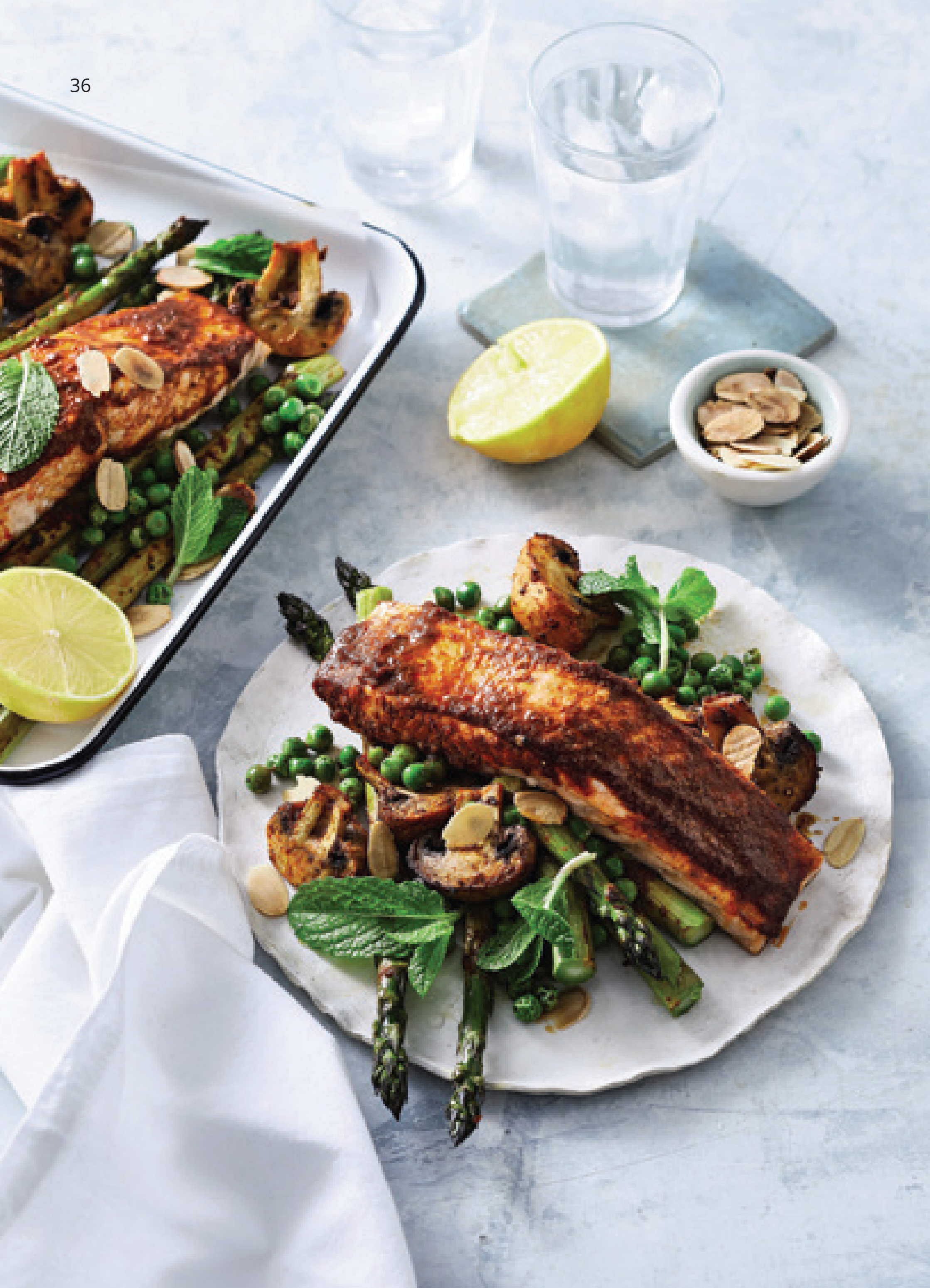

# Tandoori salmon tray bake

GRAMS CARB 8 PER SERVE

Serves 4 Preparation: 25 minutes
Cooking: 20 minutes Difficulty: Low

- 2 tablespoons tandoori paste
- Finely grated zest and juice of 1 large lemon, plus extra wedges to serve
- 4 x 150 g skinless, boneless salmon fillets
- 3 bunches asparagus, trimmed
- 600 g cup mushrooms, halved
- Olive oil spray, for cooking
- 1 cup frozen baby peas
- 40 g flaked almonds
- ½ cup small mint leaves

Preheat the oven to 200°C (180°C fan-forced) and line a large baking tray with baking paper.

Whisk together the tandoori paste, lemon zest and juice in a large bowl. Add the salmon and toss well to coat. Transfer the salmon to the prepared tray. Place the asparagus and mushrooms in the bowl with the remaining tandoori mixture and toss well to coat. Transfer to the tray, in and around the salmon pieces, then lightly spray everything with the olive oil.

Bake for 10 minutes, then add the peas and almonds and bake for a further 8–10 minutes or until cooked and golden.

Remove the tray from the oven, sprinkle with the mint leaves and serve with the lemon wedges.

UNITS PER SERVE

| BREADS, CEREALS, LEGUMES, STARCHY VEGETABLES | DAIRY | LEAN MEAT, FISH, POULTRY, EGGS, TOFU | LOW-CARB VEGETABLES | MODERATE-CARB VEGETABLES | HEALTHY FATS |
|---|---|---|---|---|---|
| 0 | 0 | 1.5 | 2 | 1 | 3 |

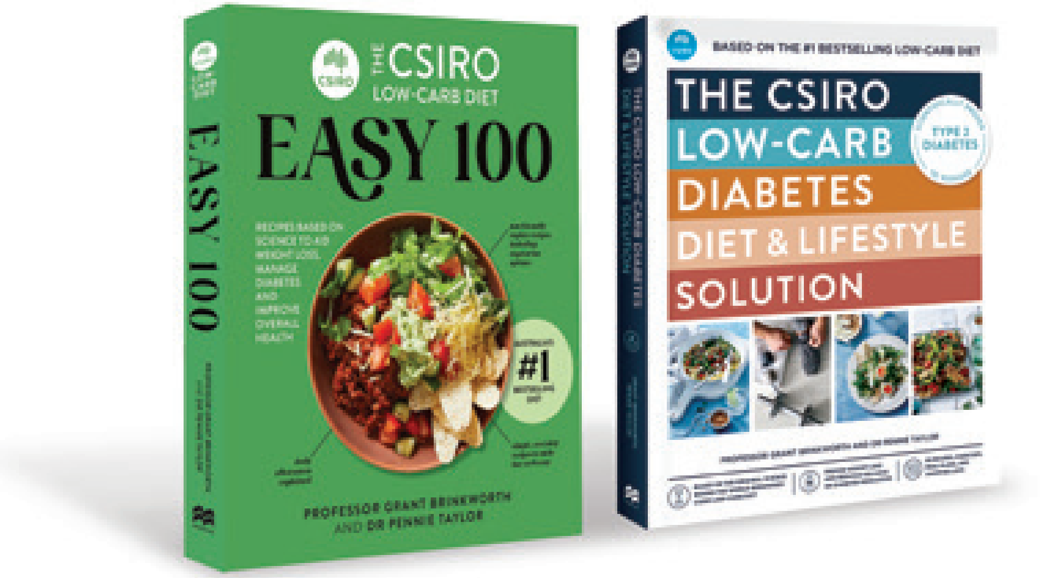


Scan this QR code for information
on purchasing these books.

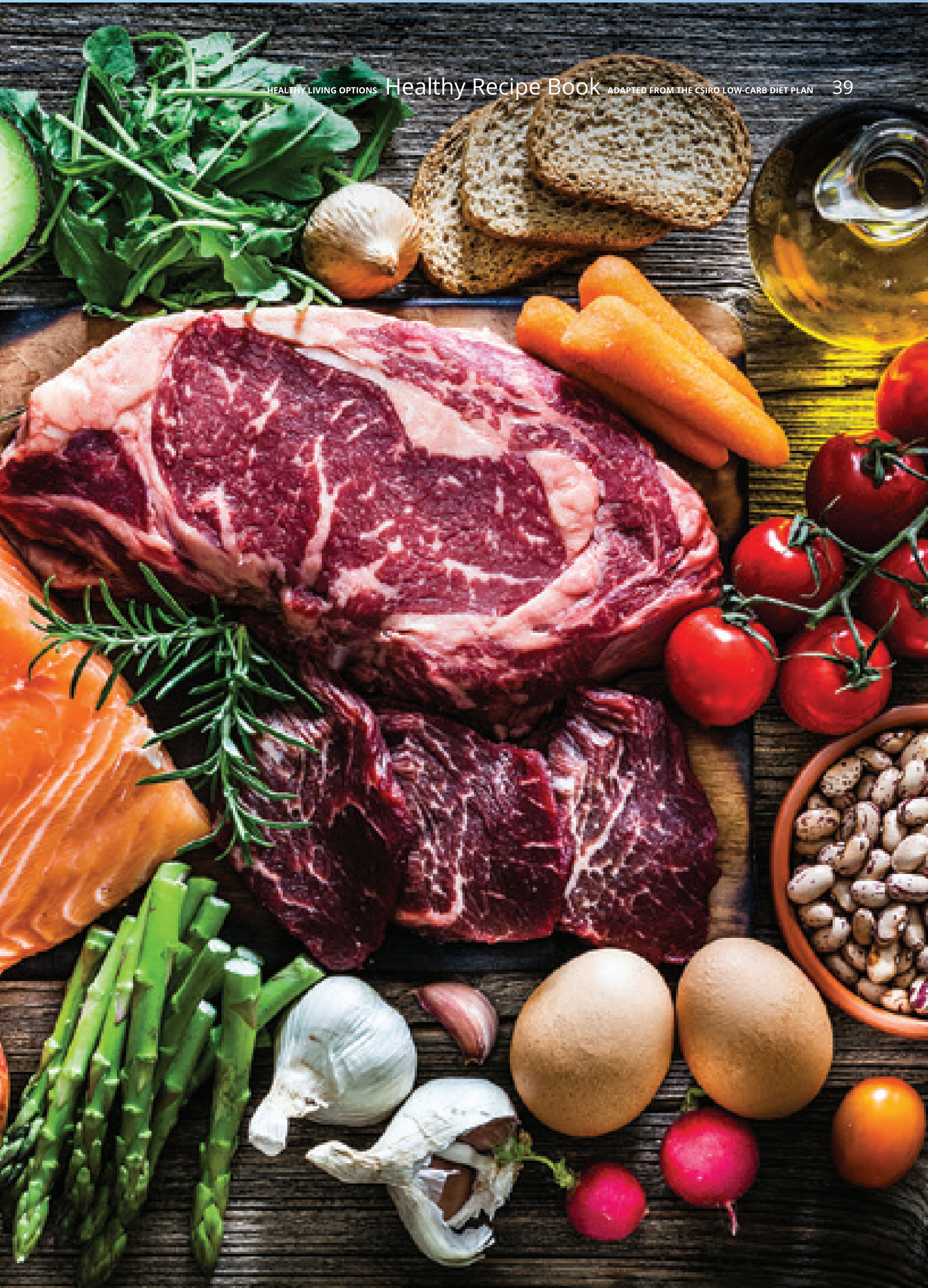

Western Sydney Diabetes
Beating Diabetes Together

**Janine Dawson**
**Diabetes Prevention Program Manager**

Telephone: (02) 8670 0015
Mobile: 0419 239 179
Fax: (02) 9851 6146
Email: janine.dawson@health.nsw.gov.au
Web: www.westernsydneydiabetes.com.au
Twitter: @wsdiabetes

The information provided in this publication is current as of February 2023. Please refer to the individual websites for the most current information and for any changes to products or services.

**© February 2023**

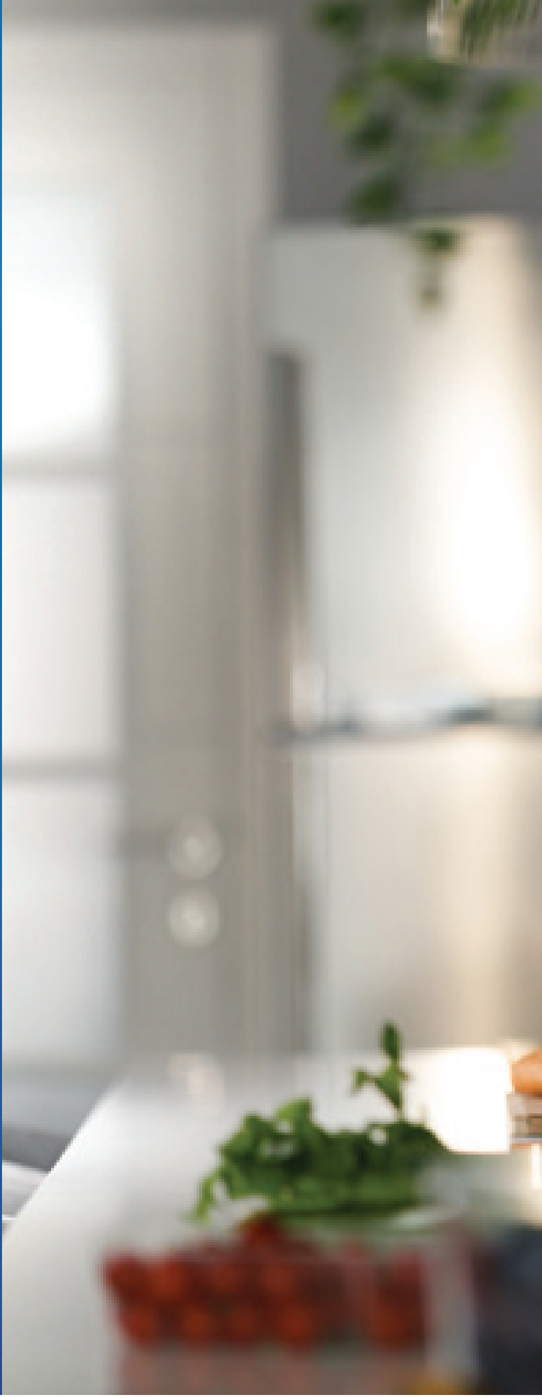

phn WESTERN SYDNEY
An Australian Government Initiative

pwc

diabetes australia

NSW GOVERNMENT
