# Supplementary material for: Integrating point-of-care diabetes detection with lifestyle counselling in community settings: outcomes from Western Sydney, Australia
Source: BMC Health Serv Res. 2024 Aug 13;24:926. doi: 10.1186/s12913-024-11335-y (PMC11323375; doi:10.1186/s12913-024-11335-y)
Supplement: Supplementary file 4 — Supplementary Material 4 [file 12913_2024_11335_MOESM4_ESM.pdf]

Western Sydney Diabetes
Beating Diabetes Together

HEALTHY LIVING OPTIONS FOR WESTERN SYDNEY

AFFORDABLE FOOD EDITION

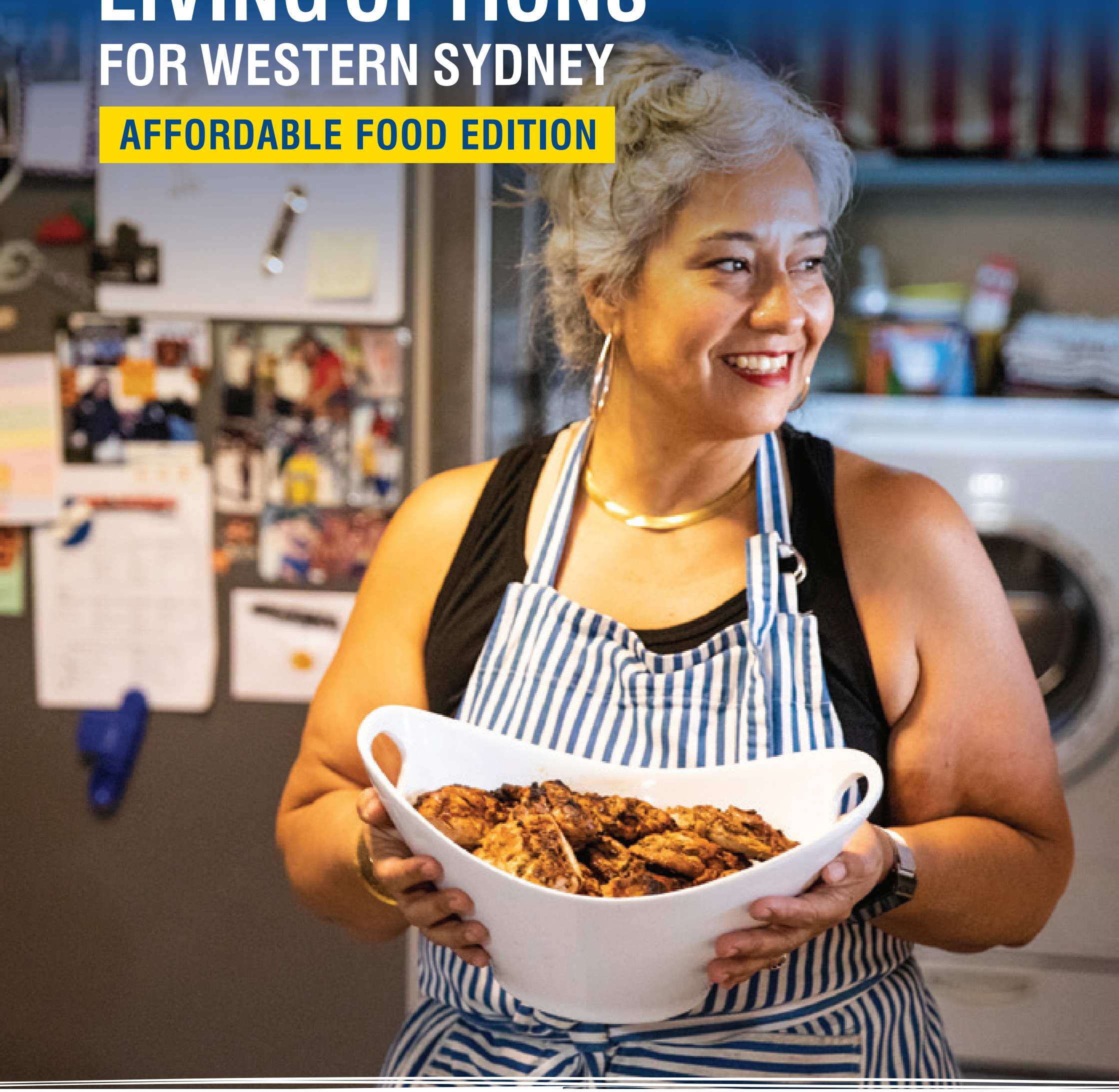

phn WESTERN SYDNEY
An Australian Government Initiative

pwc

diabetes nsw & act

NSW GOVERNMENT

About This Booklet

Improving the overall health and wellbeing of the people of Western Sydney is the aim of Western Sydney Diabetes and our Alliance partners. We want to give our residents simple and practical ways to help keep themselves and their families healthy.

We realise that sometimes it isn't easy to find the healthy option, especially when you're on a budget. For this reason we've put together this booklet which will give you some ideas on how you can find, prepare and eat healthy food, even if you're not too confident in the kitchen. We've tried to source recipes, lessons, food suppliers and classes that are either free or very inexpensive.

The programs listed here and on the Western Sydney Diabetes website are not exhaustive, but they are a starting point. Of course, due to COVID-19 things are constantly changing. We've done our best to provide up to date information, however please be aware that programs and facilities may be in a different form or maybe not available at the time you reach out to them.

Please visit our website for more information on healthy living options: www.westernsydneydiabetes.com.au

Contents

Healthy Eating on a Budget 04
Non-Cook Dinners 07
Basic Healthy Dinners 08
Reducing Food Waste 10
Buying Local Produce 11
Food Agencies 13
Learning to Cook: Face-to-Face Cooking Classes 15
Free Online Cooking Classes 18
Food Nutrition and Education Classes 21
Community Gardens 22
Finding a Dietitian 27

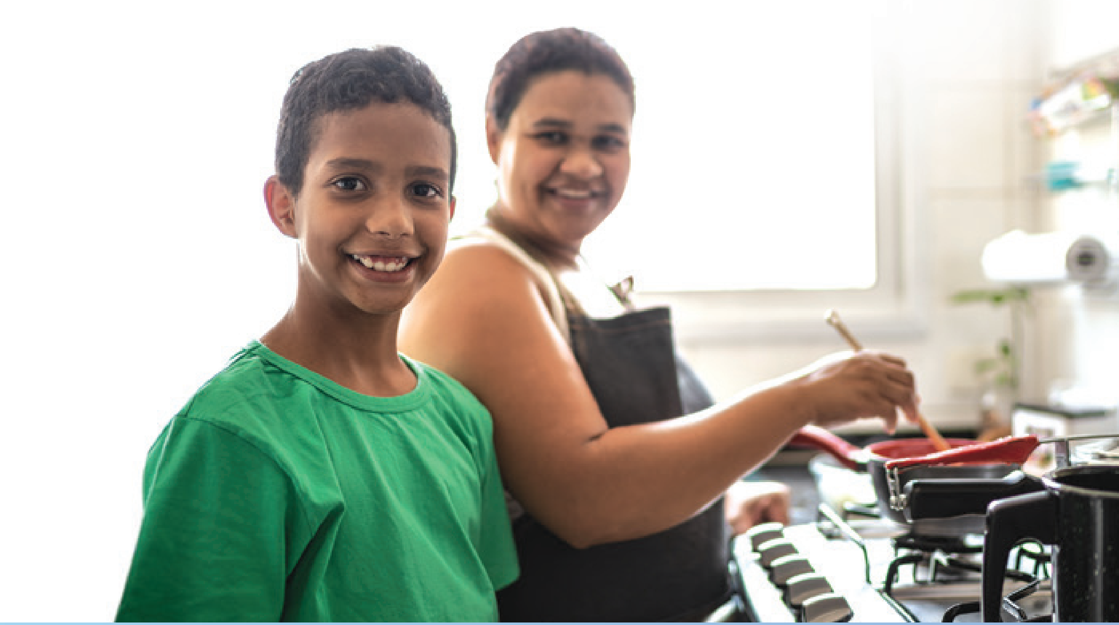

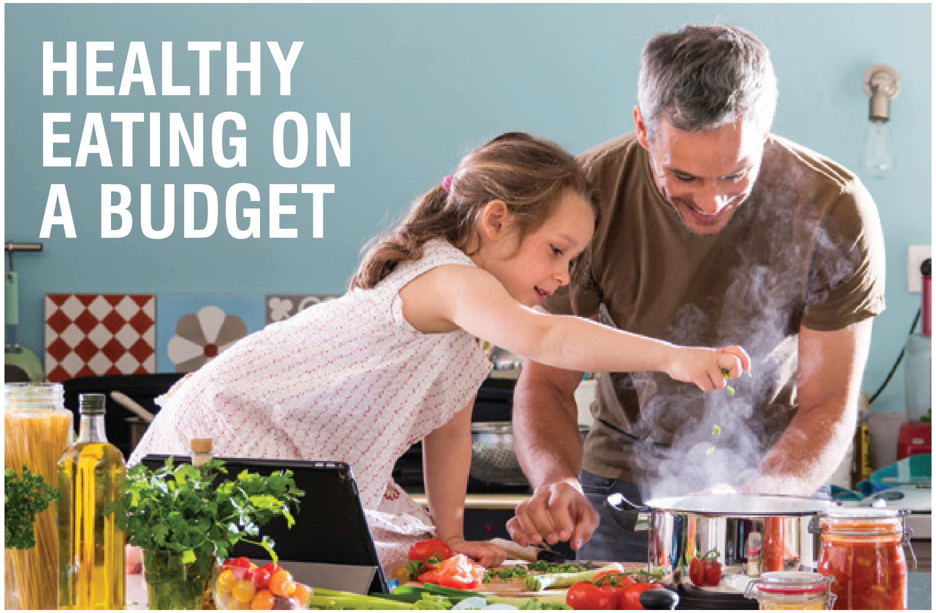

No Money, No Time

https://nomoneynotime.com.au

Looking to improve your diet? Have no money for fancy ingredients and no time to cook? This website will have you covered. Take the Healthy Eating Quiz to see where you can improve your diet and then find personalised **FREE** recipes and diet facts from leading experts in nutrition and dietetics from the University of Newcastle, Australia.

This website will help you cook healthy easy recipes. If it's not tasty, fast, cheap and packed with good stuff, you won't find it here. The recipes are constantly updated, so you can keep coming back for more inspiration. They include useful information for each recipe including the time needed to make it, the number of ingredients and the cost per person. They even outline what equipment you will need and give you tweaks that you may like to try to cater for your own taste. Along with all this, they even provide some information on why this recipe is good for you. A great resource for everyone who is busy and careful with their budget.

Australia's Best Recipes

https://www.bestrecipes.com.au/budget

This website has thousands of recipes that you may like to try. With a section devoted to 'Budget' there is plenty of choice for tasty and inexpensive meals that the whole family will enjoy.

'100 Budget Family Meals Under $5 a Serve' provides a large variety of dinners with some of the meals being prepared for as little as $3 per serve. They include one pan dinners, casseroles, satays, mornays, a selection of pasta dishes, curries, soups and stir fries. Many of these can become family dinner staples and give a base concept that can be changed to suit personal tastes.

https://www.bestrecipes.com.au/budget/galleries/cheap-family-meals-under-5-serve/hek2k6x4

You may like to sign up for their newsletter and social pages to be part of Australia's Best Recipes Budget Club where you can enter monthly competitions and receive favourite weekly specials, low-cost recipes and bargain tips.

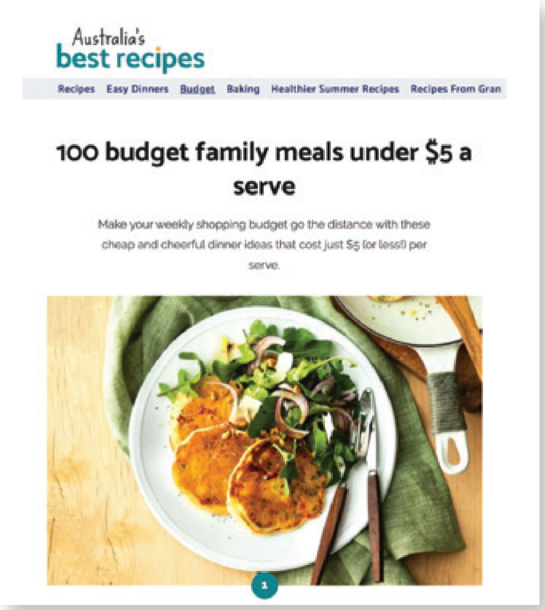

Healthy Eating on a Budget (Cont'd)

BBC Budget Recipes

https://www.bbc.co.uk/food/budget

Through this website you will be able to learn all sorts of money-saving tips, along with being able to try a large number of inexpensive recipes. Along with standard ingredients, there are also a variety of meals that you can prepare using foods from your freezer, using tinned food or just foods straight out of your pantry.

There are also tips on cutting your food waste, saving cash and reducing your food bill. A large array of family budget recipes and cheap family favourites along with 'how-to' videos will give you plenty of inspiration for preparing quick, inexpensive and delicious meals for yourself and all the family.

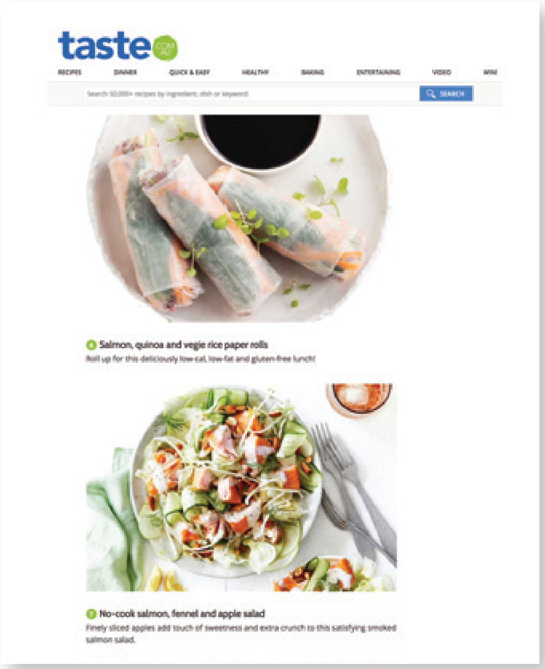

Taste

https://www.taste.com.au/recipes/collections/budget

Who says deliciously different meals have to be expensive? These cost-conscious dishes will wow your family and please your pocket at the same time.

With over 10,000 budget recipes in this collection, you are sure to find something that you would like to cook.

You can sign up for free membership, more recipes and exclusive offers.

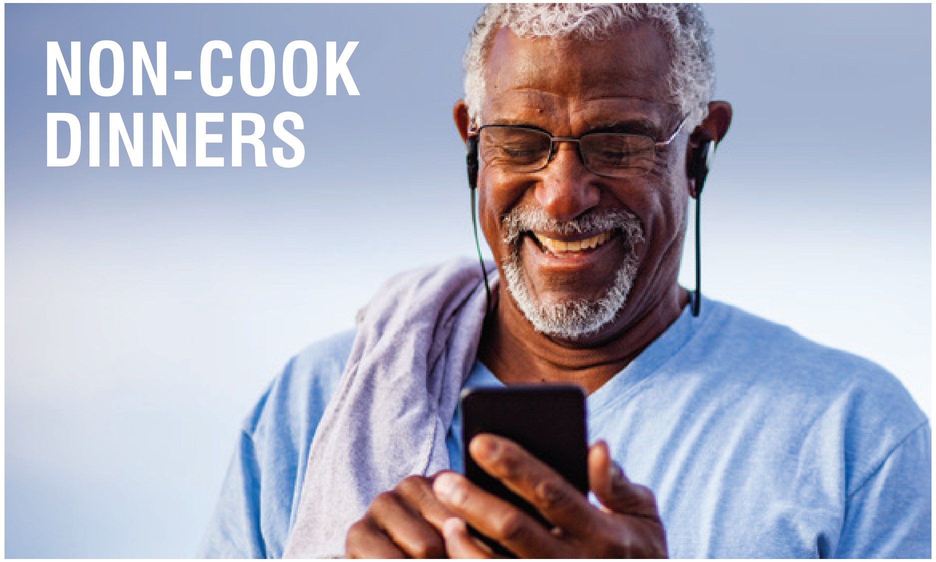

NON-COOK DINNERS

The following websites give an outstanding range of non-cook dinners. Most are healthy, very easy to prepare and require basically no cooking skills. Instead of cooking, you will chop, squeeze, slice, arrange, dollop and mix to make beautiful meals for yourself and your family.

You will be inspired to prepare tasty salads, wraps, poke bowls, snack boards, grain bowls, pitas, soups and tartines. These recipes will allow you to assemble food that may have already been cooked (such as ham), use leftovers or simply raid the pantry or fridge.

https://www.bbcgoodfood.com/howto/guide/healthy-no-cook-dinners

https://www.taste.com.au/quick-easy/galleries/our-best-ever-no-cook-dinners/l674mDpo?page=6

https://www.thekitchn.com/13-no-heat-no-cook-meals-that-still-fill-you-up-240527

https://www.delicious.com.au/recipes/collections/no-cook-dinner-recipes/aa907e5e-65bf-4113-b2f9-ac66525dc30e?page=4

BASIC HEALTHY DINNERS

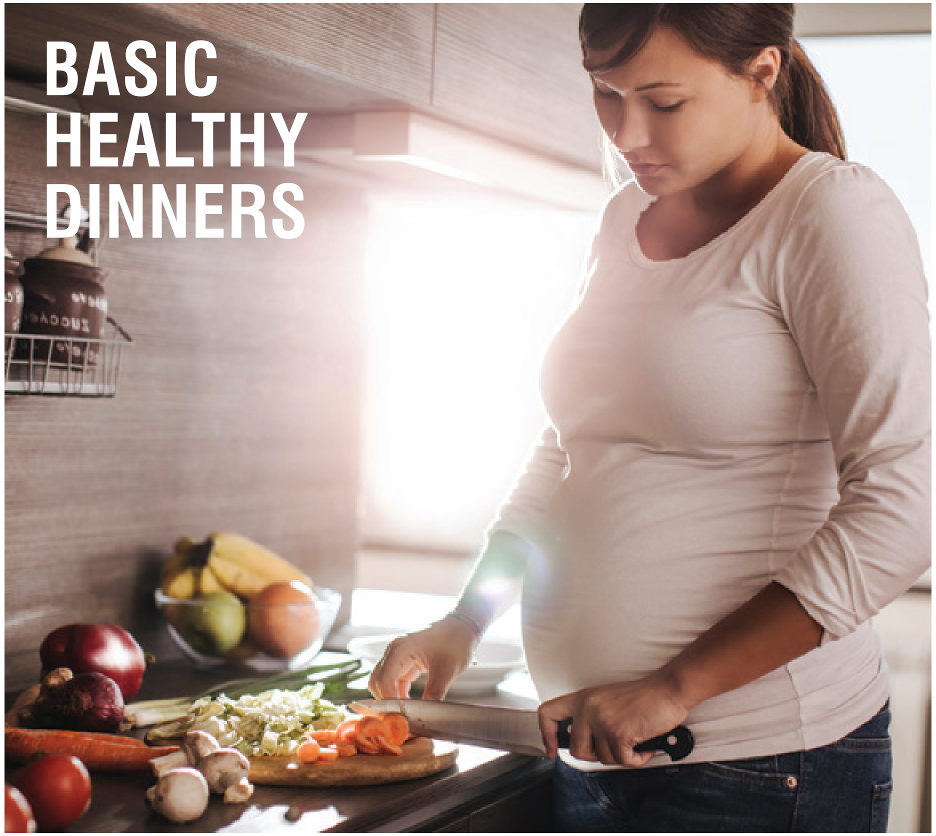

These websites are perfect for the not-too-confident cook. They provide a wide variety of very simple recipes that are quick, nourishing and easy to prepare. You can choose from soups, salads, burgers, stir fries and pasta dishes (just to name a few). You will be surprised how your skills and confidence will develop after cooking these delicious recipes.

https://www.bbcgoodfood.com/recipes/collection/quick-and-healthy

https://www.taste.com.au/recipes/collections/healthy-quick-dinner-recipes

Healthy Indian Food to Make at Home

www.rajijayadev.com.au

Move over butter chicken and lamb rogan josh. Accredited Practising Dietitian Raji Jayadev's website features 45 traditional Indian vegetarian recipes for wellness, which will leave you feeling lighter and deliciously satisfied.

Inspired by the people she's helped in hospitals around Australia and her private practice, Raji has adapted each recipe to maximise nutrition for wellness, plus suited to people living with diabetes. Each recipe has been nutritionally analysed by dietitians.

From chapatis and curries, to beverages and sweets for special occasions, Raji has curated a banquet of nutritious recipes that are easy enough to make in your home kitchen.

Australian Healthy Food Guide Magazine

https://www.healthyfoodguide.com.au

This magazine has tips and information on all kinds of healthy foods, diets, cooking skills and even foods that you can cook when you arrive home at 5pm and want to get a nutritious meal on the table.

It was voted the best food magazine in Australia in 2019 and is available at newsagents or you can subscribe. On their website, there are back issues with a library of their previous recipes, budget ideas, expert advice and health articles that you may like to look at – **FREE!**

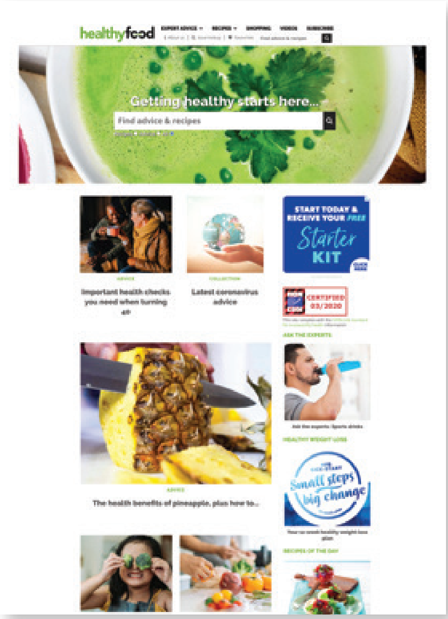

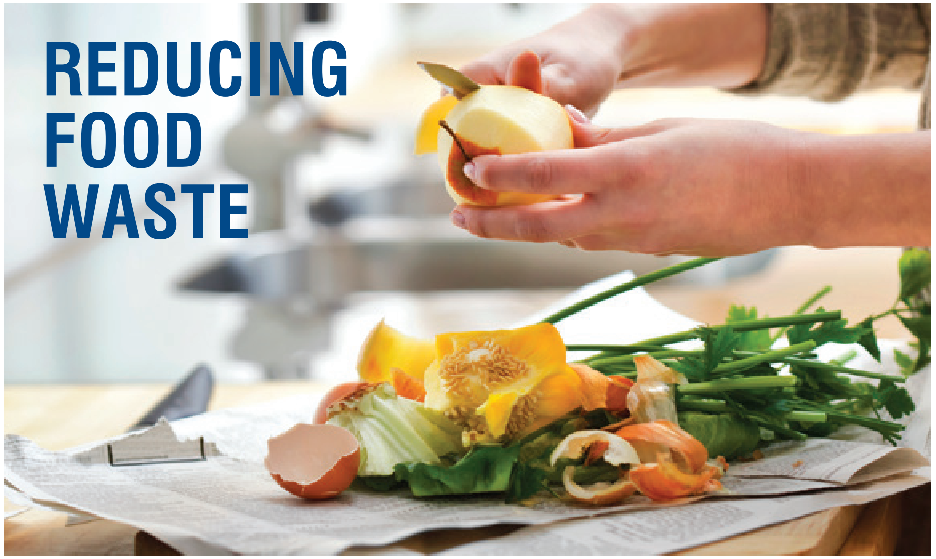

Love Food, Hate Waste

https://www.lovefoodhatewaste.nsw.gov.au/at-home/six-steps-reducing-food-waste-at-home

This website has some great ideas on how you can waste less food which is better for your pocket and the environment. To test yourself, you may like to enrol in the 2 week challenge to see how small changes can make a big difference.

Tips to Reduce your Food Waste

https://dietitiansaustralia.org.au/wp-content/uploads/2015/04/Tips-to-reduce-food-waste_final.pdf

The Dietitians Association of Australia has produced this outstanding fact sheet giving extremely useful and practical tips on how we can all reduce food waste in our homes. Not only will this help our pockets, it will also help the planet. You will find valuable tips on buying, storing, freezing and cooking all kinds of food including fruits, vegetables, meat, dairy and pantry items. This fact sheet also provides you with links to other programs and resources.

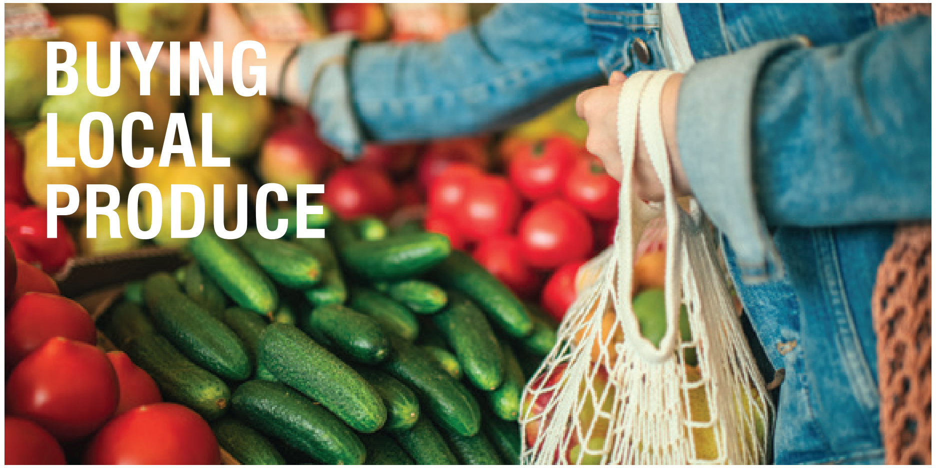

Box Divvy – A Social Enterprise

Address: Food Hubs all across Sydney
www.boxdivvy.com Tel: (02) 80065237

Cut your food bill in half! Box Divvy is the supermarket alternative where you can buy fresh food and groceries, local and Australian, 30-40% less than supermarkets because it's direct from growers and food wholesalers.

The community owns the food system whilst providing easily accessible, good quality, inexpensive food to families in the Sydney Basin. Without all the storage, food miles covered, packaging and food waste.

You can join your neighbourhood Box Divvy Food Hub where you order and pay online and either pick up from your Hubster, the person who runs it, or have your order home delivered if they offer it.

Want to be actively involved? Run a Box Divvy Food Hub and earn income from home, raise funds as a community group or as a service within a business premise. It's like running your own enterprise without the hassles. No investment required but you do need a space to pack, 2-3 hours a week dedicated to packing and communications using Box Divvy facebook page.

Interested? Either register to Join or Run a Hub at www.boxdivvy.com or email: info@boxdivvy.com

Buying Local Produce (Cont'd)

Hawkesbury Harvest

www.harvesttrailsandmarkets.com.au

Harvest Trails and Markets is a not-for-profit community incorporated organisation formed in 2000 by farmers in the Hawkesbury region of NSW.

HT&M helps connect producers directly with consumers to build alternative distribution channels for farmers, growers and producers. Their mission is to enhance the sustainability and viability of Australian agriculture, with a focus on small-scale, family farms and artisan producers. Their website provides an easily accessed directory of food, flowers and fibre, of our growing food regions and their distinctive offerings, and the diversity of artisanal products.

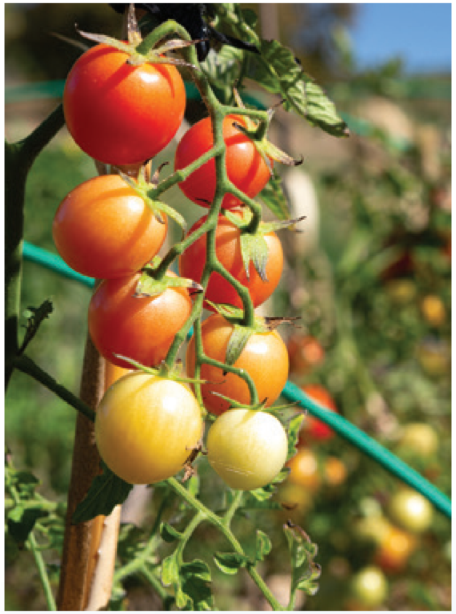

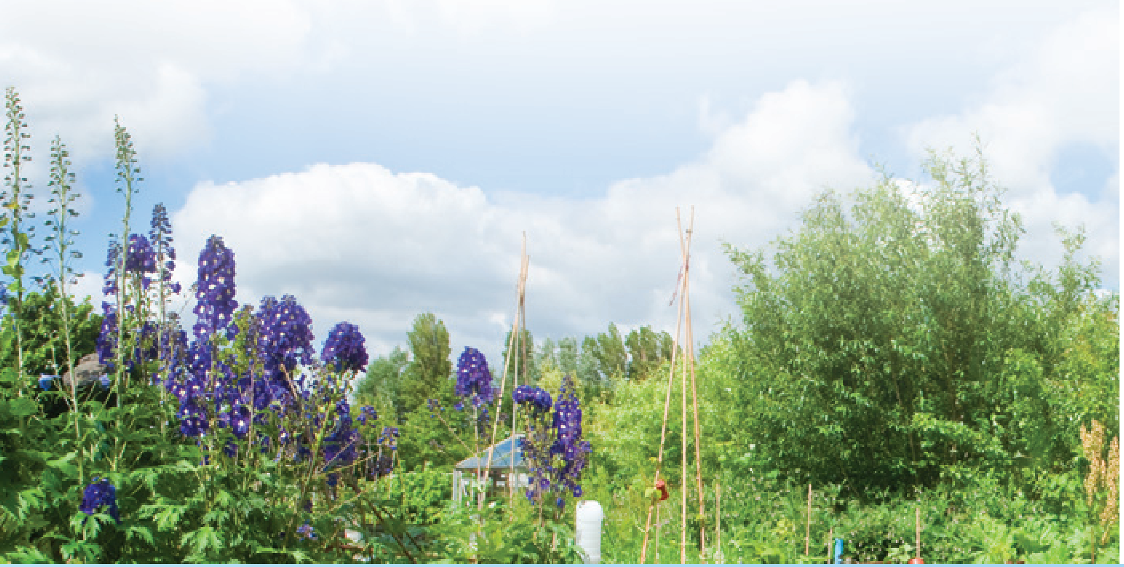

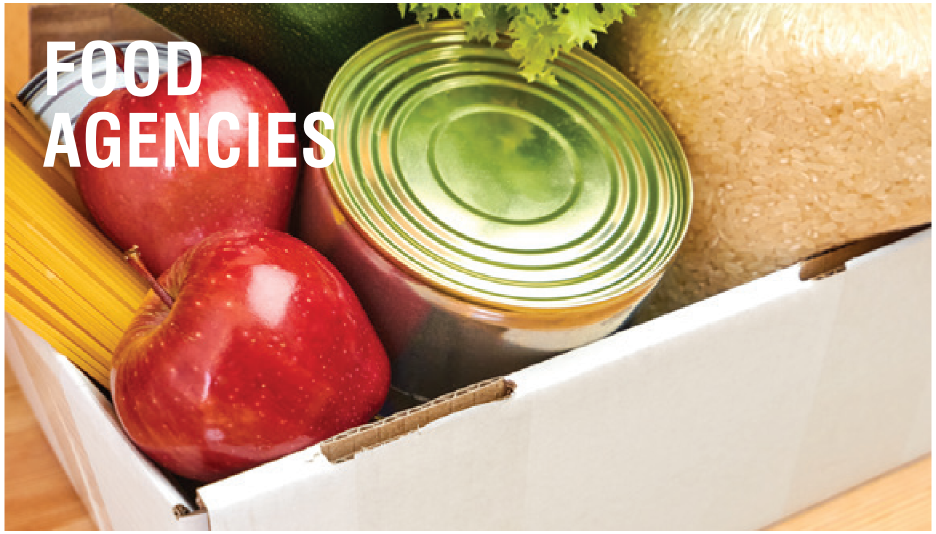

Here are some of the agencies that you may like to contact if you're having difficulty affording food:

**Reach Out**<br>Coombes Rd, Penrith<br>Contact: 0434 745 205	**3Pillars Relief Agency**<br>Mooreland Rd, Ingleburn<br>Contact: 02 8119 5126
**Your Community Needs**<br>Great Western Hwy, St Marys<br>Contact: 0468 884 717	**Mount Druitt Food Pantry**<br>Hythe St, Mount Druitt<br>Contact: 0480 154 414

Parramatta Community Care: Food Care Shop

18 Barney Street Parramatta, Friday 10am-1pm by appointment only
Phone: 9630 3892

This shop is like a 'mini-supermarket' which provides pantry items, tinned goods and fresh food at a low, affordable price. All items are heavily discounted. Centrelink and ID will be requested. This service is available to low income earners and holders of a concession card. Relief for refugees or migrants is also available.

Food Agencies (Cont'd)

Parramatta Mission

119 Macquarie Street Parramatta, Mon-Thur 2-4pm, Friday 2-3pm
Phone: 9891 2277

This service provides an 'Emergency Bag' which includes tinned goods, staples and fruit and vegetables for you to take away. ID will be requested.

Australia's Right to Food Coalition

https://righttofood.org.au/wp-content/uploads/2020/10/Food-Mapping-Resources-October-2020.docx.pdf

The Right to Food Coalition has prepared a list of publicly available food mapping and directory resources. They have included council and charity organisations who can provide local emergency food relief along with specific community directories for areas within western Sydney. Some of the information on the Council websites is very comprehensive and provides all sorts of options for sourcing inexpensive food, parcels, vouchers and prepared meals.

Foodbank NSW & ACT Limited

www.foodbank.org.au

Foodbank NSW & ACT provides food and grocery items to over 660 charities and schools who distribute food to the community. Foodbank does not provide food directly to the community, instead, they work in partnership with charities and schools to distribute the food to where it's needed most.

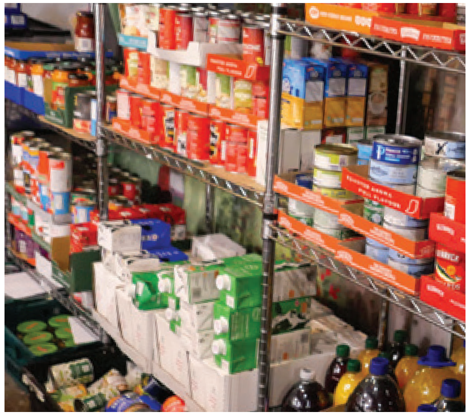

If you're in need of food assistance, please call Foodbank on **02 9756 3099** and they can help you find a charity in your area that can support you.

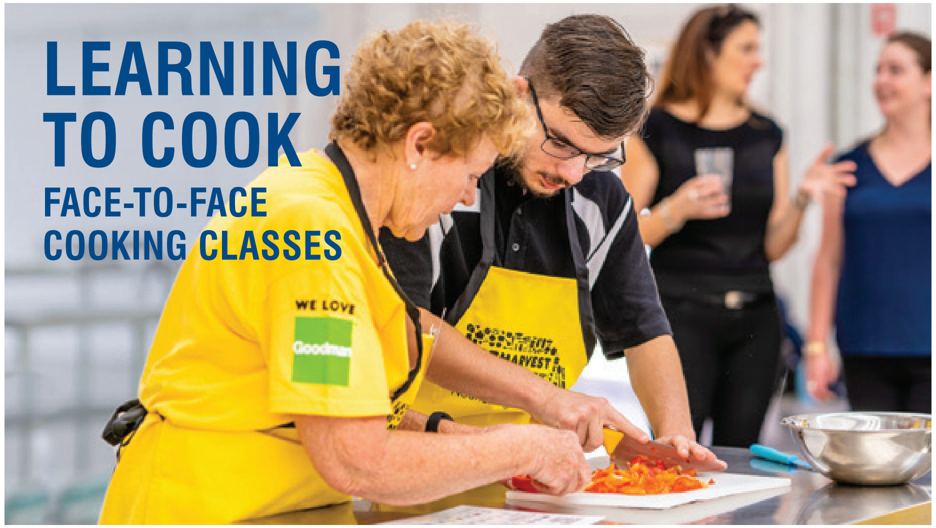

Please note that due to COVID-19, some of these classes may be currently unavailable or be conducted under different conditions.

OzHarvest

OzHarvest's NEST Program

OzHarvest's NEST (Nutrition Education and Skills Training) program is a 6-week, 15-hour public health nutrition program, designed to teach people from vulnerable backgrounds valuable life skills to help nurture their families with nutritious food on a budget. The program is run by a registered nutritionist or dietitian and combines practical cooking lessons with interactive and fun nutrition education.

Collaborating with the Institute for Physical Activity and Nutrition (IPAN) at Deakin University, OzHarvest has developed an engaging program, which aligns with the Australian Dietary Guidelines and state/territory-based healthy eating strategies.

The 6-week program takes place on the same day and time each week, with the OzHarvest team travelling to your organisation with all the cooking equipment and ingredients required. Each session runs for 2.5 hours (150 minutes) and start with a brief group discussion and themed activity. Participants then cook 2-3 recipes in small groups, then recap their learnings whilst enjoying the delicious food together.

Oz Harvest (Cont'd)

At the final NEST workshop all participants receive a certificate of completion and a take home toolkit, including nutrition fact sheets, a poster, infographics and the OzHarvest Everyday Cookbook – packed with step by step photo-based delicious recipes, which cost less than $3.00 per serve to make!

The cost of the 6-week, 15-hour program for up to 15 participants is $3,750 + GST. If you have a limited budget, please don't be put off by the full cost – let us know what your budget is, and we can discuss what we can do to help you.

For further information or to book a NEST program in Sydney please visit our website **http://www.ozharvest.org/what-we-do/nest-nutrition-education/** or contact our local NEST Program Coordinator via **Sydney.Nest@ozharvest.org**. Please also feel free to join our OzHarvest NEST Facebook Group (**https://www.facebook.com/groups/OzHarvestNEST/**) where you can access Nice Easy Simple Tips to cook and eat tasty, nutritions and affordable meals.

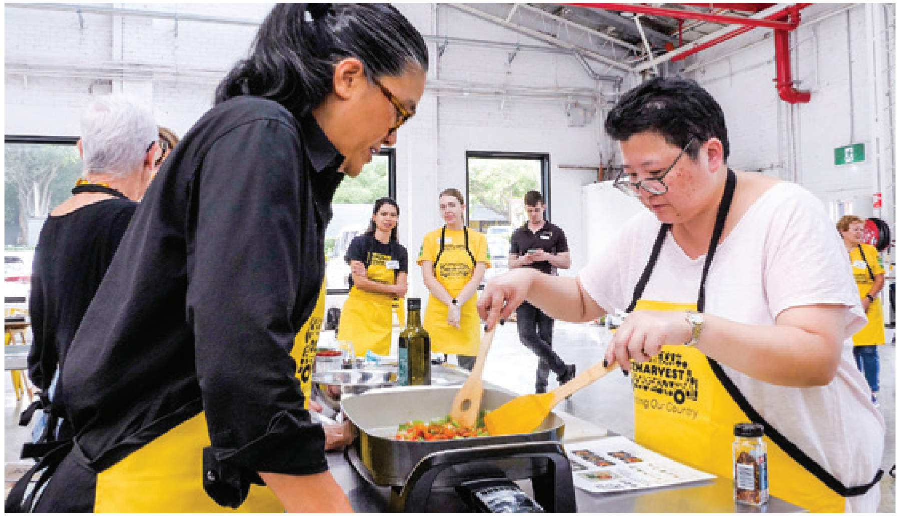

A participant cooking with chef Kylie Kwong (PHOTOS: JOYCE ONG)

TAFE

Certificate II in Kitchen Operations

Whether you're looking for an apprenticeship, returning to work or seeking a career change, the nationally recognised Certificate II in Kitchen Operations will get you cooking with gas. You will learn the basics of food preparation, storage, processes and service. You will also learn how to:

- **Prepare basic dishes using hygienic practices**
- **Manage perishable items**
- **Clean kitchen premises**
- **Fundamental cookery skills**

You will have hands-on, practical education delivered in a fully functional training restaurant. In addition, you can also learn to prepare and present items such as sandwiches, appetisers, salads, stocks, sauces, soups, vegetables, fruit and eggs, pasta, rice and noodles. Career opportunities include takeaway cook, sandwich hand, breakfast cook, catering assistant.

This qualification provides a pathway to work in kitchen operations in organisations such as restaurants, hotels, catering operations, clubs, pubs, cafes, and coffee shops; and institutions such as aged care facilities, hospitals, prisons, and schools.

This is a full time course. You will need to attend approximately 13-23 hours of class per week, over 3 days a week, for 18 weeks. As well as the in-class component, you will need to complete approximately 2.2 hours of other study per week.

This qualification is suitable for an Australian Traineeship pathway including a school based Traineeship.

This course is available at the Mount Druitt, Baulkham Hills and Ryde campuses. Subsidised fees are available for this course and you may even be eligible for the NSW Smart & Skilled Fee Free Traineeship.

https://www.tafensw.edu.au

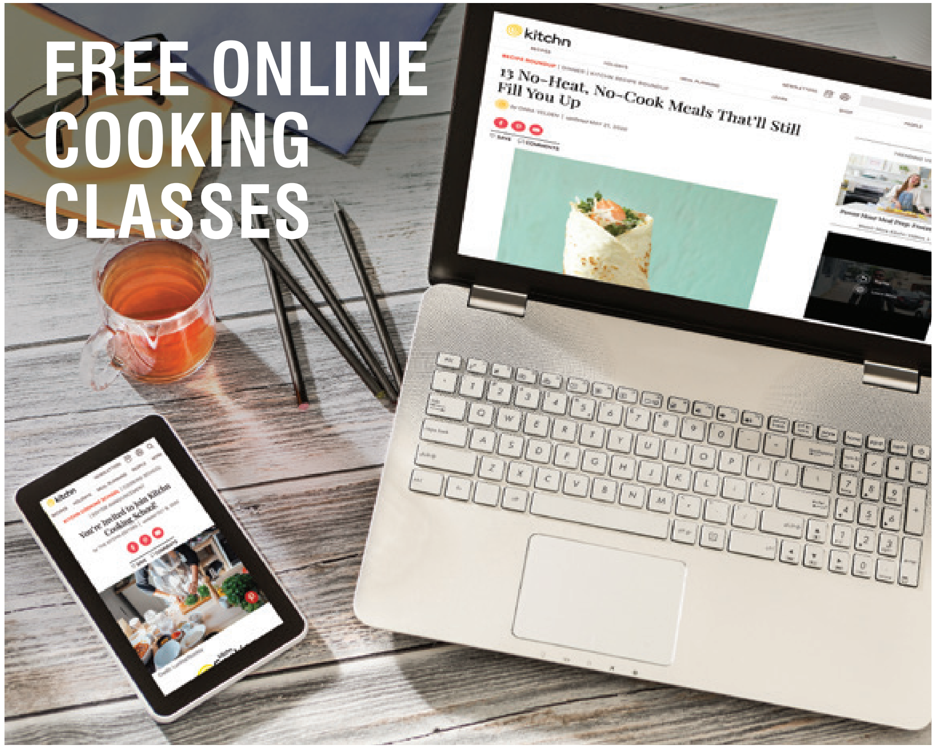

The Kitchn's Cooking School

This is a 20 lesson program where you can tackle an essential cooking topic each day. It provides videos, photos and detailed instructions. You will learn a number of techniques starting with knife skills, moving on to cooking with grains, poultry, making salads and sauces and even perfecting the presentation of your dishes. With each lesson, you'll be given a choice of assignments to try out your new skills. (**FREE** to join at the Kitchn)

https://www.thekitchn.com/how-to-join-kitchn-cooking-school-22952427

Food Techniques

https://www.bbc.co.uk/food/techniques

BBC has information on a vast selection of cooking and food preparation techniques that provide cooks of all levels with excellent information, descriptions and demonstrations. Subjects are broken up into sections including how to prepare and cook fruit and veg, meat, poultry, eggs, fish, pasta, baking and sauces. Each technique is rated with its difficulty and provides a video, written description and a number of recipes you can try which use the technique. This is really a wonderful resource for learning the very basic to advanced techniques. This website is **FREE** and there is also a selection of over 10,000 recipes on which to try out your new skills.

Dinner Tonight Texas A&M

https://dinnertonight.tamu.edu/

This website was set up in order to teach people how to make quick, inexpensive and healthy dinners for themselves and their families. It provides great information for those who haven't had much experience in cooking, so it's a great resource for those just starting out in the kitchen. This website provides recipes and also gives weekly video demonstrations of various recipes. It also provides cooking tips and techniques, nutrition topics, menu planning basics and information on healthy living.

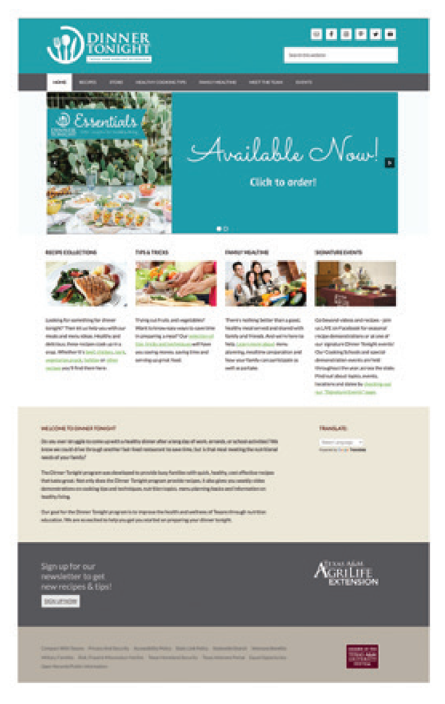

Free Online Cooking Courses (Cont'd)

Better Homes and Gardens

https://bhg.com/recipes/how-to

If you're looking to become a better cook, this website will help with its extensive cooking guide. There are cooking charts, essential for times and temperatures you need to know to cook meat, poultry, and vegetables to perfection. There are also fantastic how-to videos that allow you to see skills in action. Basic tips like how to boil potatoes to advanced skills like how to butterfly a lobster tail are all outlined in this website. (**FREE** at Better Homes and Gardens)

Delia Online Cookery School

https://www.deliaonline.com/cookery-school

Delia Online features a host of cooking technique articles of varying levels of difficulty. Each lesson has step-by-step instructions with photos. Anyone who wants to learn to cook can. Delia leads you by the hand step by step. Learn to cook in your own time at your own pace.

Instructables

https://www.instructables.com/class/Cooking-Class/

If you've ever wanted to learn how to cook, this is the class for you! By following along, you'll learn all the fundamentals of cooking you need to create delicious meals.

You will be shown which pots, pans and tools are essential in your kitchen and how to stock your kitchen pantry so you always have the ingredients on hand to make a tasty homemade meal. Along with properly outfitting your kitchen, you'll learn all about how to use a chef's knife safely and other simple ways to become a competent cook.

Once you've covered the basics, you'll be guided through eight different cooking techniques that will improve every meal you make. Each cooking technique is also paired with a recipe collection so you can practice these techniques while making a delicious meal. Take this class and you'll be feeling like a great cook in no time!

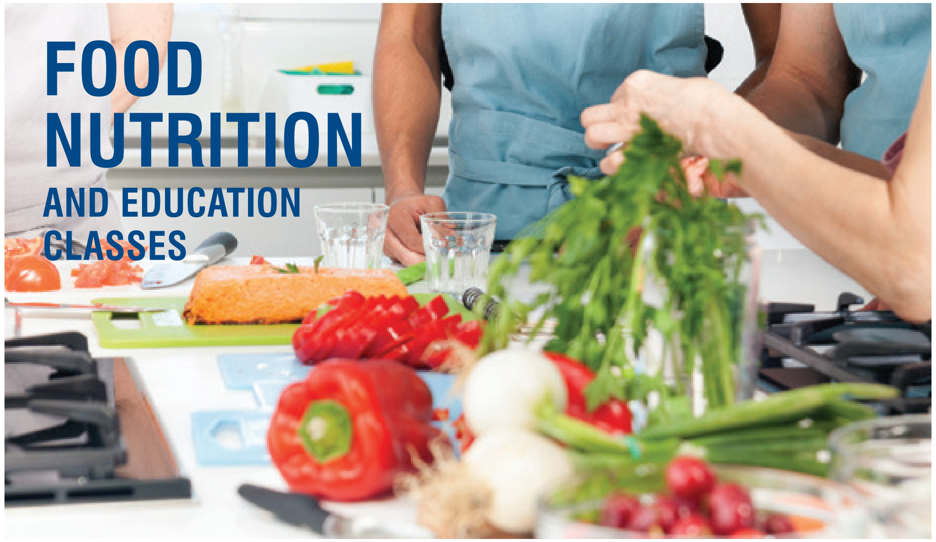

Diabetes NSW&ACT

Building Healthier Meals

Not too sure about what makes a healthy meal? This session will help you understand the basics of nutrition so you'll be able to choose and prepare a balanced meal.

You will receive practical information about:

- **Basic building blocks for balanced and nourishing meals**
- **What to drink with meals**
- **Healthy swaps**
- **Mindful eating skills to help manage portion sizes and food cravings**

For people with all types of diabetes. This event is run at a number of local venues in western Sydney throughout the year. It is **FREE** to anyone registered on the National Diabetes Service Scheme (NDSS).

For a full list of events near you, visit: https://diabetesnsw.com.au/events or phone us on **1300 136 588**

COMMUNITY GARDENS

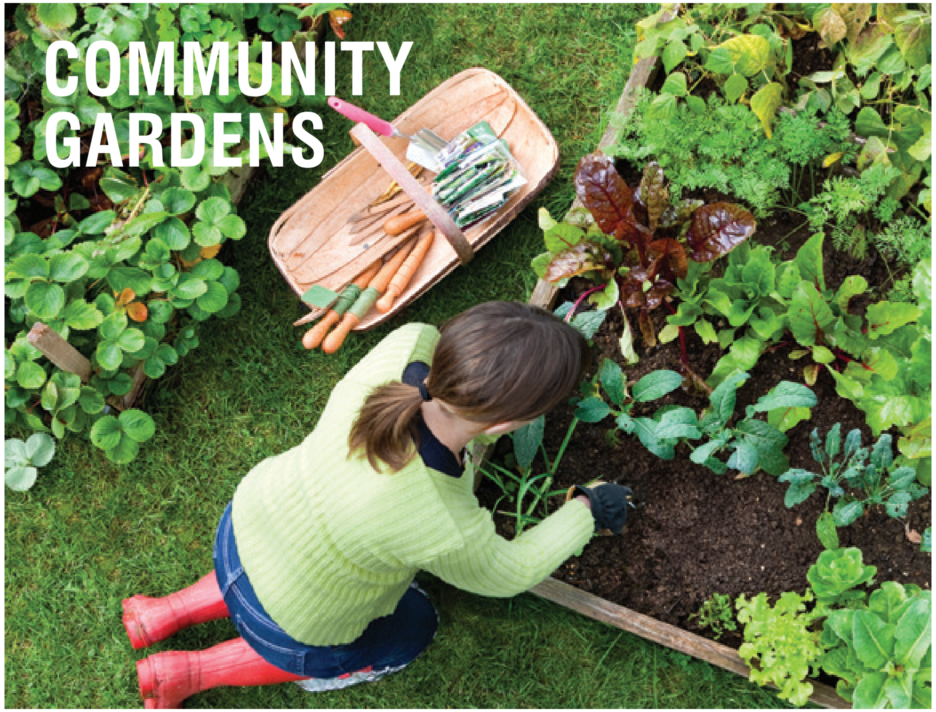

What better way to become enthusiastic about cooking than to grow your own food? Community gardens are places where people come together to grow food, create habitat and connect with nature and their local community. They are places where community members can learn new skills, help improve our community's access to fresh food, make new friends, and share in the work of maintaining the garden.

We're very fortunate that here in western Sydney there are a large number of community gardens run by groups and in many cases the local council that you may like to join. Some are purely communal, whereas others provide access to your own plots where you can grow food for your family and friends.

Here is a list of some of the local gardens. **Due to COVID-19 restrictions, check first with the garden organiser before visiting these gardens:**

Bidwill Community Garden

Location: Chestnut Crescent, Bidwill
Contact: 0415 836 531 or email: arnesenlisa.la@gmail.com
Opening times: 9am-2pm on Wednesdays, Fridays and Sundays

The garden has a number of themed garden beds of edible and non-edible plants, including vegetable gardens, orchard of fruit trees, a native patch and succulents!

Bungarribee Community Garden

Location: 20 Sir Hercules Drive, Bungarribee
Contact: ourenvironment@blacktown.nsw.gov.au
Opening times: 9:30am-11:30am Thursdays

The garden has a number of attractive productive vegetable beds, with natives and flowering plants to attract good insects. The garden also features a drip irrigation system and rainwater tank.

Lalor Park Community Garden

Location: Parkside Drive, Lalor Park
Contact: lalorparkcommunitygarden@gmail.com
Opening times: Thursday and Saturday (9am-12 noon)

The Lalor Park Community Garden is a not-for-profit community focused group run entirely by volunteers. The garden grows a variety of vegetables, fruit and herbs. We encourage the community to join our membership and garden with us.

Lindley Square Community Garden

Location: Lindley Square, Bidwill
Contact: ourenvironment@blacktown.nsw.gov.au

This is a new community garden in Bidwill that is currently in the planning and construction stages. If you would like to get involved, contact **ourenvironment@blacktown.nsw.gov.au** and we can put you in touch with the local residents who are preparing to start this garden.

Community Gardens (Cont'd)

Quakers Hill Uniting Community Garden

Location: 32 Highfield Road, Quakers Hill, Contact: 9837 2001

The Gardening Group at Quakers Hill Uniting Community Garden is a strong mix of local community members, Noah's Ark Pre-school children and parents, families from Quakers Hill Family Services and families from Quakers Hill Uniting Church. Some are whole families and some are individuals but all enjoy the invigorating joy of working in the garden and seeing the garden grow.

Riverstone Community Garden

Location: Park Street, Riverstone
Contact: Riverstone Neighbourhood Centre 9627 3622, or email: reception@riverstone.org.au Opening times: Tuesday, Wednesday, Thursday 9am-1pm

The garden has a number of community beds for organisations (Bhutanese group, Dementia Connections, Mens' Shed) and some individual garden allotments.

Showground Community Garden

Location: Blacktown Showground, Garden Gate, Richmond Road, Blacktown
Contact: ourenvironment@blacktown.nsw.gov.au
Opening times: Tuesday 9am-11:30am, first and third Saturday each month 8:30-11:30am, second and fourth Sunday each month 8:30-11:30am

Productive diverse gardens for growing vegetables, fruit trees and native plants and shrubs. Irrigation system to water the garden, with shared gardening activities. This garden has concrete paths and raised garden beds to help people with disabilities access them.

The Ponds Community Garden

Location: The garden will be located next to The Ponds Community Hub, 45 Riverbank Drive, The Ponds, Contact: ourenvironment@blacktown.nsw.gov.au

This is a new community garden in The Ponds that is currently in the planning stages. If you would like to get involved, then contact ourenvironment@blacktown.nsw.gov.au and we can put you in touch with the local residents who are preparing to start this garden.

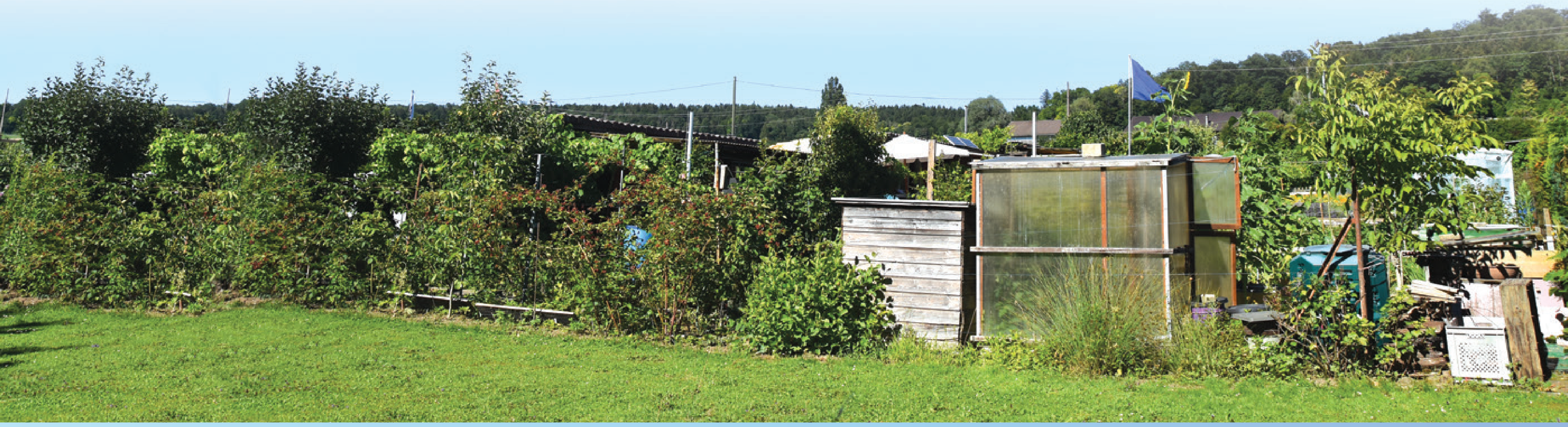

Willmot Community Garden

Location: Willmot Public School, Contact: 9628 0222

Community garden cared for by the community and the school. Please contact the School Office for more details.

Wentworthville Community Garden

Address: 73 Fullagar Rd, Wentworthville
Opening times: Wednesdays 3:30-4:30pm, Saturday 10am-1pm and Sundays 3:30-4:30pm, weather permitting. Contact: 0420 893 186

Wentworthville Community garden is a place where you can grow your own veggies, spend time with others or simply sit and relax.

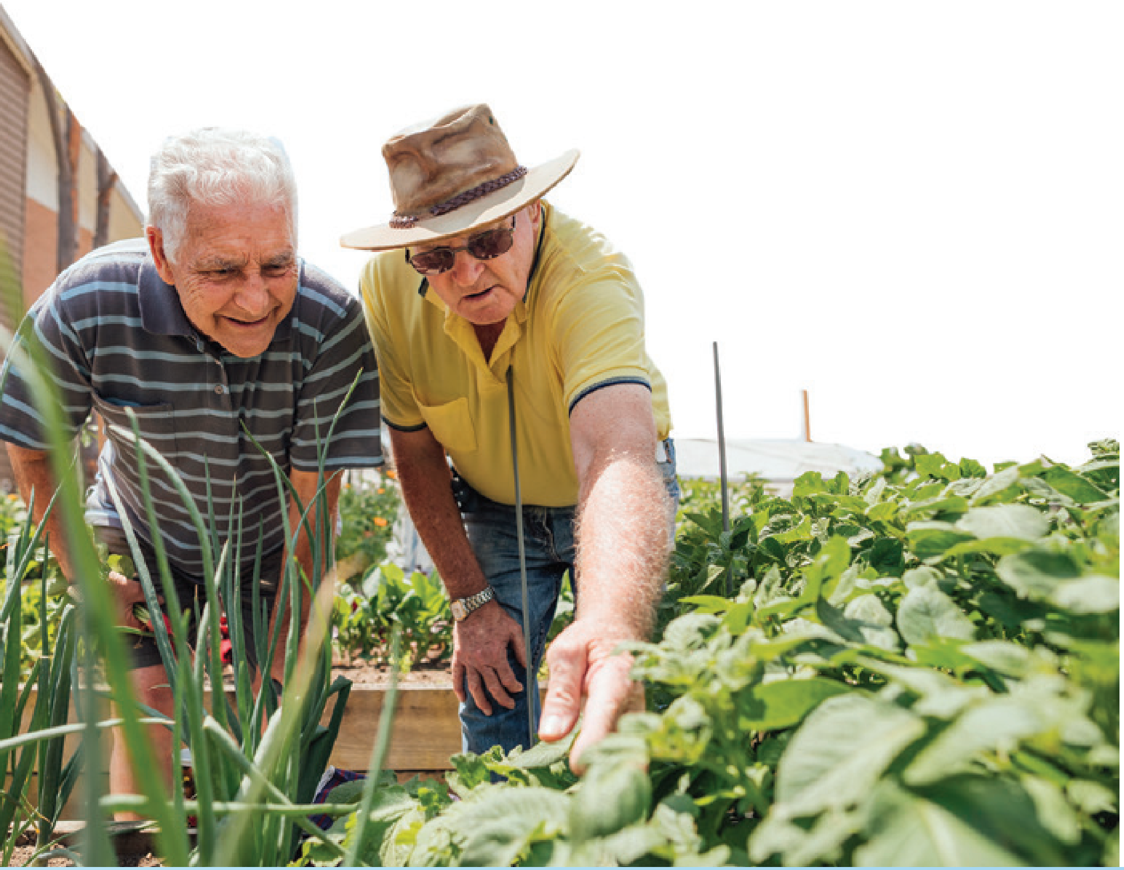

FINDING A DIETITIAN

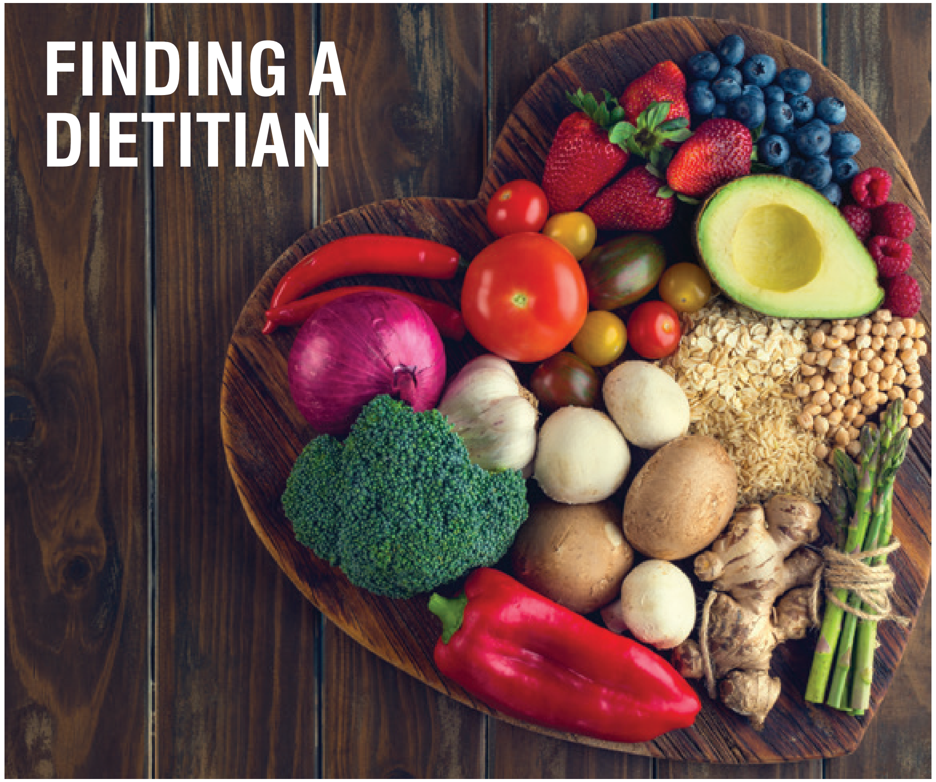

https://dietitiansaustralia.org.au/find-an-apd/

It's not always easy to know which foods are best for your health and this can be especially difficult if you have certain medical conditions, dietary requirements, or come from another country with a specific cuisine.

Help is available. The Dietitians Australia website has a comprehensive directory where you can not only find an accredited practising dietitian in your chosen geographic area, but also find one who can speak languages other than English.

Western Sydney Diabetes
Beating Diabetes Together

Janine Dawson
Diabetes Prevention Program Manager

Telephone: (02) 8670 0015
Mobile: 0419 239179
Fax: (02) 9851 6146
Email: janine.dawson@health.nsw.gov.au
Web: www.westernsydneydiabetes.com.au
Twitter: @wsdiabetes

The information provided in this publication is current as of March 2021. Please refer to the individual websites for the most current information and for any changes to products or services.

© March 2021

phn WESTERN SYDNEY
An Australian Government Initiative

pwc

diabetes nsw & act

NSW GOVERNMENT
