# Supplementary material for: Integrating point-of-care diabetes detection with lifestyle counselling in community settings: outcomes from Western Sydney, Australia
Source: BMC Health Serv Res. 2024 Aug 13;24:926. doi: 10.1186/s12913-024-11335-y (PMC11323375; doi:10.1186/s12913-024-11335-y)
Supplement: Supplementary file 5 — Supplementary Material 5 [file 12913_2024_11335_MOESM5_ESM.pdf]

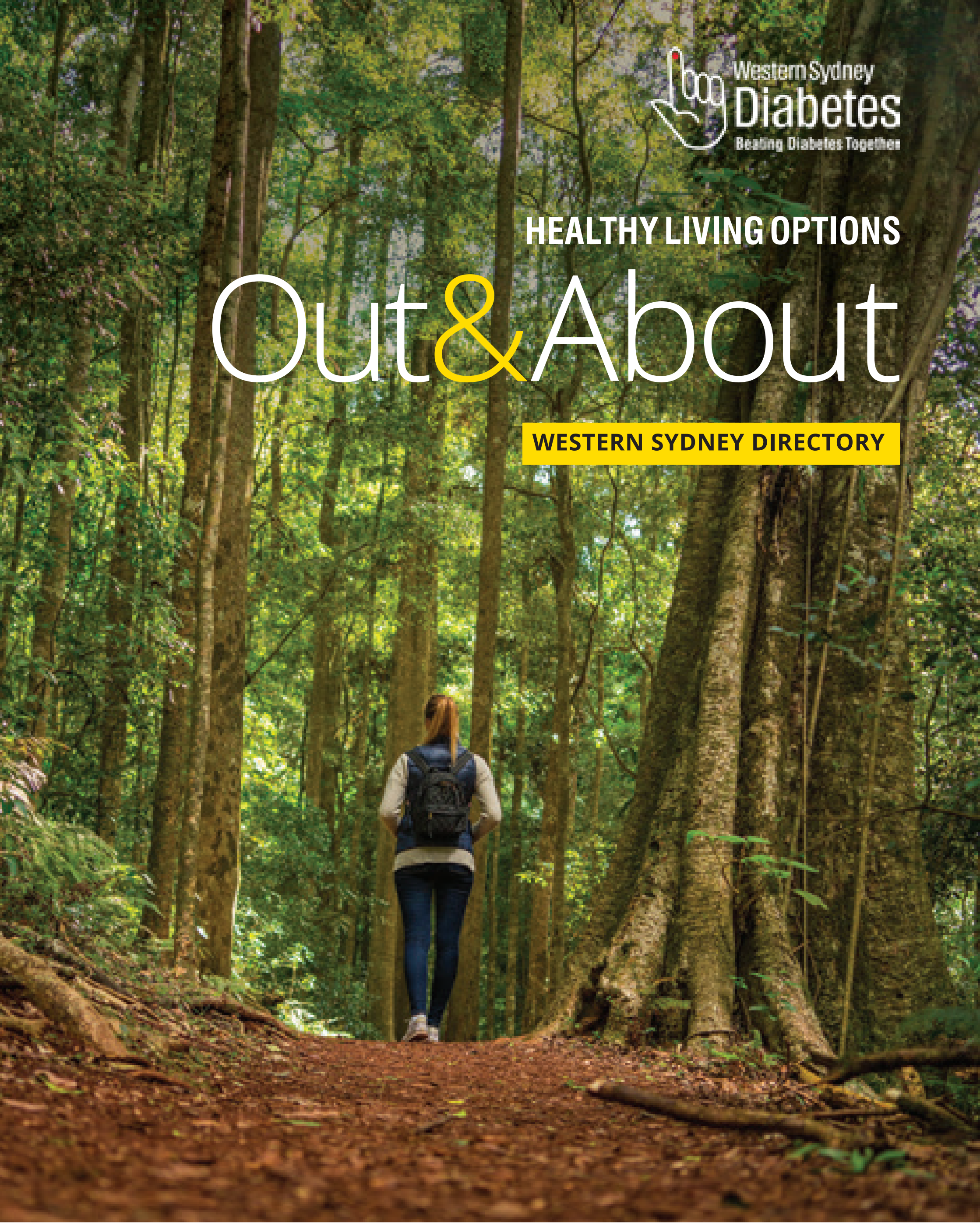

Western Sydney Diabetes

Beating Diabetes Together

HEALTHY LIVING OPTIONS

Out&About

WESTERN SYDNEY DIRECTORY

phn WESTERN SYDNEY

An Australian Government Initiative

pwc

diabetes nsw & act

NSW GOVERNMENT

Foreword

Western Sydney Diabetes has produced this booklet with the help of Alliance members and community organisations to encourage our residents to explore and enjoy the array of outdoor activities available in western Sydney.

This isn't a complete list but we hope it will give every family the inspiration and a few ideas to go outside and see or try something new.

Getting outdoors, keeping active, and eating well all contribute to the prevention of type 2 diabetes.

We have included a range of outdoor facilities, as well as where you can learn new skills to enjoy them. We understand that sometimes it's more fun to do activities in groups, so have added a range of clubs which you may want to join.

We also understand that a lot of people don't have the opportunity to connect with nature as much as they would like. For this reason, we've added ways that you may want to experience or protect our natural environment.

We hope everyone finds something in this booklet they would like to do and it gives them pleasure.

Please visit our website for more information and copies of our Healthy Living Options and Affordable Food booklets: www.westernsydneydiabetes.com.au

Janine Dawson

Primary Prevention Manager
Western Sydney Diabetes

Contents

Section 1 – Visit outdoor venues 2

Councils 2
State Forest 11
National Parks 12
Outdoors NSW&ACT 16
Parramatta Park 17
Robinhood Fitness Equipment 19
Sydney Olympic Park 20
Toilet Finder 22
Western Sydney Parklands 22
Wildwalks 26

Section 2 – Learn outdoor skills 28

Bikeriding 28
Bushcraft/camping 30
Physical skills 34
Land based skills 35
Water skills 37

Section 3 –Clubs to join 46

Section 4 – Engage with nature 58

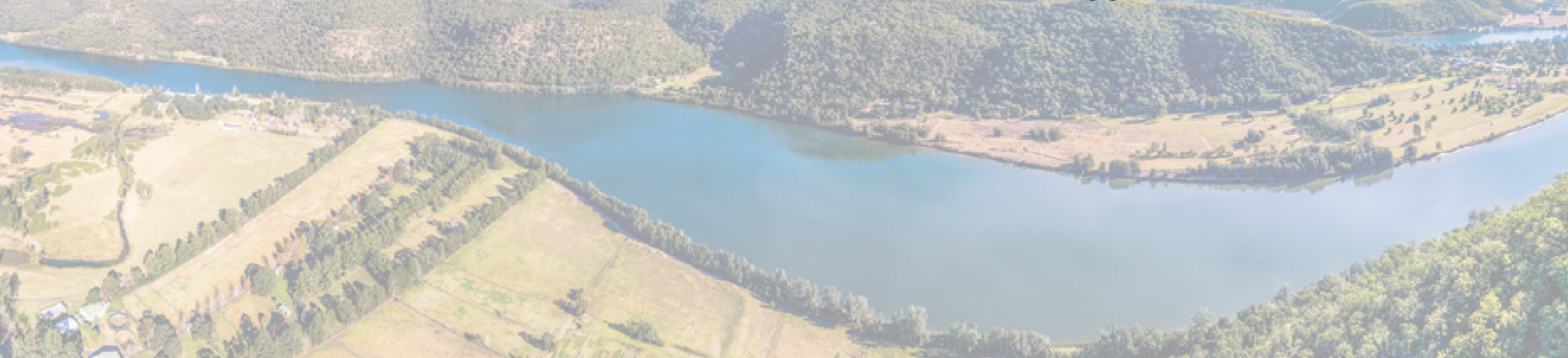

Section 1

VISIT OUTDOOR VENUES

Blacktown City Council

Blacktown City Council has an abundance of well-maintained parks, reserves and facilities for recreation. These are outlined in the extensive directory: **https://www.blacktown.nsw.gov.au/Sport-and-recreation/Parks-and-recreation-directory**

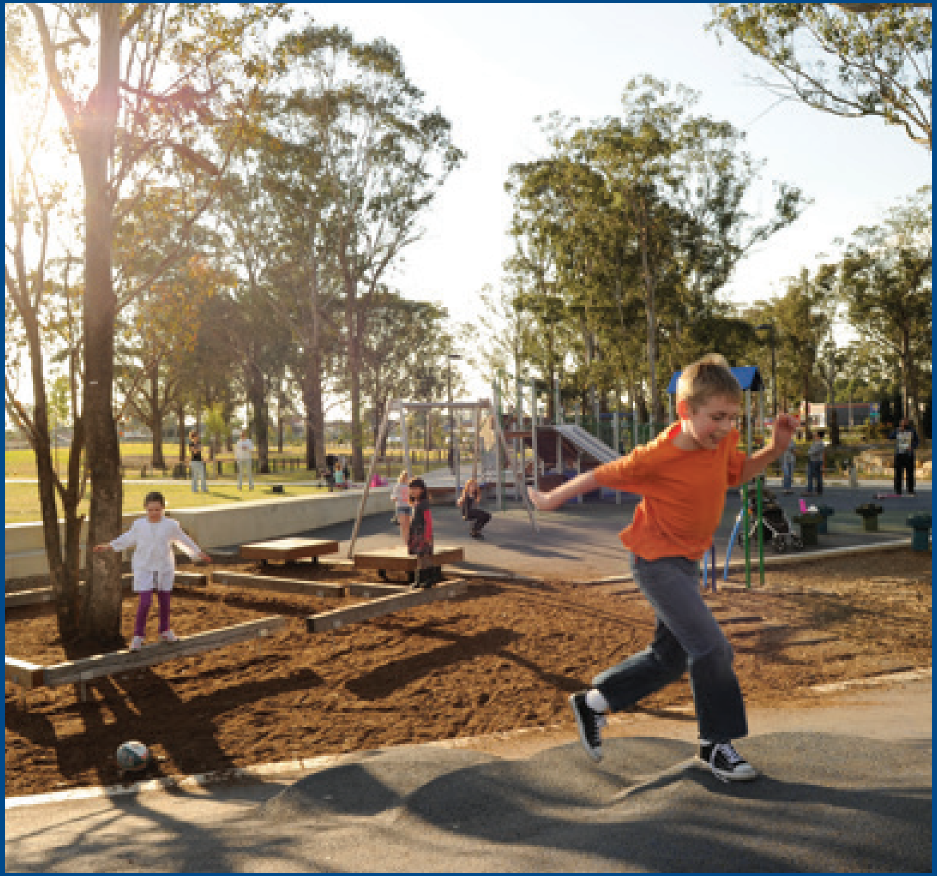

Courtesy of Blacktown Council

Parks

All 318 parks are included and you can refine your search to find local parks, discover new places to explore and pick the perfect park for you, your kids, friends, family and even your dog.

You can do this by selecting a:

- Phrase/keyword
- Suburb
- Category – parks and gardens, recreation reserve, nature, sports ground
- Feature – such as sports/playing fields, walking/bike paths, playgrounds, skate parks, waterplays, fitness equipment, or even a community garden

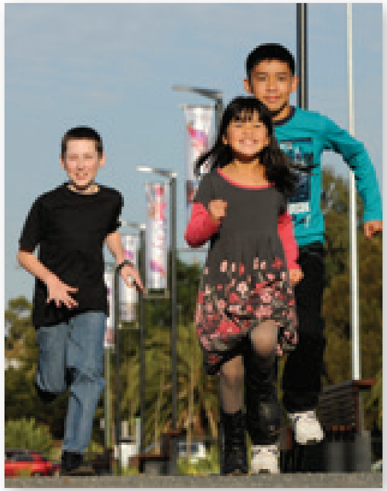

Courtesy of Blacktown Council

The website will tell you who the park or reserve is suitable for and what kind of facilities and equipment is available. It also provides a snapshot of how accessible it is and whether it has additional features such as any sporting equipment, picnic tables, toilets, walks, or water refill stations.

You can find out where you can take your dog to an off-leash park and discover water play parks available for the kids. If you're keen on walking, a very useful brochure 'Walking in Blacktown City', can be downloaded. This resource is also available in Arabic, Chinese, Nepalese, Punjabi and Tamil.

Sport and Leisure facilities

You may also wish to access our sport and leisure facilities, including walking, running, cycling, skateboarding, outdoor fitness equipment, dog off-leash areas, play spaces and outdoor courts. This can be viewed by visiting: https://www.blacktown.nsw.gov.au/Sport-recreation/Sport-and-Leisure

Some of the specific recreation facilities you can access in Blacktown include:

Aquatic and Leisure Centres

Council has 5 aquatic and leisure centres across the city. You can access the details of these facilities by visiting: https://www.blacktown.nsw.gov.au/Services/Aquatic-and-leisure-centres

Great West Walk

The Great West Walk is a network of reserves, parks, footpaths, cycleways and bush trails between Parramatta and Katoomba.

A significant part (34 km) of this walk is within Blacktown City. Starting from McCovy Street, Seven Hills at the railway overbridge, it continues through Marayong, Doonside, Rooty Hill, Mount Druitt, Whalan and Tregear through to Mainwaring Street, Ropes Crossing in Wianamatta Regional Park.

This east to west trail can be easily completed in small selected sections from, and to, many of our parks and reserves. More info on the Great West Walk and other walking, running and cycling networks in Blacktown can be found by visiting: https://www.blacktown.nsw.gov.au/Sport-recreation/Sport-and-Leisure/Walk-run-cycle

Community Gardens

Community gardens are places where you can grow food, create habitat and connect with nature and other community members.

Community gardens can be located on different types of land including schools, churches, community centres and public parks. They can have fruit trees, vegetables, flowers, insect hotels, bee hives, chickens and frog ponds.

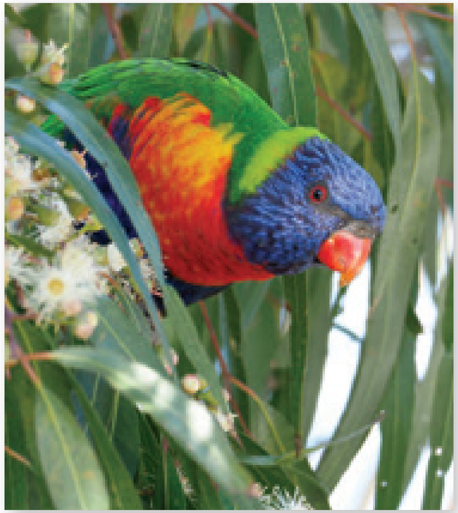

They are places where community members can learn new skills, help improve our community's access to fresh food, make new friends, and share in the work of maintaining the garden. More information on community gardens can be found by visiting: https://www.blacktown.nsw.gov.au/Community/Sustainable-living/Community-gardens

Cumberland City Council

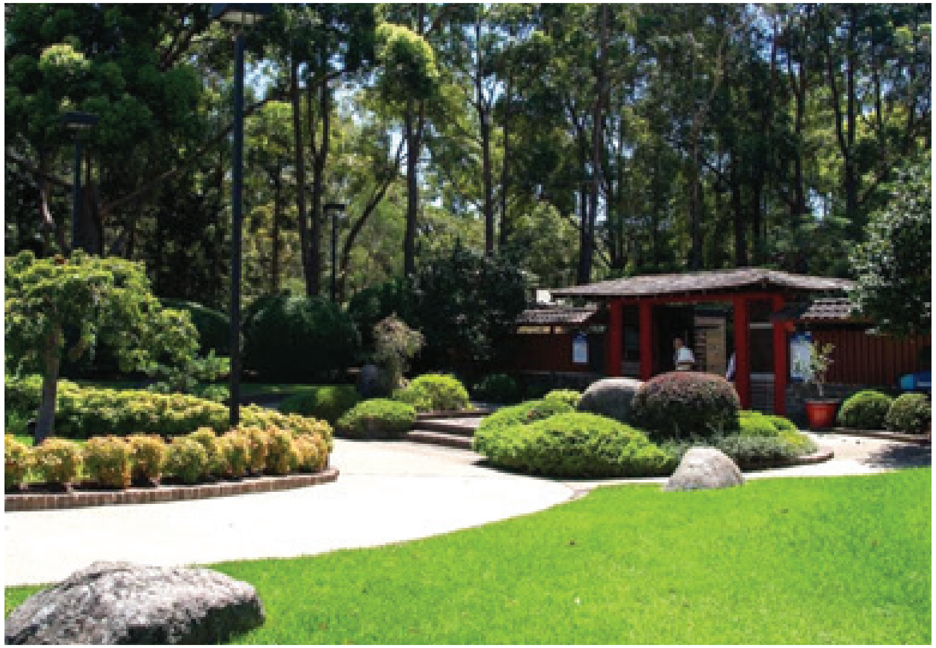

Source: Cumberland Council website

Getting out and about in Cumberland City Council is made easy with this helpful website: https://www.cumberland.nsw.gov.au and click on 'Play. You will be given a choice.

Local Events

Discover what's happening with events, online events, activities and webinars for everyone. Includes Healthy Kids and Youth programs: What's On | Cumberland City Council website: https://www.cumberland.nsw.gov.au/whats-on

Parks and Gardens

This section outlines the 3 major parks in the Cumberland LGA: the stunning Auburn Botanic Gardens, Central Gardens Nature Reserve and Holroyd Gardens Nature Reserve: https://www.cumberland.nsw.gov.au/parks-and-gardens

Playgrounds

This will give you an extensive list by suburb – you can see a photo of the park, along with a Google map to help find it, as well as a street view which will allow you to see what equipment is available and the surrounding area.

https://www.cumberland.nsw.gov.au/playgrounds

Swimming pools

This provides details of the 5 swimming centres within the district (Auburn, Granville, Guildford, Lidcombe and Wentworthville) including where they are, what's available in each pool, opening hours, costs, classes and facilities.

https://www.cumberland.nsw.gov.au/swimming-pools

Outdoor Fitness

Keep fit and exercise for free – improve your fitness and strength at an outdoor fitness gym, available at local park locations.

Outdoor fitness gyms | Cumberland City Council website: https://www.cumberland.nsw.gov.au/outdoor-fitness-gym-locations

Community Gardens

Wentworthville, Merrylands and Auburn Friendship Garden – Community gardens are places where people come together to grow fruit and vegetables, enjoy healthy exercise, meet new people and share fresh food. They allow people to connect with nature and participate in their local community.

Get involved with our environment | Cumberland City Council website: https://www.cumberland.nsw.gov.au/get-involved-our-environment

Environment

Bushcare, Sustainability Workshops

Get involved with our environment | Cumberland City Council website: https://www.cumberland.nsw.gov.au/get-involved-our-environment

Walking/Cycling Tracks and Trails

Warali Wali Trail | Cumberland City Council website: https://www.cumberland.nsw.gov.au/warali-wali-trail

Lower Prospect Canal cycleway

Also check out Walking Volunteers for walking tracks across Western Sydney:
The Walking Volunteers: https://walkingvolunteers.org.au/

The Hills Shire Council

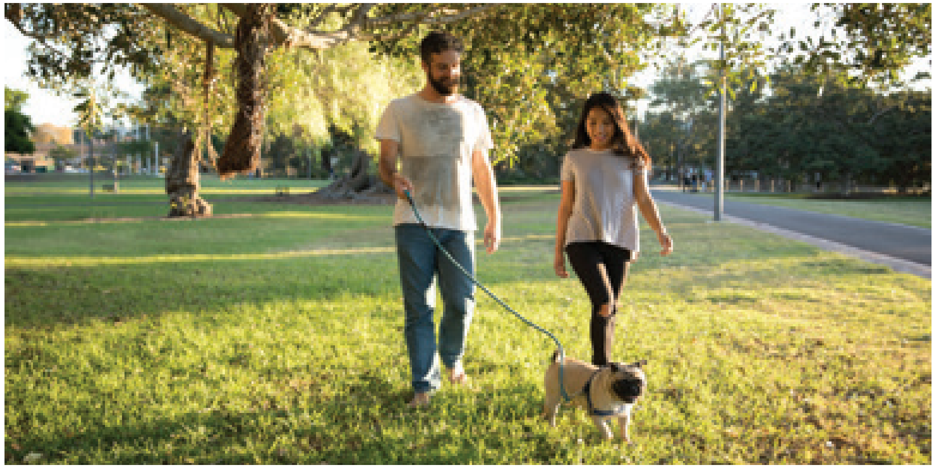

There are 300 parks and reserves in The Hills Shire Council area and this directory allows you to search for the ones that suit your requirements. You can search by suburb, category or for a specific feature. There is a description of each park along with pictures, features and the location on a Google map. You can also find your local off-leash dog parks and skate parks in this directory.

https://www.thehills.nsw.gov.au/Venues/Parks-Recreation/Parks-Recreation-Directory

The Hills Shire has a range of beautiful recreation locations including great cycleways and walking tracks. These walks take you to a number of small lakes and creeks lines where you can see plenty of bird life and wildlife, as well as visit a number of parks and playgrounds to stop in and have a leisurely break with the family.

There are also numerous bushwalk fact sheets featured which provide detailed information including starting points, distances, difficulty levels, step-by-step directions, time to complete and points of interest along the way.

https://www.thehills.nsw.gov.au/Venues/Parks-Recreation/Tracks-Trails

In addition to the bushwalks you might like to explore walks between the Hills metro stations. Discover more about your local community and get active in the process.

https://www.thehills.nsw.gov.au/Venues/Parks-Recreation/Metro-Walks-in-The-Hills

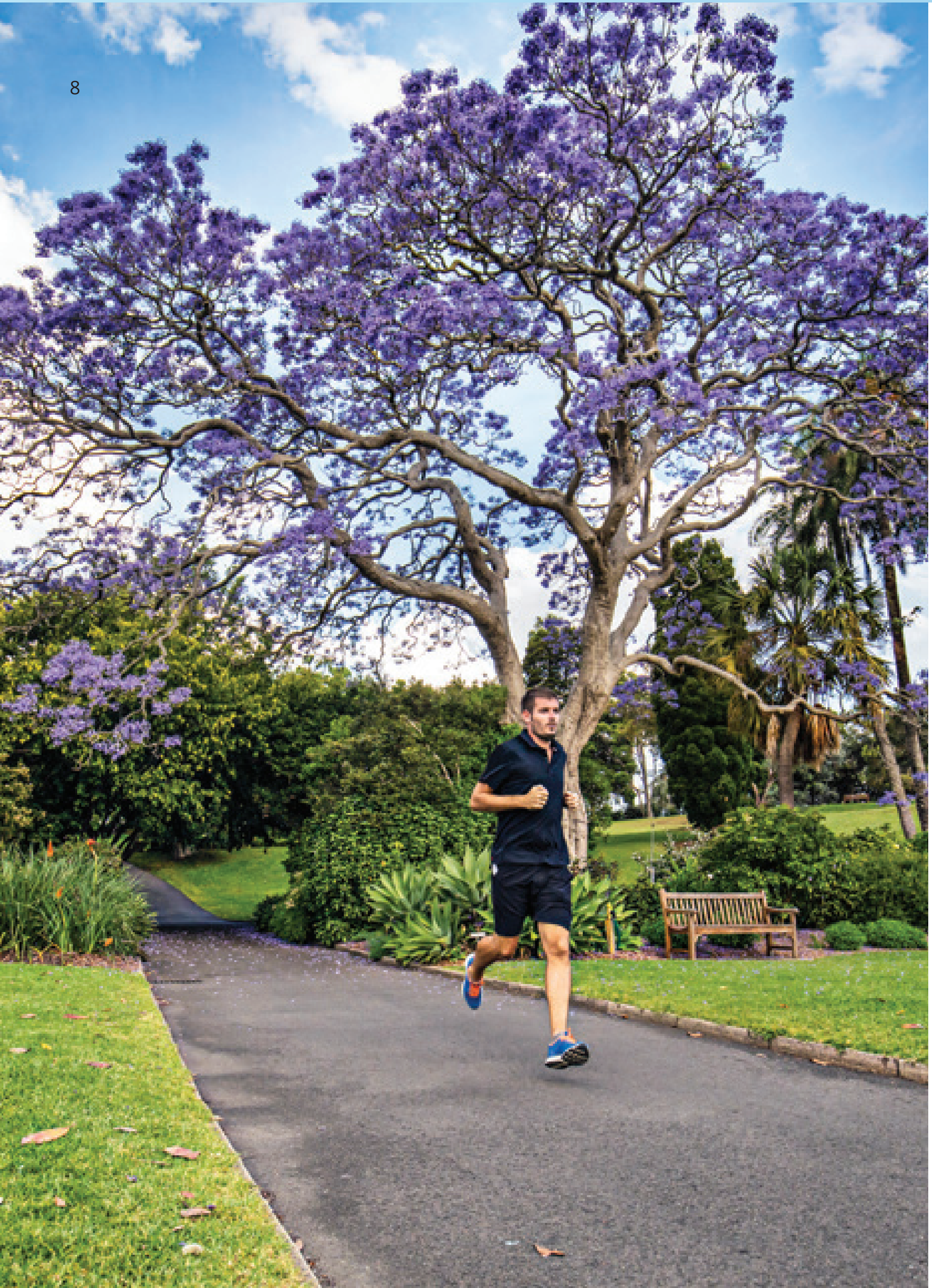

Parramatta City Council

Bushwalks

Parramatta abounds with bushwalks and 15 popular walking tracks found in Parramatta and surrounding suburbs are featured here. You are provided with a description of the track, distance, time to walk, vegetation and even the history of the area. These are outlined in the following link:

https://www.cityofparramatta.nsw.gov.au/environment/city-in-nature/bushland-and-biodiversity/bushwalks-of-parramatta

There are also brochures available online for walking trails which provide maps, walks, history of the area and difficulty. There is also one for the Great West Walk.

Parks and Recreation

This section gives a directory of each park and reserve in the LGA. It provides a wealth of information to help you choose the perfect destination. As well as giving you the location and directions on how to get to the park, there are great details on the amenities, barbeque facilities, circuits, cricket pitches, water, toilets, equipment, whether it's dog friendly and the availability of sports fields, play spaces as well as equipment and cubbies. This directory can be found at:

https://www.cityofparramatta.nsw.gov.au/parks-and-recreation

Skate Parks

These can be found at:
https://www.cityofparramatta.nsw.gov.au/recreation/our-parks/skate-parks

Water parks

Can be found at:
https://www.cityofparramatta.nsw.gov.au/recreation/our-parks/water-parks

Outdoor swimming and boating at Lake Parramatta Reserve

This lovely spot is just north of Parramatta. It is a 73ha area with picnic areas, walking trails and plenty of scope for water activities including swimming, canoeing and paddle boating. You can even hire a boat. The lake itself is 10.5 hectares in size and was officially re-opened for public swimming and water recreation activities in January 2015. There are lifeguards there every weekend and every day during the summer school holidays.

Visit the Parramatta City Council website: https://www.cityofparramatta.nsw.gov.au/

Health and Fitness Programs

https://www.cityofparramatta.nsw.gov.au/recreation/active-parramatta
https://www.cityofparramatta.nsw.gov.au/community/over-55s-activities-ageing-wellness/over-55s-leisure-and-learning/term-based-activities. Both these initiatives offer a range of opportunities for residents, workers and visitors to improve their health and wellbeing, and encourage active participation.

Great West Walk

An inspiring example of collaboration between councils, government and the community has resulted in the Great West Walk.

The Great West Walk is a 65km walking route stretching from Parramatta, crossing large areas of western Sydney and ending up at the foot of the Blue Mountains. Officially opened in October 2019, the walk traverses a varied landscape of protected Cumberland Plain woodland, local river systems, public parklands, Australia's oldest architecture and some of Western Sydney's most iconic urban landscapes.

The Great West Walk was mapped by The Walking Volunteers and delivered by local landholders including Blacktown City Council, City of Parramatta Council, Penrith City Council, NSW National Parks and Wildlife Service, and the Western Sydney Parklands Trust.

This website will give you very useful details on walk highlights along with FAQs and a map. There are also track notes to help you use and enjoy the walk providing details on public transport connections, vehicle access, parking, food & drink, water and toilets. https://greatwestwalk.com.au/

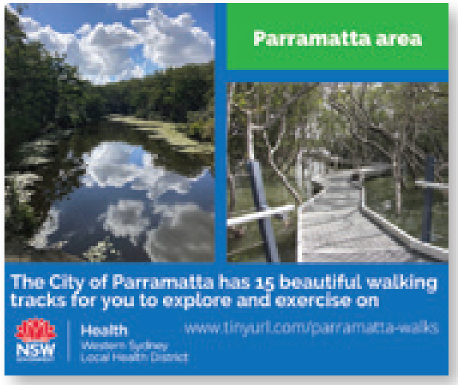

STATE FOREST

Cumberland State Forest

Australia's only metropolitan state forest, Cumberland State Forest, is at 95 Castle Hill Road, West Pennant Hills.

Cumberland State Forest contains 40 hectares of native forest. Enjoy a forest walk, picnic or barbecue, discover the surprising diversity of wildlife in the forest on a guided walk as part of an extensive school holiday and activities program, or participate in a volunteer program. Cumberland State Forest also runs a forest school education program.

Normal forest gate opening times are 8.30am - 5.00pm, except during daylight saving times where they are open 8.30am - 6pm.

Please note: Camping, trail bike riding and 4WDing are not permitted in Cumberland State Forest. Visit the website: https://www.forestrycorporation.com.au/visit/forests/cumberland-state-forest

Treetops Adventure The Hills

You'll also find one of Sydney's most magical tree rope parks, Treetops Adventure The Hills. With nine different tree rope courses to choose from, family members of all ages can experience the fantastical serenity of this park.
Visit: https://treetopsadventure.com.au/location/the-hills

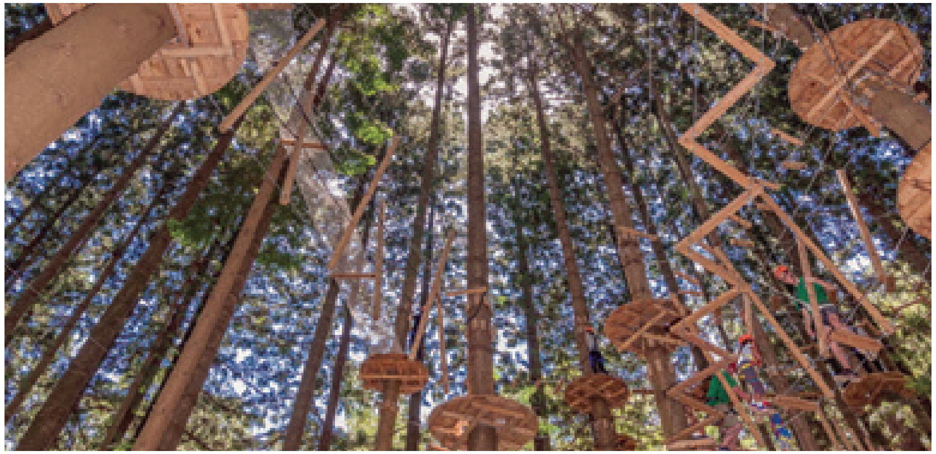

NATIONAL PARKS

Heading to a NSW national park? Be prepared with the NSW National Parks app.

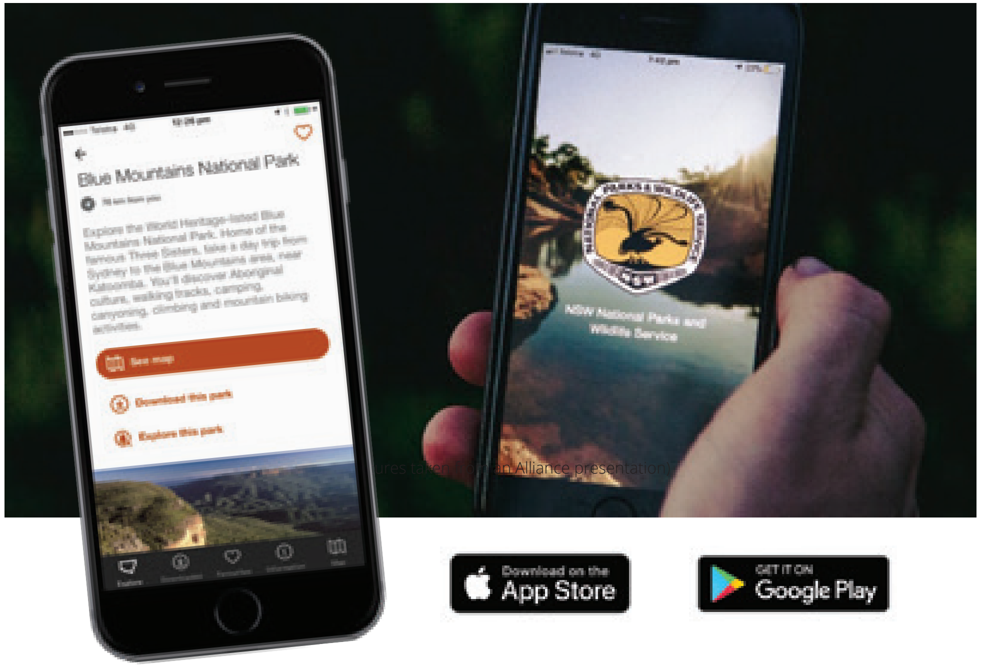

Download on the App Store

GET IT ON Google Play

With information on over 225 national parks and hundreds of things to see, do and places to stay, it's the ultimate NSW parks guide for your phone.

The NSW National Parks app is free to download on iOS and Android and offers an alternative to the traditional park brochure or map. Download information on selected parks when you're at home, then you can access park maps, guides and important info when you're in a national park and may not have internet access or mobile reception.

You're never out of range with the NSW National Parks app.

Key app features:

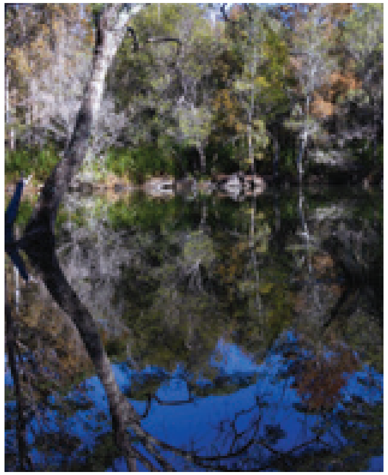

Downloadable maps

- Download a park before you go, then navigational GPS functionality allows you to view information and maps even without internet access.
- Use the map to locate parks, trails, things to do and places to stay.

Discover things to do

- Explore activities and attractions like a local
- Find things to do that are close to your current location
- Plan ahead using information about distances, accessibility and facilities

Find places to stay

- Locate campgrounds, caravan sites, cabins, homesteads and cottages in national parks
- Book national park accommodation (internet access required)

Find parks nearby

- Use your location to find the national parks closest to you
- Find parks all over NSW using the map or the search tool

Save your favourites for quick access later

- Favourite parks, things to do and places to stay to quickly access them later via your 'Favourites' tab.

Here are three national parks on our doorstep – all easily accessible for a daytrip.

https://www.nationalparks.nsw.gov.au/visit-a-park/parks/rouse-hill-regional-park
https://www.nationalparks.nsw.gov.au/visit-a-park/parks/scheyville-national-park
https://www.nationalparks.nsw.gov.au/visit-a-park/parks/blue-mountains-national-park

For general inquiries call the National Parks Contact Centre, 7am-7pm daily on **1300 072 757 (13000 PARKS)** for the cost of a local call within Australia (excluding mobiles): parks.info@environment.nsw.gov.au

The National Parks office is at Memorial Drive, Scheyville, **(02) 4580 2750** or visit: npws.cumberland@environment.nsw.gov.au

Dharug National Park

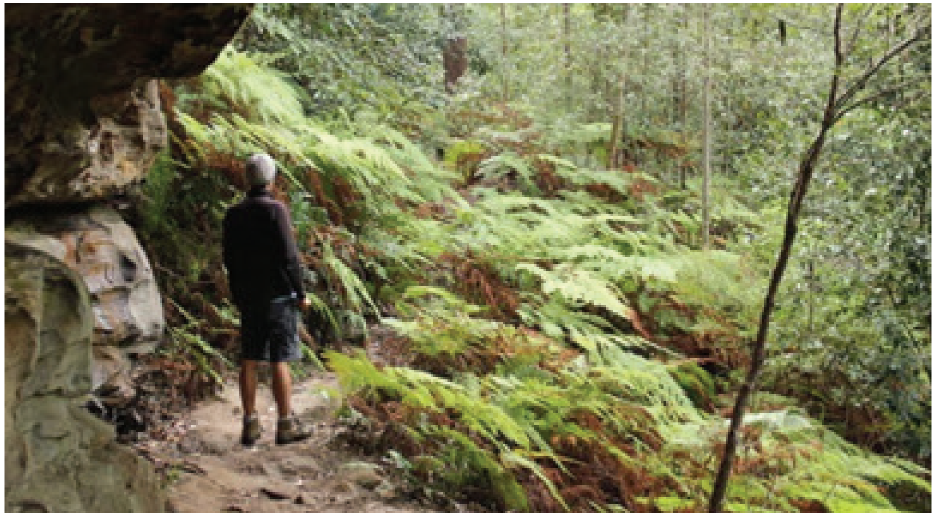

Explore the World Heritage-listed Old Great North Road, go camping, mountain biking or canoeing at Dharug National Park near Wisemans Ferry, northwest of Sydney.

Dharug National Park covers rugged bushland just north of the Hawkesbury River, with dramatic sandstone cliffs and a variety of natural habitats. It's the perfect place to escape to on a daytrip or for a weekend getaway.

There is loads to explore; take a walk, mountain bike or guided tour into history along the Old Great North Road – World Heritage walk; built by up to 720 convicts between 1826 and 1836. Download the Convict Road app before heading out to accompany you along the track. Take a canoe or kayak out to explore the Hawkesbury River or camp out under the stars at Mill Creek or Ten Mile Hollow campground.

The park is bursting with bird and animal life. You're likely to see gang-gang cockatoos, satin bowerbirds and green catbirds on the mountain slopes and, if you're camping overnight, take a torch to look for yellow-bellied and greater gliders and sugar gliders.

Check park alerts and visit COVID-19 updates for information before visiting any park.

Details: **Wisemans Ferry Road, Gunderman**:
https://www.nationalparks.nsw.gov.au/visit-a-park/parks/dharug-national-park

Wisemans Ferry

Wisemans Ferry is about 75km north-west of Sydney's CBD and is at the tip of western Sydney. Surrounded by beautiful national parks and a village that takes you back in time, Wisemans Ferry is home to one of Australia's most famous waterways, the Hawkesbury River.

The wide riverbanks, miles of skiable water and the most gorgeous backdrop of rolling green hills make Wisemans Ferry a must-visit water sports destination.

The park is also a great destination for mountain biking and camping, so make sure you bring all of the gear for an awesome summer river adventure.

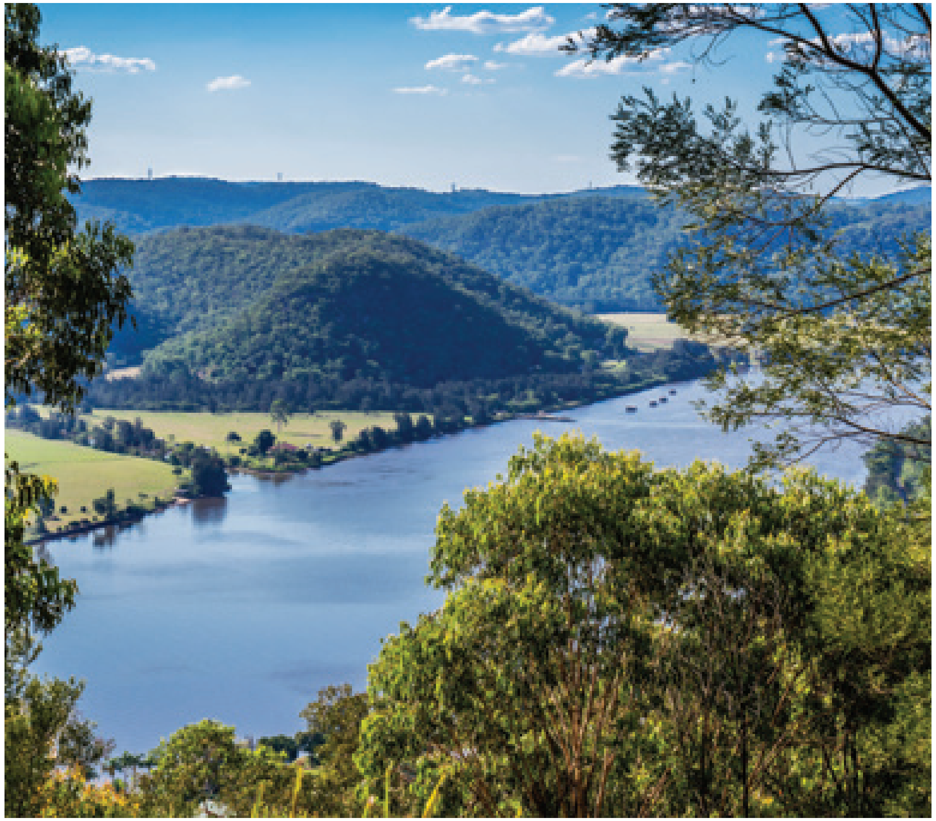

OUTDOORS NSW & ACT

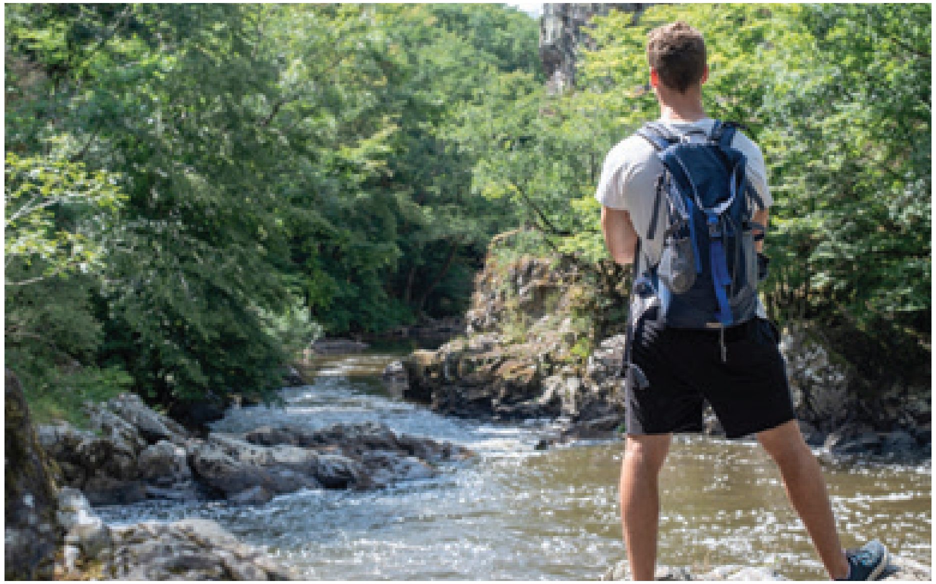

This organisation supports the people and organisations dedicated to facilitating outdoor experiences for the NSW & ACT communities.

They actively encourage participation in 4 outdoor pillars – Outdoor Recreation, Education, Nature/Adventure Tourism and Therapy – for the benefit of the individual, community, society and planet.

Outdoors NSW & ACT aims to assist its members who are working in outdoor recreation, outdoor education, nature therapy and adventure tourism services. These are made up of individuals and organisations such as practitioners, providers, retailers and active recreation clubs/ associations.

If you are working in the outdoor industry this organisation will have all the information to assist you learn, connect and promote your organisation.

If you live in western Sydney and would like to be more involved in the Outdoors, contact Outdoors NSW & ACT and they can link you up with one or more of their many member organisations. This can be for a range of activities as varied as organising a school camp, to becoming involved in forest bathing. Outdoors NSW & ACT is sure to have a member organisation who can help you get out and enjoy the great outdoors.

Outdoors NSW & ACT
PO Box 222, Galston NSW 2159
info@outdoorsnswact.com.au
Phone: 0407 411 566
Visit the website: https://www.outdoorsnswact.org

Parramatta Park

163-year-old Parramatta Park is known as “a park for the people” – a fantastic place to picnic, celebrate family milestones, get active and explore our unique history.

Walking and Cycling

Parramatta Park is a beautiful setting for walking and cycling. With its 3.2km loop cycle lane and shared paths, it is suitable for cyclist of all levels and it connects to cycle routes travelling along the Parramatta River, all the way to Sydney Olympic Park.

Walking and cycling are the ideal choice for exploring the historically-significant sites in and around the park, whether you’re following one of the self-guided tours or retracing the steps of the traditional Burramattagal people along the Parramatta River.

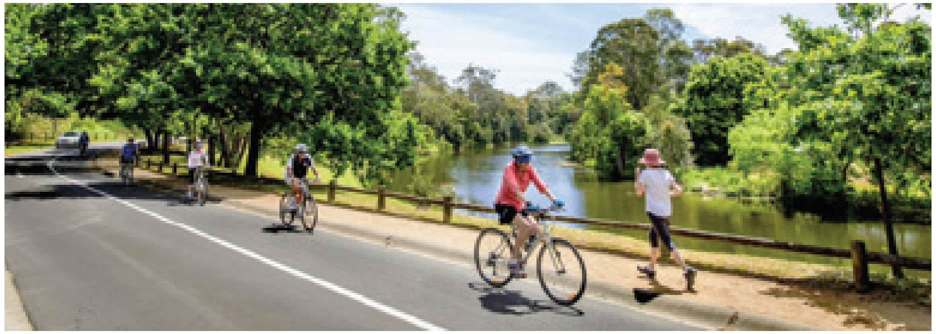

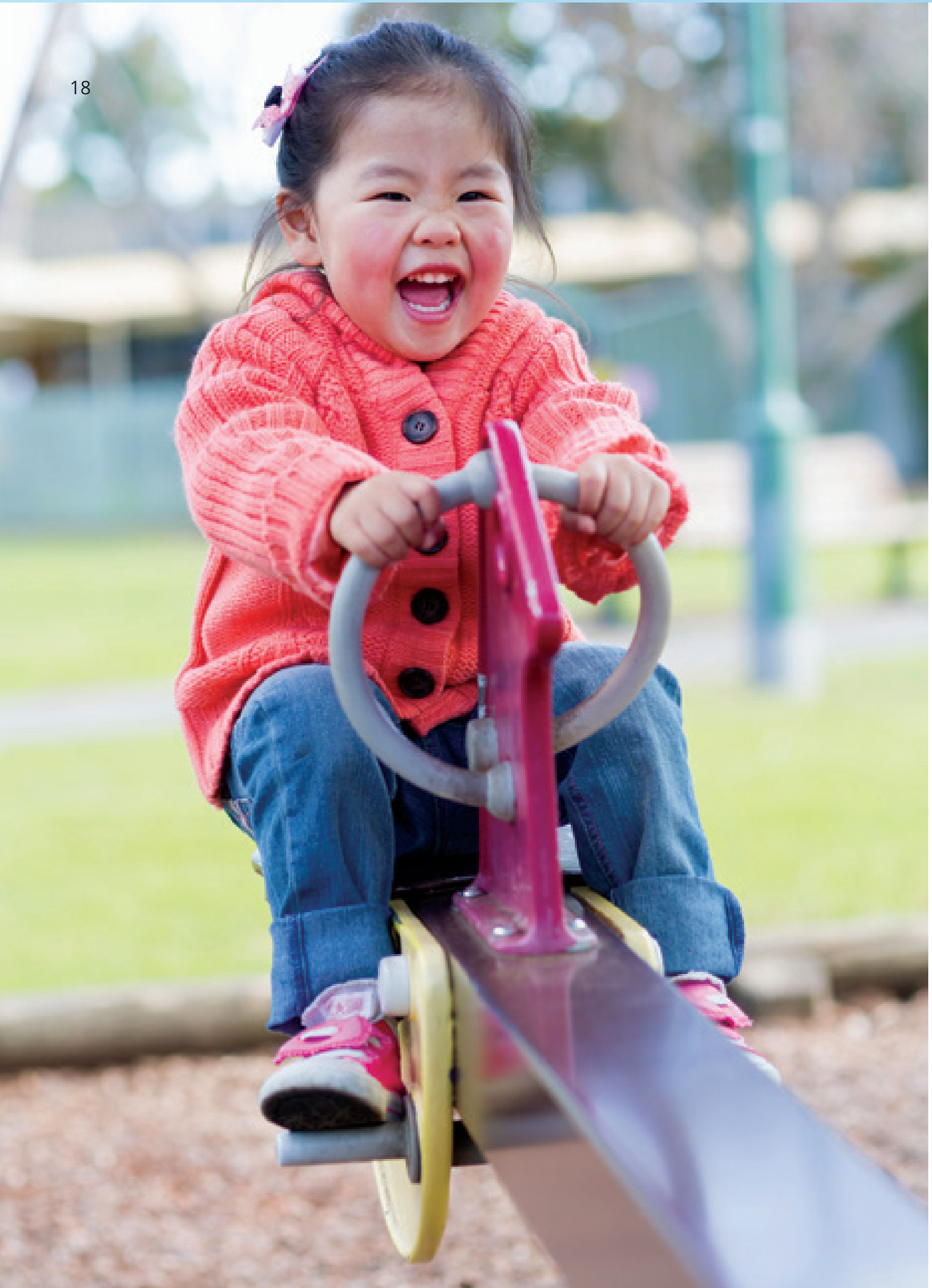

Picnicking

Parramatta Park is the perfect place to gather with family and friends for a picnic. With plenty of green open space, you can choose a pretty spot anywhere in the park to lay down your picnic rug. There are also two fun playground areas to keep the children entertained – the inclusive Paperbark Playground and Domain Creek Playground.

Heritage Tours

Immerse yourself in history with Parramatta Park's heritage tours. Taking guided and self-guided tours of Parramatta Park is a great way to learn about its significance and gain insight into Australia's early colonial and First Nations history.
https://www.parrapark.com.au/

Robinhood Fitness Equipment

ROBINHOOD FREE OUTDOOR FITNESS

This website provides excellent information on local fitness facilities and the good news is that they are all free. The website provides information on outdoor gyms, pools and fitness activities including group fitness classes, dance and mind/body groups.

To find suitable facilities just type in your location and how far you are prepared to travel. You will be presented with a range of free fitness facilities. They include a list of what equipment is available at each venue, as well as maps and directions on how to get there.

Photos of the equipment are included as well as videos which provide exercise instructions on how to use the equipment. Visit the website:
https://freeoutdoorfitness.net

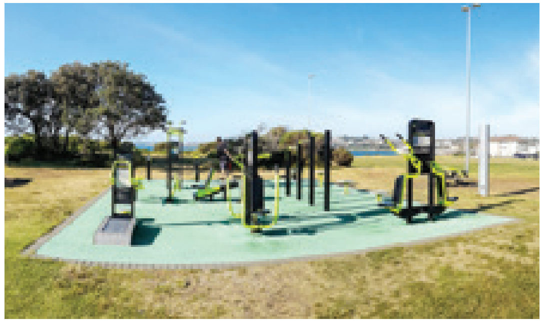

SYDNEY OLYMPIC PARK

https://www.sydneyolympicpark.com.au

Discover something new to see and do in Sydney Olympic Park. Enjoy a swim at the Aquatic Centre, a bike ride along the 35km of safe cycling pathways, a stroll through 430 hectares of parklands, a game of archery at the Archery Centre, plus so much more!

You can swim laps in the pool where Ian Thorpe won three Olympic gold and two silver medals. Check out the Dive into History exhibition at the Aquatic Centre, which celebrates the great stories and triumphs of Australia's swimming legends.

You can hire a bike at both Bicentennial Park and Newington Armoury to explore the cycle ways across the Parklands, one of Australia's largest urban parklands. To find a map of the cycle circuits, simply go to https://www.sydneyolympicpark.com.au/map

There are free barbecue facilities in the park as well as lakeside & riverside picnic areas, splashing fountains and water play. Other activities include Segway tours at Newington Armory. You can even try your hand at archery at the Sydney Olympic Park Archery Centre. There are a constantly new and changing community events, so check out the events calendar https://www.sydneyolympicpark.com.au/Whats-On for more.

BMX and Mountain X at Monster BMX Track

https://www.sydneyolympicpark.com.au/Things-to-Do/family-and-leisure/bmx-and-mountain-x

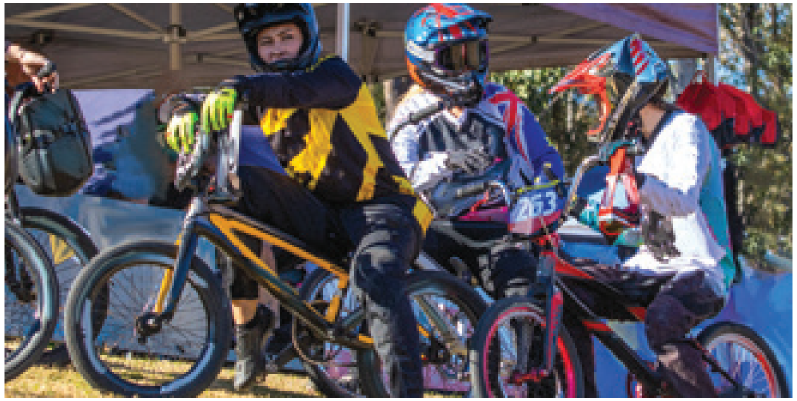

Love BMX and Mountain Bike riding? Test your off-road bike skills on 250 metres of track, with wicked downhills and flying dirt jumps!

The Monster BMX track is for freestylers and racers, from beginners to pros – with a world-class 4 X design. Bring your crew and battle it out on one of the best downhill tracks in Sydney. Best of all the track is free to use outside club event times!

Riders must use appropriate bikes and safety gear for BMX and Mountain X.

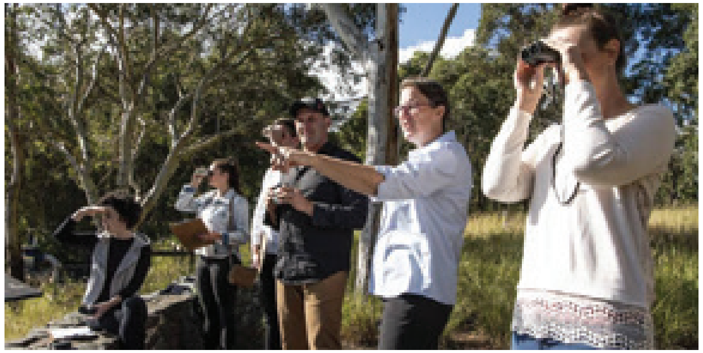

Self-guided tours

Discover the many stories and experiences at Sydney Olympic Park, by visiting them yourself or explore from wherever you might be. Whether you are visiting the Park, are a resident, or just curious to see what's there, use the self-guided tours app to discover the Park at your own pace and in your own space.

With 8 tours available there is something there for everyone. Tours currently include Sydney Olympic Park's Games Legacy Experience, Bicentennial Park and Surrounds, Newington Nature Reserve Circuit, Marvel in the Mangroves, Urban Art, Mangrove Magic Kids Sensory Tour, Eco-ramble (Lake Belvedere and Eco-ramble with Louise Sauvage).

You'll be able to discover the natural environments, species and more, that make up the 640 hectares of Sydney Olympic Park.

Keep the app updated to access new tours as they become available. Download from Apple iTunes and on Android from Google Play Store.

https://www.sydneyolympicpark.com.au/Things-to-Do/learning-and-discovery/self-guided-tour-app

Toilet Finder

https://toiletmap.gov.au

You may like to look at this website before you head out and about. This website outlines what facilities are available throughout the community including parks and outdoor areas. It is also available as an app: https://www.health.gov.au/resources/apps-and-tools/national-public-toilet-map-app

WESTERN SYDNEY PARKLANDS

Western Sydney Parklands is a place for people of all backgrounds to meet, celebrate, learn, play and appreciate the environment.

Set in the heart of Western Sydney, Western Sydney Parklands is comprised of 5,280 hectares, stretching 27 kilometres from Blacktown in the north to Leppington in the south.

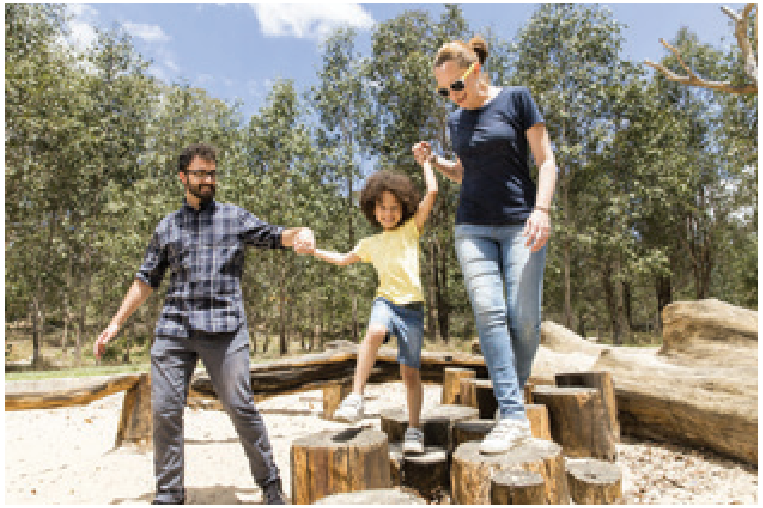

Walking & Cycling

Get back on track. Get off the couch and get back into nature in Sydney's Biggest Backyard!

Western Sydney Parklands is home to more than 60km of tracks and trails. Come and explore them and get back to what really matters – nature, family, friends, and fun. Tracks and trails across Western Sydney Parklands are perfect for walking and running, and some are suitable for cycling.

A walk can take anywhere from 30 minutes to three hours to complete. Most tracks are paved or gravelled, but some are grass tracks, fire trails or old farm roads. Whether you want a leisurely stroll, quick jog after work, or to train for an epic trek or marathon, they've got a route for everyone. Check out Western Sydney Parklands' digital maps to find the right trail for you.

Dog walking

Dogs are welcome anywhere in the Parklands, but they must be on a leash when not in designated off-leash areas. These dog parks are located at Warrigal Run, Bungarribee in Doonside (Sydney's largest off-leash area) or Shale Hills Dog Park at West Hoxton.

Treetops Adventure

Get up-close and personal with the natural wonders of the Parklands, as you climb, weave and fly through the treetops on self-guided rope courses. Enjoy an adventure high in the treetops, complete with flying foxes and suspension bridges. Choose the Treetops Adventure course for kids aged 3 and over, or try the Treetops Zipcoaster Express, the fastest rollercoaster zipline in the world! Training and safety equipment are provided, just bring your sense of adventure.

Bird watching

Western Sydney Parklands is home to many birds including native cockatoos, lorikeets, rosellas, parrots and more. Birdwatching enthusiasts should head to Sugarloaf Ridge and Glendenning and explore the Parklands track through Plough and Harrow for the best views of the wildlife. Download a copy of the 'Photo guide to the birds of Western Sydney' to help you on your birdwatching.

BMX & Mountain Bike Riding

Wylde is a purpose-built facility located off Elizabeth Drive in Cecil Park, with a range of sections suitable for any rider – from beginner to expert!

These include a jump run, pump track and series of loops. Before you go for a ride, always check the open status on the website or on the Wylde Facebook group under 'Announcements'.

First-time visitors should take extra precautions until they are familiar with the Trail. Those under 16 years of age must ride the Trail in the company of an adult.

Calmsley Hill City Farm

This is a place for children and adults to enjoy a variety of shows and hands-on exhibits, with an insight into the workings of a farm.

Get up-close and personal with the native and farmyard animals – you'll be able to feed and pet nursery animals, milk cows and even walk into the kangaroo wildlife sanctuary as well as watch the shows and demonstrations.

Experience the best of Rural Australia in Sydney's backyard

Picnicking

Western Sydney Parklands has no shortage of beautiful picnic areas with barbecues, shelters and amenities, as well as some of Sydney's most exciting playgrounds. Bungarribee is a 200-hectare recreational park with 13 picnic shelters and 20 barbecues. With its wide-open space, Bungarribee is fantastic for walking and cycling and letting the dog play off-leash at the Warrigal Dog Run. Children go wild for the Bungarribee playground, which boasts a 14m-high climbing tower, slides, a 50m-long flying fox, swings and water play area.

Surrounded by beautiful native bushland, Plough and Harrow is split into two areas. The East side is action-packed, while the West side is designed for quieter gatherings. Both Plough and Harrow East and Plough and Harrow West have picnic shelters for hire.

The playground at Plough and Harrow East has a water play area and lots of climbing equipment, plus all sorts of weird and wonderful play equipment. Favourites include the flying fox, a giant hamster wheel, and 5-sided swing set. The natural wonderland at Lizard Log is a perfect place for a picnic. It has several shelters, electric barbecues and purpose-built coal barbecuing stations and is surrounded by beautiful bushland and grasslands: westernsydneyparklands.com.au

Raging Waters Sydney

Raging Waters Sydney is the place to make your summer memories! Splash and slide into the fun at Sydney's biggest water park, offering over 30 rides, slides and attractions.

There is something for everyone from the splash pads for the little water adventurers, to the adrenaline-pumping slides for the thrill seekers.

Challenge your family and friends on the H2Go Racers, experience the thrills on the 360 Rush, ride the wave of Bombora, chill out by the beach or laze around the Dinosaur Lagoon: ragingwaterssydney.com.au

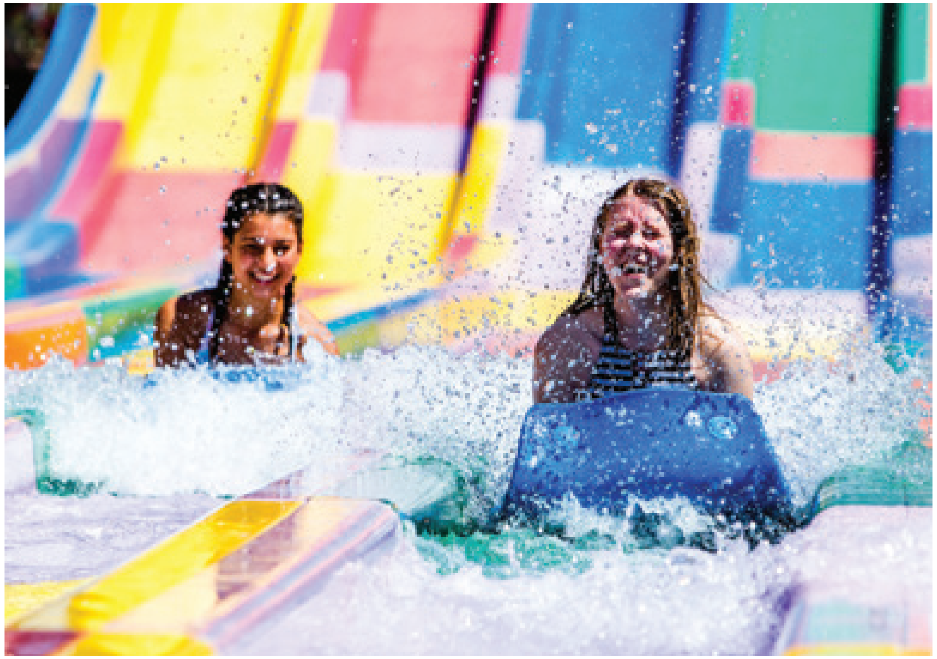

Courtesy of Raging Waters

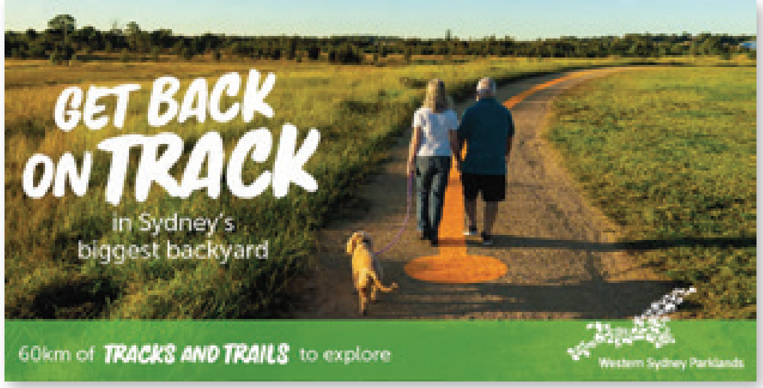

Wildwalks

Wildwalks is an online Bushwalking Guide Book with the aim of helping more people get out and safely enjoy the many great bushwalks and camping opportunities in NSW. It does not matter if you are new to bushwalking or experienced, there is something here for everyone.

WildWalks

Wildwalks is developed by a small group of people who are passionate about the outdoors. We want to encourage people to “get out there” and enjoy the great places on offer.

This is a very informative website for anyone interested in exploring outdoors in western Sydney and beyond. It describes parks and walking facilities in regions throughout NSW, along with providing information on the best walks for overnight, experienced walkers and even those taking children along. It breaks up walks by lengths and also grades them from very easy, to those suitable for the very experienced.

‘Universally accessible’ walks can be found here http://www.wildwalks.com/lists/very+easy

You are also given a tool where you can enter a postcode and be provided with a list of local walks. You can also download a list of campsites with descriptions, along with registration forms for the National Parks and Crown Lands. This website also provides useful information on bushwalking and topographical maps.

http://www.wildwalks.com/

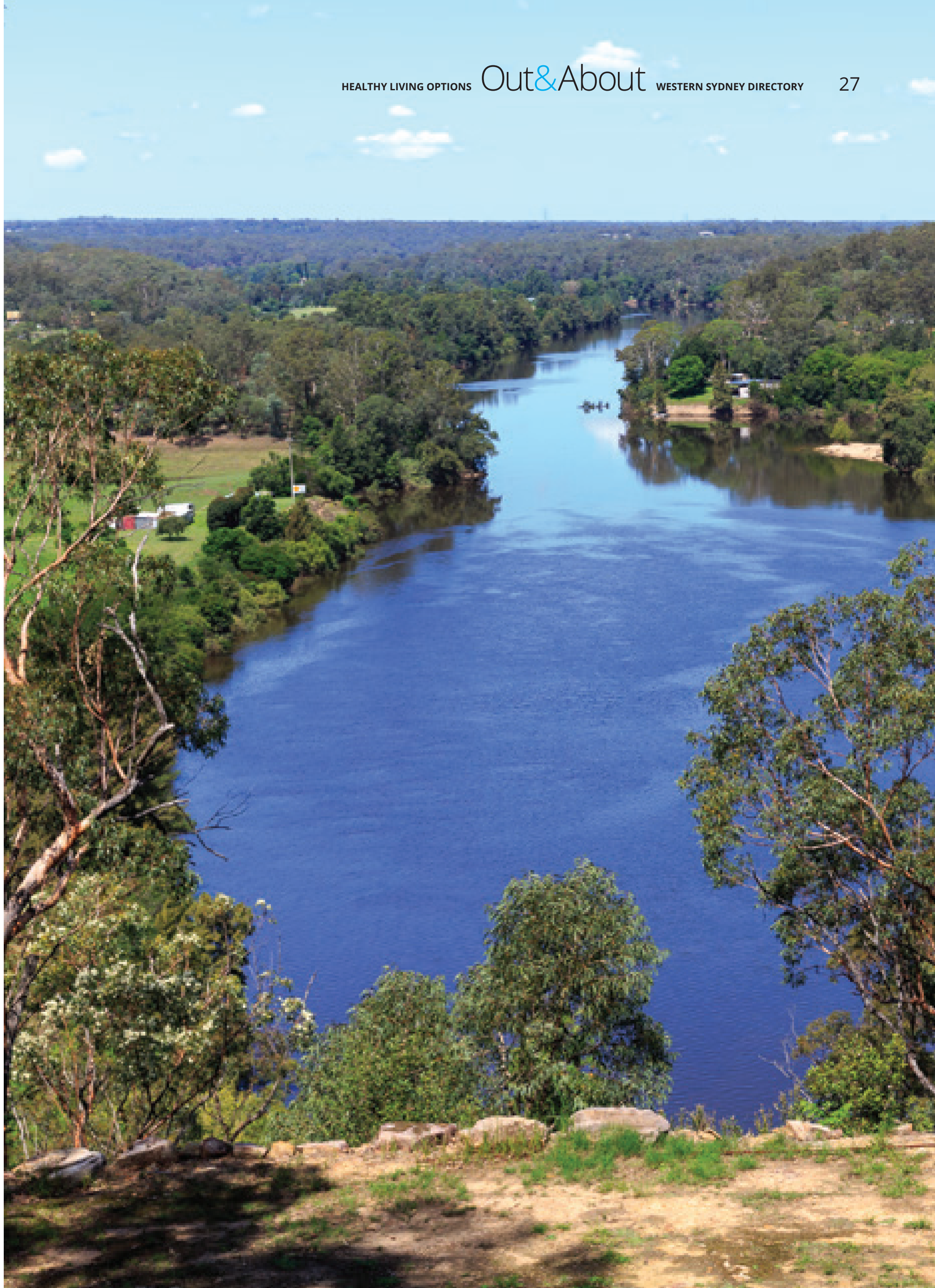

Section 2

LEARN OUTDOOR SKILLS

Bike riding

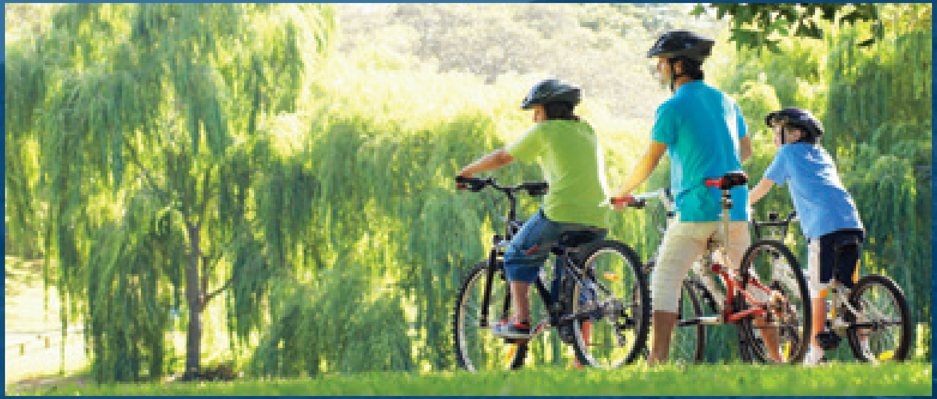

Kids on Bikes

Kids on Bikes is a ride school for all ages and skill levels. The trained coaches can make riding fun. Private or Group coaching classes are available to meet your needs. You can go from 'zero to hero' in no time at all. Whether you are looking to ride for pleasure, develop ride competency, ride to school or race competitively, they can help. No one is ever too young or too old to enjoy the simple pleasure of riding a bike. Cycling is great for fitness, health, transport, recreation and sport. Kids on Bikes can get you riding and having fun on your bike.

The coaching classes are available seven days a week. All learn to ride lessons are 1 hour in length and they can supply the bikes. You just need to bring your helmet if you own one.

Although they are based near Sydney Olympic Park the instructors are happy to deliver classes within the wider Sydney region.

They also cater for 'big kids' who'd like to learn to ride or just improve their cycling skills.

Classes are run one on one, at your own pace in a safe open environment that is both informative and enjoyable. The trained professional coaches have years of experience to support you from learning to ride, through to achieving your cycling related goals.

Call for more information on the classes:

- Learn to Ride
- Cycle Proficiency
- Women's Specific Classes
- Shared Pathways
- Group Rides
- Commuting & Traffic Skills
- Fitness & Performance Coaching

Details: Gay Watson, **0403 147 166;** Ian Watson: **0403 203 253**;
email: 2learn2ride@gmail.com or website: https://kidsonbikes.com.au

Ride Nation

Ride Nation is a place the bike riding community can come to connect, learn, improve and have fun. Wherever you are on your bike riding journey, Ride Nation houses programs, activities, events, coaches, and adventures that suit your needs. This website allows you to find programs and services for bike riding including learning to ride a bike, bike safety, bike maintenance and having a try at BMX, Track or Road or Mountain Biking.
https://www.ridenation.com.au

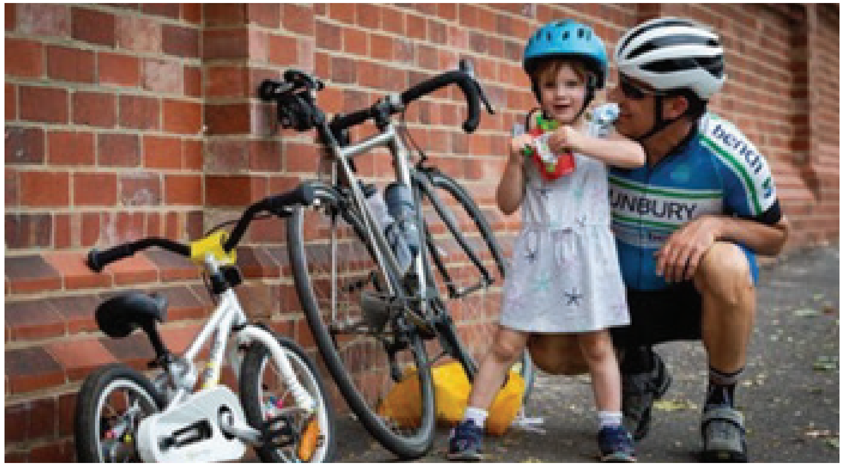

BUSHCRAFT/CAMPING SKILLS

Bushcraft Survival Australia

Bushcraft Survival Australia (BSA) is an outdoor bushcraft survival school which is dedicated to teaching authentic traditional and modern outdoor living skills through carefully designed educational courses. Their qualified instructors are properly trained in both military survival and in bushcraft, holding qualifications from some of the top survival and bushcraft schools worldwide.

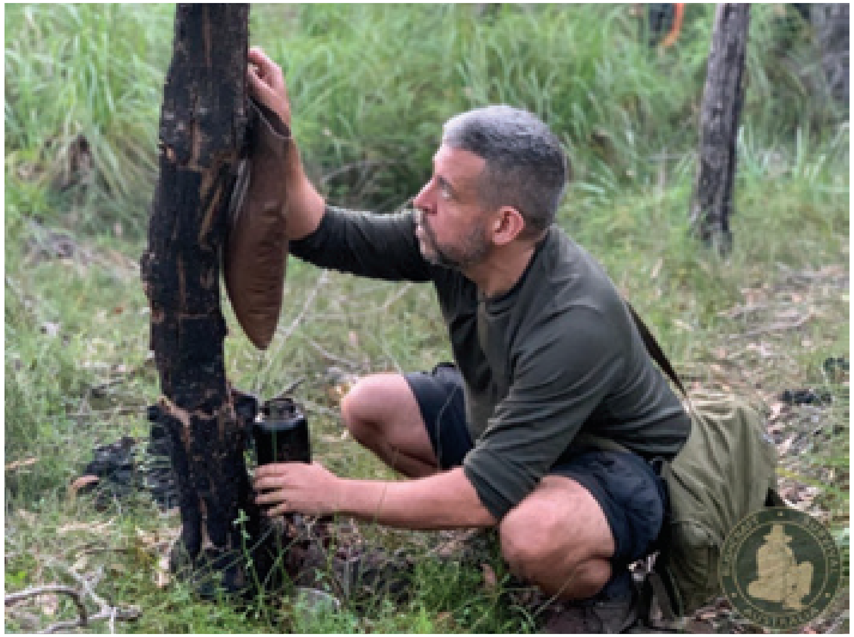

Their courses and workshops for both adults and children are designed to span a range of specialist knowledge and skill sets, from the priorities of basic survival, foraging for water and food, fire and shelter construction, right through to natural navigation, traditional cooking methods and other traditional skills.

This is done while always respecting and learning from the insights provided from traditional wisdom.

They cater to suit all levels of experience: from the novice to the experienced outdoors-man, as well as campers, fisherman, hikers, travellers, bushcraft and survival enthusiasts, as well as the young and the old. In fact, anyone with a passion for the outdoors and the natural world.

https://bushcraftsurvivalaustralia.com.au

Girl Guides

Girl Guides is Australia's leading volunteer organisation for girls and young women in leadership and personal development. Our mission is to empower girls and young women to discover their potential as leaders of their world through informal education and self-selected new experiences and challenges in creative pursuits, the great outdoors and making a contribution to their community.

There are a large number of Girl Guide units in Western Sydney for girls aged 5 to 17 years. The Australian Guide Program is facilitated by trained volunteer Leaders with a focus on programs being girl-led. This means that all the activities the girls do, from weekly Unit meetings, to camps, to expeditions and hikes, are driven by the girls themselves with input and assistance from their Leaders. Activities are also planned and designed to match the girls' abilities. Girls will join a Unit within their area, and with the guidance of a trained adult volunteer Leader, will discover, decide, plan and participate in a wide range of activities that reflect her interests.

Girl Guides have a range of opportunities for women over the age of 18 years including the opportunity to become a Volunteer Unit Leader. As a volunteer you will be involved in developing and delivering fun and creative activities for girls in a safe environment on a weekly basis. You will receive training and support continuously throughout your volunteer journey and become part of a global community of over 10 million women and girls – all while making a real difference in your community and the lives of young women and girls.

To find out more visit the Girl Guides NSW, ACT & NT website:
https://www.girlguides-nswactnt.org.au
Phone **1300 447 548**, Email: guides@girlguides-nswactnt.org.au

Scouts

Scouts Australia provides young people aged 5 to 25 with fun and challenging opportunities to grow through adventure. With around 70,000 members, Scouts Australia is one of the largest youth development organisations in Australia.

Today's Scouts can take part in an extraordinary variety of outdoor activities, from 'traditional Scouting skills' such as camping and bushcraft, through to more extreme challenges such as abseiling, overnight hiking, rafting, canoeing, canyoning, snow activities, rock climbing, sailing… even flying!

From starting out as a Joey Scout, you will learn how to recognise native birds and animals along with developing an understanding of the impact of our modern world on the environment.

You'll move on to going on camps with your Pack Group, and you might attend a Cuboree camp with hundreds of other Cub Scouts! As well as learning things like how to use a compass, you'll go on epic bushwalks and camping expeditions, get out on the water in a kayak, canoe or sailboat or even going up in a plane on a visit to an Air Activity Centre. These are just some of the adventures Scouts get up to!

As a Venturer Scout, you may find yourself exploring wilderness Australia, camping above the snowline or diving on coral reefs. You could be abseiling into a limestone cave or climbing a sheer rock face. Perhaps you'll learn white water kayaking or ride your mountain bike on an expedition across real mountains.

With over 30 groups in western Sydney, you're sure to find a group near you. Check out the website for details. https://nsw.scouts.com.au or call the Greater Western Sydney region office on **(02) 9639 2488**.

Want to get involved as a volunteer? Being part of Scouts as an adult is a very rewarding and fun volunteer activity. Whether you are a young person wanting to contribute to your local community or a parent wanting a fulfilling activity to enjoy with your children, Scouts has a range of volunteer opportunities from adult helper through to leadership roles. Leaders can access accredited training programs on a wide range of outdoor activities that develop skills. Volunteering with Scouts offers adults the chance to give back but also develop a wide range of life skills. Find out more here: Leaders | Adult Leader | Volunteering | Scouts NSW (joinscouts.com.au)

YMCA camps

These camps give you the opportunity to work with them to plan and develop programs that are specifically designed and facilitated for your group, based on your desired learning outcomes.

Features of a YMCA Outdoor Education & Camping Program:

- YMCA Staff role model positive, responsible behaviour
- All Y staff understand the positive impact Outdoor Education & Camping experiences can have on individuals and teams and work with the intent to provide the best learning experience possible for all participants
- Young people demonstrate positive values by caring for and encouraging each other
- Young people gain independence by taking responsibility for themselves
- Programs at the YMCA are a friendly, inclusive place where all participants are welcome and diversity is embraced
- Programs are places that spark imagination and awaken a sense of adventure, a place of fun where outdoor play is always encouraged.

The Outdoor Education & Camping values form the foundation of the community. Participants are introduced to these values at the beginning of a program and they guide the way to interact with each other during the time on the program. If you have a group of 20 or more who would like to learn more about participating in the great outdoors, contact the relevant camp organiser.

(02) 9737 8139 – Sydney Olympic Park Lodge
(02) 4776 1037 – Camp Yarramundi

Outdoor Education & Camping Learning Framework
https://www.ymcansw.org.au/camping/outdoor-ed-learning-framework

PHYSICAL SKILLS

Can Too Foundation

Can Too will help you achieve something amazing this year!

Can Too is a health promotion charity who offer professionally coached training programs for all levels, from beginners to more experienced athletes, in structured 6-20 week training programs tailored to specific physical challenges. This year, you could achieve a 10km run, Half Marathon, Marathon, Ocean Swim, Trail Walk or Adventure Challenge. You can even choose your own event!

In return for professionally coached training programs, our participants fundraise much-needed support for Australian cancer research to fund the most brilliant, innovative early-career cancer researchers.

At Can Too, we believe everyone can achieve goals they once thought impossible. We believe life is what you make of it, that anything is possible, and if you're willing to take that first step, you CAN TOO!

Take charge of your lifestyle. Reduce your risk of cancer. Fundraise for research. Join us now: https://www.cantoo.org.au/home

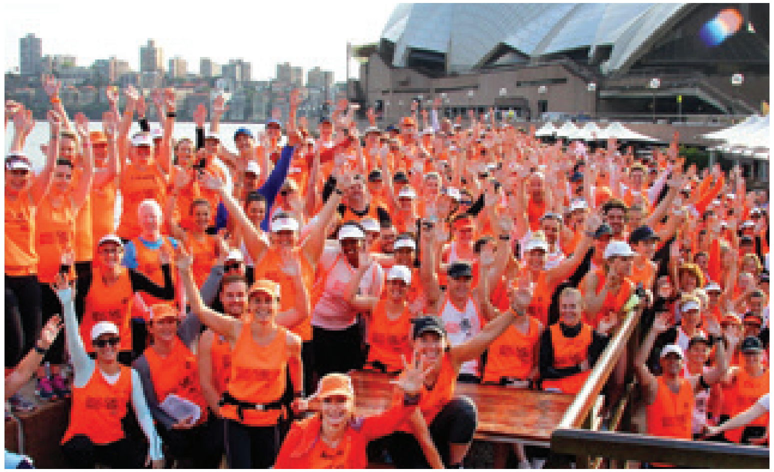

LAND-BASED SKILLS

Golf

Golf Australia

Keen to learn to play golf? Just use this website. You will need to enter your postcode and the distance you're prepared to travel. You will then be provided with the details of all the golf clubs (both private and public) within your specified radius. You can find out how you can arrange to have a game and also learn how to go about organising lessons.

You can learn the basics of golf at Get Into Golf, the program is fun and social.

There is an introductory program to golf for children called MyGolf which is available to teach kids the skills of golf. Once again, you can find local participating clubs through this useful website: https://www.golf.org.au/ and https://www.golf.org.au/courseSearch

Find a golf program near you for adults: https://www.golf.org.au/getintogolf/home
Find a golf program near you for kids: https://www.golf.org.au/mygolf/home/

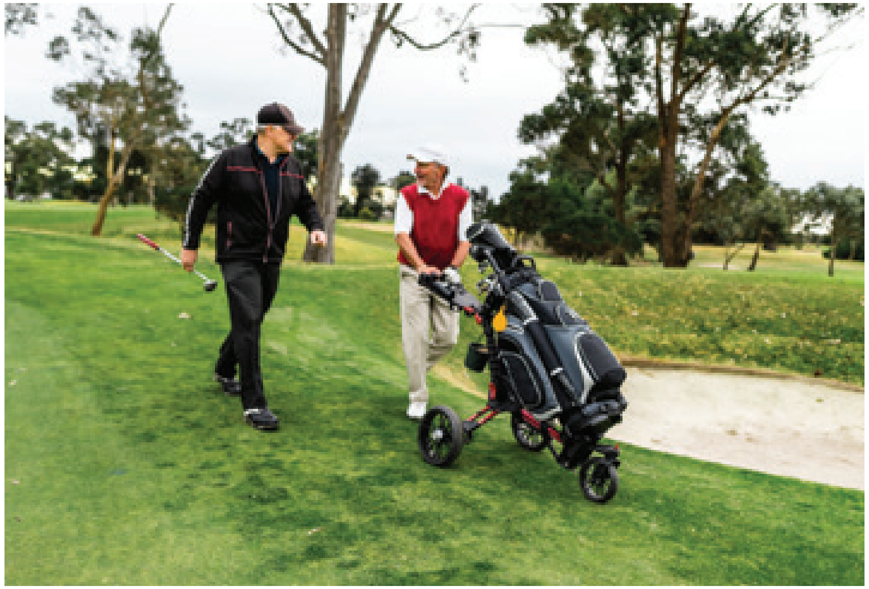

Horseriding

Calm Wood Equestrian Centre

Riding lessons with EA qualified, caring coaches in a fun, safe environment.

Calm Wood caters for nervous beginners right through to competitive riders. Weekend clinics throughout the year with visiting coaches. Pony rides for the tiny tots.

Whether you want to learn basic riding skills, dressage, hacking or show jumping, or need help preparing for competition, we are here to help you. We have quiet school horses available for use, or students are welcome to bring their horse or pony for lessons.

CalmWood operates under a COVID safe policy.

Details: 102 Goods Road Oakville, NSW, **0422 042 477.**
horsesatoakville@hotmail.com

Sydney Hills Horse Riding Centre

66 Kenthurst Rd Dural 0421 218 983 Carolyn Stubbs
Sydney Horse Riding Centre focuses on riders feeling safe and comfortable while increasing confidence and abilities. They have well trained horses and ponies for all levels and ages.

They provide a comprehensive learning and riding experience with a number of different lessons and programs available.

The lessons program includes group and private horse riding lessons, as well as a mother's group (after school drop off time). Three day school holiday camps are available.

The horses are matched with the age and ability of the rider. The experienced and qualified coaches will provide you with the skills and knowledge to start you on a horse riding journey that can last a lifetime!

The coaches provide instruction so the students learn to care for, handle and ride a horse and hopefully have a lot of fun along the way.
https://sydneyhillshorseriding.com

Walking

This website gives some outstanding tips, links and ideas for getting out and about, and walking in western Sydney. It also provides a number of links to council and other walking organisations.
https://www.wslhd.health.nsw.gov.au/Healthy-Children/Families/Information-for-a-healthy-family/walking-in-western-sydney

WATER SKILLS

Swimming

Learning to swim is a lifelong skill which will allow you to safely enjoy so many of the outdoor opportunities available in western Sydney.

However, many residents in western Sydney did not have the opportunity to learn to swim as children. The good news is that it's never too late to learn. The swim schools in western Sydney have classes specifically tailored to any level of skills and experience. Whether it's your first time swimming lesson, or if you want to improve your swimming skills, there will be a group for you.

Swimming classes for children and even babies are readily available at all the swim schools and public swimming pools in western Sydney.

All centres have professional and fully accredited swim instructors who can teach and encourage you at your own pace, whatever your age. The instructors and coaches undertake rigorous training in the teaching of specialist skills such as stroke development and are qualified to teach groups with different needs including infants, pre-schoolers, adults and people with disabilities.

As a guide, most group classes run for 30-45 minutes and cost between $17-$25 per session. Private lessons are also available at most venues. Active Kids vouchers are also accepted for children's lessons.

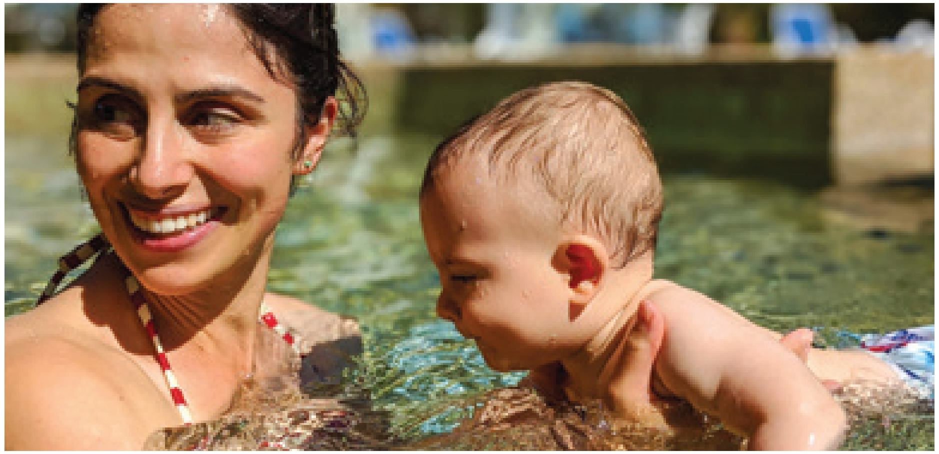

Learn to Swim venues in Western Sydney

Blacktown
Blacktown Aquatic Centre, 4 Boyd Street, Blacktown
aqua.blacktown@blacktown.nsw.gov.au - **02 8869 9000**

Stanhope Gardens
Blacktown Leisure Centre Stanhope. Sentry Drive, Stanhope Gardens
aqua.stanhope@blacktown.nsw.gov.au - **02 9421 2600**

Emerton
Charlie Lowles Leisure Centre Emerton. Jersey Road, Emerton
aqua.emerton@blacktown.nsw.gov.au - **02 8822 6000**

Mount Druitt
Mount Druitt Swimming Centre, Ayres Grove
aqua.mtdruitt@blacktown.nsw.gov.au - **02 9625 6360**

Riverstone
Riverstone Swimming Centre. Corner Market and Piccadilly Streets
aqua.riverstone@blacktown.nsw.gov.au - **02 9627 1496**

Castle Hill
Castle Hill Fitness & Aquatic Centre. 77 Castle Street, Castle Hill, NSW 2154
chfac@chrg.com.au **(02) 9846 1200**

Granville
Granville Swimming Centre, Enid Avenue, Granville.
https://www.cumberland.nsw.gov.au/granville-swimming-centre
02 8757 9050. (Central number for Granville, Guildford and Merrylands pools.)

Merrylands
100 Burnett Street Merrylands - **02 8757 9050**
https://www.cumberland.nsw.gov.au/merrylands-swimming-centre

Wentworthville
115 Dunmore Street, Wentworthville - **02 8848 7700**
https://www.wentworthvilleswimcentre.com.au

Guildford
Guildford Indoor Centre, 1 Tamplin Road Guildford - **02 8757 9050**
https://www.cumberland.nsw.gov.au/guildford-swimming-centre

Auburn
Auburn Ruth Everuss Aquatic Centre, Church St, Lidcombe - **02 9749 5031**
https://www.auburnaquaticcentre.com.au

Epping
Epping Swimming Centre, Dence Park, 26 Stanley Road, Epping - **02 9806 5633**
https://www.cityofparramatta.nsw.gov.au/recreation/swimming/epping-aquatic-centre

Parramatta
Macarthur Girls High School Pool, 1-19 Macarthur Street, Parramatta
(entrance & parking on Stewart Street) - **02 9806 5700**
https://www.cityofparramatta.nsw.gov.au/recreation/swimming/macarthur-girls-high-school-pool

Royal Life Saving Academy Australia Aquatic Academy
78 Best Road Seven Hills, NSW
(on the grounds of Hills Sports High School)
Phone **02 8814 8637**

ROYAL LIFE SAVING AUSTRALIA | AQUATIC ACADEMY

The Aquatic Academy delivers the Royal Life Saving Swim & Survive program, a broad and balanced program teaching a range of skills and knowledge to develop swimming, water safety, survival and basic rescue skills. It aims to develop a strong and safety aware swimmer who has a healthy respect for any aquatic environment.

Swim and Survive is a swimming and water safety initiative of Royal Life Saving that seeks to increase swimming and water safety skills of Australian children in order to prevent drowning and increase participation in safe aquatic activity.

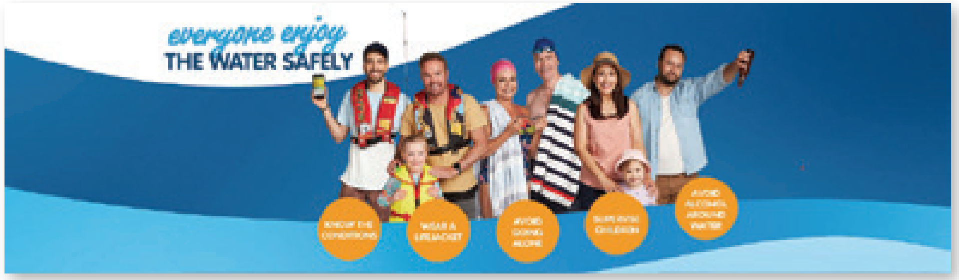

Swim and Survive ensures that the essential components of personal survival and water safety are provided, in addition to a process of developing strong and effective swimming technique.

Swim and Survive provides learning pathways incorporating three programs that provide child focused swimming and water safety education for each of the key life stages between 6 months and 14 years.

Learning swimming, survival and lifesaving skills is a lifelong process. Royal Life Saving recommends continuous participation and regular practice to develop and consolidate skills for a lifetime of fun and safe aquatic activity.

The Aquatic Academy has also developed a flexible visit pass system for adults looking to learn to swim.

Sydney Harbour Kayaks

Phones: **02 9969 4590** / **02 9960 4389**
Email: info@sydneyharbourkayaks.com.au
Address: **81 Parriwi Road, Smiths Boat Shed, The Spit Bridge, Mosman**
https://www.sydneyharbourkayaks.com.au

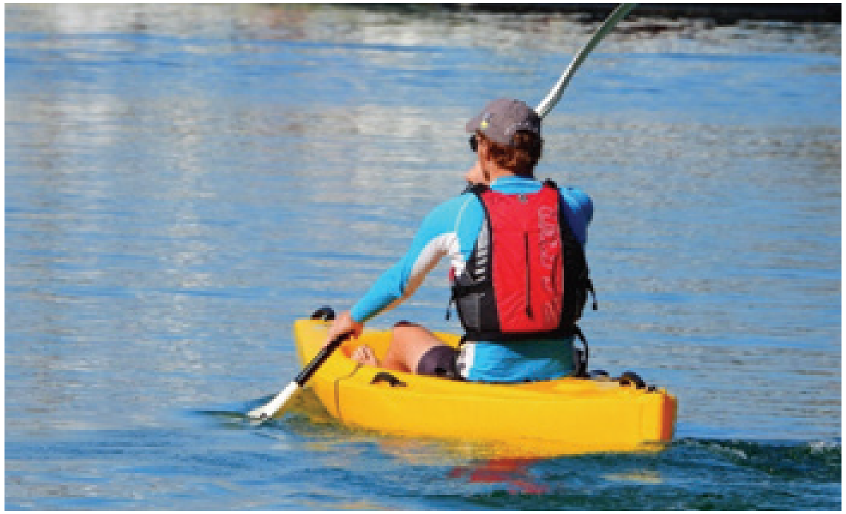

Free Three Hour Introduction to Kayaking Course

Start your paddling experience safely and comfortably with a Free Three Hour Paddling Session from Sydney Harbour Kayaks' Professional Paddling Staff.

The Intro to Kayaking lesson is the simplest introductory course, where we will focus on establishing the basic concepts that will allow you to be self-sufficient and efficient in your paddling.

We will cover many important aspects of paddling including safety and basic strokes, that will allow you to learn to manoeuvre your kayak (ie keep it going straight!) and allow you to enjoy the process of paddling.

If you do not have your own kayak, then we will be teaching you in a stable and comfortable sit-on-top kayak. The kayak rental will cost $55.00 for the duration of the 3 hour lesson.

You must be a beginner paddler, however you can do this lesson several times until you feel confident when paddling.

Note: we can teach children as young as 12 years of age however they must be accompanied by an adult. This is a great family activity, so why not learn as a family.

Rowing NSW

"Learn to Row" programs are designed to teach basic rowing skills to people who have never rowed before. After completion, you may like to take the sport further by becoming a member of Rowing NSW and a Recreational Member of an affiliated club: https://www.rowingnsw.asn.au/home/

Nepean Rowing Club

Address: PO Box 7344, Penrith South 2750
Club Phone: **02 47312255**
Boatshed: **Bruce Neale Drive, Penrith**
Contact: **Daniel Waddell**
rowing@nepeanrowingclub.com.au
https://www.nepeanrowingclub.com.au

Learn To Row Programs

Programs are run every weekend from 9-10am
Contact Person: **Daniel Waddell (0403 743 189)**
Cost: **$30 per session**
Venue: **Nepean Rowing Club, Nepean River, Bruce Neale Drive, Penrith**

Penrith Rowing Club

Address: **38 Bruce Neale Drive, Penrith 2750**
Club Phone: **0408 699131**
Boatshed Phone: **0408 699131**
Contact: **Fiona Toose**
penrithrowing@gmail.com
http://www.penrithrowing.com

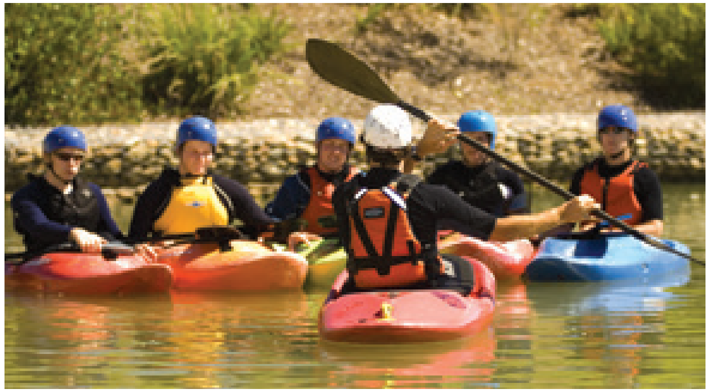

Learn To Row Programs

Programs are run on a Sunday for 2 ½ hours. There are 8 programs consisting of 5 lessons run each year. The cost is $150 per program. Bookings are essential. One-on-one learn to row classes are also available throughout the year, price and availability upon request.

Contact Person: **Fiona Toose (0408 699 131)** Email: penrithrowing@gmail.com

Venue: **Penrith Rowing Club, Nepean River, Schools Boatshed, Bruce Neale Drive, Penrith**

PaddleNSW

Learn to Paddle

Good basic skills are the foundation of paddling flatwater, whitewater or out in the ocean. Learn to paddle for fun or competition, and participate in Club events, State or National Championships.

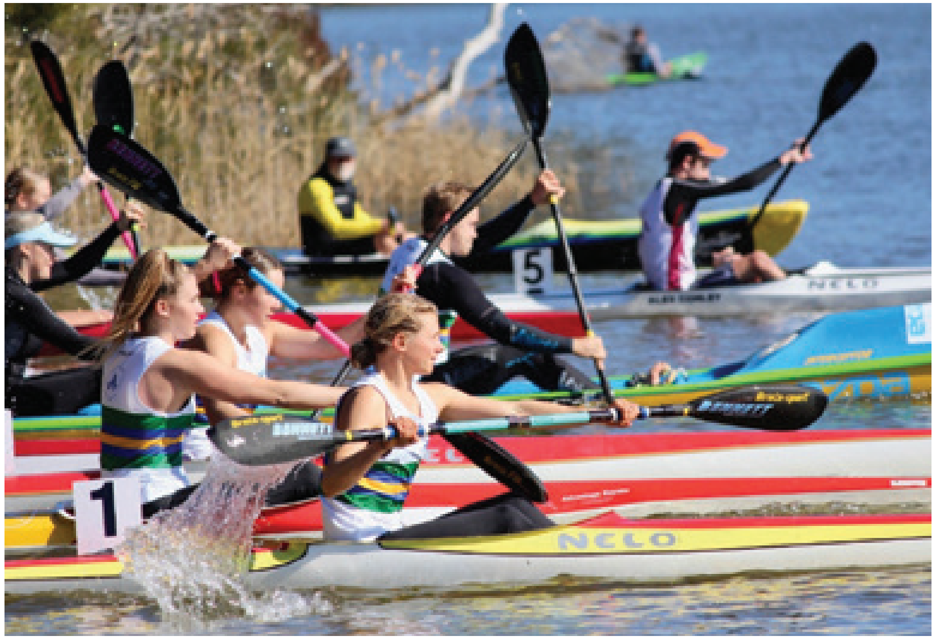

Explore the PaddleNSW website and find places to paddle, clubs to join, and qualified instructors in your area.

You can become involved in a range of activities like touring magnificent waterways, playing canoe polo, and participating in sprints, marathon, slalom or freestyle.

Clubs are the foundation of paddling in NSW, providing strong communities and opportunities to develop your paddling. Whether you're looking for competition, recreation, training or opportunities to volunteer, there's sure to be a good fit close to you. Enjoy the greatest lifestyle imaginable: http://www.nsw.paddle.org.au

Penrith Whitewater Stadium

Kayak Instruction

Would you like to learn a new and exciting sport at one of the world's best facilities?

Then why not give kayaking a try at Penrith Whitewater Stadium, the competition venue for the canoe/kayak slalom events during the Sydney 2000 Olympic Games.

Let our talented and enthusiastic instructors build your confidence and broaden your paddling horizons.

Penrith Whitewater offers a variety of lessons and courses that vary in length from a one hour lesson to multi-day skills improvement courses.

Instruction is offered in two basic forms – group or private. Check out the website for details on the courses and see our 'how to book' section for important booking information

https://www.penrithwhitewater.com.au

Details: **McCarthy's Lane, Cranebrook: 02 4730 4333**

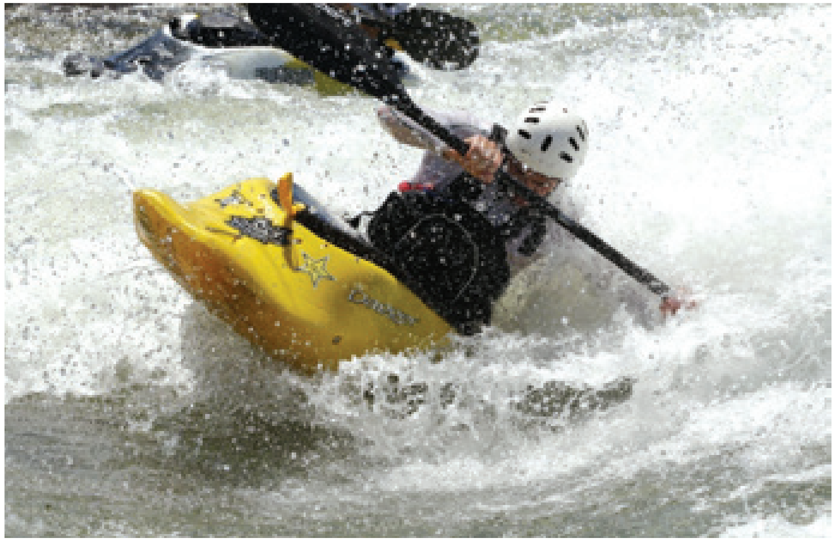

Wisemans Ferry Water Sports

Waterski Instruction

At Wisemans Ferry Water Sports you can learn to water ski if you are a beginner, or if you have water skied before, you can learn new skills and improve your style – and you don't have to own a boat! The school uses the latest coaching techniques and equipment and the boat is fitted with a boom bar to help you learn the easy way. All the coaches are accredited by the Australian Sports Commission.

The Ski School operates from the NSW Water Ski Federation Ski Grounds, River Road Wisemans Ferry

Email: skischool@skischool.com.au
https://www.skischool.com.au

NSW Ski Gardens
2916 River Road, Wisemans Ferry NSW 2775
Bookings essential: **02 4566 4544.**

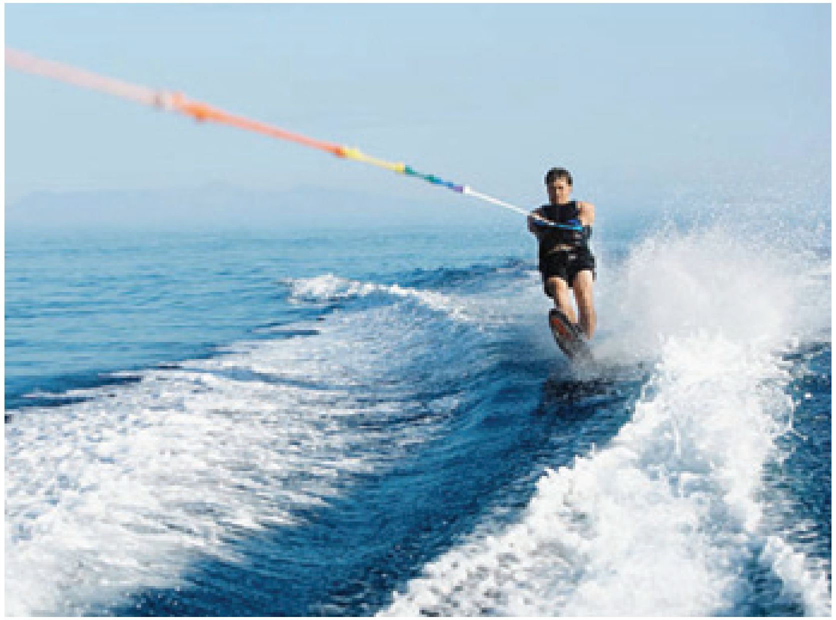

Section 3

CLUBS TO JOIN

Bushwalking clubs

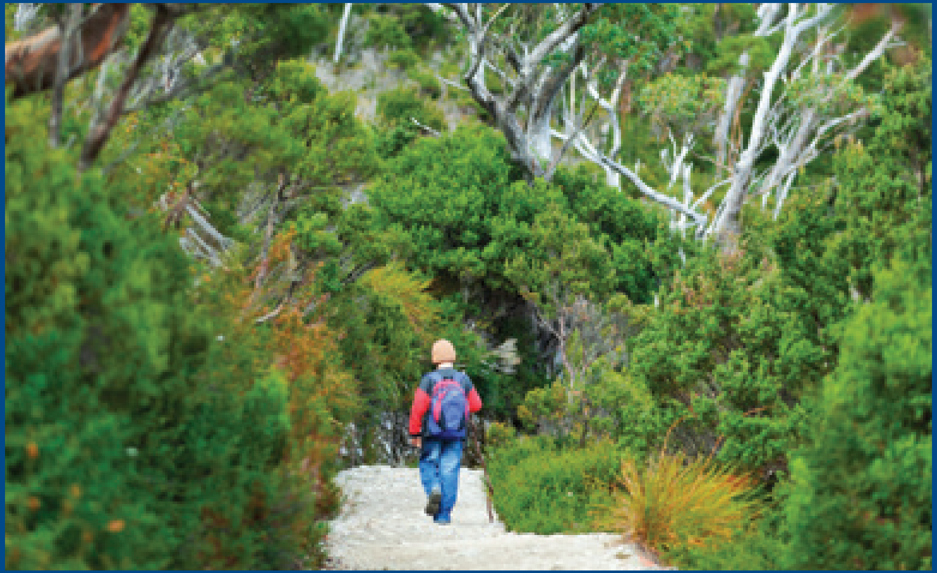

Bushwalking NSW

This is a really helpful website which will give you a rundown on all the bushwalking clubs in NSW.

There are many very active friendly clubs in western Sydney which are all outlined. You can also find out what other activities they may be involved in such as multi-day walks, cycling, mountain biking, paddling, canyoning and even cross-country skiing.

This website also gives excellent advice on how to look for a club which will suit your specific requirements.

If you need convincing as to why you should join a bushwalking club, read on.

Why join a club?

Fun: Walking and outdoors adventure is more fun with a club and you get to make new friends. Clubs are friendly places, and always welcome new members. They give you the opportunity to meet people of all ages, and from all walks of life, who share your interest in bushwalking and outdoor adventure.

Safety: It's much safer to walk with a club. There is safety in numbers, and with experience. In case of an accident or emergency the group will be able to handle the situation much better than one or two bushwalkers on their own. If you're unsure of your skill in map and compass navigation, the club leaders will be able to guide you safely. Clubs share loads of great knowledge and experience – often for free!

Adventure: Being a club member allows you to discover new places to walk – in your local region, the rest of the state, country and across the world! The clubs often arrange weekends or weeks away exploring exciting walking destinations. These can vary from a 2 hour walk, bike-ride, paddle or social gathering, all the way to a multi-day wilderness trek, and everything in between!

Low cost: Most trips cost nothing more than your fuel, food and gear.

Many clubs offer visitor membership so you can try them out by attending a couple of walks without becoming a full member. This way you can meet some of the people and experience the kinds of walks the club does.
Details: https://www.bushwalkingnsw.org.au/clubs-location

Wanderers Bushwalking & Outdoors Club

The Wanderers, is a Bushwalking Club founded by an enthusiastic group of outdoor lovers in 1976.

Since then an organisation that possesses a special camaraderie between members has developed. The members turn up in significant numbers every weekend for the walks and other programmed activities. The activities the club organises comprise easier city and coastline walks of the lesser grades, to harder walks of higher grades.

Camping and accommodated weekend activities include walks around the local area.

Our Club which is affiliated with Bushwalking NSW Inc is based in the Hills District of Sydney. The Wanderers Bushwalking and Outdoors Club currently has just over 100 members: https://www.bushwalkingnsw.org.au/wanderers/about

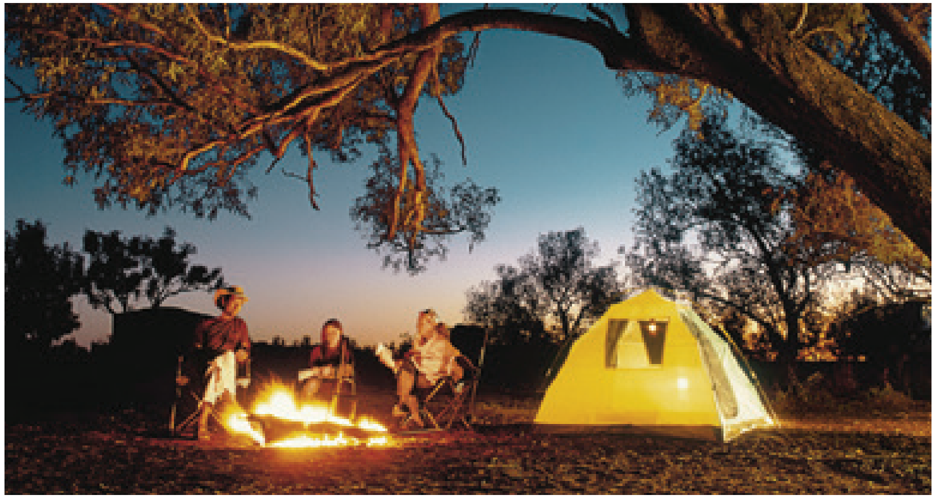

Cumberland Plains Walkers

The Cumberland Plains Walkers is a small, friendly club with members from all over Greater Western Sydney.

It is a non-profit, volunteer association, made up of members who devote their time to leading walks for the enjoyment of all.

Cumberland Plains Walkers Inc offers you the opportunity to meet people with the same interests, to see the beauty of our National Parks and wilderness areas and to enjoy the adventure of exploring them through a wide range of activities. You can view magnificent rugged scenery and see many varieties of flora and fauna, as well as enjoying the tranquility the outdoors has to offer.

There are activities for all ages, you are never too old! Training is available and you will find it very easy to learn.

Bushwalking is the ultimate experience. Camping in a clearing by a cool mountain stream or perched high on a mountain, far from the cares of the world, adds warmth to a walker's soul.

The club supports minimal impact bushwalking and camping plus generally caring for and respecting the environment.
Email: cpwclub1@gmail.com

Clubs of Australia

This website is an online club and association directory for over 30,000 clubs in Australia. You can find clubs related to a variety of categories and they provide a short description, location, email and website links. There are a huge number of categories available including bushwalking, camping, canoeing and kayaking, running and walking clubs. Plenty of options to get you out and about.

Details: https://www.clubsofaustralia.com.au/clubs-in-new-south-wales-84/

Fishing Clubs

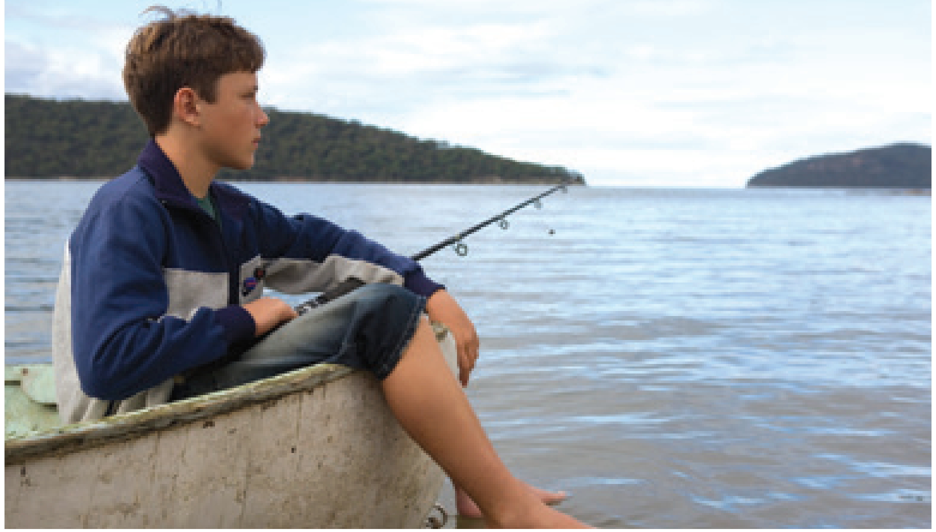

SeaBees Fishing Club

SEA BEES FISHING CLUB INC

The SeaBees is a non-profit social/fishing club located in The Hills district of Sydney.

The club currently has about 100 members – men, women, teenagers and young children. It recently celebrated its 40th Anniversary.

They meet at **Crestwood Community Centre, Crestwood Drive, Baulkham Hills.**
Details: sydneyseabees@gmail.com

Western Sydney Bream & Bass

Western Sydney Bream & Bass is a Sydney based fishing club and is an all year round fishing experience that allows anglers to enjoy a social, educational and fun atmosphere. Members can be involved in angling, monthly meetings, seminars, prize giveaways and much, much more,

Monthly Seminars, prize giveaways and much, much more. Whether it be a boat, canoe or kayak, WSBB offers opportunities to all anglers to fish all major waterways around Sydney.
http://wsbb.com.au

Triathlon Clubs

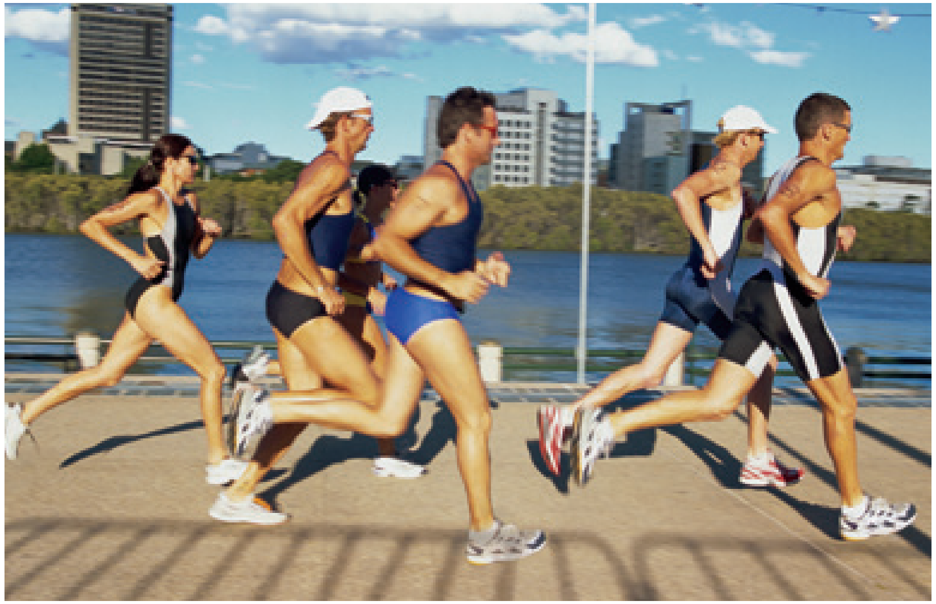

Hills Triathlon Club

What is Triathlon?
Triathlon is a mix of three sports that you do all in a row for an overall race time.

Swim-Bike-Run
There are various distances to compete in. For new people to the sport, we strongly recommend you start out and do an 'enticer' event for your first race to get a feel for things. At Hills Tri Club our members race across all distances, however our own race series is 'Sprint', 'Enticer' and 'Junior' distance events.

Hills Triathlon Club is an inclusive, fun club for both beginners and experienced triathletes of all ages and abilities. Join a great bunch of people to train and race with all year round.

Hills Triathlon Club runs a triathlon series, Swim Bike Run through the warmer months of the year. These events have a variety of distances to encourage participation for all levels and ages of athletes and visitors are welcome. As the cooler months arrive we have a duathlon series called Run Ride Run.

Our Triathlon Series and Duathlons are held at the Sydney International Regatta Centre and our races are safe as it is a closed facility for us to enjoy racing.
https://hillstriclub.com/

Outdoor Fitness Classes

Live Life Get Active

Live Life Get Active is a registered health promotion charity with full DGR status. We provide FREE outdoor fitness classes to Australians in 100's of locations across the country with a clear mission to address chronic health issues such as obesity, diabetes and poor mental health.

Our proposition is simple – get people up and moving and having fun in a group. Help them understand their health issues, lifestyle behaviours and get them to review how they eat. We provide FREE daily activity programs that include yoga, cross training and boxing. FREE tailored nutritional programs and FREE educational wellness content. All our services are delivered by our team of in-house and local professionals. It's a proven interlinking strategy that gets results and changes lives. What makes us unique is that we can measure our outcomes and show our impact on both physical and mental health.

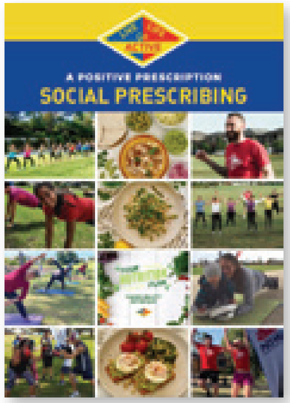

What started as an in park offering has expanded to include a full online program with daily LIVE classes, online LIVE workshops and a library of over 300 pre-recorded fitness, nutrition and wellbeing videos. Which means we are available to any individual 24/7 and can reach right into rural and remote communities.

As well as providing our services to councils, state government, PHNs and commercial enterprise, we work actively with GP's and health professionals providing a service direct to their patients FREE of charge in the form of a social prescription.

https://livelifegetactive.com/about/

Meetup

Whatever you're looking to do outside this year, Meetup can help. For 20 years, people have turned to Meetup to meet people, make friends, find support, and explore their interests. There are groups in western Sydney for most kinds of outdoor activities including hiking, exploring, adventure, bushwalking, mountain biking, kayaking, sailing, camping, and cycling clubs.

Go onto the website and enter the category of group you're interested in and specify a Sydney location. Each Meetup group has its own page, where you can get learn more about what they do, see what kind of people attend their events, and get an idea of how large the events are. Become a member of a group that interests you to get updates on what the organisers are planning. Thousands of events are happening every day—join the fun.

https://www.meetup.com/en-AU/

Parramatta Exploration Group

Parramatta Exploration Group is the place to be if you want get out and explore the stunning grandeur of the Blue Mountains and the rivers, creeks and walking tracks of your own local environment.

If you just want to get outdoors and enjoy walking with new found friends in a relaxed atmosphere then this is what they're all about.

You're welcome to join in and share your own experiences as part of an expanding and progressive organisation where members are first and foremost. Only two rules, firstly, it's free to participate and most importantly, get out there and enjoy yourself.

https://www.meetup.com/en-AU/Parramatta-Exploration-Group/

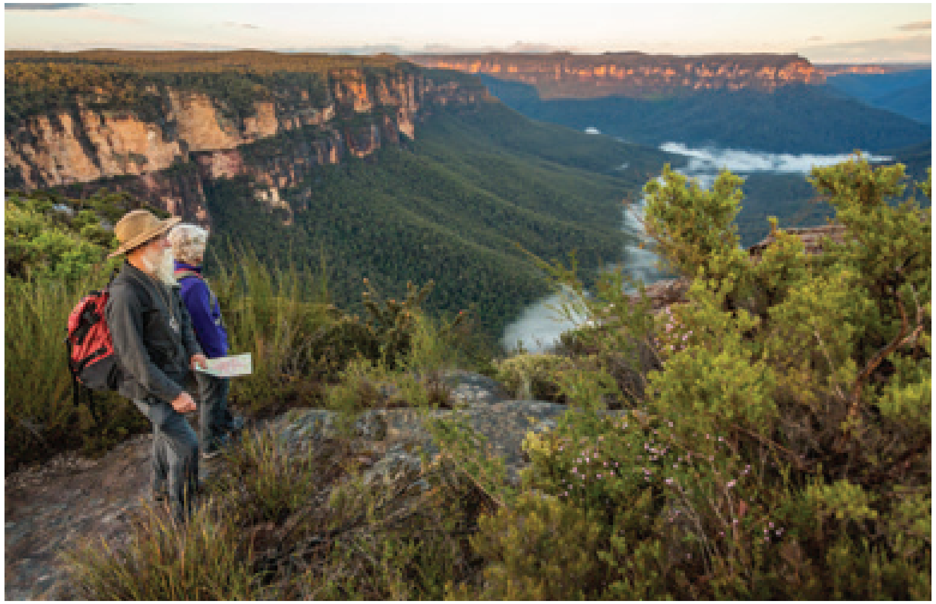

Orienteering NSW

Orienteering is a sport that challenges both the body and the mind. It's also loads of fun!

The aim is to use a special orienteering map to navigate your way around a course and visit marked check points along the way. You choose a course that suits your age and experience and proceed at your own pace: walk, jog or run. It's a race, but you decide if you want to just race yourself or be the next world champion! The course may take you through urban areas, parks, schools, farmland or forests.

Events are conducted weekly across NSW and beginners are welcome at all events.

It is mostly done solo, but you may also go with a friend or in a small group.

Beginners are always welcome. Each event offers courses for newcomers, and host club members will be happy to get you underway.

Bold Horizons organises the Saturday Orienteering Series and has events based in the west.

Saturday Orienteering Series (boldhorizons.com.au)
Orienteering NSW https://onsw.asn.au/events/summer-series/summer-series-west

Western and Hills Orienteers Inc. (WHN)

This club covers the Parramatta, Blacktown, Penrith and Hills District. You may like to look at their website to check out all the local events.
https://whorienteers.net

parkrun

parkrun is the name, but look a bit closer and you'll discover why this simple intergenerational activity has captured the imagination of more than half a million Aussies in 350 communities around the country.

The concept is simple and completely free! parkruns are established and coordinated by volunteers from the local community. Each event takes place in a public open space every Saturday at 8.00am on a 5km course. People are invited to take part in whichever way they choose- walk or run the route, lend a hand as a volunteer, or simply spectate and socialise. After the event it's off to a nearby café or meeting place for a coffee and a chat.

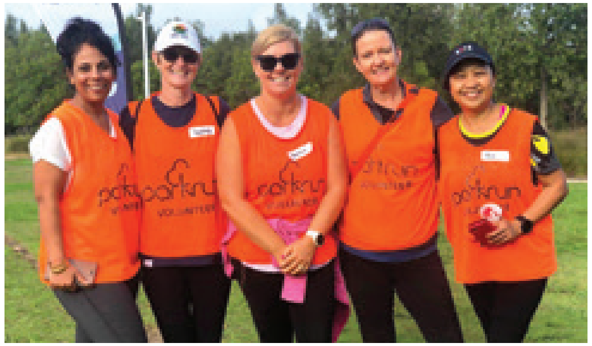

parkruns are non-competitive with the emphasis on regular participation rather than performance. The benefits of parkrun for individuals and communities are extensive and parkrun is being increasingly utilised by primary healthcare professionals as a social prescription.

Register for free before your first parkrun at www.parkrun.com.au/register, print your personal parkrun barcode and take it along to any parkrun in the world on any Saturday. It's that easy!

Details: www.parkrun.com.au

Ramblers

This is a very active club which provides recreational bushwalking for exercise, an appreciation of the natural environment and social interaction. It also promotes conservation of native flora and fauna.

Walks are scheduled both on weekdays and weekends. Walks are based mainly in the Sydney metropolitan area and Greater Sydney region. Most can be accessed by public transport. Each walk is graded according to its difficulty and a variety of walks are offered to cater for different abilities.

These include:

- Day walks such as historical city walks, harbour and coastal track walks, and harder bushwalks in national parks, e.g. Blue Mountains National Park, Royal National Park, Ku-ring-gai Chase National Park.
- Short holidays with day walks. Accommodation is usually in cabins, hotels and motels. Public transport or shared private vehicles are used to transport walkers to the holiday location. Recent destinations include Kosciuszko National Park and Blue Mountains National Park.

Prospective members are welcome to attend three walks before joining the club, but must notify and gain permission from the walk leader/s in advance. To obtain an information package and details of Short Notice Walks, prospective members should contact enquiries@wearamblers.org.au

Details: http://wearamblers.org.au

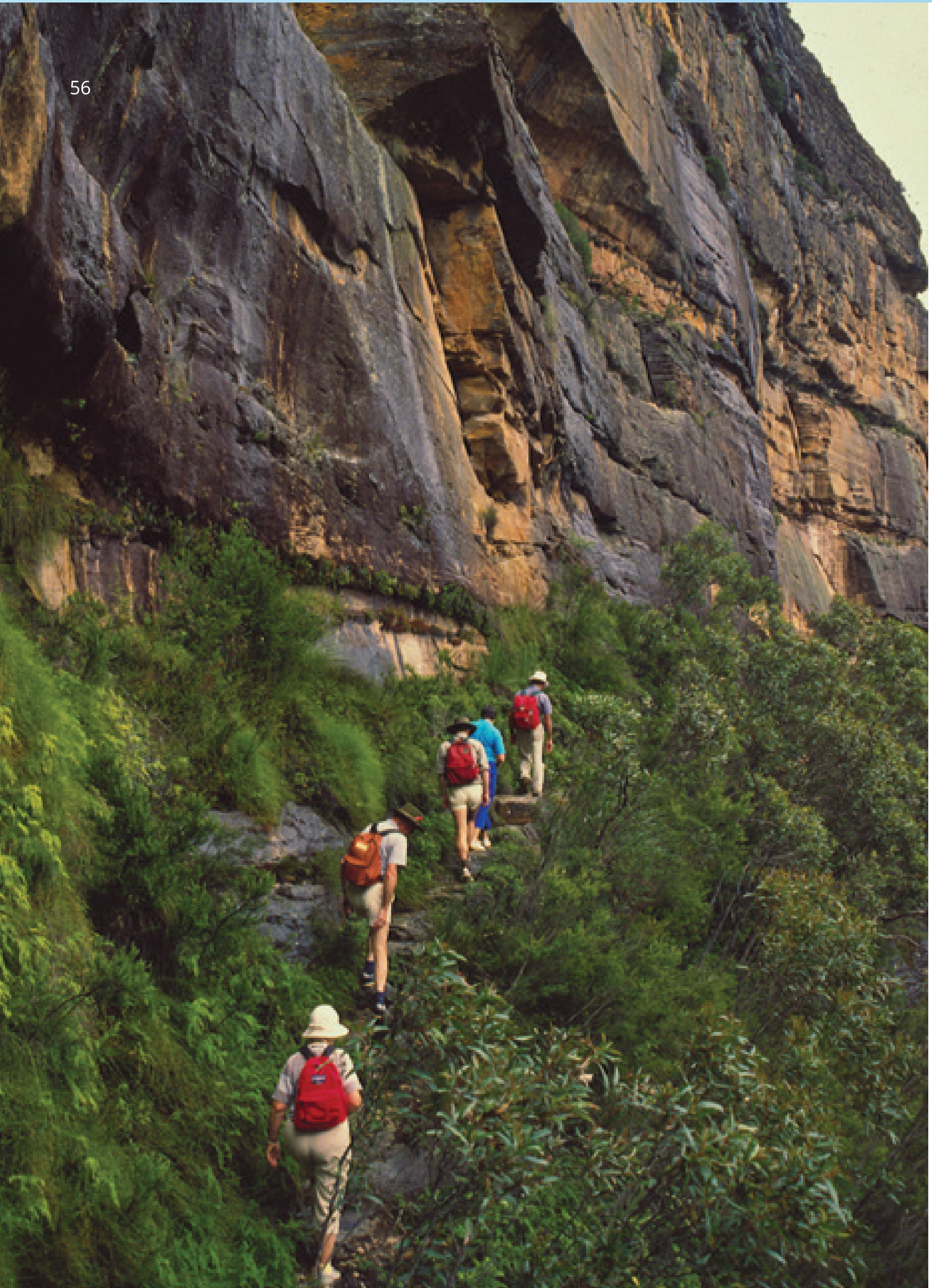

Heart Foundation Walking

Heart Foundation Walking offers two ways in which you can start walking your way to a healthier you. Whether you are interested in walking in a group or on your own, we have plans that can suit your needs.

Heart Foundation Walking Groups is a group-based walking program with regular walks led by community volunteer Walk Organisers. Heart Foundation walking is Australia's largest FREE walking network. It is a social, fun and an easy way for people to walk and be active. Walking is suitable for people of all ages and fitness levels.

The popularity of the walking groups is evident in that over 80% of walkers are continuing to participate after six months. As well as the physical benefits, activity can also make you feel happier and improve your quality and enjoyment of life. Walking groups come in different sizes and walk at various times of the day, days of the week, for varying distances and differing levels of difficulty.

Heart Foundation Personal Walking Plans are more than information on how and when to exercise. Over six weeks, we'll help you experience pleasure in movement and connect with what's going on in the world around you. With motivational text messaging, confidence in your ability will grow, your strength and flexibility will increase, and you'll generally feel better.

To find a group suitable for you or to register, call the Heart Foundation on **13 11 12** or email walking@heartfoundation.org.au

Or visit the website http://walking.heartfoundation.org.au to sign up for your free Personal Walking Plan or learn more about the benefits of exercise.

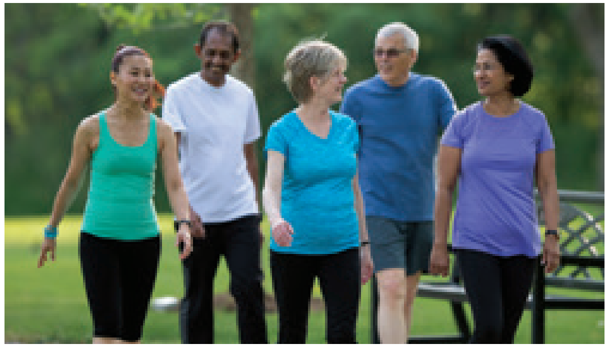

Section 4

NATURE

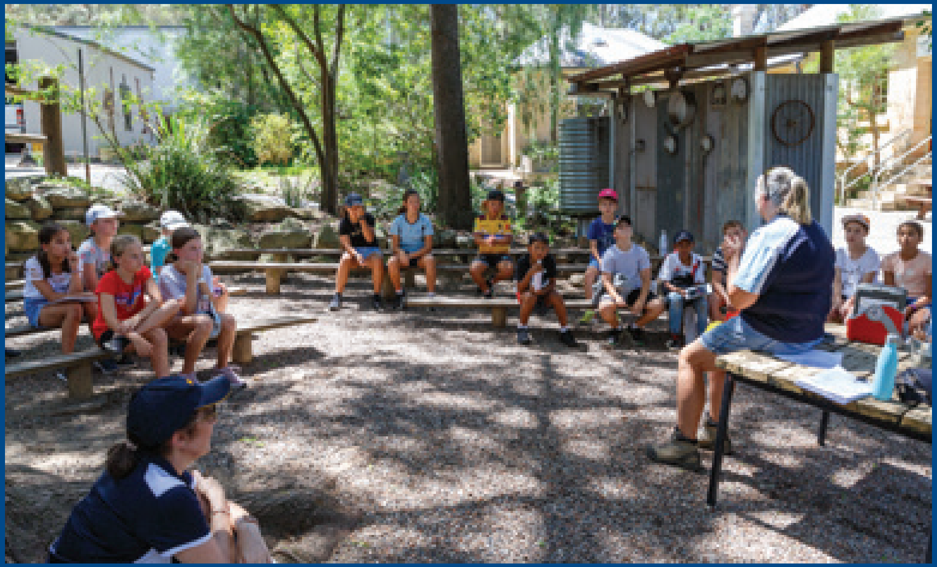

Birds Australia Discovery Centre

Located adjacent to the Newington Nature Reserve in the heritage precinct Newington Armory, the Birds Australia Discovery Centre is open to the public every Sunday and provides regular monthly tours, an extensive bird book library, education room and interpretation facilities.

The adjoining Nature Reserve is a haven of significant environmental value including bird habitats, and the facility is the centrepiece of Birds Australia's engagement with the community, providing education about biodiversity and the value of birds and their habitats.

Newington Armory, Building 133 1 Jamieson Street, Sydney Olympic Park
(02) 9647 1033
southernnsw@birdlife.org.au
https://birdlife.org.au/visit-us/discovery-centre

Brewongle Environmental Education Centre

A NSW Department of Education facility providing inspiring and authentic curriculum based day and camp programs for all schools and students from K-12. Our vision is to connect every learner to the natural world and inspire change for a sustainable future.

Brewongle EEC embraces themes of environment, sustainability and Indigenous values among many others.

The centre is part of the wider Environmental and Zoo Education Centre (EZEC) Network that leads environmental education in NSW to empower learners for a sustainable future.

Brewongle is a Darug Aboriginal word that we think is derived from the local Boorooberongal Clan of the Darug People who cared for this land of mighty rivers, lagoons, giant blue gums, forests and verdent plains. A rich country with the rugged Blue Mountains to the west, Cumberland Plain to the south and endless sandstone ridges and rainforest gullys to the north. A land of kangaroo (booroo), possum and koala, of eels, ducks and fish, of goanna, sea eagles and black cockatoos. We acknowledge and pay tribute to the giants of sustainability, The Darug People.

Wherever possible, local Indigenous knowledge is incorporated into Brewongle programs. Our strong connections with local Elders and Community help us to deliver meaningful, accurate and current culture. Whether through traditional storytelling, games and ways of learning, making a traditional canoe or learning about local bush resources, Darug Culture is woven through curriculum at Brewongle. These connections and more enable us to deliver highly regarded teacher professional learning courses.

Web: https://brewongle-e.schools.nsw.gov.au
Email: brewongle-e.school@det.nsw.edu.au
Facebook: https://www.facebook.com/BrewongleEEC

Bushcare

Bushcare is a volunteer-based, environmental conservation program coordinated and supported by local councils. Bushcare is a great way to meet new people, have fun and help improve the natural environment. Bushcare also helps to protect and preserve bushland that our local wildlife lives in. Join a local Bushcare group to care for a nearby bushland reserve.

Bushcare Groups meet once a month to assist with activities such as weed removal and mapping, habitat monitoring and restoration, tree planting and surveying. You can also be involved in community education, plant propagation and training/workshops

Become a part of something bigger than yourself. Involvement with Bushcare not only allows you to give back to the environment, but also supports you to:

- Learn about your local, natural environment and receive expert guidance
- Meet new, like-minded people
- Learn new skills, and learn how to use new equipment and tools
- Protect and care for the environment with restoration training
- Help educate the community
- Get outdoors and stay active
- Continue environmental education with Bushcare meetings and flora and fauna workshops.
  https://www.aabr.org.au/volunteering/bushcare-and-landcare-volunteering

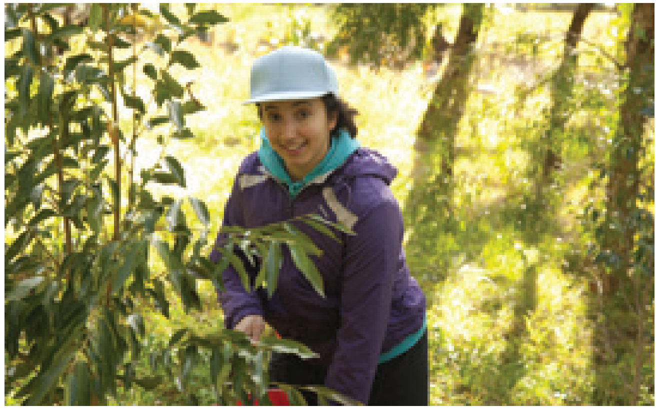

Source: Blacktown Council Bushcare program

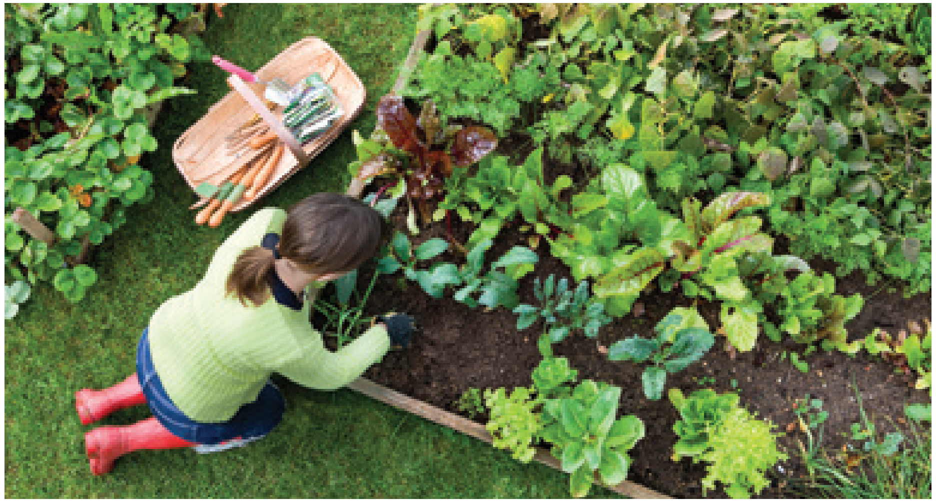

Community Gardens

Community gardens are places where people come together to grow food, create habitat and connect with nature and their local community. They are places where community members can learn new skills, help improve our community's access to fresh food, make new friends, and share in the work of maintaining the garden.

We're very fortunate that here in western Sydney there are a large number of community gardens run by groups and in many cases the local council that you may like to join. Some are purely communal, whereas others provide access to your own plots where you can grow food for your family and friends.

Here is a list of some of the local gardens. Due to covid-19 restrictions, check first with the garden organiser before visiting these gardens:

Bidwill Community Garden

Location: Chestnut Crescent, Bidwill
Contact: **0415 836 531** or email: arnesenlisa.la@gmail.com
Opening times: 9am-2pm on Wednesdays, Fridays and Sundays

The garden has a number of themed garden beds of edible and non-edible plants, including vegetable gardens, orchard of fruit trees, a native patch and succulents!

Bungarribee Community Garden

Location: 20 Sir Hercules Drive, Bungarribee
Contact: ourenvironment@blacktown.nsw.gov.au
Opening times: 9:30am-11:30am Thursdays

The garden has a number of attractive productive vegetable beds, with natives and flowering plants to attract good insects. The garden also features a drip irrigation system and rainwater tank.

Lalor Park Community Garden

Location: Parkside Drive, Lalor Park
Contact: lalorparkcommunitygarden@gmail.com
Opening times: Thursday and Saturday (9am-12 noon)

The Lalor Park Community Garden is a not-for-profit community focused group run entirely by volunteers. The garden grows a variety of vegetables, fruit and herbs. We encourage the community to join our membership and garden with us.

Lindley Square Community Garden

Location: Lindley Square, Bidwill
Contact: ourenvironment@blacktown.nsw.gov.au
This is a new community garden in Bidwill that is currently in the planning and construction stages. If you would like to get involved, contact ourenvironment@blacktown.nsw.gov.au and we can put you in touch with the local residents who are preparing to start this garden.

Quakers Hill Uniting Community Garden

Location: 32 Highfield Road, Quakers Hill, Contact: **9837 2001**

The Gardening Group at Quakers Hill Uniting Community Garden is a strong mix of local community members, Noah's Ark Pre-school children and parents, families from Quakers Hill Family Services and families from Quakers Hill Uniting Church. Some are whole families and some are individuals but all enjoy the invigorating joy of working in the garden and seeing the garden grow.

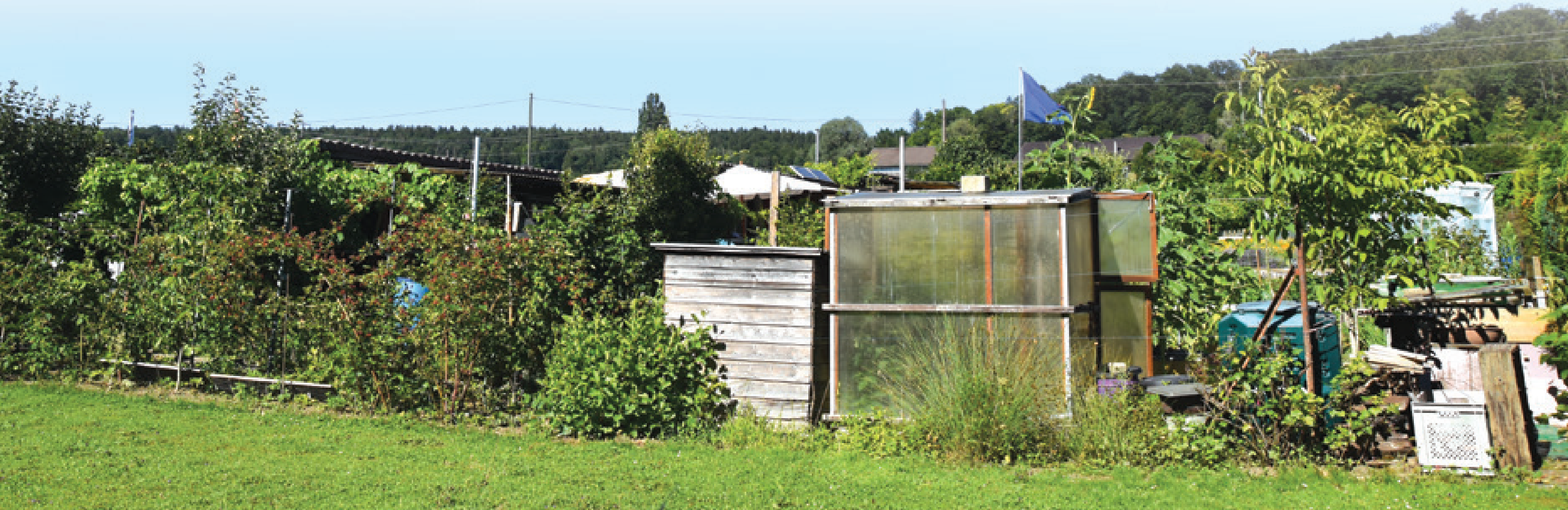

Riverstone Community Garden

Location: Park Street, Riverstone
Contact: Riverstone Neighbourhood Centre **9627 3622**, or email: reception@riverstone.org.au Opening times: Tuesday, Wednesday, Thursday 9am-1pm. The garden has a number of community beds for organisations (Bhutanese group, Dementia Connections, Mens' Shed) and some individual garden allotments.

Showground Community Garden

Location: Blacktown Showground, Garden Gate, Richmond Road, Blacktown
Contact: ourenvironment@blacktown.nsw.gov.au
Opening times: Tuesday 9am-11:30am, first and third Saturday each month 8:30-11:30am, second and fourth Sunday each month 8:30-11:30am

Productive diverse gardens for growing vegetables, fruit trees and native plants and shrubs. Irrigation system to water the garden, with shared gardening activities. This garden has concrete paths and raised garden beds to help people with disabilities access them.

The Ponds Community Garden

Location: The garden will be located next to The Ponds Community Hub, 45 Riverbank Drive, The Ponds, Contact: ourenvironment@blacktown.nsw.gov.au This is a new community garden in The Ponds that is currently in the planning stages. If you would like to get involved, then contact ourenvironment@blacktown

Willmot Community Garden

Location: Willmot Public School, Contact: **9628 0222**

Community garden cared for by the community and the school. Please contact the School Office for more details. nsw.gov.au and we can put you in touch with the local residents who are preparing to start this garden.

Wentworthville Community Garden

Address: 73 Fullagar Rd, Wentworthville
Opening times: Wednesdays 3:30-4:30pm, Saturday 10am-1pm and Sundays 3:30-4:30pm, weather permitting. Contact: **0420 893 186**

Wentworthville Community garden is a place where you can grow your own veggies, spend time with others or simply sit and relax.

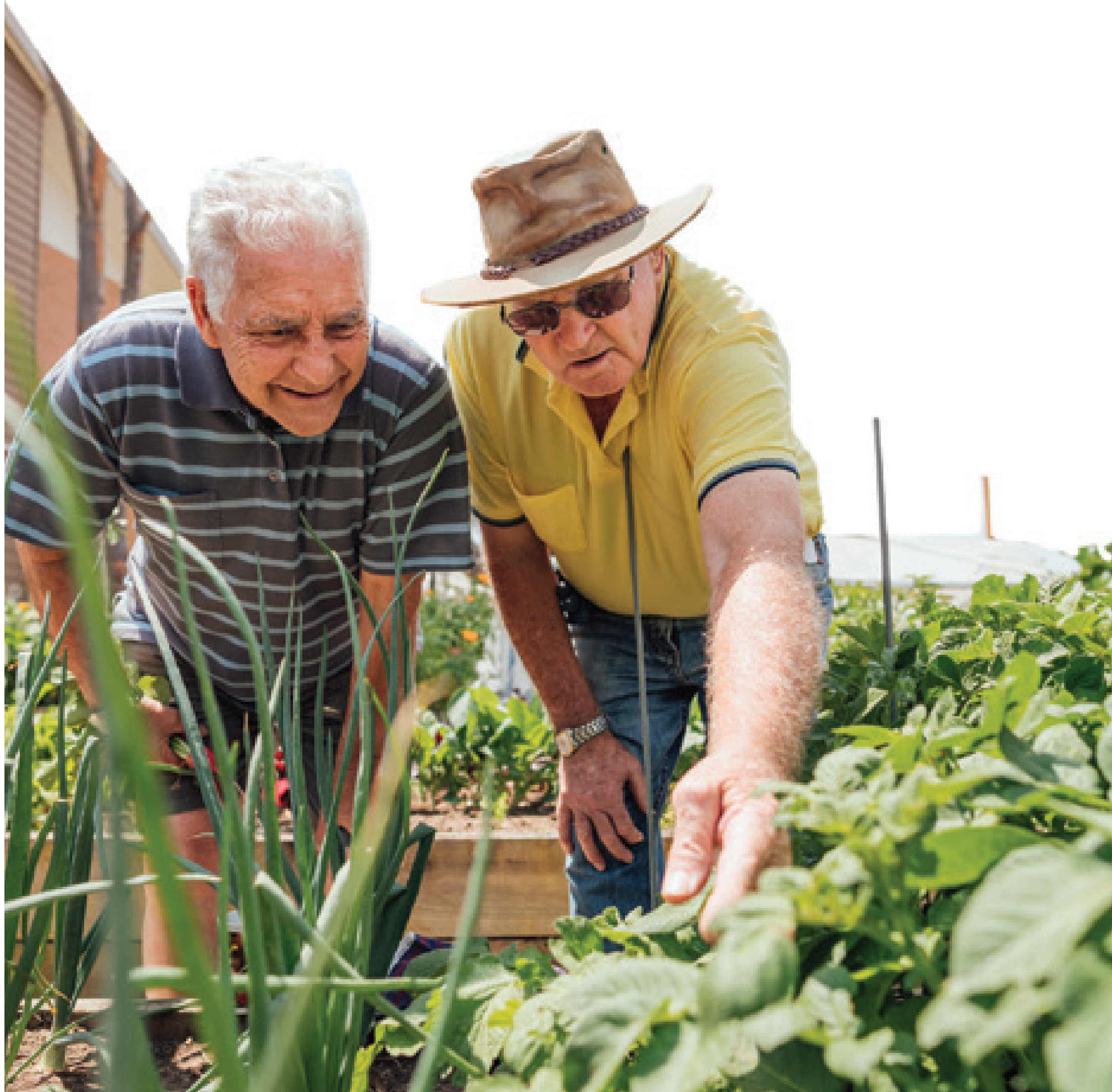

Conservation Volunteers

We're creating a world where people and nature flourish together.

Conservation Volunteers Australia is a national organisation working with communities to rebalance nature for a stronger, more resilient future. We combine evidence-based practices and people power to make real and lasting change at scale.

Together we take action for nature across a wide range of opportunities to nurture healthier communities and more resilient ecosystems. From ocean litter to bushfire resilience, and citizen science to climate change – there's something for everyone.

The Community Environment Program (CEP) is supporting community groups to address local environmental priorities. The program is also encouraging the community to connect with their local natural environment and building and strengthening local communities.
https://conservationvolunteers.com.au

Garden Clubs

Garden Clubs

The Garden Clubs of Australia Inc. is a not-for-profit umbrella group established to provide encouragement and support for over 780 autonomous garden clubs and societies. These are like minded people who share a passion for gardening across the Nation supported by the motto 'Friendship through Gardens'.

The primary and major object of The Garden Clubs of Australia is to extend the culture of gardening into the wider community for the benefit of all citizens and, in particular:

- To promote and encourage various aspects of community- based horticulture through the Community, Private and Civic activities.
- To extend knowledge throughout the community of gardening, horticulture and its uses, and garden design through training, organisation and direct support.
- To educate the public in the protection and conservation of our natural resources.
- To contribute to, encourage and participate in activities assisting Botanic Gardens and other like-minded horticultural institutions.

Look on their website to see the large number of clubs throughout Western Sydney catering for all kinds of horticultural interests.

Home – Garden Clubs of Australia

https://gardenclubs.org.au/locate-a-club

Greater Sydney Landcare Network

The Greater Sydney Landcare Network (GSLN) aims to be the regional umbrella organization for Landcare, Bushcare and other 'care' groups and individuals across greater Sydney. GSLN wants to promote to the wider community the values of Landcare and Bushcare, and to increase the understanding, recognition and support for the work that land-caring volunteers, professional bush regenerators and related organisations do for the greater Sydney region. GSLN aims to be a bridge between professionals and non-professionals in the environmental field, and a voice for land-carers at a volunteer level.

The Network has a large number of opportunities for volunteers to help with important conservation and bushcare work. These are outlined in detail on the website.
https://greatersydneylandcare.org/home

Nature Play

Children are able to create their own adventures and experiences in nature with Nature Play activities at Western Sydney Parklands.

Lizard Log is a child's wonderland in a bushland setting. It has a nature-themed playground for the kids, scenic walks for all and cycling tracks of various lengths for those who like a physical challenge.

The sprawling nature playground was designed to blend in with the natural landscape while offering fun features. It has a climbing wall, slide, dual and single flying foxes, balancing beams, giant swings and a water play deck. The wood-carved animals that decorate the playground were made using timber salvaged from the area. Loop paths run through the site – perfect for scooter and bike riders.

Through nature play, children are encouraged to use their imagination and push their own mental and physical boundaries to grow. They get to leave their screens behind, get dirty and feel, smell, climb and touch nature.
www.westernsydneyparklands.com.au/places-to-go/lizard-log/

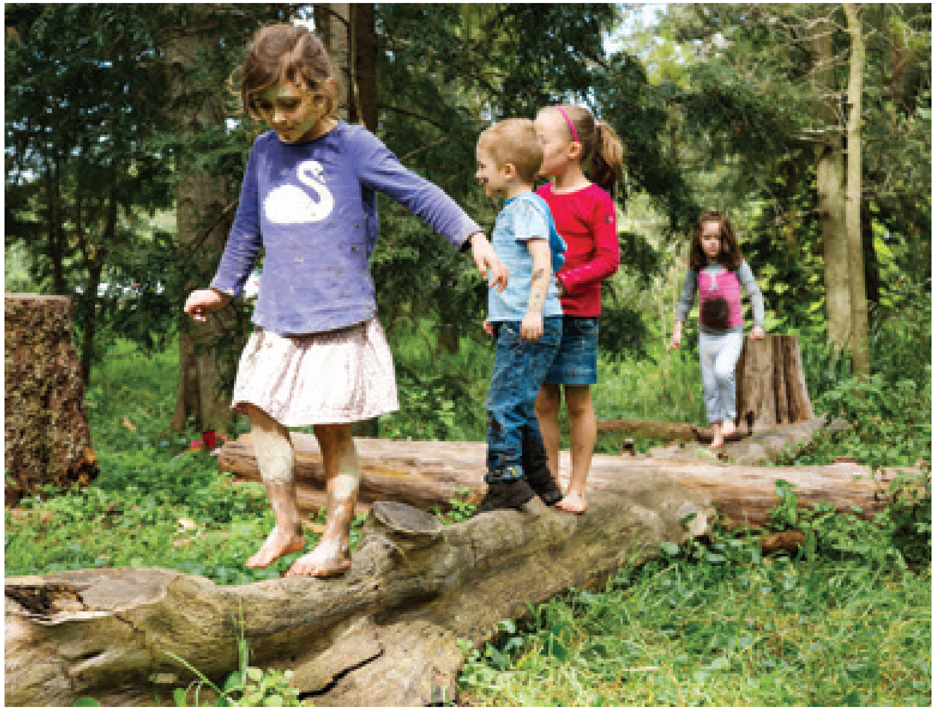

Courtesy of NaturePlay

Explore the trails and facilities at Lizard Log, and bring a downloaded copy of Western Sydney Parkland's Lizard Log Kids Trail Map to discover the beauty of this natural area on your next visit.

NSW Volunteering

This is a great chance for you to become involved in helping our local environment. If you want to meet new people, have fun, learn new skills, use your skills, build confidence and contribute in a positive way to your community, volunteering is a great place to start! You can choose what category or categories (such as animal welfare, conservation or recreation) that interests you and you'll have access to a large number of opportunities to help out.

Details: https://makeadifference.volunteering.nsw.gov.au

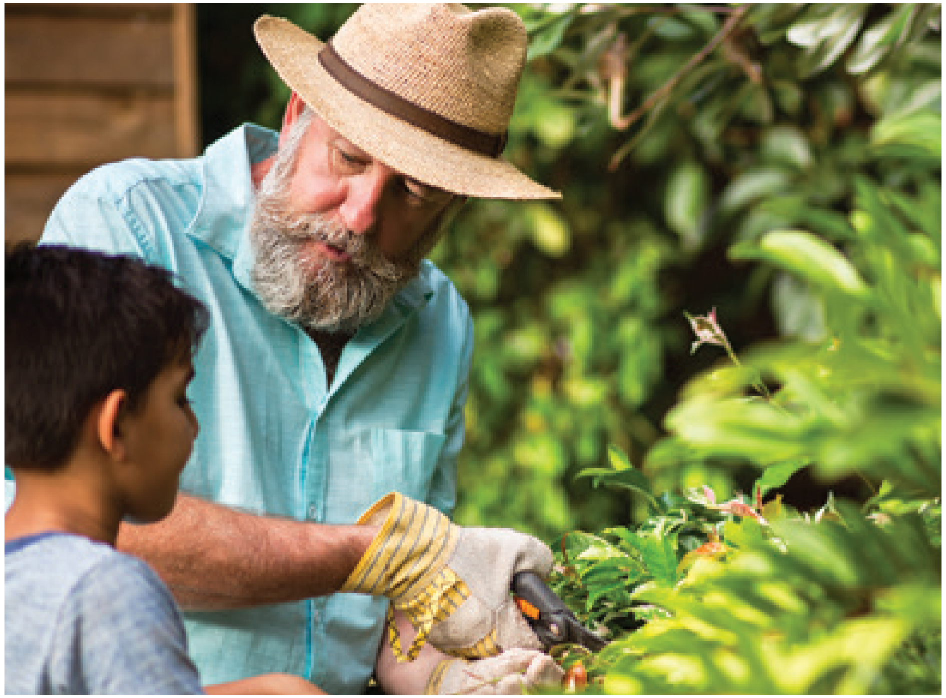

Penrith Lakes Environmental Education Centre (PLEEC)

Penrith Lakes Environmental Education Centre (PLEEC) is one of 25 Environmental and Zoo Education Centres operated by the NSW Department of Education.

PLEEC provides quality curriculum related fieldwork programs for students (K-12) across different Key Learning Areas. These engaging programs support students in achieving syllabus outcomes as well as in appreciating their application in the 'real world context'. In addition, PLEEC also provides programs that address sustainability as a cross curriculum priority of the new Australian Curriculum.

In 2014 PLEEC relocated to Sydney International Regatta Centre on a temporary lease agreement due to Penrith Lakes Development Corporation requesting back the founding site after 18 years of tenure.

Recently, great progress has been made towards establishing a new world class 'Centre of Excellence' in environmental education and sustainability, equipped with high-quality innovative facilities designed to best cater to our locally contextualised programs and outdoor learning experiences.

The new PLEEC facility will be 'future proofed' and will cater to more schools as the demand for authentic and outdoor 'real world' programs continues to grow across all curricula.

Details: Penrith Lakes Environmental Education Centre (penrithlakeseec.com)

Location: **Gate A, 153-233 Old Castlereagh Road Cranebrook NSW 2749**

Sydney Wildlife Rescue

Sydney Wildlife Rescue is a rescue organisation that is active in the greater Sydney Metropolitan Area.

We are a non-profit wildlife organisation operating under licence from the NSW National Parks and Wildlife Service. Our mission is to rescue and care for sick, injured, and orphaned native wildlife and to safely release them back into the wild.

As well as caring for sick, injured and orphaned native wildlife, Sydney Wildlife is also involved in educating the community about native wildlife and its habitat. From kindergartens to retirement villages, community education packages are tailored to meet the specific needs of a wide range of audiences.

You can be trained to become one of the carers, but there are many other ways you can help. You can be trained to answer our rescue phone.

This runs 24/7 but you can select shift times and it can be done from home. You can help with transport of animals and food to carers. You can use your skills in graphic arts/IT/public relations to help us.

If you are a handy person, you might like to help put aviaries and nest boxes together for us.

Let us know what you can do and we'll find a job for you!

Details: https://www.sydneywildlife.org.au/become-a-volunteer

https://www.sydneywildlife.org.au/

E: info@sydneywildlife.org.au

Phone: **(02) 9413 4300 24hrs**

Mail: **PO Box 78, Lindfield, NSW 2070**

WIRES

Continuing suburban development puts pressure on wildlife in our area as their habitat reduces. WIRES plays a very important role in rescuing and rehabilitating local wildlife.

WIRES is always in need of more volunteers to assist with wildlife rescue and rehabilitation. If you are interested in joining, please read the information about becoming a volunteer.

https://www.wires.org.au/rescue/become-a-rescuer

WIRES Wildlife rescue 1300 094 737

There are 2 local WIRES branches in western Sydney.

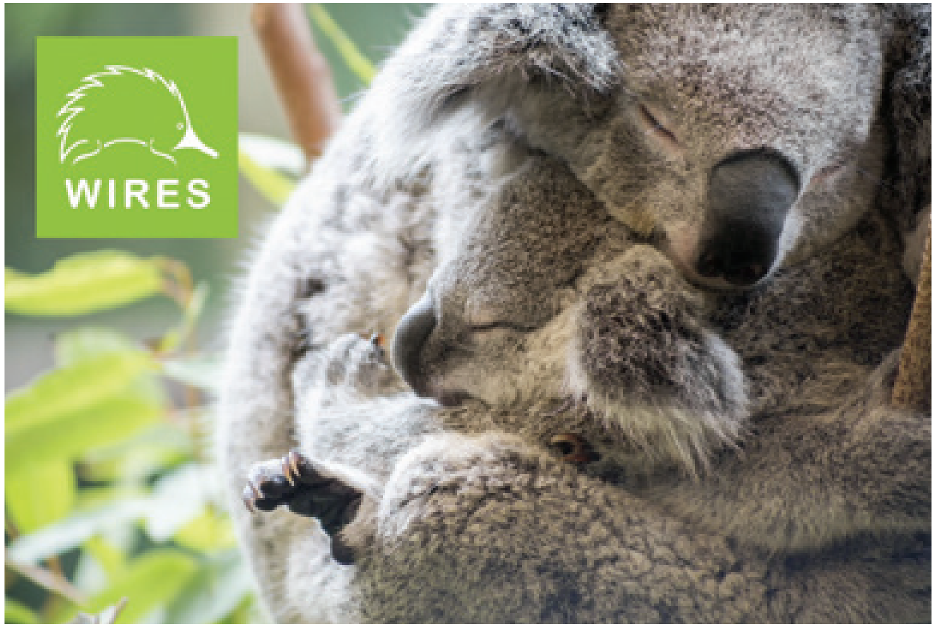

WIRES Cumberland Branch

Cumberland Branch encompasses a very large area covering parts of the Camden, Canterbury-Bankstown, Cumberland, Fairfield, Liverpool, Parramatta, Penrith, Sutherland Shire and Wollondilly local government areas.

WIRES Cumberland Branch services a very large area extending from Silverdale on our western edges to Rookwood in the east, north to Erskine Park and south to Sandy Point. Continuing suburban development puts pressure on wildlife in our area as their habitat reduces. We cover a very multicultural area of Sydney and perhaps a surprisingly broad range of native wildlife including koalas, kangaroos, bats, reptiles, birds and possums.

https://www.wires.org.au/branch/sydney-cumberland

WIRES North West Sydney Branch rescue wildlife in an area roughly bordered by the M4 in the south, the Northern Road in the west, and Old Northern Road in the east. Continuing suburban development puts pressure on our wildlife as their habitat reduces.

We rescue a wide variety of native animals: mostly birds, but also possums, reptiles, turtles and snakes and an occasional kangaroo, wallaby, echidna and quoll.

We meet on the third Monday of the month at Kings Langley Community Centre between 7:30 pm and 9:30 pm.

https://www.wires.org.au/branch/sydneynorthwest

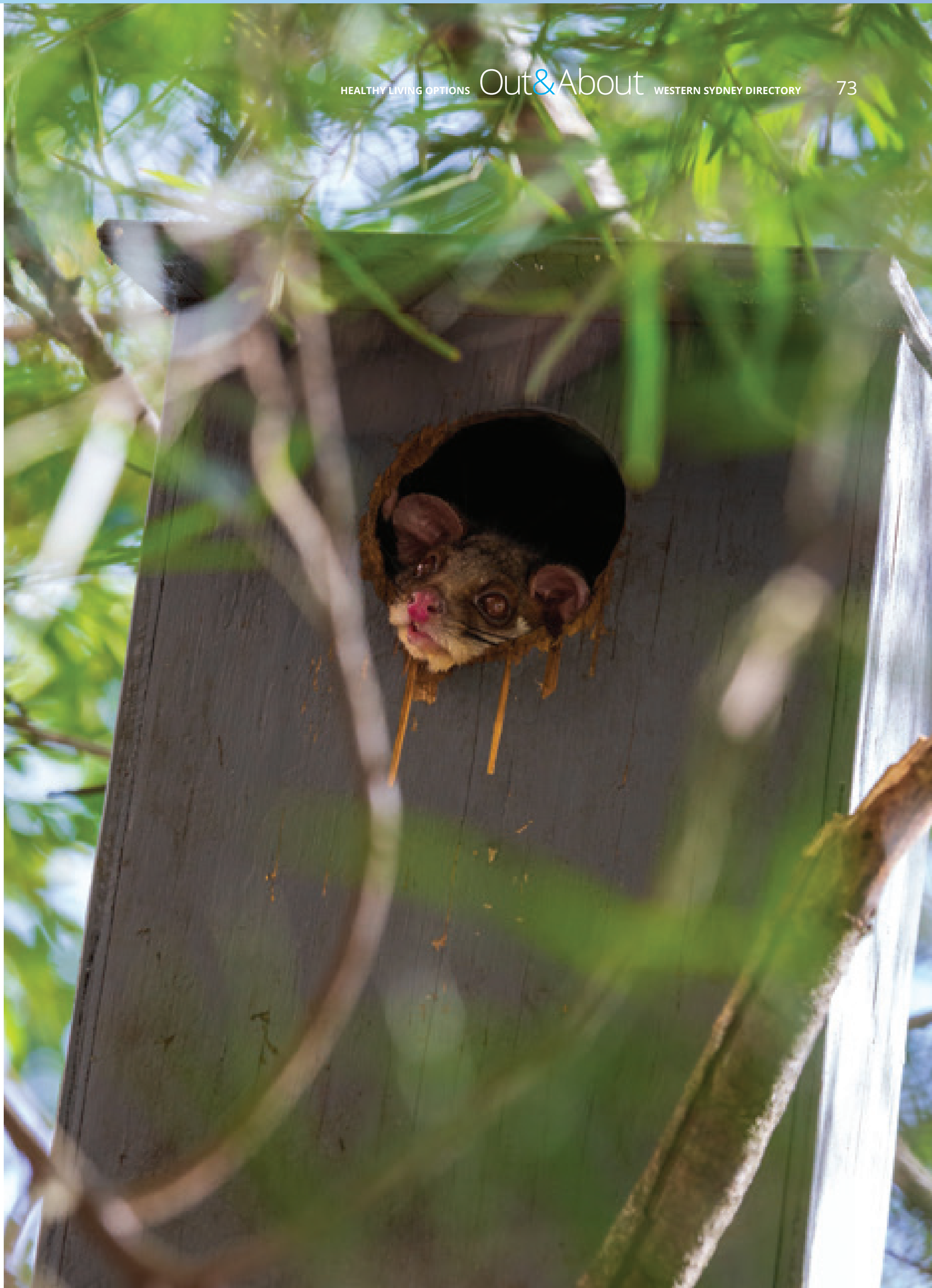

Western Sydney Diabetes
Beating Diabetes Together

Janine Dawson
Diabetes Prevention Program Manager

Telephone: (02) 8670 0015
Mobile: 0419 239 179
Fax: (02) 9851 6146
Email: janine.dawson@health.nsw.gov.au
Web: www.westernsydneydiabetes.com.au
Twitter: @wsdiabetes

The information provided in this publication is current as of March 2022. Please refer to the individual websites for the most current information and for any changes to products or services.

© July 2022

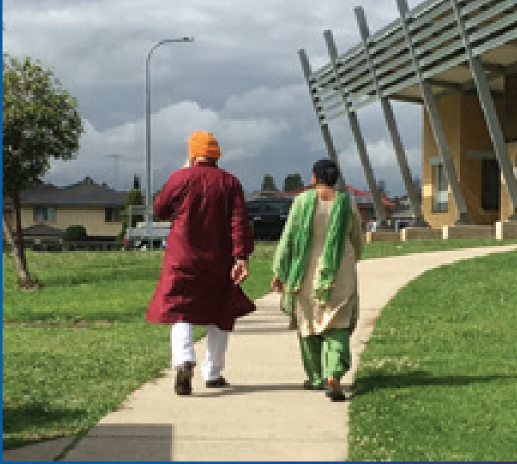

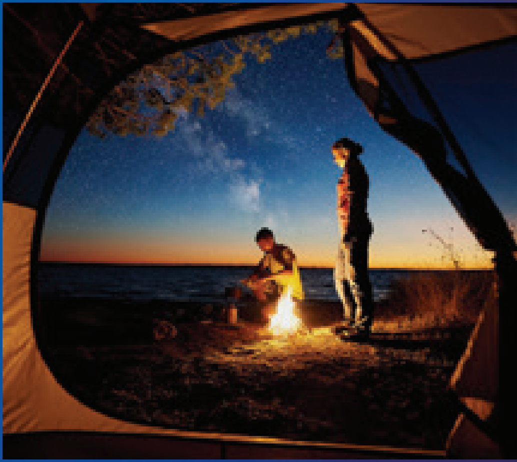

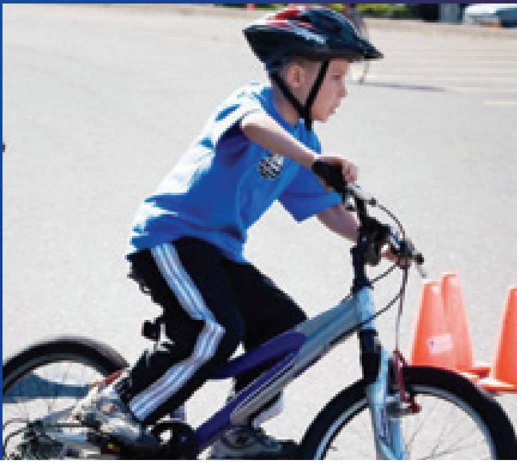

phn WESTERN SYDNEY
An Australian Government Initiative
pwc
diabetes nsw & act
NSW GOVERNMENT
