# Supplementary material for: Integrating point-of-care diabetes detection with lifestyle counselling in community settings: outcomes from Western Sydney, Australia
Source: BMC Health Serv Res. 2024 Aug 13;24:926. doi: 10.1186/s12913-024-11335-y (PMC11323375; doi:10.1186/s12913-024-11335-y)
Supplement: Supplementary file 6 — Supplementary Material 6 [file 12913_2024_11335_MOESM6_ESM.pdf]

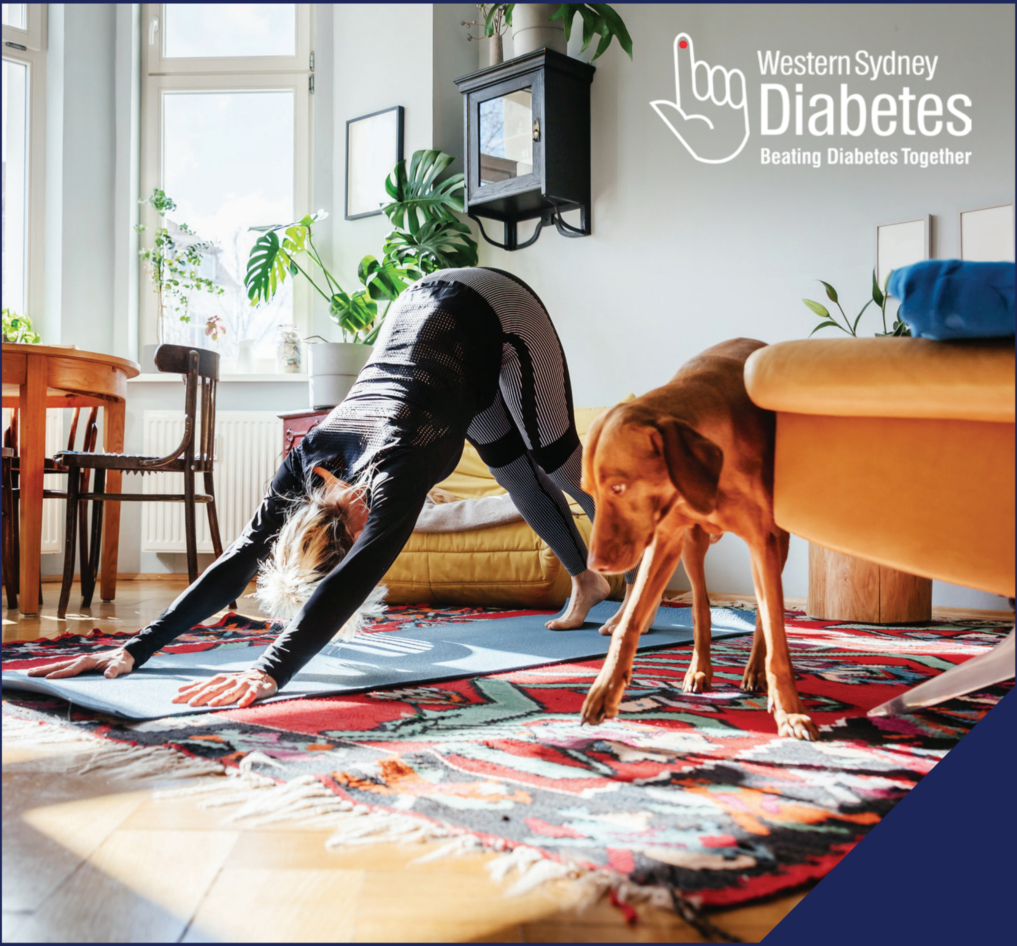

Western Sydney Diabetes
Beating Diabetes Together

# HEALTHY LIVING OPTIONS

## ONLINE EDITION

phn WESTERN SYDNEY
An Australian Government Initiative

diabetes australia

NSW GOVERNMENT

# About this booklet

At Western Sydney Diabetes we want all our residents to have the opportunity to lead happy healthy lives. But we realise that our everyday life often gets in the way. Family commitments, daily commutes, work, cost and availability often limit our ability to engage in healthy activities.

For this reason, we've produced this Healthy Living Options online version. We want to reduce as many health obstacles as possible. We've included all kinds of exercise classes, dietary assistance and mental health resources that you can access online at a time and place that suits you.

As with all the booklets in the Healthy Living Options series, we've tried to only include activities that are either free or very inexpensive. We've included options for all kinds of people, ages and fitness levels in the hope that everyone will find something that they would like to do that will enrich their lives.

We hope that you enjoy this booklet and share it with your friends and family.

Please visit our website for more information and copies of our Healthy Living Options, Affordable food, and Out & About booklets: https://westernsydneydiabetes.com.au/resources/brochures/

# Contents

**1 Free Online Exercises** **4**
Active At Home 5
Fitness Blender 5
Get Active Victoria 6
Live Life Get Active 7
MWH Method 8
NHS Fitness Studio 9
NIKE Training Club 9
NSW Active And Healthy 10
Parramatta Council Online Activities 11
Peloton 12
Personal Walking Plans 13
Popsugar Dance Fit 14
Sweat Fitness App 15
Tone It Up 16
Canstar – Top 10 Fitness Apps 17
Virtual Y 19
Yoga With Adriene 19

**2 Free Online Dancing Classes** **20**
Howcast 21
Learn To Dance 21
YouDance 22

**3 Looking After Your Mental Health** **23**
Beyond Blue 24
Free Meditation 24
Gardening Australia 25
Head To Health 25
HeadGear 26
Headspace 27
MindGuide 27
MindSpot 28
Smiling Mind 29

**4 Telephone Coaching Programs** **30**
Get Healthy 31
The COACH Programs 31

**5 Dietary Guidance** **32**
FoodSwitch app 33
No Money No Time 33

**6 Free online cooking classes** **34**
The Kitchn's Cooking School 35
Food Techniques 35
Dinner Tonight Texas A&M 35
Better Homes And Gardens 36
Delia Online Cookery School 36
Instructables 36

**7 Easy Dinners** **37**
Web Help For Non-Cook Dinners 38
Basic Healthy Dinners 38
Australian Healthy Food Guide Magazine 39

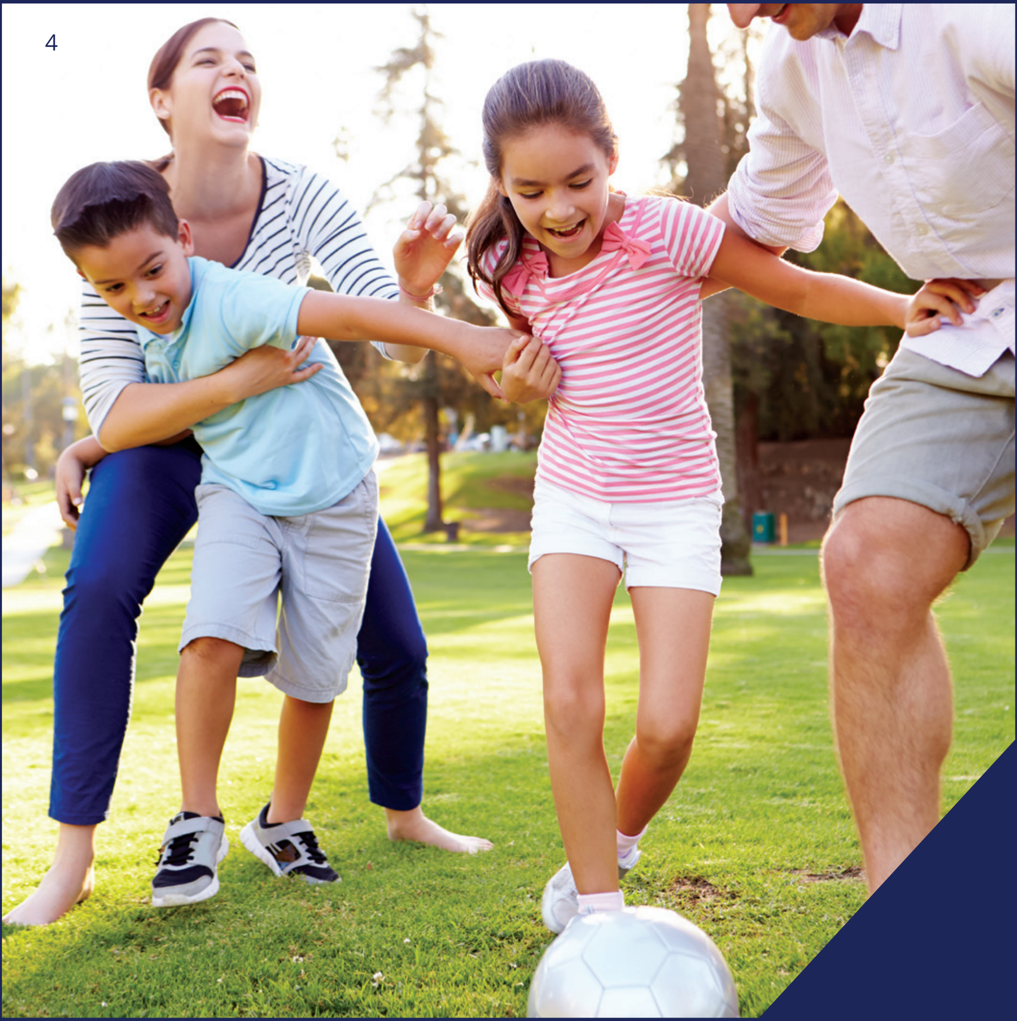

# 1 FREE ONLINE EXERCISES

# Active At Home

https://www.sportaus.gov.au/schools/active-at-home

With many schools impacted by the COVID-19 pandemic, sporting organisations put together a huge array of resources and online activities to support students at home. In most cases these resources are still highly useful and practical for both adults and children, including a huge variety of sports such as athletics, AFL, baseball, basketball, bowls, cricket, cycling, equestrian, football, golf, gymnastics, hockey, lacrosse, NRL, snow sports, softball, squash, Surf Life Saving, table tennis, tennis, tenpin bowling, triathlon, volleyball and water polo.

---

# Fitness Blender

Fitness Blender is a popular YouTube channel featuring over 600 workouts that can be searched by length, difficulty, training type, calories burned and muscle group. They have videos suitable for all fitness levels, as well as excellent options for when you might want a really short but intense workout. Over 500 **FREE** workouts range from 10-85 minutes long, from beginner level to elite athlete, from HIIT to Pilates, and strength training to bodyweight.

Fitness Blender believe reliable fitness, nutrition, and wellness content should be accessible to everyone, regardless of income. You can also pay a small membership fee to join up and receive access to full length workout programs, challenges and meal plans.

Visit the website: **https://www.fitnessblender.com/page/about-fitness-blender**

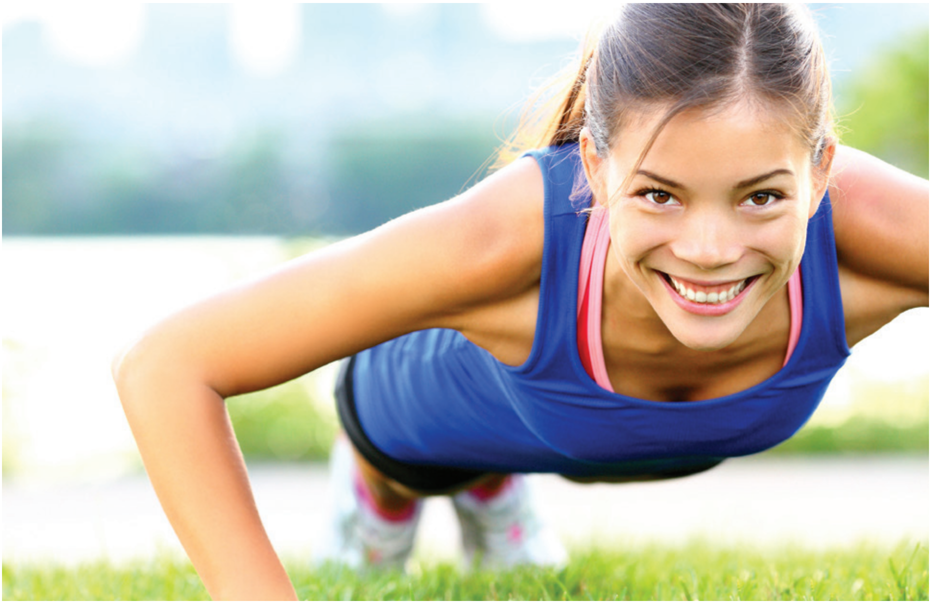

# Get Active Victoria

https://www.getactive.vic.gov.au/around-home/workouts/

Get Active Victoria is helping people make positive changes to their health and wellbeing. It's **FREE** to join and gives access to a huge resource of workout videos, educational tips and interactive tools to assist you to be more physically active in a way that suits you.

The Get Active Victoria website is full of useful ideas for getting active along with expert advice. When you join Get Active Victoria, you unlock **FREE** tools to help you be active and a huge library of guided home workouts in addition to joining a community of more than 99,000 people all using Get Active Victoria to inspire more active choices.

Exercise classes include a large number of cardio, core, stretching, dance, pilates & yoga, strength and low impact workouts. You need to join up and then can access all these excellent resources.

# Live Life Get Active

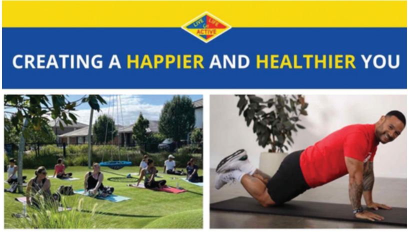


Live Life Get Active is all about helping you become the person you want to be: someone with good physical, emotional, nutritional, and social health. We work in communities right across Australia, but one of our first partnerships was with Blacktown Council, and we are still here today.

We started by offering **FREE** outdoor activity camps—they run across several communities (check out our locations) **https://livelifegetactive.com/locations/**

and they are really popular. Then, as a result of COVID, we added a comprehensive online offering. This offering is made up of LIVE sessions where you still workout with a group, and we offer a full library of on-demand videos that you can access whenever you want.

**Want to see what it's like?** You can. We offer new people access to five different videos before we ask them to become members. So, you can try morning meditations, belly-busting workouts, wind-down yoga, or one of our energy-boosting nutritional videos. If you like what you see and want more, then we ask you to join.

**What does that involve?** We ask you to complete a pretty comprehensive application form. It takes about 10 minutes to complete. We ask you lots of things about how you feel, your health, and your current behaviours. Why do we ask this? Because we want to provide you with information to really help you feel happier and healthier.

**What do I get as a member?** You will be sent your own personalised nutrition plan. This will show you how to eat to feel great. Our approach to nutrition is not restrictive; it's about giving you energy, making you strong, and supporting a positive outlook on life.

You will get access to our full library of videos covering physical, emotional, nutritional, and social health.

## Live Life Get Active (continued)

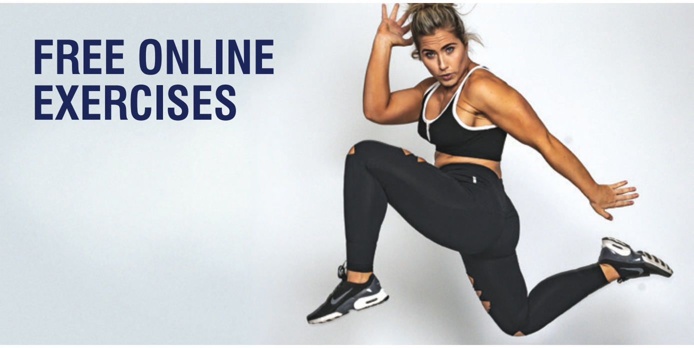


You will get access to 26 LIVE classes each week, offering a diverse range of activities to explore. From energetic aerobics and cardio boxercise to restoring yoga and mind-body tai chi, there's something for everyone. These live classes run throughout the day, seven days a week.

You will get access to a program that will help you understand and cope with anxiety. It's an easy-to-understand program that helps you cope.

You will be able to join programs run by our team of health professionals that discuss everything from body image to better sleep patterns and improved physical and mental energy. They are very popular.

You have nothing to lose and so much to gain because we are always here to support you, and absolutely everything we do is **FREE**!

We encourage you to explore and enjoy **www.livelifegetactive.com**

# MWH Method

Suitable for people with limited space, Melissa Wood Tepperberg's classes involve small and precise movements that can be done anywhere. She offers 500+ workouts meditations, pilates, yoga and more. The first week of workouts (available online and in the App Store) are **FREE**. After that the program is $USD14.95 per month.

**https://melissawoodhealth.com/**

# NHS Fitness Studio

Take your pick from 23 instructor-led videos across our aerobics exercise, strength and resistance, and pilates and yoga categories.

These workouts have been created by fitness experts InstructorLive and range from 10 to 45 minutes.

There are exercises to tone your abs, raise your heart rate, and tone your upper arms. You will also find workouts suitable for new mums, such as postnatal yoga, or health problems, such as pilates for back pain.

Plus, there's the Wake up! workout, Vinyasa flow yoga, and Belly dancing for beginners to get you moving.

For more **FREE** exercise ideas, check out our Strength and Flex exercise plan.

Visit the website: **https://www.nhs.uk/conditions/nhs-fitness-studio**

---

# NIKE Training Club

Reach your fitness goals with the Nike Training Club App (NTC). With access to 200+ **FREE** workouts and targeted training programmes, the NTC App will support you every step of the way. From invigorating yoga classes to bodyweight sessions with and without equipment, you'll find the right workout for you.

Follow our Nike Master Trainers through varied classes, ranging from 5-50 minutes. Do them at home, in the gym, or outdoors – whenever and wherever you want. Plus, our **FREE** video library is constantly growing, so expect new videos to flow in.

No matter what fitness level you're at, NTC will give you the tools you need to succeed.

Visit the website: **https://www.nike.com/au/ntc-app**

# NSW Active And Healthy

Active and Healthy is an online directory of exercise programs within NSW for people 50 years and older of varying fitness levels. Searches on the website produce a number of exercise programs located near your chosen suburb/town and can be refined to display specific types of exercise.

Some of the programs include Tai Chi, qigong, falls prevention, cycling, gentle exercise, strength and resistance training, yoga, pilates, seniors fitness, aqua fitness, line dancing and zumba.

The website is easy to use and provides a large range of exercise options with contact details, maps, addresses, class times and fees. The search results can also be printed with the class name and contact details displayed. Because there is such a large range and class details change regularly, we suggest that you contact the program provider to confirm the class details.

Visit the website: **https://www.activeandhealthy.nsw.gov.au**

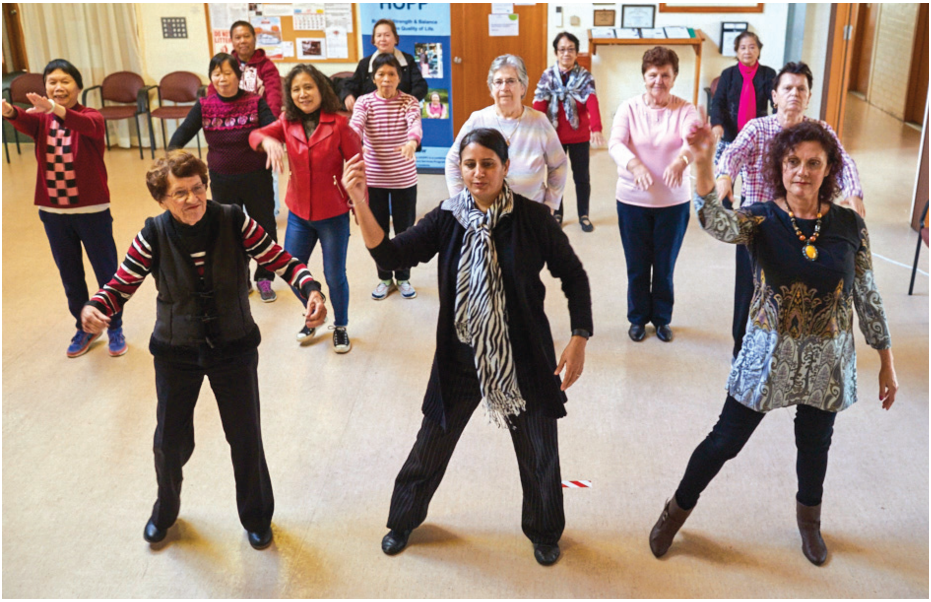

# Parramatta City Council Online Activities

Active Parramatta Online is dedicated to delivering a variety of high-quality programs and resources online to support the health and wellbeing of residents, workers and visitors of Parramatta.

https://www.cityofparramatta.nsw.gov.au/recreation/active-parramatta/active-parramatta-programs

Registrations to a variety of fitness activities are open all year, with classes suitable for many levels of fitness and wellbeing goals. The cost is very reasonable at about $30-40 per term. The current online programs include:

**Bodyweight training** (also called calisthenics) involves no equipment, but rather uses a number of exercises that achieve full body workout by moving yourself. These classes will involve a wide range of exercises that can cater for a beginner, up to very advanced trainers. Developing core strength is a key aspect of bodyweight training. Almost all exercises are low impact.

**Yogalates** is a method of exercise that fuses the practices of yoga and Pilates. The exercises cultivate strength, stamina, stability and flexibility with particular attention paid to building tone in deeper postural "core" muscles.

Yogalates is suitable for all ages and275urages a connection with the self through breathing techniques to soothe stress and aid relaxation, instil inner calm and overall wellbeing. This class is for those at the beginner/intermediate level.

**Yoga** cultivates health and wellbeing (physical, emotional, mental and social) through the regular practice of a range of many different techniques, including postures and movement, breath awareness and breathing exercises, relaxation and concentration, self-inquiry and meditation.

Yoga is an approach to life that values appropriate effort, based on balance and harmony, within each person and with each other. The class is perfect for those who are beginners or new to yoga

**Pilates** caters for everyone, from beginner to advanced. You can perform exercises using your own body weight, or with the help of various pieces of equipment. A typical Pilates workout includes a number of exercises and stretches. Each exercise is performed with attention to proper breathing techniques and abdominal muscle control.

## Parramatta City Council Online Activities (continued)

Pilates lengthens and stretches all the major muscle groups in the body in a balanced fashion. It improves flexibility, strength, balance and body awareness.

**Core & Stretch** is a Pilates-inspired workout with lots of standing and balance work, using core stability and conditioning to help improve hip, legs and shoulder stability. This will help people with bad knees or hip movements. Stretches can be done in standing or on chair positions whilst working their core stabiliser muscles. This class is ideal for people that sit or need to work at a desk for long hours.

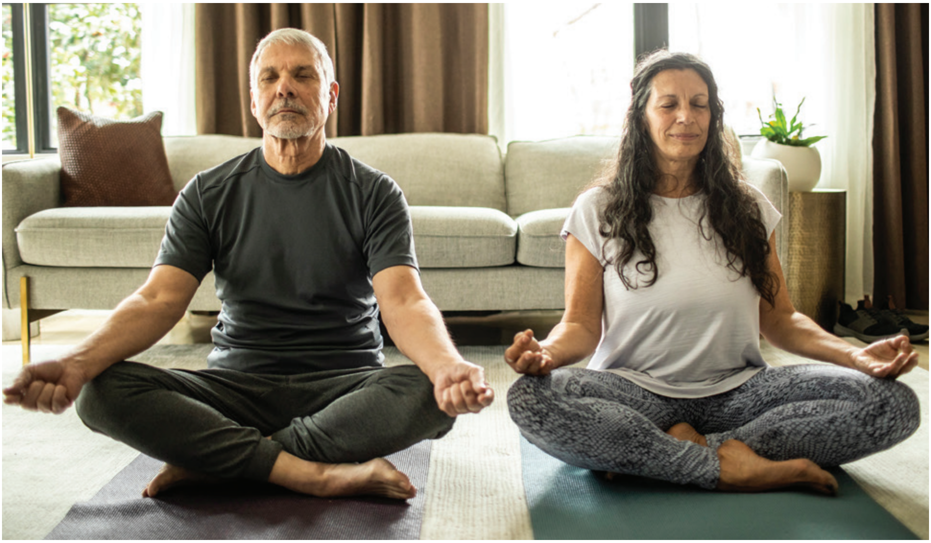

# Peloton

https://www.onepeloton.com.au/app

Whether or not you have a Peloton bike or treadmill, you can access the Peloton app for its running and spinning workouts. Peloton's motivating instructors (many of whom have huge followings) lead live-streamed classes where users can virtually high-five other participants. The Peloton app also offers bootcamps, guided runs, yoga and strength training routines. Peloton is offering its app (available in the App Store, Google Play and Amazon Appstore) for **FREE** for 60 days. After that, it's $USD16.99-$35/mth.

# Personal Walking Plans

Discover the Heart Foundation's Personal Walking Plan program designed for anyone wanting to start or improve their weekly exercise habits. Tailored to meet your current fitness levels, this program spans six weeks and sets out your plan for walking, strength exercises, and stretching routines. Created by experts in the field, it provides more than just technical instructions – it aims to make your fitness journey enjoyable while helping you cultivate a lasting habit.

Throughout the program, you'll experience a gradual increase in confidence, strength, and flexibility, leaving you with a profound sense of accomplishment!

To support you every step of the way, you'll receive a personalised weekly plan delivered straight to your email inbox. In addition, we'll send you valuable tips via SMS, and you can reach out with any questions you may have. Plus, gain exclusive access to our vibrant online community, where participants and like-minded individuals gather to share their walking experiences.

In a recent survey, an impressive 99 percent of participants expressed their commitment to sustaining regular walking or engaging in physical activity even after completing the program. Don't miss out on this incredible opportunity to improve your walking habits and consistency. Take the first step today and sign up now!

1. **Complete the 2-minute form** – We'll work out the right plan for you based on your current level of activity.
2. **Get your plan** – We'll send your plan directly to your email.
3. **Get moving and feel the benefits** – Over six weeks, we'll send information and advice to your email and mobile to help you stick with it.

For more information visit **http://walking.heartfoundation.org.au**, call the Heart Foundation on **13 11 12**, or email **walking@heartfoundation.org.au**

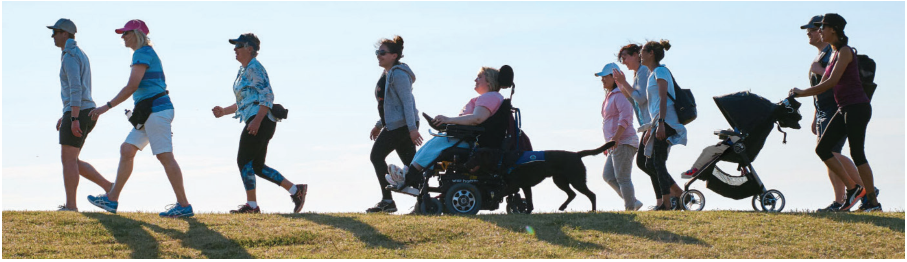

# Popsugar Dance Fit

https://www.youtube.com/playlist?list=PLI37FJmOtrj1R0fTaqRH6XH5BKEudl0Jq

Dance into your fitness goals with high energy, cardio dance workouts. Running for 3 to 30 minutes, you will have a wonderful choice of dance styles. These include Hip-Hop, Latin dance, Cardio dance, Bollywood, Salsa PopFusion and Jazz-dance. You can even learn to Twerk!

New episodes roll out every other Wednesday on the POPSUGAR Fitness YouTube channel.

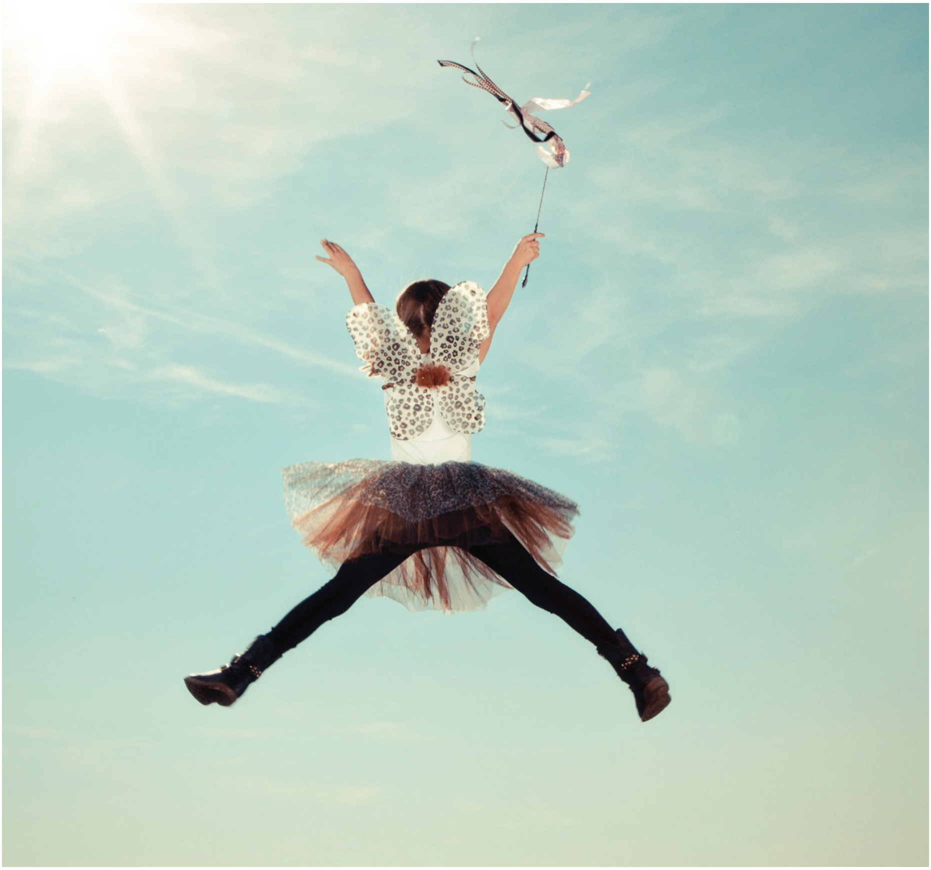

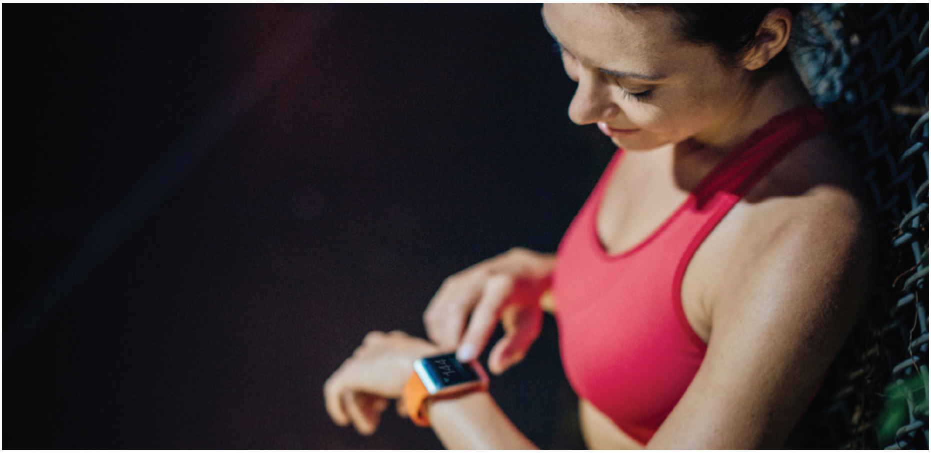

# Sweat Fitness App

https://www.sweat.com/

Train at home or in the gym with one of the world's largest women's fitness communities! Get the fitness motivation you need with Sweat, a personal training app featuring co-founder and elite head trainer, Kayla Itsines.

Sweat offers a broad suite of workouts you can do anywhere, anytime to progressively build your fitness. New programs give you more ways to push yourself and get you closer to reaching your goals. Stay accountable with virtual community challenges and feel supported by like-minded women.

Find a training style you enjoy with easy-to-follow beginner, intermediate and advanced workouts, including: HIIT (High-intensity interval training) – Circuit Training- Bodyweight exercises – Powerbuilding – Strength training – Yoga – Barre – Pilates – Recovery – Cardio- Pregnancy and postnatal.

Train your way — follow a program or work out on demand. Choose workouts that suit your lifestyle with no equipment, **FREE** weights or machines and express workouts when you are short on time.

Sweat's features make it simple to stay on track and keep on top of your progress.

Sweat is **FREE** to download. Ongoing use requires an active subscription, available on a monthly or yearly basis. New customers who choose a monthly subscription are eligible for a **FREE** trial period.

# Tone It Up App

https://my.toneitup.com/pages/tone-it-up-app

Uplifting trainers Katrina Scott and Karena Dawn host live classes and have a library of hundreds of workouts that range from 10 to 40 minutes. Their goal is to help you boost metabolism and tone up. You can do plenty of their efficient workouts with just a yoga mat. You'll be able to do even more with a dumbbell and resistance band. Tone It Up (available in the App Store) is now **FREE** until April 13 to 'has a **FREE** 7 day trial. After that, it's $USD14.99/mth.

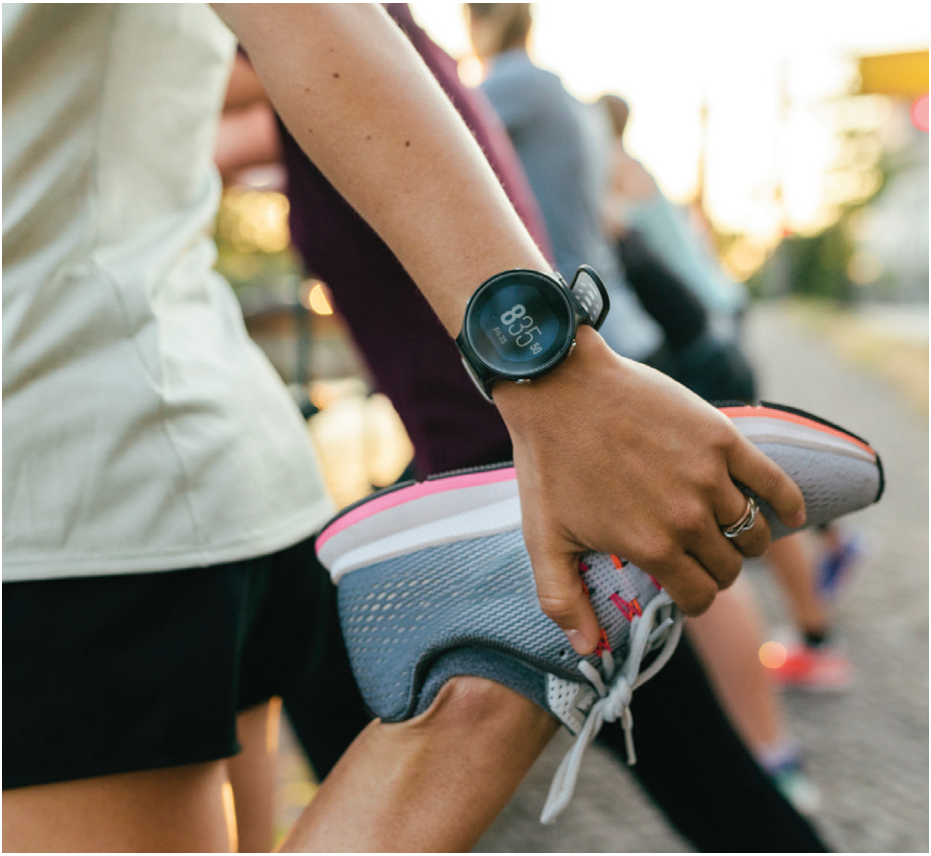

# Canstar – Top 10 Fitness Apps

https://canstar.com.au/health-insurance/workout-apps/

There are more than 250,000 fitness apps available, all vying for our attention. Canstar has done the heavy lifting and compiled a list and description of the workout apps that are currently topping the Google Health & Fitness chart. Here are some of them:

### MyFitnessPal

A food and fitness tracker, this app offers a range of workout routines for hi-intensity interval training to yoga and cardio. You can also link your account to smart wearable device apps. This app will allow you to track your meals either manually or by using the inbuilt barcode scanner to gain insights into the calorie and nutritional content.

It's currently **FREE** to download and also provides a premium subscription service which offers a one-month **FREE** trial.

### Fitbit

This app was designed to accompany the Fitbit wearable. It has been designed to encompass all parts of your day and can track your activity, workouts, sleep, nutrition and stress. This app also offers video and audio workouts that you can do at home including HIIT, cardio, strength and yoga. It is **FREE** to download and you can subscribe to become a premium member.

### Strava: Run, Ride, Hike

The Strava app allows you to track a range of activities and share them to their network. It has over 30 activities you can record including foot sports, water sports along with other a whole lot of other more unusual activities. It can track and analyse stats such as distance, heart rate, speed and calories burnt. You can connect with friends, become involved in challenges and leaderboards and have live GPS tracking. It is **FREE** to download and you can also purchase a subscription with more features.

### Centr

With this app you can train, eat and improve your wellbeing with the help of Chris Hemsworth and his team of trainers, chefs and meditation experts. Centr offers a range of exercise programs that can either be done in the home or in the gym. The app also offers daily workout activities such as HIIT, boxing, pilates or yoga. This app has a 7 day **FREE** trial and then a subscription is currently $29.99 per month.

# Canstar – Top 10 Fitness Apps (continued)

## Sweat: Fitness App for Women

The app offers multiple programs created and hosted by well-known personal trainers and fitness/health influencers from HIIT and circuit training workouts to yoga and barre

## All Trails: Hike, Bike & Run

All Trails focuses on nature and the outdoors, allowing you to plan, record and share your outdoor activities and exercises. It caters for all kinds of activities such as hiking, mountain biking, trail running, backpacking and rock climbing. You can find trails in your area and narrow your search down to specific requirements such as pet-friendly.

## BetterMe: Health Coaching

This is a fitness and meal planning app with an emphasis on a holistic approach. It offers workout programs, meal planning and progress tracking including water intake and step count.

## Muscle Booster Workout Planner

This app is tailored to men seeking to build strength and feel healthy. It provides you with a tailored exercise plan based on your goals and personal data.

## FitCoach: Fitness Coach & Diet

This app is for those who want to start a new active lifestyle, build healthy habit, lose weight and get fit without the gym. It includes workout and exercise routines such as yoga, pilates and HIIT, progress tracking, meal plans and recipes.

## Yoga-Go: Yoga for Weight Loss

This is a yoga-specific fitness app for at-home use but also includes other exercises such as pilates. There are 3 levels of difficulty and you can track your progress. You have personalised workout programs based on your goals and preferences.

# Virtual Y

https://virtualy.ymca.org.au/

virtualy@ymca.org.au

Welcome to Virtual Y – an online youth hub! We are an online platform brought to you by the Y.

On Virtual Y, you'll be able to access exclusive fitness, nutrition, wellbeing, family and youth content. From keeping fit through workouts and training at home and learning all about nutrition with our delicious recipes and Healthy Living Magazine. Not only does this youth health hub focus on physical health, but also mental – offering methods of looking after your wellbeing with mindfulness practices.

Online fitness events and classes include strength and cardio training ranging from beginners to the very fit, high intensity training, yoga & meditation, dance, Pilates and stretch & mobility training.

This is a great resource, especially for people under 25.

---

# Yoga With Adriene

https://yogawithadriene.com/

On a mission to make tools for mental, emotional, and physical health accessible for all, Adriene Mishler hosts the YouTube channel Yoga With Adriene, an online community of over 12 million subscribers. Yoga With Adriene provides high quality practices on yoga and mindfulness at no cost to support and inspire people of all ages, ethnicities, and backgrounds across the globe. She has a large range of videos, from long morning routines to a 10 minute session and quick, post-run stretches and movements for neck pain. The instructor is down to earth and easy to follow, often accompanied by her chilled dog Benji.

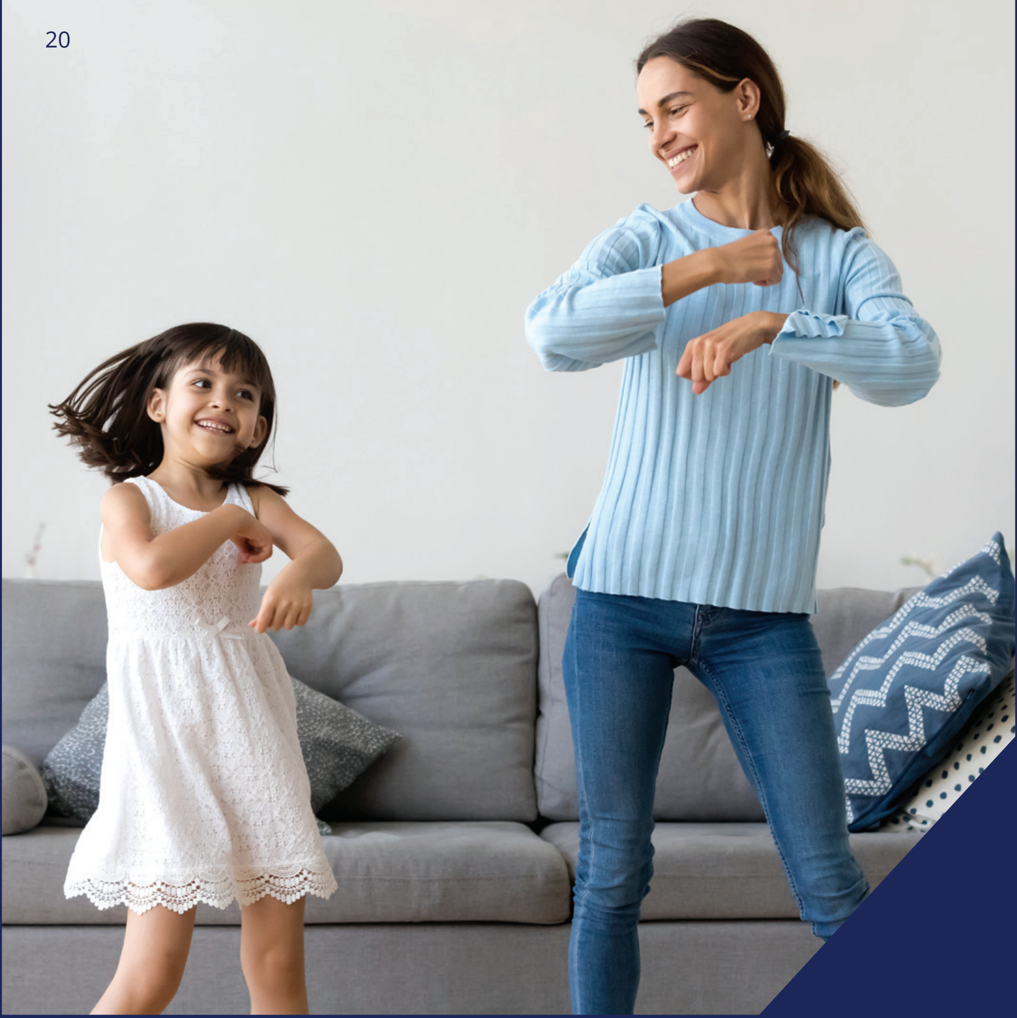

# 2 FREE ONLINE DANCING CLASSES

# Howcast

www.howcast.com

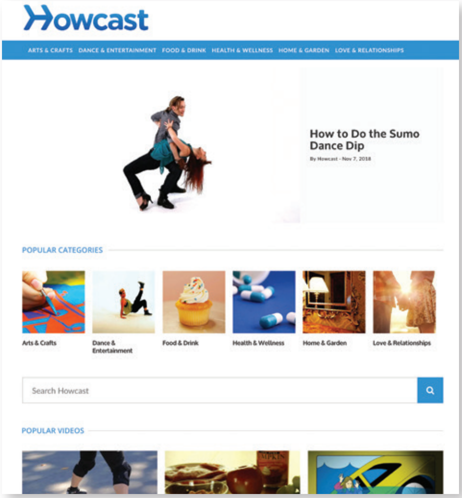


This website offers a very extensive range of videos giving instruction on a large number of hobbies, including numerous dance styles and dancing skills. Instructional videos cover everything from etiquette on the dance floor, to how to dance like Michael Jackson. The videos are **FREE** and due to the broad range, suitable for dancers of all levels.

# Learn To Dance

www.learntodance.com

Two left feet? Now is the time to learn to dance in the privacy of your own home. As well as being a super way to exercise for weight loss, fitness and muscle tone, dancing also makes us happy.

This website provides **FREE** online dance classes for over 10 styles. It will provide you with demonstrations on the basics of ballroom and Latin, Hip hop, Salsa, Ballet, Club dancing, tap dance, freestyle, breakdancing, Irish step dance, line dancing, country western, Argentine tango and even belly dancing!

After trying and practising the basics, you may want to continue learning a particular style. In that case, you can pay to download up to 50 instructional videos or purchase DVDs. One-on-one coaching is also available for an additional cost.

# YouDance

https://youdance.com/

At YouDance.com, the mission is to create a more convenient, fun and affordable way to learn to dance. When you enrol, you will get unlimited access to all the content which includes over 1,000 videos for you to learn your chosen dance style step-by-step.

You will have access to 7 different dance genres: Hip Hop, Ballet, Contemporary, Clogging, Jazz, Kids Dance Along and Dance for Exercise. The videos range from 2-20 minutes.

YouDance is suitable for all levels of dancers for people aged from 2 years upwards. Each dance lesson on YouDance.com ends with a quiz. Take it, pass it and you'll know that you're on the right track.

It can be streamed through your TV with chromecast, Apple TV, Xbox, Playstation and Roku. You can also view on your laptop, tablet or smartphone. There is a **FREE** trial for 7 days and after that it costs $15 US per month.

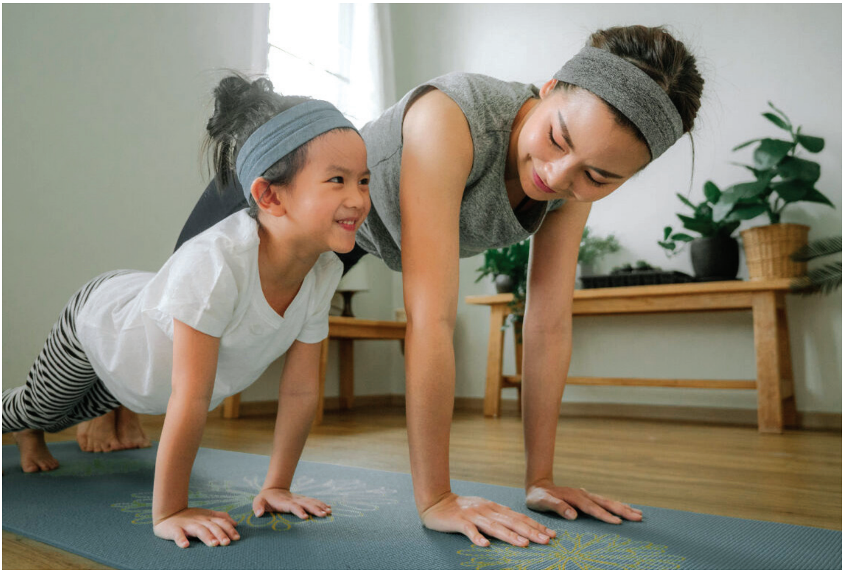

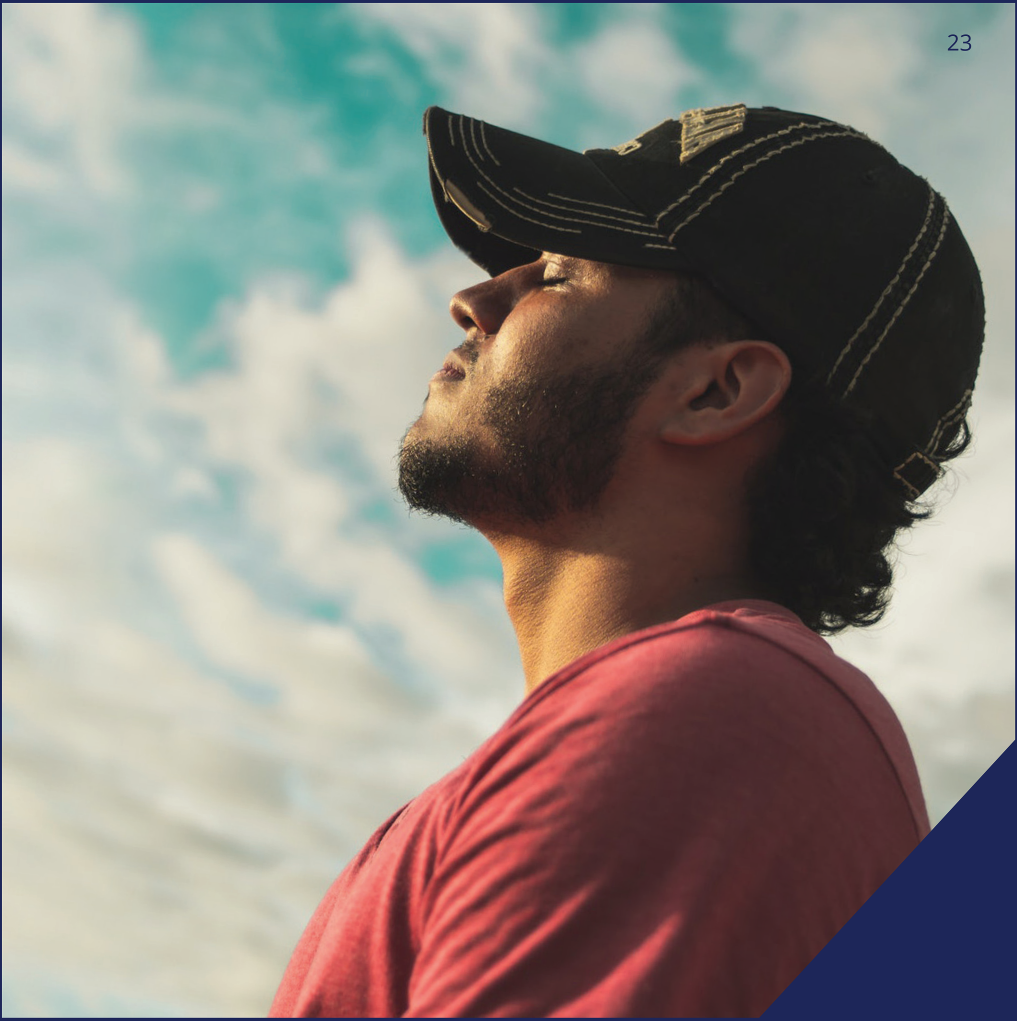

# 3 LOOKING AFTER YOUR MENTAL HEALTH

# Beyond Blue

www.beyondblue.org.au

**Support line 1300 224636**

Beyond Blue aims to provide information and support to help everyone in Australia achieve their best possible mental health, whatever their age and wherever they live.

Their website currently has an excellent section entitled 'Looking after your mental health during the coronavirus outbreak'. This provides practical and useful information on mental health, along with useful links where you can find further information on health and welfare from a wealth of sources. You can also join in on forums relating to COVID-19.

There is also a very large range of outstanding articles on achieving your personal best. These encompass a variety of topics that are especially useful during these uncertain times. They are as varied as stress busting activities to help you relax, meditation, self-care, how to make your space more mentally healthy and even desk yoga poses. Beyond Blue also provide access for you to chat online, email for a response, or to join forums.

# Free Meditation

https://www.freemeditation.com.au/

Millions around the world are enjoying **FREE** meditation. Meditation promotes mental, emotional, physical and spiritual wellbeing. Follow a guided meditation to experience inner peace and silence. It takes only a few minutes and really works! You can sit on a chair or on the floor with your shoes removed.

# Gardening Australia

https://www.abc.net.au/gardening

Gardening is a wonderful hobby which has multiple benefits for both your body and mind. Wherever you live, now is the time you might like to begin gardening as a new hobby, or else put some time into creating or working in your garden. You may enjoy trying your hand at growing some indoor plants, creating a balcony garden or giving your garden a long awaited tidy up.

Gardening Australia has a truly extraordinary set of resources that will provide you with a wealth of information on how to make and maintain any kind of garden. It has fact sheets on all kinds of garden activities including Tips/Tools/Techniques, growing in pots and garden design.

An extensive plant finder will give you photos and information on a huge number of native and introduced species of flowers, shrubs and trees. A vegie guide will help you set up a productive, healthy vegie garden full of all kinds of edible plants and fruits.

If you need more inspiration, they even have a space where readers can show their gardens, plants and DIY projects.

---

# Head To Health

https://headtohealth.gov.au/about-us

Head to Health can help you find digital mental health services from some of Australia's most trusted mental health organisations. Provided by the Australian Department of Health, Head to Health brings together apps, online programs, online forums and phone services, as well as a range of digital information resources.

Whether you are trying to improve your own sense of wellbeing or helping someone you care about, Head to Health is a good place to start. Resources include:

- Evidence-based apps and online programs that can help you build personal skills and track progress
- Mental health websites created by trusted organisations that give you detailed information and advice on specific mental health topics.
- Phone, chat, and email services, if you're looking for confidential, professional support
- Dedicated online forums and other peer support services where you can talk to people with similar experiences

# HeadGear

https://www.blackdoginstitute.org.au/resources-support/digital-tools-apps/headgear/

HeadGear is a **FREE**, easy-to-use smartphone app that guides you through a 30–day mental fitness challenge designed to build resilience and wellbeing and prevent things like depression and anxiety.

Based on techniques scientifically proven to build good mental health, it features a range of simple engaging daily activities to help reduce and manage stress, improve sleep, connect better with friends and deal with difficult situations.

### How does it work?

HeadGear is based on recognised and evidence-based psychological approaches including behavioural activation and mindfulness.

Once downloaded, users are guided through daily activities and challenges (5-10 minutes per day) for 30 days. These challenges include a variety of proven techniques used to develop mental fitness and resilience. Activities cover topics such as:

- Relaxation, mindfulness and breathing exercises
- Coping strategies to help manage life's challenges and reduce stress
- Being supportive to mates going through tough times
- Getting active and staying healthy
- Improving sleep
- Building meaningful connections

HeadGear is also personalised. You'll get to shape your own set of actions for building your mental health and wellbeing.

### Who's it for?

HeadGear has been designed with a male audience in mind, however, is appropriate for anyone who would like to build good mental health, is aged 18 and above and can read English with ease.

# Headspace

www.headspace.com

Headspace is an organisation dedicated to the mental health of young people. They have an excellent app relating to incorporating mindfulness into your everyday life.

Mindfulness is the ability to be fully present in the moment and can have numerous benefits, from decreasing stress to increased levels of focus and happiness.

Mindfulness is a way of living in which you are able to step back and be in the present moment in any situation. It doesn't eliminate stress, but gives you a choice with how to deal with difficult situations.

If you're looking for a beginner's guide to mindfulness, Headspace offers a 10-day beginner's course on the essentials of meditation that will help you develop mindfulness – available for **FREE** – which is an ideal way to start building the foundation for a life-changing daily meditation practice.

Headspace also offers guided meditations for stress, anxiety, sleep and more, as well as movement exercises. Once you have learnt the basics, you may want to sign-up for Headspace Plus, which for a small fee will send a new daily meditation to your phone.

Headspace has also added an excellent section to their website entitled 'Coping with stress related to COVID-19' specifically for 12-25 year olds.

# MindGuide

https://mindguide.org.au/

MindGuide has been commissioned by WentWest, Western Sydney Primary Health Network to provide guidance regarding mental health to people living or working in the Western Sydney Region. It is a wonderful source of material on directories, resources, information, networks and advocacy.

For more information contact WentWest on **02 8811 7100**

# MindSpot

https://www.mindspot.org.au

Access **FREE**, online, personalised mental health care with MindSpot.

Funded by the Australian Government, and proudly operated by MQ Health, Macquarie University, MindSpot has provided **FREE** mental health services to adults across Australia since 2012.

Our services include confidential psychological assessments and clinically proven treatments. MindSpot services are designed to reduce barriers to mental health care.

Our aim is to support people to learn more about their psychological health and learn skills to become emotionally resilient.

We provide care to Australian adults troubled by conditions like depression, anxiety, stress, worry, panic, OCD and chronic pain and distress due to chronic health conditions.

You can choose online or teletherapy sessions with a MindSpot Therapist.

For online programs, there is a 4 step process:

1. **Assessment**
2. **Feedback**
3. **Consultation Schedule** a confidential appointment to speak to a MindSpot therapist about your results and about treatment options.
4. **Treatment Enrol** and start an online treatment course to learn to manage your symptoms. You can choose to speak to a MindSpot therapist each week to help guide you through the course.

Each lesson comes with a workbook that helps you practice strategies and skills in your everyday life. The workbook takes you through skills step-by-step.

Use your personalised treatment dashboard to track your symptoms and your progress through the course. Your dashboard lets you see how your symptoms are changing in real-time, based on psychometric questionnaires.

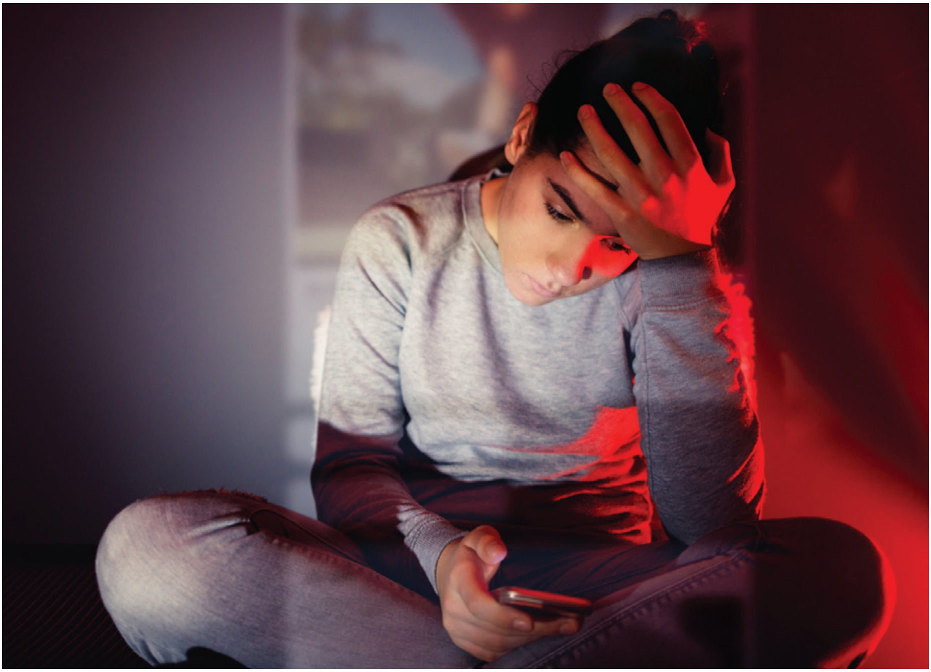

# Smiling Mind

https://www.smilingmind.com.au/

Smiling Mind is a unique tool developed by psychologists and educators to help bring balance to your life. They do this through an evidence-based **FREE** app where you can practice your daily meditation and mindfulness exercises from any device.

They explain mindfulness as paying attention to the present moment with openness, curiosity and without judgement. The team at Smiling Mind suggest you can do this by using the tool for around 10 minutes per day. This app was voted the #1 Apple Health & Wellbeing App 2018 in Australia and has been used by over 4.7 million people.

Smiling Mind has also posted a page on 'Supporting yourself and each other through Coronavirus' to help everyone navigate their way, as best they can, through a rapidly evolving global health crisis.

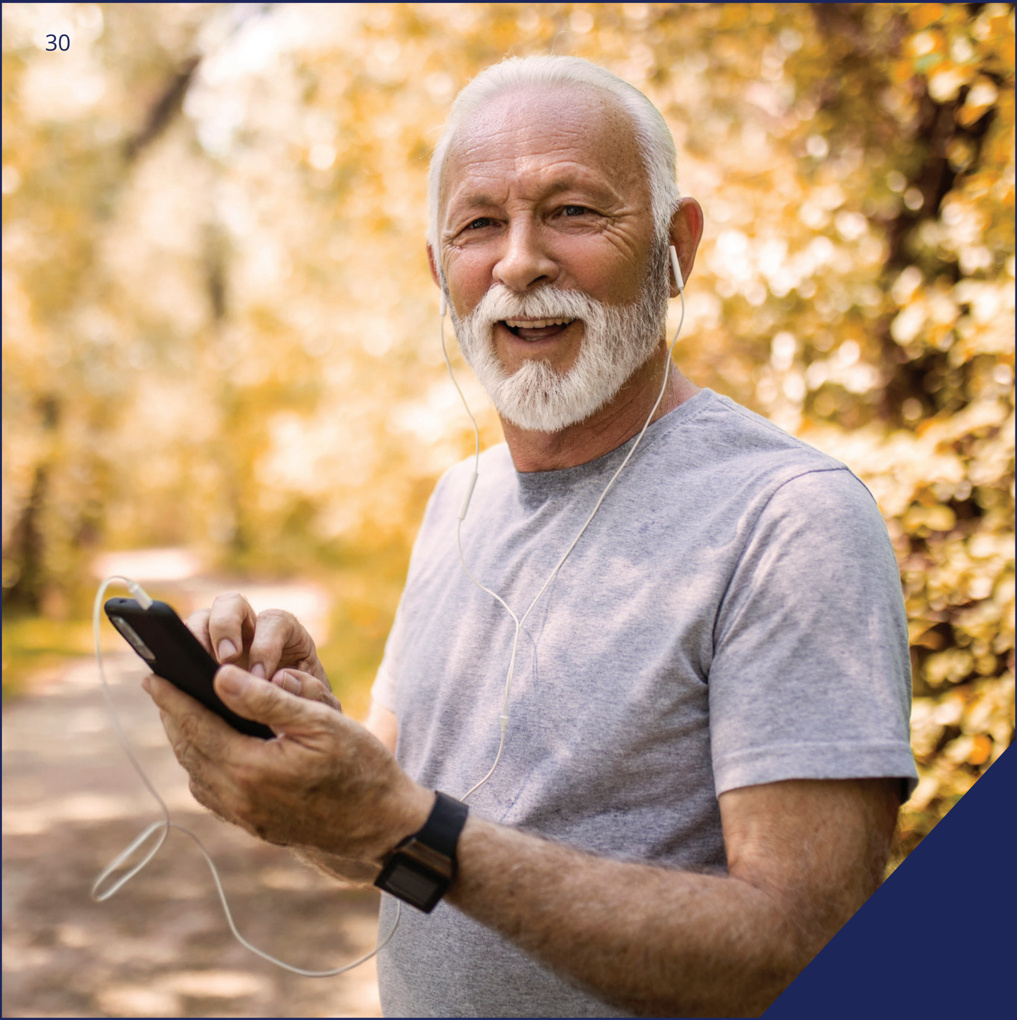

# 4 TELEPHONE COACHING PROGRAMS

# Get Healthy

The Get Healthy Information and Coaching Service is a **FREE**, confidential telephone-based service which helps people make lifestyle changes regarding: healthy eating, being physically active, and achieving and maintaining a healthy weight.

Tel: **1300 806 258**. Open: Monday – Saturday, 8.00am-8.00pm. Send an email with your details to **contact@gethealthynsw.com.au** and your health coach will call you back

The service runs for 6 months and delivers the coaching support and information you need to help you reach your health goals.

- When you enrol you will receive **FREE** of charge:
- Your own personal university-qualified health coach
- Up to **6 FREE** coaching calls
- Support to make changes over 6 months
- An information booklet and a coaching journal
- Access to a website where you can download tools and track your progress.

Visit the website: **www.gethealthynsw.com.au/**

---

# The COACH Program

## Individual Telephonic Health Coaching

Integrated and Community Health Directorate. Integrated Care Program.
Tel: **1800 113 644**.
Email: **WSLHD-IntegratedChronicCareProgram@health.nsw.gov.au**

### About the Program

The COACH Program, run by Western Sydney Local Health District, helps people with a chronic disease, such as diabetes, heart and lung disease, to manage their risk factors while working in partnership with their usual doctor/s.

Each patient with a chronic disease is allocated their personal coach. The coach will work with the patient, either on the telephone or by mailing out information in a series of regular coaching sessions over 6 months. The coach helps the patient to maintain their health by setting and achieving health goals for themselves.

Visit the website: **www.thecoachprogram.com/**

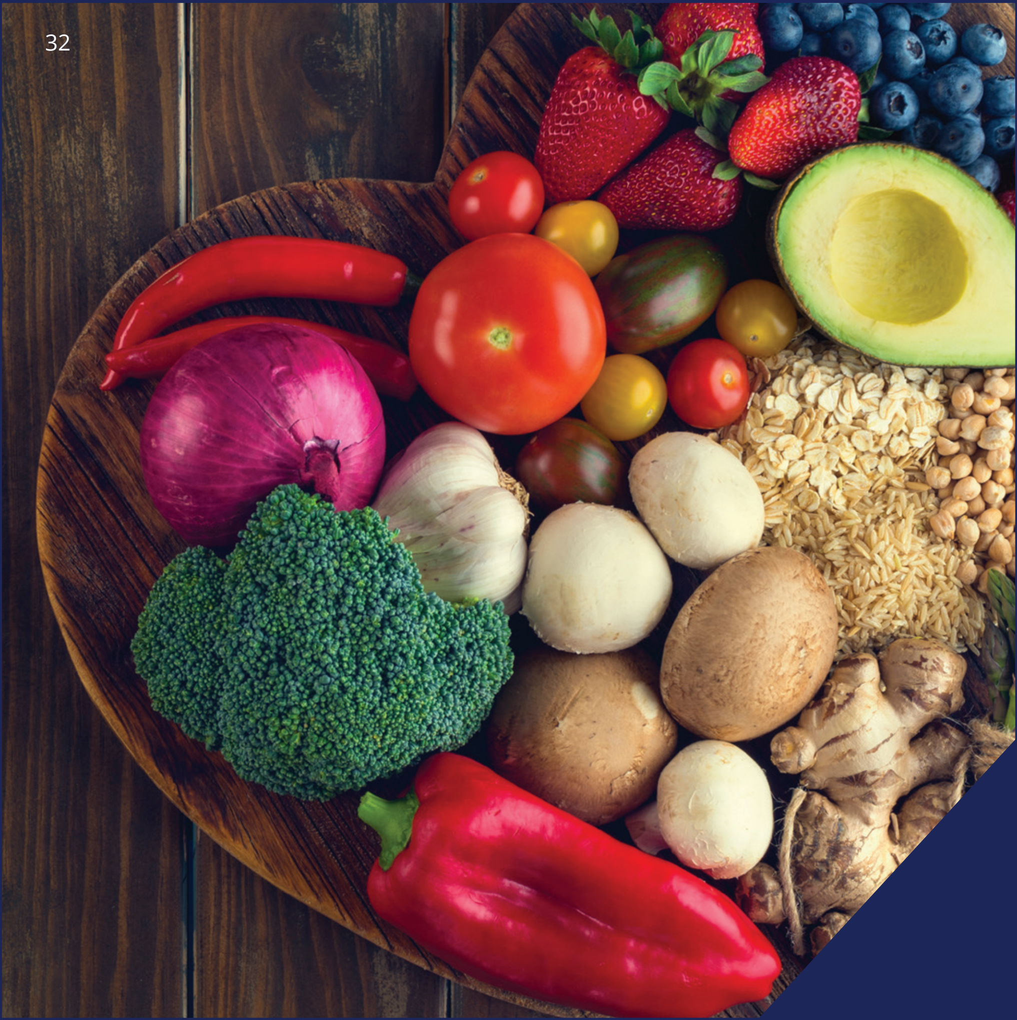

# 5 DIETARY GUIDANCE

# FoodSwitch App

### Health app | By: The George Institute for Global Health.

This **FREE** app allows you to make better food choices by providing simple health and nutrition information on a scanned product and suggesting healthier alternatives to 'switch' to. It is suitable for food shoppers and grocery buyers.

The FoodSwitch app works by using your mobile phone camera to scan the barcode of a packaged food. It then uses science-based algorithms to calculate and then display simple nutrition profiles of the food. This is presented as easy-to-understand nutrition information that can be viewed as either a Health Star Rating (HSR) or traffic-light coloured icons for key nutrients and energy.

The 'Health Star Rating' scores a food between 0.5 stars (least healthy) to 5 stars (healthiest). In the Traffic Light viewing mode, colour-coded icons show the amount of adverse nutrients such as fat, salt and sugar.

The FoodSwitch app can be downloaded from Apple App Store and Google Play Store.

Visit the website: **https://www.healthdirect.gov.au/foodswitch-app**

---

# No Money No Time

Looking to improve your diet? Have no money for fancy ingredients and no time to cook? This website will have you covered. Take the Healthy Eating Quiz to see where you can improve your diet and then find personalised **FREE** recipes and diet facts from leading experts in nutrition and dietetics from the University of Newcastle, Australia.

This website will help you cook healthy, easy recipes. If it's not tasty, fast, cheap and packed with good stuff, you won't find it here. The recipes are constantly updated, so you can keep coming back for more inspiration. They include useful information for each recipe including the time needed to make it, the number of ingredients and the cost per person. They even outline what equipment you will need and give you tweaks that you may like to try to cater for your own taste. Along with all this, they even provide some information on why this recipe is good for you. A great resource for everyone who is busy and careful with their budget.

Visit the website: **https://nomoneynotime.com.au**

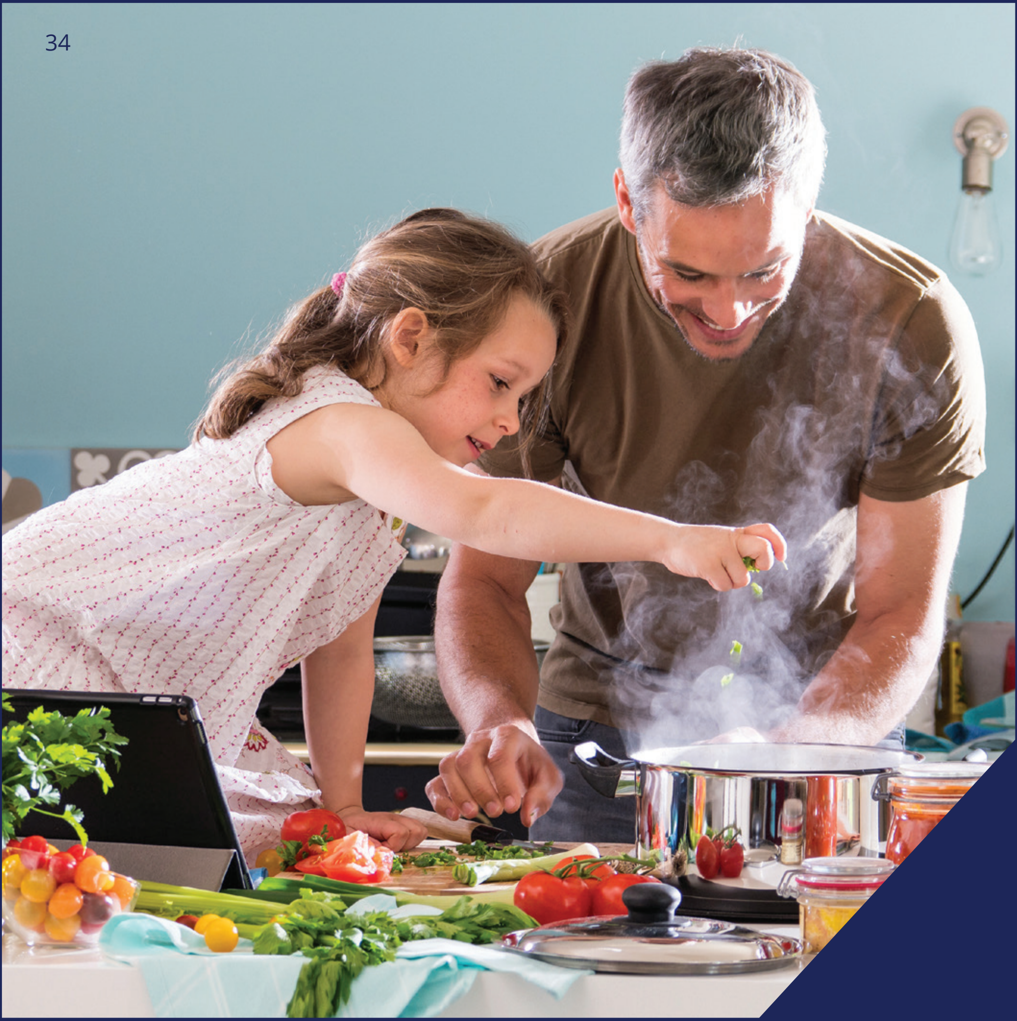

# 6 FREE ONLINE COOKING CLASSES

# The Kitchn's Cooking School

https://www.thekitchn.com/how-to-join-kitchn-cooking-school-22952427

This is a 20 lesson program where you can tackle an essential cooking topic each day. It provides videos, photos and detailed instructions.

You will learn a number of techniques starting with knife skills, moving on to cooking with grains, poultry, making salads and sauces and even perfecting the presentation of your dishes. With each lesson, you'll be given a choice of assignments to try out your new skills. (**FREE** to join at the Kitchn).

# Food Techniques

https://www.bbc.co.uk/food/techniques

BBC has information on a vast selection of cooking and food preparation techniques that provide cooks of all levels with excellent information, descriptions and demonstrations. Subjects are broken up into sections including how to prepare and cook fruit and veg, meat, poultry, eggs, fish, pasta, baking and sauces. Each technique is rated with its difficulty and provides a video, written description and a number of recipes you can try which use the technique.

This is a really wonderful resource for learning the very basic to advanced techniques. This website is **FREE** and there is also a selection of over 10,000 recipes on which to try out your new skills.

# Dinner Tonight Texas A&M

https://dinnertonight.tamu.edu/

This website was set up in order to teach people how to make quick, inexpensive and healthy dinners for themselves and their families. It provides great information for those who haven't had much experience in cooking, so is an excellent resource for those just starting out in the kitchen.

This website provides recipes and also gives weekly video demonstrations of various recipes. It also provides cooking tips and techniques, nutrition topics, menu planning basics and information on healthy living.

# Better Homes And Gardens

**https://bhg.com/recipes/how-to**

If you're looking to become a better cook, this website will help with its extensive cooking guide. There are cooking charts outlining the times and temperatures you need to cook meat, poultry, and vegetables to perfection. There are also fantastic how-to videos that allow you to see skills in action.

From basics tips like how to boil potatoes, to advanced skills such as how to butterfly a lobster tail – all are outlined on this website. (**FREE** at Better Homes and Gardens).

---

# Delia Online Cookery School

**https://www.deliaonline.com/cookery-school**

Delia Online features a host of cooking technique articles of varying levels of difficulty. Each lesson has step-by-step instructions with photos. Anyone who wants to learn to cook, can learn the skills.

Delia leads you by the hand step by step. This website allows you to learn to cook in your own time at your own pace.

---

# Instructables

**https://www.instructables.com/class/Cooking-Class/**

If you've ever wanted to learn how to cook, this is the class for you! By following along, you'll learn all the fundamentals of cooking that you need to create delicious meals.

You will be shown which pots, pans and tools are essential in your kitchen, and how to stock your kitchen pantry so you always have the ingredients on-hand to make a tasty homemade meal.

Along with properly outfitting your kitchen, you'll learn all about how to use a chef's knife safely and other simple ways to become a competent cook.

Once you've covered the basics, you'll be guided through eight different cooking techniques that will improve every meal you make. Each cooking technique is also paired with a recipe collection so you can practice these techniques while making a delicious meal. Take this class and you'll be feeling like a great cook in no time!

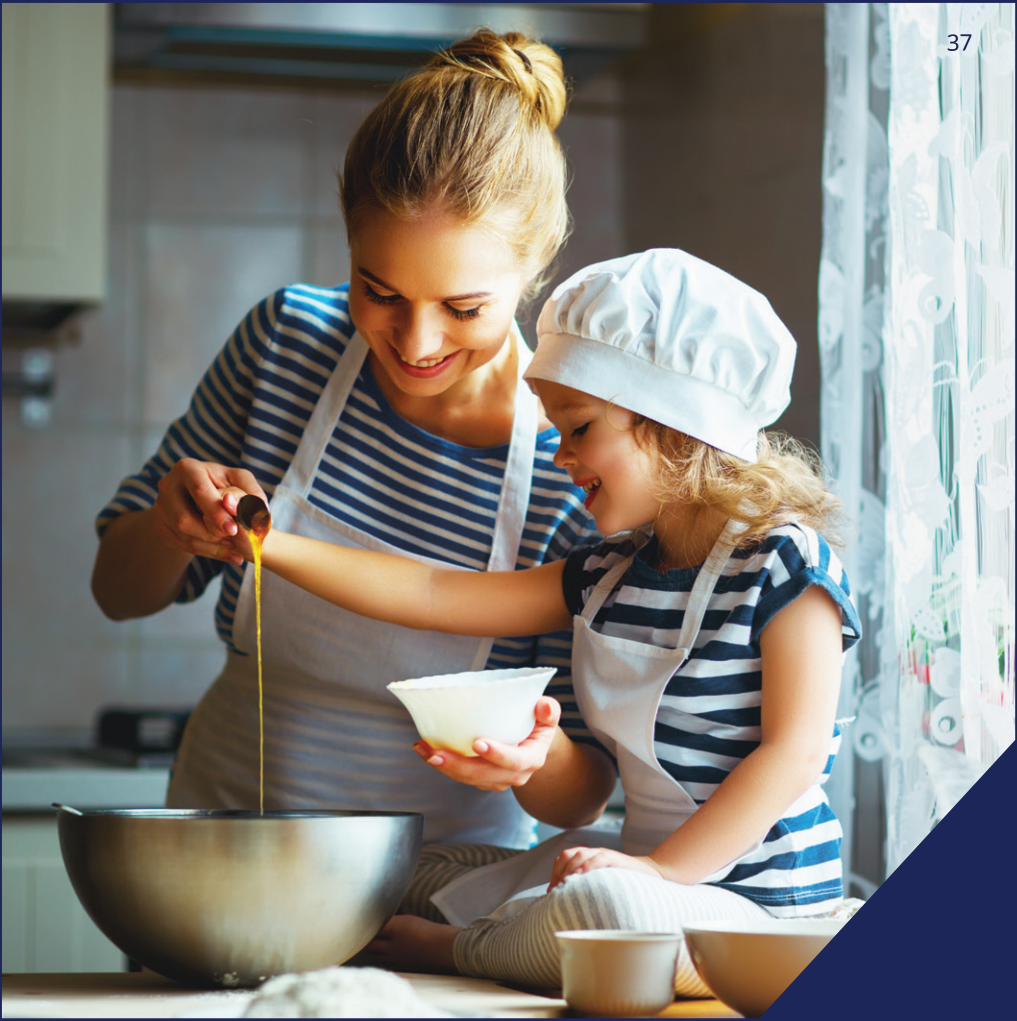

# 7 EASY DINNERS

# Web Help For Non-cook Dinners

The following websites give an outstanding range of non-cook dinners. Most are healthy, very easy to prepare and require basically no cooking skills.

Instead of cooking, you will chop, squeeze, slice, arrange, dollop and mix to make beautiful meals for yourself and your family. You will be inspired to prepare tasty salads, wraps, poke bowls, snack boards, grain bowls, pitas, soups and tartines.

These recipes will allow you to assemble food that may have already been cooked (such as ham), use leftovers, or simply raid the pantry or fridge.

https://www.bbcgoodfood.com/howto/guide/healthy-no-cook-dinners

https://www.taste.com.au/quick-easy/galleries/our-best-ever-no-cook-dinners/l674mDpo?page=6

https://www.thekitchn.com/13-no-heat-no-cook-meals-that-still-fill-you-up-240527

https://www.delicious.com.au/recipes/collections/no-cook-dinner-recipes/aa907e5e-65bf-4113-b2f9-ac66525dc30e?page=4

# Basic Healthy Dinners

These websites are perfect for the not-too-confident cook. They provide a wide variety of very simple recipes that are quick, nourishing and easy to prepare.

You can choose from soups, salads, burgers, stir fries and pasta dishes (just to name a few). You will be surprised how your skills and confidence will develop after cooking these delicious recipes.

https://www.bbcgoodfood.com/recipes/collection/quick-and-healthy

https://www.taste.com.au/recipes/collections/healthy-quick-dinner-recipes

# Australian Healthy Food Guide Magazine

https://www.healthyfoodguide.com.au/

This magazine has tips and information on all kinds of healthy foods, diets, cooking skills – and even foods that you can cook when you arrive home at 5pm and want to get a nutritious meal on the table.

It was voted the best food magazine in Australia in 2019 and is available at newsagents, or you can subscribe. On their website, there are back issues with a library of their previous recipes, expert advice and health articles that you may like to look at – **FREE**!

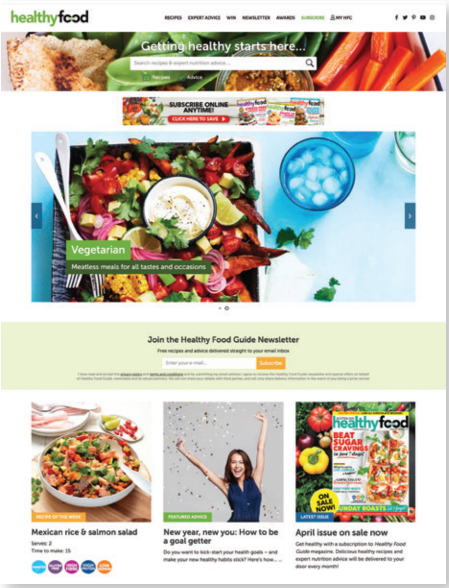


There is a massive range of healthy recipes that contain both instructions and extensive nutritional information. You will even be given the approximate time it takes to make each recipe and the cost per serve.

For anyone trying to have a healthy diet, lose some weight or even give up takeaway food, this magazine will give you an endless supply of delicious recipes and healthy alternatives.

**Aruni Ratnayake**
**Diabetes Prevention Officer**

Telephone: (02) 8670 0079
Mobile: 0428 681 983
Fax: (02) 9851 6146
Email: Aruni.Ratnayake@health.nsw.gov.au
Web: www.westernsydneydiabetes.com.au
Follow us on LinkedIn

© February 2024
