# Supplementary material for: Integrating point-of-care diabetes detection with lifestyle counselling in community settings: outcomes from Western Sydney, Australia
Source: BMC Health Serv Res. 2024 Aug 13;24:926. doi: 10.1186/s12913-024-11335-y (PMC11323375; doi:10.1186/s12913-024-11335-y)
Supplement: Supplementary file 7 — Supplementary Material 7 [file 12913_2024_11335_MOESM7_ESM.pdf]

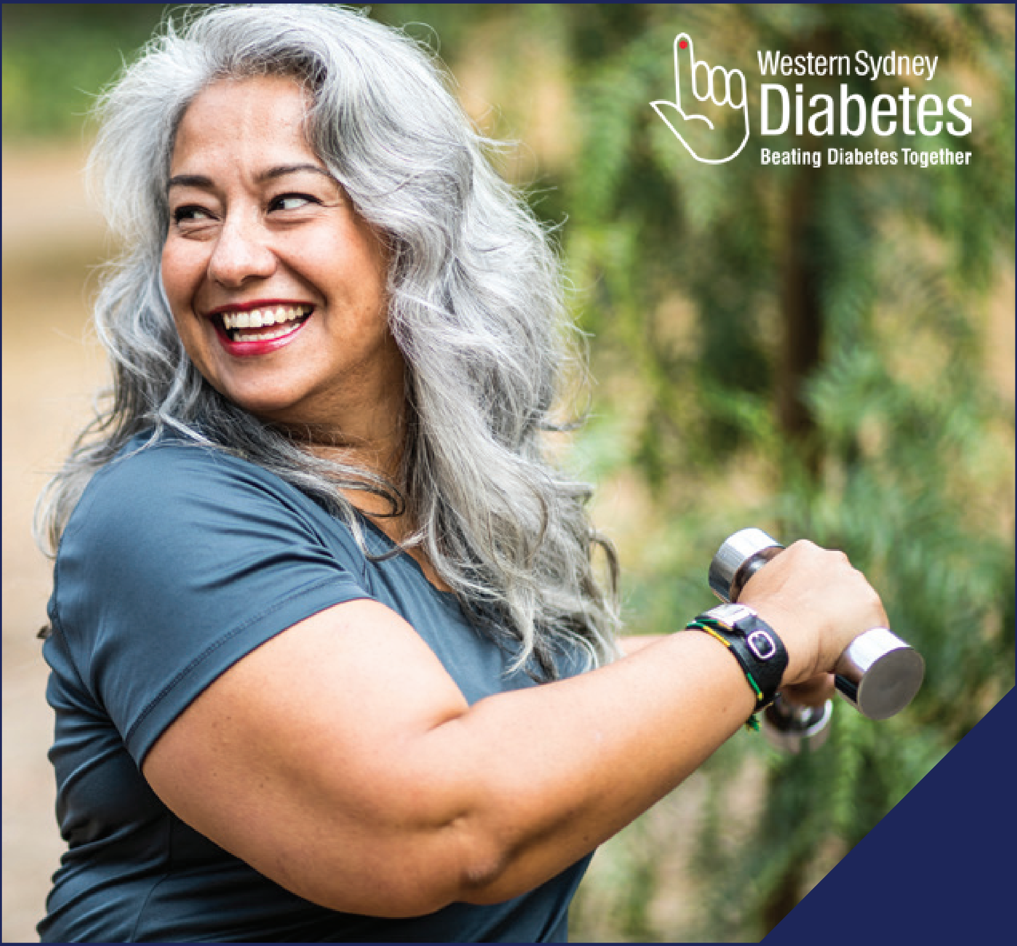

HEALTHY LIVING OPTIONS

FOR WESTERN SYDNEY

phn WESTERN SYDNEY
An Australian Government Initiative

diabetes australia

NSW GOVERNMENT

About this booklet

Improving the overall health and wellbeing of people is the way to stop the slide towards diabetes in western Sydney.

Western Sydney Diabetes has worked with its Alliance partners and others in the western Sydney area to identify programs that can help the whole community embrace healthy eating and exercise options – right on their doorstep.

Too often, people feel they do not know where to turn to for advice on managing their health. The programs listed here, and on the Western Sydney Diabetes website, are not exhaustive but are a perfect starting point.

Our main focus has been on programs that are either free or inexpensive, and many offer a range of programs to provide options for everyone.

Please visit our website for more information on healthy living options: www.westernsydneydiabetes.com.au

Contents

1 Local Exercise Programs 4
- Blacktown Aquatic Centre 5
- Blacktown Leisure Centre Stanhope 6
- Charlie Lowles Leisure Centre 7
- City of Parramatta Rec & Wellbeing 9
- Cumberland Council 10
- Cumberland Council Lifelong Learning 11
- Football NSW Walking Football 12
- Guildford Swim Centre 13
- Granville Swimming Centre 14
- Heart Foundation Walking Groups 15
- Kids On Bikes 17
- Live Life Get Active 18
- Merrylands Pool 19
- Mount Druitt Swimming Centre 20
- North West Community Services 21
- NSW Active and Healthy 22
- OLOC Get yourself Moving 23
- parkrun 24
- Reclink 25
- Riverstone Swimming Centre 26
- Robinhood Free Outdoor Fitness 26
- Ruth Everuss Swimming Centre 27
- Waves Fitness & Aquatic Centre 28
- Wentworthville Swimming Centre 29

2 Online Healthy Lifestyle Classes 30
- Fitness Blender 31
- Healthy and Active for Life Online 32
- NHS Fitness Studio 33
- NIKE Training Club 33

3 Dietary Assistance 34
- Box Divvy 35
- Cancer Council Free Resources 36
- CSIRO Low Carb Diet 36
- No Money No Time 37

4 Phone Based Programs 38
- Get Healthy Information and Coaching Service 39
- The Coach Program 39

5 Face to Face Programs 40
- Bilingual Community Education 41
- Blacktown Older Women's Network 42
- Blacktown Women's and Girl's Health Centre 43
- Chronic Disease Self Management Program 44
- Collective Leisure 45
- Diabetes Australia Education Programs 46
- Go4Fun 48
- New Edge Performance 49
- Optimum Health Solutions 51
- Street Soccer 53
- Sydney West Sports Medicine 54
- Workers Blacktown Getting Healthy Programs 55

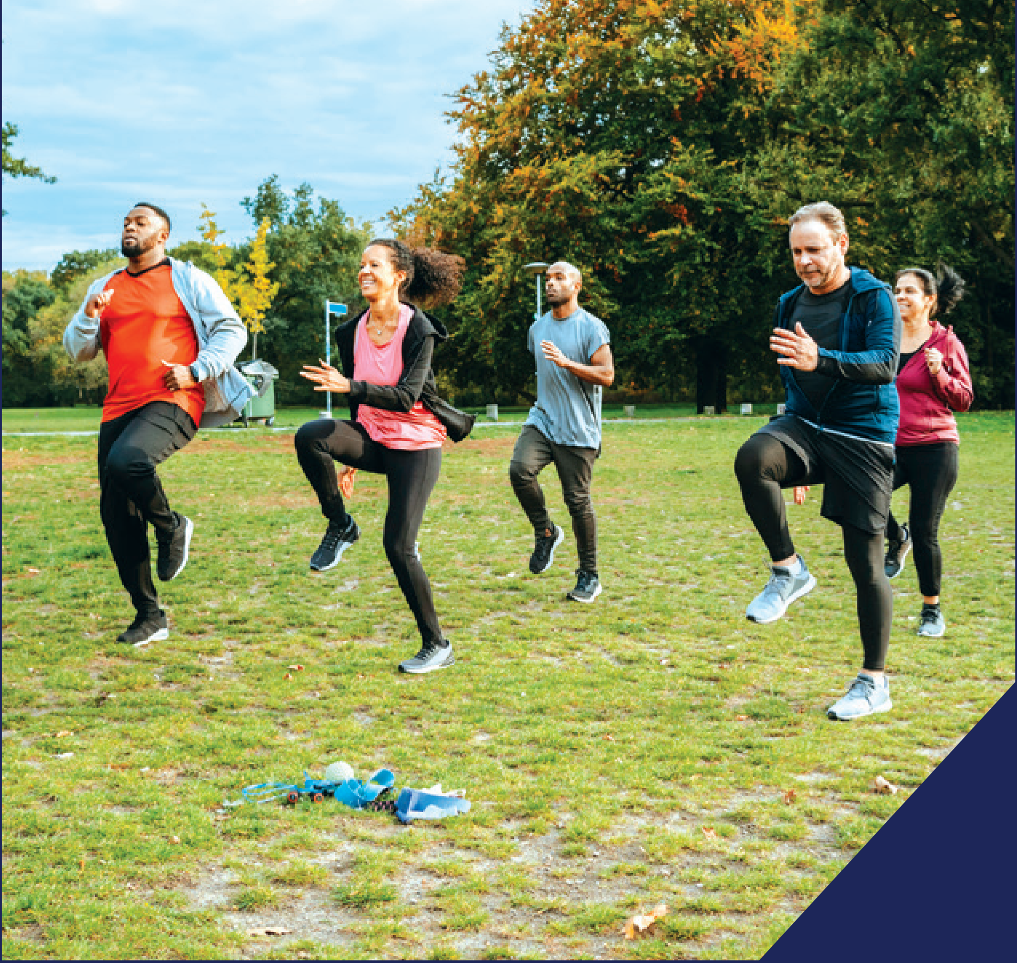

1 LOCAL EXERCISE PROGRAMS

Blacktown Aquatic Centre

Address: 4 Boyd Street, Blacktown
Tel: (02) 8869 9000

Operating Hours				
	Pool Sep-Mar	Pool Apr-Sep	Gym Sep-Mar	Gym Apr-Sep
Monday-Friday	5.30am-7.30pm	5.30am-7.30pm	6.00am-9.00pm	5.30am-7.30pm
Saturday	6.00am-5.00pm	6.00am-2.30pm	6.00am-5.00pm	6.00am-2.30pm
Sunday	8.00am-5.00pm	6.00am-2.30pm	8.00am-5.00pm	6.00am-2.30pm
Public Holidays	8.00am-5.00pm	8.00am-2.30pm	8.00am-6.00pm	8.00am-2.30pm

The Centre prides itself on being more than just a competition pool, offering health, fitness, fun and leisure opportunities for everyone. This includes 3 pools, splash pad, gym, group fitness studio, creche, Aqua Learn to Swim, Kidz Blitz school holiday program and even a skate ramp!

Group fitness classes – HIIT, Pilates, Yoga, Staying Active, Gym circuit, Mind Body, Young at Heart

Aqua Group Fitness – Aqua Combo, Aqua Shallow, Aqua Zumba, Aqua Blast, Aqua Deep.

Yoga Natal – is designed for expectant mums from 16 weeks to birth. The Yoga Natal program consists of gentle stretching and breathing techniques designed to increase the strength and flexibility of muscles needed for childbirth.

There are charges for these classes, however they are quite reasonable and appear on the website.

For learn to swim classes contact Blacktown Aquatic Centre:
aqua.blacktown@blacktown.nsw.gov.au or **02 8869 9000**

Our brand new splash pad has various water features and play zones. The splash pad is designed for children of all ages and abilities to cool off and have fun.

The splash pad is included in aquatic entry prices.

Visit the website: **https://www.blacktown.nsw.gov.au/Services/Aquatic-and-leisure-centres/Our-pools-and-leisure-centres/Blacktown-Aquatic-Centre**

Blacktown Leisure Centre Stanhope

Address: Cnr Stanhope Parkway and Sentry Drive, Stanhope Gardens
Tel: (02) 9421 2600

Blacktown Leisure Centre, Stanhope offers the community the latest in an exciting range of health, aquatic, leisure and recreation programs and services. This includes pools, gym, group fitness, creche, 4 court multi- sport stadium, Aqua Learn to Swim, Kidz Blitz school holiday program, tennis courts, squash courts, spa and sauna, venue hire.

Our Group Fitness classes suit varied abilities from beginner through to experienced, with low-impact and high-impact class options. With over 50 group fitness classes to choose from weekly, there's something for everyone. There are charges for these classes however they are quite reasonable. A full description of the classes, enrolment and timetable for each term is outlined on the website.

Operating Hours			
	Pool	Gym/Stadium	Tennis/Wave Pool
Monday-Friday	5.30am-9pm	5.30am-9pm	9am-9pm
Saturday & Sunday	8am-5pm	8am-5pm	8am-5pm
Easter Monday, Boxing Day & New Years Day	Noon-5pm	Noon-5pm	Noon-5pm
ANZAC Day	1pm-5pm	1pm-5pm	1pm-5pm
All other public holidays	8am-5pm	8am-5pm	8am-5pm

Closed Good Friday and Christmas Day

H2O No Limits This fabulous, simple to follow aqua program is a great way to get started in our Lifestyle Zone. Includes toning with the aqua dumbells and woggles. H2O is suitable for everyone!

Young at Heart Aqua 45 minute low impact water class for people over 50 years.

Group exercise classes and personal training are also on offer. These include classes in Body Pump, Body Combat, Boxing, Pilates, Zumba, Young at Heart, Spin classes, HIIT, Yoga, Staying Active Yoga, Pilates, and Cycle Group Fitness Classes.

Aqua Natal and Yoga Natal is designed for expectant mums from 16 weeks to birth. The Yoga Natal program consists of gentle stretching and breathing techniques designed to increase the strength and flexibility of muscles needed for child birth.

Visit the website: **https://www.blacktown.nsw.gov.au/Services/Aquatic-and-leisure-centres/Our-pools-and-leisure-centres/Blacktown-Leisure-Centre-Stanhope**

Charlie Lowles Leisure Centre Emerton

Address: 35 Jersey Road, Emerton
Tel: (02) 8822 6000

More than just a pool, Charlie Lowles Leisure Centre Emerton offers the community the latest in an exciting range of health, aquatic, leisure and recreation programs and services. This includes 6 pools, beach volley ball courts, 2 multi-purpose indoor sports courts, group fitness classes (HIIT, Mind Body, Boxfit and Aqua Aerobics), Aqua Learn to Swim (**aqua.emerton@blacktown.nsw.gov.au**) and our Kidz Blitz school holiday program.

Aqua Aerobics: Low impact total body workout includes toning with aqua dumbbells and noodles. Suitable for all fitness levels.

Aqua Boxfit: A 45-minute class combining boxing combinations, HIIT and resistance training in a water environment.

Aqua Tabata: A HIIT class designed for water using maximum effort for 20 seconds and recovery for 10 seconds.

Boxfit: Boxfit is a high intensity cardio class with gloves and pads. Learn combinations and correct technique while raising your heart rate and working up a sweat.

HIIT: High Intensity Interval Training combining strength and cardiovascular exercises for the ultimate workout.

Mind Body: This class consists of gentle stretching, correct breathing techniques, yoga postures and relaxation. It improves your body and mind awareness, increases your flexibility and assists with stress management. This class caters for everyone.

Operating hours: See next page:

Visit the website: **https://www.blacktown.nsw.gov.au/cllce**

Charlie Lowles Leisure Centre Emerton (continued)

Operating Hours			
	Pools	Stadium	Warm water therapy pool
Monday - Friday	Indoor Lap Pool – 7.30am-8pm<br>Indoor Program Pool – 3pm-8pm<br>Indoor Leisure Pool – 3pm-8pm<br>Outdoor Pools (Oct-Mar) – 3pm-6pm	7.30am-9pm	7.30am-Noon<br>3.00pm-6.00pm (Monday & Wednesday)
Saturday	Indoor Lap Pool – 8am-5pm<br>Indoor Program Pool – 9am-5pm<br>Indoor Leisure Pool – 9am-5pm<br>Outdoor Pools (Oct-Mar) – 11am-5pm	8am-5pm	8am-Noon
Sunday	Indoor Lap Pool – 8am-5pm<br>Indoor Program Pool – 9am-5pm<br>Indoor Leisure Pool – 9am-5pm<br>Outdoor Pools (Oct-Mar) – 11am-5pm	8am-5pm	Closed
Public holidays	Indoor Lap Pool – 8am-5pm<br>Indoor Program Pool – 9am-5pm<br>Indoor Leisure Pool – 9am-5pm<br>Outdoor Pools (Oct-Mar) – 11am-5pm	8am-5pm	Closed

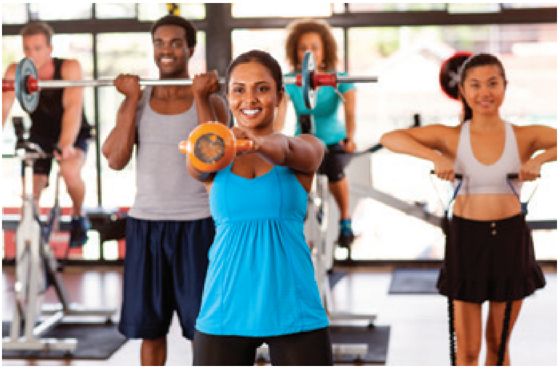

City of Parramatta Recreational & Wellbeing Programs

City of Parramatta Council offers an extensive range of physical and recreational activities through the Active Parramatta Fitness Program and the Over 55's Leisure and Learning Service. Classes are offered throughout the Parramatta Local Government Area and run on a term by term basis.

Active Parramatta Fitness Program

A number of **FREE** and low cost classes are offered throughout the year and cater to all fitness levels. Classes typically include, but are not limited to Zumba, Pilates, Yogalates, Circuit Training, Outdoor Fitness Training, Core and Strength, Aerobic Boxercise, Fitness Training and Bodyweight Training. There are also school holiday, multisport and dance programs for kids. More information is available on: **www.cityofparramatta.nsw.gov.au/recreation/active-parramatta/active-parramatta-programs**. Registrations are made through an online booking service: **parramatta-events.bookable.net.au**

Over 55's Leisure and Learning Service

A large range of educational, physical, recreational and social activities are offered throughout the year for people over 55 years of age through this service. Classes are offered at low cost or **FREE** for some activities and include, but are not limited to: Zumba Gold, Tai Chi, Yoga, Pilates, Gentle Exercise, Line Dancing, Walking Groups and Tennis. Online classes are also available for Strength & Balance and Chair Yoga.

More information is available on: **www.cityofparramatta.nsw.gov.au/community/over-55s-activities-ageing-wellness/over-55s-leisure-and-learning/term-based-activities**

For more information, call the team on **(02) 9806 5121** or email: **communitycareadmin@cityofparramatta.nsw.gov.au**. Registrations are made through an online booking service: **parramatta-events.bookable.net.au**

Active Parramatta Van

The Active Parramatta Van is a mobile service that directly delivers **FREE** health and fitness programs in the Parramatta LGA. The Van services the whole community across the lifespan but works more closely with youth, families, over 55's and Culturally and Linguistically Diverse (CALD) groups. Classes such as Mums N Bubs Fitness, Adult Fit and Better Ageing Strength/Cardio/Gentle Exercise are available.

For enquiries email: **apvan@cityofparramatta.nsw.gov.au**

Cumberland Council

ETS Gym

Located inside Eric Tweedale Stadium at 10 Montrose Avenue, Merrylands, the ETS Gym is open 7 days a week, from 6am to 12 midnight: https://www.cumberland.nsw.gov.au/etsgym

For more information contact: Dean Savetta (Manager, Events Management): dean.savetta@cumberland.nsw.gov.au

Get healthy and trim with:

- Memberships from $10 per week
- Brand new venue and equipment
- Fitness classes every week
- Regular Women's classes
- Membership packages with Swim Centre classes
- Fully airconditioned facility

Discount for Seniors

Seniors that provide their valid pensioner concession card when enrolling into any services will receive a 20% discount.

Wellness classes for Seniors

Our low impact classes, Pilates, Stretch and Mobility and the Zumba morning class are all suitable for active seniors over 55 years of age.

Classes

Body Conditioning – Women's only class
Bootcamp – High impact
Boxing – Medium to high impact
Cross Training – High impact
HIIT – High Intensity Interval Training – Medium to high impact
Pilates – Low impact
Stretch and Mobility – Low impact
Zumba – Low impact

Phone 8757 9311 or email: ETS.granville@cumberland.nsw.gov.au

Cumberland Council Lifelong Learning

Tel: (02) 8757 9000
Email: lifelonglearningofficer@cumberland.nsw.gov.au
For more information contact: Teneille Bush, Senior Coordinator, Seniors & Disability Services: teneille.bush@cumberland.nsw.gov.au

These classes are run by Cumberland Council on a term by term basis and are available to Seniors (over 55s). Both day and evening times are available. Classes are very reasonably priced and run at a variety of venues throughout the Cumberland Local Government Area.

Classes

Classes differ in each location but may include:

- Gentle Fitness for Seniors
- Table Tennis
- Yoga and Relaxation
- Zumba Gold
- Line Dancing
- Dancefit

Visit the website: **www.cumberland.nsw.gov.au/lifelonglearning**

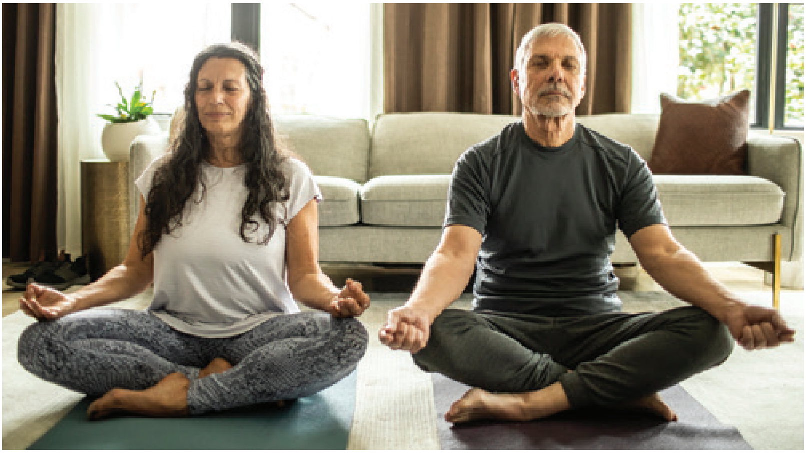

Football NSW Walking Football

- The Football NSW Walking Football programs are a slow-paced version of the beautiful game aimed at over 50s (male and female)
- Matches are played on weeknights with modified formats (5v5/6v6) on smaller pitches with shortened game times
- Walking Football is non-contact with the Walking Rule defined as ‘always having one foot on the ground’
- Football NSW Walking Football programs are designed to help people maintain an active lifestyle and promote participation of the game in a fun, inclusive and social environment
- New players are always welcome and helped to connect with the teams.

Email: nikki.riddle@hillsfootball.com.au

Visit the website: https://hillsfootball.com.au/walking-football-2/

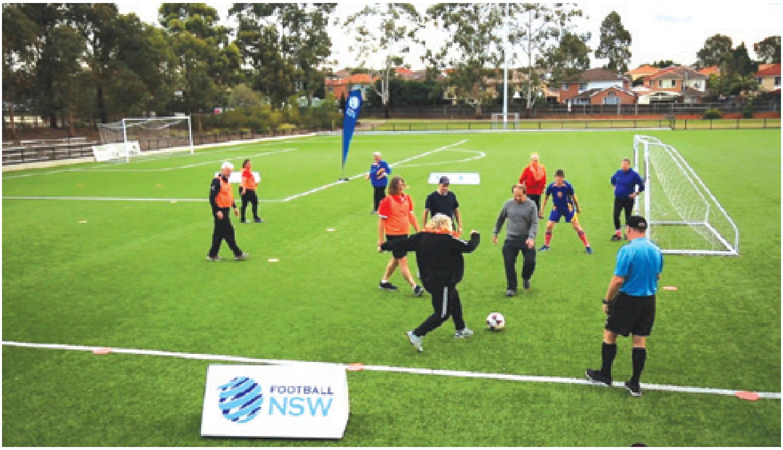

Guildford Swim Centre

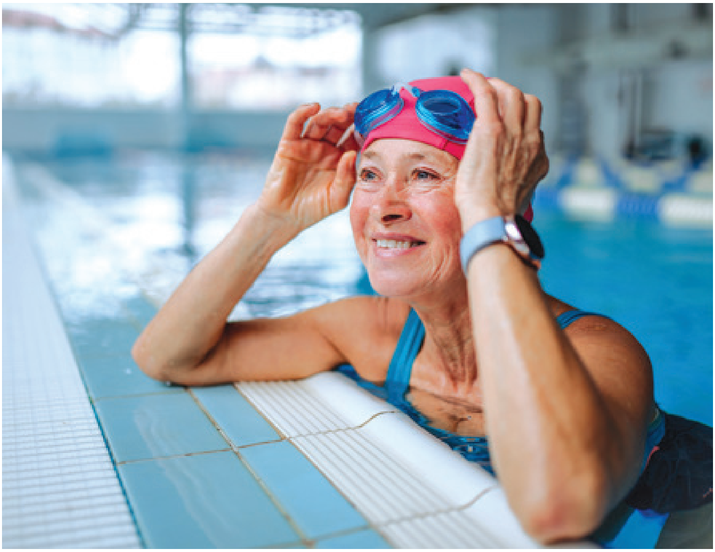

Address: 1 Tamplin Road, Guildford
Tel: (02) 8757 9050

Regular opening hours (year round)

- Monday to Friday: 6am to 8pm
- Saturday: 6am to 5.30pm
- Sunday and public holidays: 7.30am to 5.30pm
- Closed Good Friday, Christmas Day and Boxing Day
- 25m indoor pool
- 25m outdoor pool
- Infants pool
- Lap lanes
- Swimming lessons for all ages. Group and private lessons are available.

Visit the website: **https://www.cumberland.nsw.gov.au/guildford-swim-centre**

Granville Swimming Centre

Address: Enid Avenue Granville
Tel: (02) 8757 9050 (Granville, Guildford and Merrylands)

Located in the heart of Granville, there are 3 pools including an Olympic pool, 25 metre program pool and leisure pool.

Entry is very inexpensive at approximately $7.60 for an adult and $5.60 per child. For a full list of fees visit Swim Centre Fees and Charges.

Opening hours

October to March

- Monday to Friday: 5am to 8pm
- Saturday, Sunday and Public Holidays: 7am to 7pm
- Closed Christmas Day and Boxing Day

April to September

- Monday to Friday: 5am to 7pm
- Saturday, Sunday and Public Holidays: 7am to 4pm

Classes

- Swimming lessons from preschool to adults. Private lessons are also available.

Visit the website: **https://www.cumberland.nsw.gov.au/granville-swim-centre**

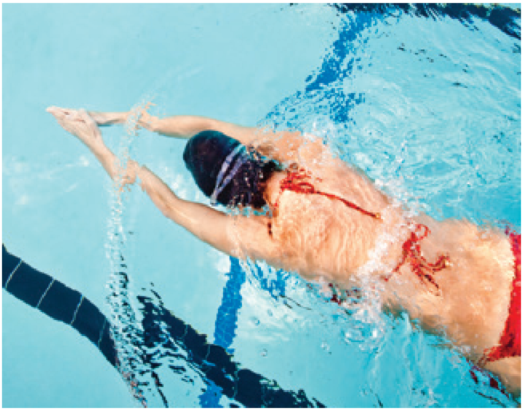

Heart Foundation Walking Groups

Heart Foundation Walking is Australia's largest **FREE** walking network and there are a few ways you can join!

Join a Walking Group

Heart Foundation Walking have established a network of walking groups all over Australia, led by community volunteer Walk Organisers. The Walking Groups are a social, fun and easy way to get more physically active. Walking groups come in different sizes and walk at various times of the day and days of the week, for varying distances and differing levels of difficulty.

You can search and join a group in your area here: **walking.heartfoundation.org.au/walking**

Lead a Walking Group

If there are no walking groups that suit, you can volunteer to start your own group as a Walk Organiser here: **https://walking.heartfoundation.org.au/lead-a-walk**. You can create a group for your local community members to join or set up a private group for your workplace or community group.

Personal Walking Plans

Discover the Heart Foundation's Personal Walking Plan program designed for anyone wanting to start or improve their weekly exercise habits. Tailored to meet your current fitness levels, this program spans six weeks and sets out your plan for walking, strength exercises, and stretching routines. Created by experts in the field, it provides more than just technical instructions – it aims to make your fitness journey enjoyable while helping you cultivate a lasting habit.

Throughout the program, you'll experience a gradual increase in confidence, strength, and flexibility, leaving you with a profound sense of accomplishment!

To support you every step of the way, you'll receive a personalised weekly plan delivered straight to your email inbox. In addition, we'll send you valuable tips via SMS, and you can reach out with any questions you may have. Plus, gain exclusive access to our vibrant online community, where participants and like-minded individuals gather to share their walking experiences.

Heart Foundation Walking Groups (continued)

In a recent survey, an impressive 99 percent of participants expressed their commitment to sustaining regular walking or engaging in physical activity even after completing the program. Don't miss out on this incredible opportunity to improve your walking habits and consistency. Take the first step today and sign up now!

1. **Complete the 2-minute form**
   We'll work out the right plan for you based on your current level of activity.
2. **Get your plan**
   We'll send your plan directly to your email.
3. **Get moving and feel the benefits**
   Over six weeks, we'll send information and advice to your email and mobile to help you stick with it.

For more information visit **http://walking.heartfoundation.org.au**, call the Heart Foundation on **13 11 12**, or email **walking@heartfoundation.org.au**

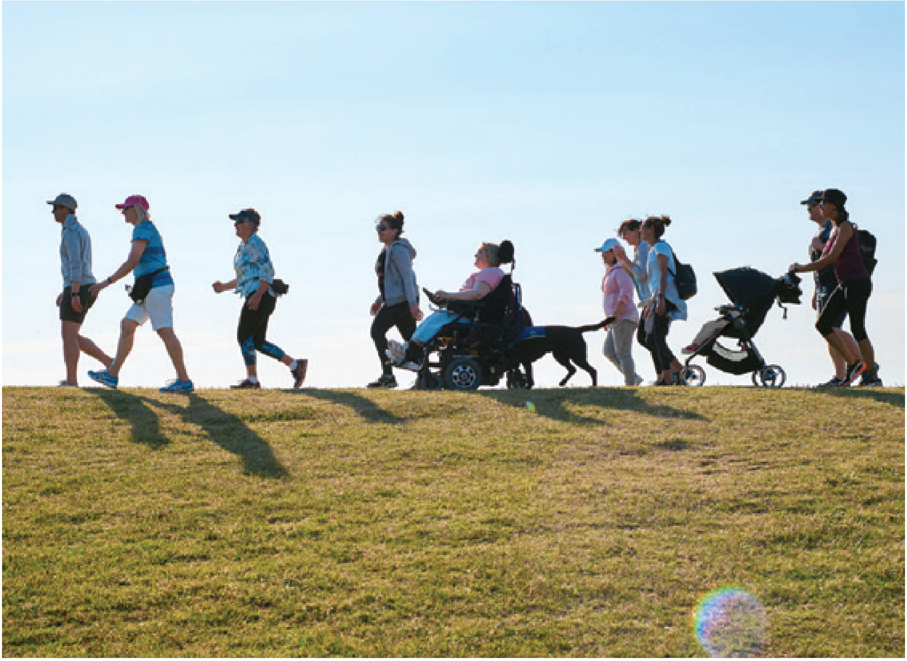

Heart Foundation Walking Groups (continued)

New 'Culture-based' walking group in western Sydney

Meets every Saturday morning at 7am, Jannawi Gardens, Nurragingy Reserve, Knox Road, Doonside.
Email: Filipinowalkers@gmail.com

Use QR code to join the group.

Kids on Bikes

Kids on Bikes is a ride school for all ages and skill levels. The trained coaches can make riding fun. Private or Group coaching classes are available to meet your needs. You can go from 'zero to hero' in no time at all. Whether you are looking to ride for pleasure, develop ride competency, ride to school or race competitively, they can help. No one is ever too young or too old to enjoy the simple pleasure of riding a bike. Cycling is great for fitness, health, transport, recreation and sport. Kids on Bikes can get you riding and having fun on your bike.

The coaching classes are available seven days a week. All learn to ride lessons are 1 hour in length and they can supply the bikes. You just need to bring your helmet if you own one. Although they are based near Sydney Olympic Park the instructors are happy to deliver classes within the wider Sydney region. They also cater for 'big kids' who'd like to learn to ride or just improve their cycling skills.

Classes are run one on one, at your own pace in a safe open environment that is both informative and enjoyable. The trained professional coaches have years of experience to support you from learning to ride, through to achieving your cycling related goals.

Call for more information on the classes:

- Learn to Ride
- Cycle Proficiency
- Women's Specific Classes
- Shared Pathways
- Group Rides
- Commuting & Traffic Skills
- Fitness & Performance Coaching

Details: **Gay Watson: 0403 147 166**; **Ian Watson: 0403 203 253;**
email: **2learn2ride@gmail.com** or website: **https://kidsonbikes.com.au**

Live Life Get Active

A Fitter, Healthier and Happier Australia

WOULD YOU LIKE TO: Have more energy, feel stronger, improve your balance, reduce your anxiety and have fun in the fresh air whilst making new friends all for **FREE**?

All you have to do is register to become a member of Live Life Get Active and all of this is for **FREE.**

Live Life Get Active is a registered health promotion charity that specifically looks at addressing some of the key chronic health issues facing Australians today. Issues such as diabetes, obesity, poor mental health and social isolation. Their proposition is to get people up moving and having fun either in a local park or online. They help you understand your health, lifestyle behaviours and review how you eat.

They have 14 park locations across Western Sydney, where you can attend **FREE** exercise sessions.

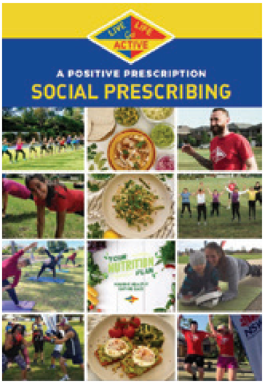

Why not try Yoga where you can relax, stretch and take your mind off your day to day activities or Cross training to build your strength and improve your fitness, heart health and can also help with weight loss. And they have Boxing to build your fitness whilst having fun!

These sessions run Monday to Friday during school terms, each class runs for 45 minutes with all equipment provided and all you need to bring is your water bottle. Our trainers are all qualified and are there to help you improve your fitness in a fun and non-judgemental environment. All sessions are adjusted to cater to all abilities and members are encouraged to go at their own pace. All this is for **FREE.**

Registered members also have access to their online fitness. So if you cannot make it to an In Park session, why not try their online fitness Live Streams or On-Demand videos. These sessions include meditation, yoga, pilates, cross-training, Tai Chi and even dance.

Join Live Life Get Active today and start your new journey to health, fitness and happiness for **FREE**.

Register to become a member at **www.livelifegetactive.com**

Merrylands Pool

Address: 100 Burnett Street, Merrylands
Tel: (02) 8757 9050
For more information contact: Tony Micallef (Swim Centre Operations Manager): tony.micallef@cumberland.nsw.gov.au

This venue has:

- 8 lane, 50m outdoor, heated competition pool
- Outdoor, heated training pool
- Outdoor, heated baby pool.

Check the website for entry fees.

Opening hours

November and December

- Monday to Saturday: 6am to 7pm
- Sunday and Public Holidays: 7am to 7pm
- Closed Christmas Day and Boxing Day

January and February

- Monday to Saturday: 6am to 8pm
- Sunday and Public Holidays: 7am to 8pm

March

- Monday to Saturday: 6am to 7pm
- Sunday and Public Holidays: 7am to 7pm

April to September

Merrylands Swim Centre closes during the winter season. Granville and Guildford Swim Centres remain open over winter.

October

- Monday to Saturday: 6am to 7pm
- Sunday and Public Holidays: 7am to 6pm

Visit the website: **https://www.cumberland.nsw.gov.au/merrylands-swim-centre**

Mount Druitt Swimming Centre

Address: Ayres Grove, Mount Druitt
Tel: (02) 9625 6360

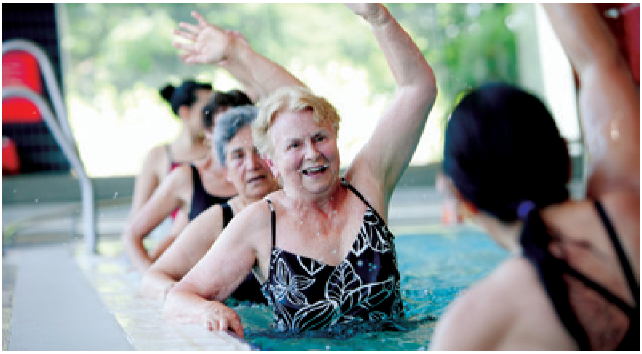

This seasonal pool is open from 9.00am to 6.00pm daily from September to March each year. Cost: Adult $7.20, children 4 years and under FREE, concession $5.70 and children 5 years and over $5.70 and eligible pensioner (Blacktown LGA residents) FREE.

Take advantage of the fantastic fitness program at Mount Druitt Swimming Centre.

Aqua Zumba

You can dance up a storm with the latest fitness trend, Aqua Zumba. Ring the Centre for times and details as they will vary during the year.

If you're not convinced about Aqua Zumba, here are five great reasons to give it a try:

- Aqua Zumba is low impact – easy on your joints, but still a high energy calorie burn.
- Classes are great for beginners who want to dip their feet into an exercise class (pun intended!).
- Working against water is a great way to work and tone those muscles.
- Mount Druitt Swimming Centre is heated.
- And of course it's **FREE** (included with pool entry).

Grab a friend and bring your sunnies. Ditch the workout and join the party! See you at the pools.

Visit the website: **www.blacktown.nsw.gov.au/Services/Aquatic-and-leisure-centres/Our-pools-and-leisure-centres/Mount-Druitt-Swimming-Centre**

North West Community Services

NORTH WEST
COMMUNITY SERVICES INC.
Building a stronger community together

Address: Sam Lane Complex, 9 Park Street, Riverstone
Tel: (02) 9627 3622
Email: reception@nwcs.org.au

Make new friends and get fit at this friendly neighbourhood centre. Classes are available at least once a week for all fitness levels and ages.

Classes include:

Zumba – Zumba is a fitness program that involves cardio and Latin-inspired dance. It is easy to follow choreography that focuses on balance, range of motion and coordination. Thursdays 12 noon-12.45pm

Zumba Gold – Active older adults that are looking for a modified Zumba class that recreates the original moves at a lower intensity. Mondays 12 noon-12.45pm

Zumba Elara – Regular Zumba class located at the Community Hub in Elara Marsden Park. Monday-Thursday 1.00pm-1.45pm

Visit the website: nwcs.org.au

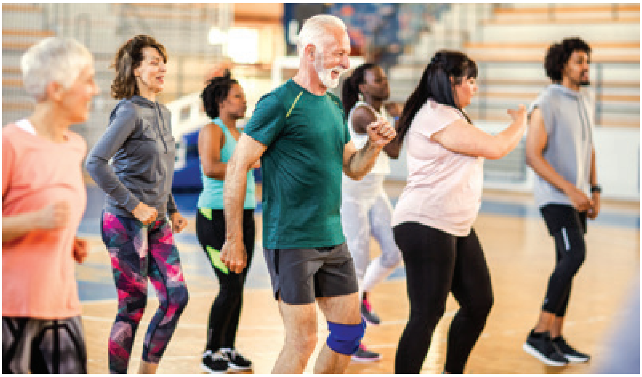

NSW Active and Healthy

Active and Healthy is an online directory of exercise programs within NSW for people 50 years and older of varying fitness levels. Searches on the website produce a number of exercise programs located near your chosen suburb/town and can be refined to display specific types of exercise.

Some of the programs include Tai Chi, qigong, falls prevention, cycling, gentle exercise, strength and resistance training, yoga, pilates, seniors fitness, aqua fitness, line dancing and zumba.

The website is easy to use and provides a large range of exercise options with contact details, maps, addresses, class times and fees. The search results can also be printed with the class name and contact details displayed. Because there is such a large range and class details change regularly, we suggest that you contact the program provider to confirm the class details.

Visit the website: **https://www.activeandhealthy.nsw.gov.au**

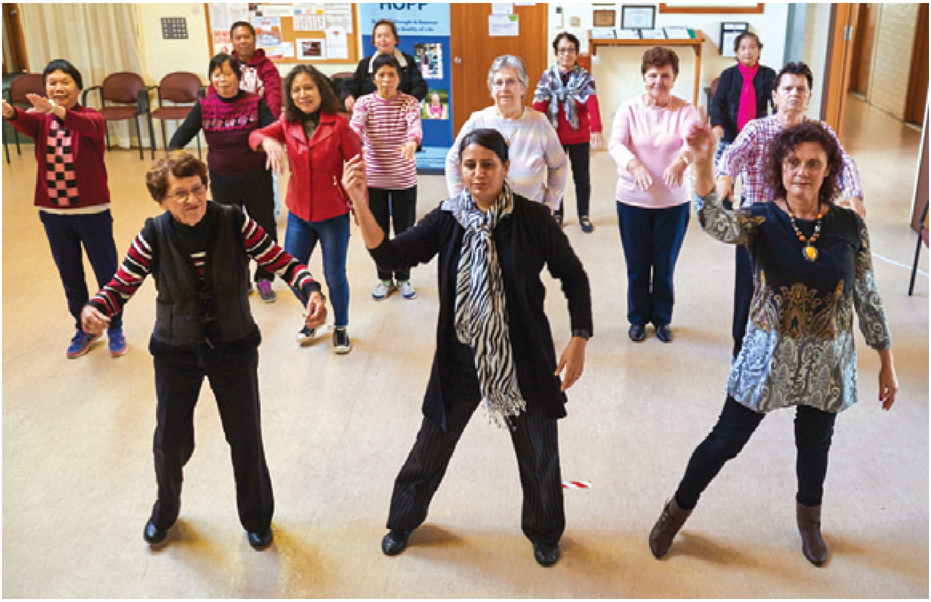

OLOC Get Yourself Moving Programs

These programs are for people 55 and over who live at home and who might want to improve their balance, strength, flexibility or cardiovascular fitness. The exercise programs also target people who have a chronic health condition and may benefit from a targeted exercise intervention.

The exercise program is suitable for anyone who may:

- Want to improve their ability to move about independently at home;
- Be concerned about the effects of sitting for too many hours of the day; and
- Be recovering from an acute illness or a stay in hospital.

Whatever the reason, for a small contribution, you can meet with our Exercise Physiologist and enjoy a group-based exercise program focusing on real-life goals and improving functional capacity.

OLOC Get Yourself Moving Programs operate from two centres in the western suburbs of Sydney:

Toongabbie Community Centre – 70 Aurelia Street, Toongabbie (parking available under building)

St Marys Community Hub – 29 Swanston Street, St Marys

For further details on the above programs, or our other Western Sydney locations, please contact:

Andrea El Boustani
GYM Program Manager
Phone: **(02) 9832 5451**
Email: **andreaelb@oloc.com.au**

Visit the website: **www.olocgetyourselfmoving.com**

parkrun

(Australia's Latest Social Prescription)

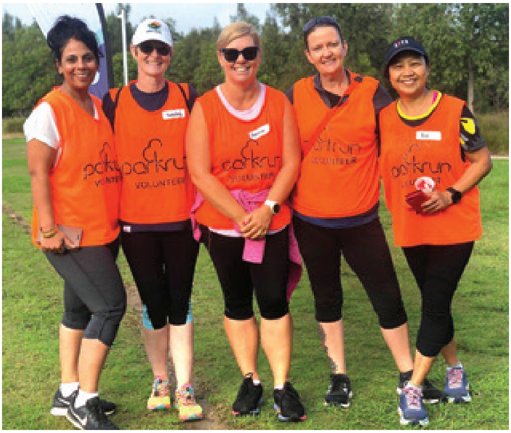

parkrun is the name, but look a bit closer and you'll discover why this simple intergenerational activity has captured the imagination of over 800,000 Aussies in 450 communities around the country.

The concept is simple and completely **FREE!** parkruns are established and coordinated by volunteers from the local community. Each event takes place in a public open space every Saturday at 8.00am on a 5km course. People are invited to take part in whichever way they choose – walk or run the route, lend a hand as a volunteer, or simply spectate and socialise. After the event it's off to a nearby cafe or meeting place for a coffee and a chat.

parkruns are non-competitive with the emphasis on regular participation rather than performance, with milestone shirts available to participants throughout their parkrun journey. Walkers and runners can earn shirts after 10 parkruns for juniors and 25 for adults.

The benefits of parkrun for individuals and communities are extensive and parkrun is being increasingly utilised by primary healthcare professionals as a social prescription.

Beyond the obvious benefits of fitness, fresh air and friendships, more and more patients and practitioners are identifying ways that parkrun can help transform people's lives. These include: motivation to get up and out on a Saturday morning, integrating into a new community, practicing speaking English, discovering local open spaces, belonging to the community, developing skills, increasing confidence, quality family time, and interacting with people of different ages, cultures and backgrounds.

Register for **FREE** before your first parkrun at **www.parkrun.com.au/register**, print your personal parkrun barcode and take it along to any parkrun in the world on any Saturday. It's that easy!

Reclink Australia

This fantastic program is coordinated by Reclink Australia. The aim is to enhance the lives of those experiencing disadvantage by providing inclusive sports and recreation programs. In partnership with more than 200 community organisations, Reclink Australia's programs create pathways to improved health and wellbeing, education and employment outcomes for all participants.

Reclink was launched in Victoria over 30 years ago. It has only recently been introduced to NSW and there are a range of sport and recreation activities available to member agencies. Activities include exercise programs, dance programs, tai chi, ten pin bowling, swimming activities, tennis, soccer, basketball, touch football and netball sessions, yoga and much more.

Organisations can join Reclink easily, **www.reclink.org/get-involved/become-member**. If your organisation would like to provide or encourage sport and physical activity for disadvantaged communities, you can become a member organisation. It will cost $130 for your organisation to join. Once you have joined, you will be able to access discounted or **FREE** sporting activities and make them available to your clients.

Reclink will also work with you to develop additional programs specifically for the interests or requirements of your clients.

To find out more, check out the Facebook page, **www.facebook.com/ReclinkAustraliaNSW/** or contact the State Manager.

Visit the website: **www.reclink.org**

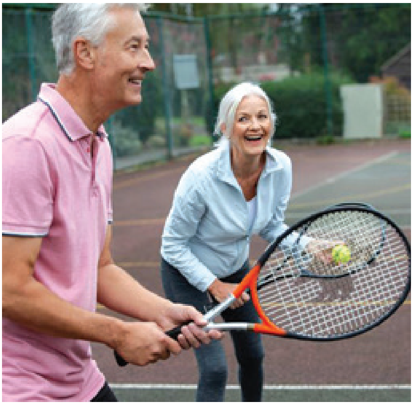

Riverstone Swimming Centre

Address: Cnr Market Street and Piccadilly Street, Riverstone
Tel: (02) 9627 1496

This seasonal pool is open from 9.00am to 6.00pm daily, from September to March each year. The Centre prides itself on being more than just a pool, offering health, fitness, fun and leisure opportunities for everyone. This includes 3 pools (including a toddlers pool), Aqua Learn to Swim, outdoor playground and vast open space. For pool entry fees, visit the centre website: **www.blacktown.nsw.gov.au/Services/Aquatic-and-leisure-centres/Our-pools-and-leisure-centres/Riverstone-Swimming-Centre**.

Robinhood Free Outdoor Fitness

ROBINHOOD
FREE OUTDOOR FITNESS

This website provides excellent information on local fitness facilities and the good news is that they are all **FREE**. The website provides information on outdoor gyms, pools and fitness activities including group fitness classes, dance and mind/body groups.

To find suitable facilities just type in your location and how far you are prepared to travel. You will be presented with a range of **FREE** fitness facilities. They include a list of what equipment is available at each venue, as well as maps and directions on how to get there.

Photos of the equipment are included as well as videos which provide exercise instructions on how to use the equipment.

Visit the website: **https://freeoutdoorfitness.net**

Ruth Everuss Swimming Centre

Address: Church Street, Lidcombe (near Olympic Drive)
Tel: (02) 9749 5031 – Contact: Tony Micallef (Swim Centre Operations Manager): tony.micallef@cumberland.nsw.gov.au

At Auburn Ruth Everuss Aquatic Centre we offer you the best in swimming, fitness and fun. Our multi-activity facility serves the Lidcombe/Auburn community and surrounding suburbs. You can experience advanced aquatic and leisure programs and facilities, catering to the inclusion of people of all ages and abilities.

With an outdoor 50m and waterpolo pool, indoor 25m, family splash area and toddler pool, indoor program pool with privacy curtains and an outdoor splash park for hours of fun, we have your Aquatics needs well and truly covered.

We also offer you a warm program pool for your hydrotherapy needs and a splash pad with a wide slide to keep your children entertained.

Bookings will need to be made in advance to avoid disappointment for our gym, group fitness classes, and use of the pool. To book visit the Auburn Ruth Everuss Aquatic Centre website or contact the centre on 1300 482 575.

General admission and fees are available on the website:
www.auburnaquaticcentre.com.au/

Regular opening hours

- 6am to 7pm, Monday to Friday (The Pools close at 7pm and The Gym closes at 9pm)
- 7am to 7pm, weekends
- Closed Good Friday, Christmas Day and Boxing Day

Classes

Auburn Ruth Everuss Aquatic Centre offers a range of fitness and health activity options for people of all ages and abilities:

- GOswim Program with group and private lessons available for all ages and levels of experience
- Swimming squad
- Aqua aerobics

We have a fully functional gym and over 30 group exercise classes each week to keep you motivated and moving. You'll be well on the way to being fitter and healthier than ever with Boxing, Cross Training, ABT (Abs, Butts & Thighs), Yoga & Gentle Yoga and Zumba & Zumba Gold.

Waves Fitness & Aquatic Centre

Address: 44 Mileham Avenue, Baulkham Hills
Tel: (02) 8848 6789

General Opening and Fitness Centre Hours:

- Mon – Thurs: 6am-9pm
- Friday: 6am-8pm
- Weekends: 7am-7pm
- Public Holidays: 8am-6pm
- Closed on Good Friday & Christmas Day

Pools have individual opening and closing times, so check the website for specific details.

The new Waves complex, consisting of two outdoor and two indoor swimming pools, the latest state-of-the-art Techno-Gym equipment, fitness classes, sauna, spa, and café will offer a very high standard of facility for our members and casual users, and is equal to the best facilities in Sydney.

Our Fitness Centre accommodates both the serious athlete and the casual health, recreational, and social user and offers the latest Techno-Gym equipment. This centre has a variety of exercise programs suitable for all levels of fitness. Prices vary per class and membership discounts and concessions are available. It's a good idea to check with the friendly staff to find out which class (or classes) best suit you and the cost involved.

An example of the classes available include Cycle, Boxing, Pilates, Circuit, Body Pump, Yoga, Zumba and Move for Life.

Aqua exercises include Aqua Fit and Aqua Tabata.

Visit the website: **https://www.wavesfitnessandaquatic.com.au/Home**

Wentworthville Memorial Swim Centre

Address: 115 Dunmore Street Wentworthville
Tel: (02) 8757 9050

Wentworthville offers 2 pools, a 50 metre competition pool and a 25m pool with beach and ramp entry, as well as a splash pad, swimming lessons, aqua aerobics and **FREE** parking.

This pool is open from October to March and closed from April to September. Check the website for opening times during the Summer months.

Visit the website: **https://www.cumberland.nsw.gov.au/wentworthville-memorial-swim-centre**

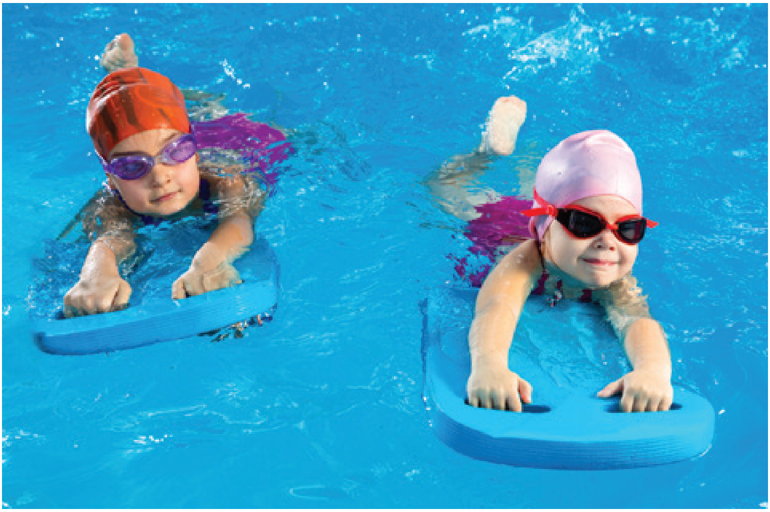

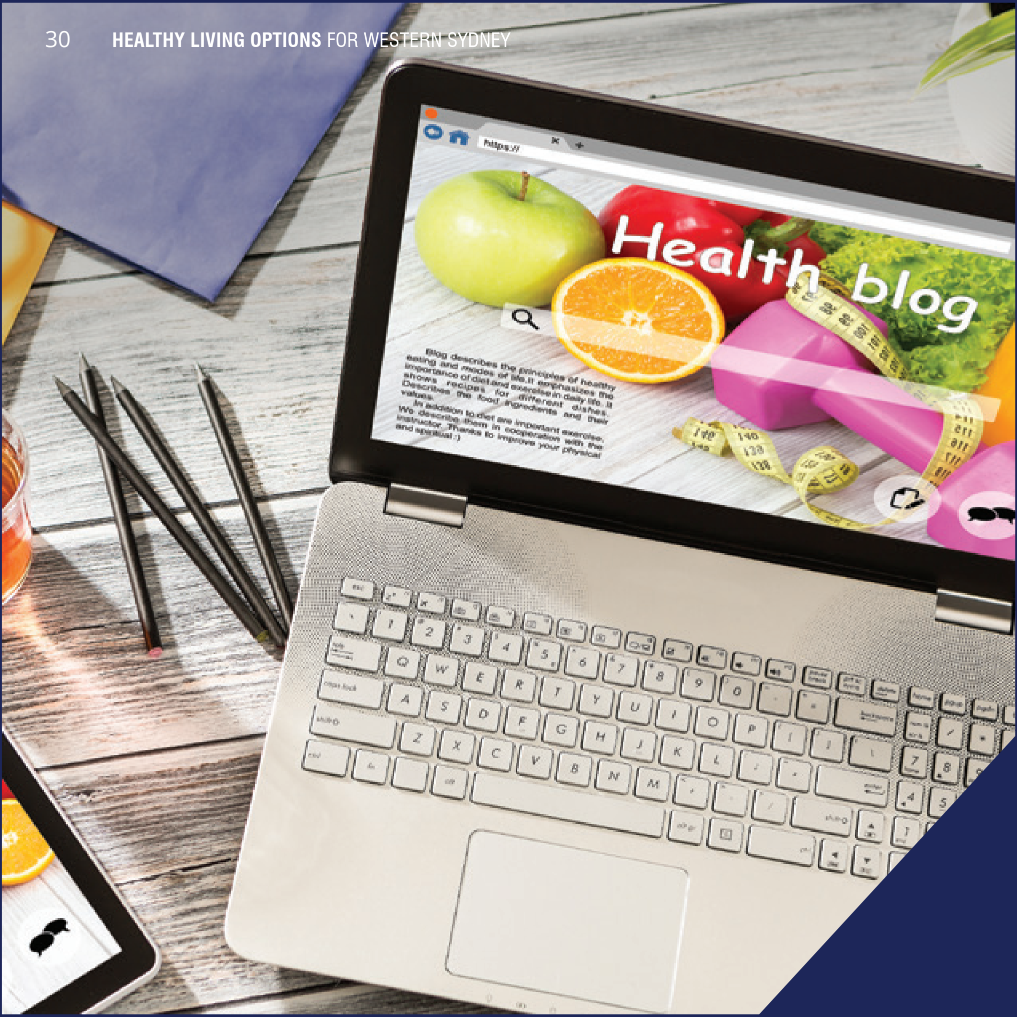

2 ONLINE HEALTHY LIFESTYLE CLASSES

Fitness Blender

Fitness Blender is a popular YouTube channel featuring over 600 workouts that can be searched by length, difficulty, training type, calories burned and muscle group. They have videos suitable for all fitness levels, as well as excellent options for when you might want a really short but intense workout. Over 500 **FREE** workouts range from 10-85 minutes long, from beginner level to elite athlete, from HIIT to Pilates, and strength training to bodyweight.

Fitness Blender believe reliable fitness, nutrition, and wellness content should be accessible to everyone, regardless of income. You can also pay a small membership fee to join up and receive access to full length workout programs, challenges and meal plans.

Visit the website: **https://www.fitnessblender.com/page/about-fitness-blender**

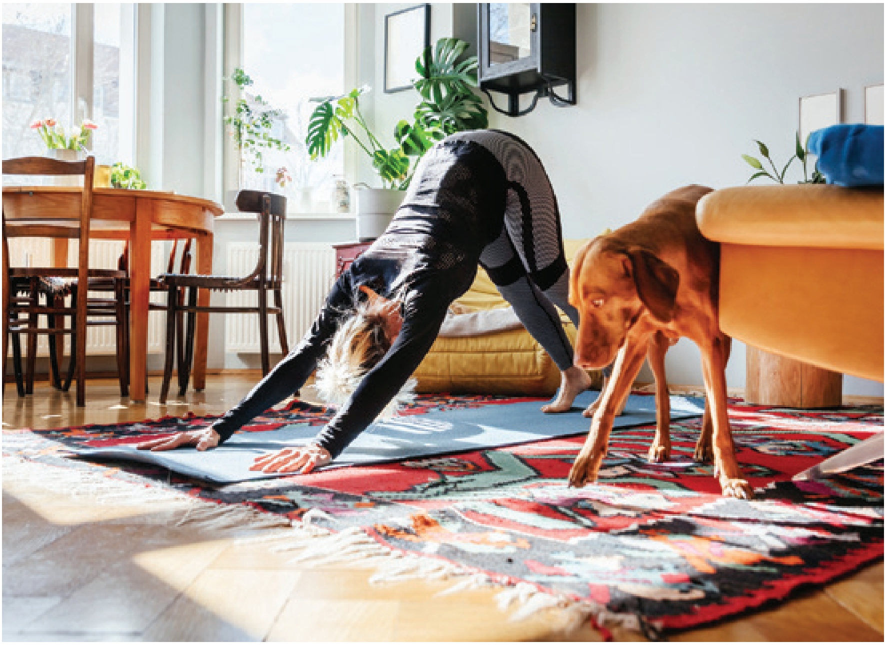

Healthy and Active for Life Online

Healthy and Active for Life Online is a **FREE** 10-week healthy lifestyle program for adults aged 60 years* and over. Each program begins at the start of school term.

- The program will help you learn how to make small, sustainable changes in your lifestyle to improve your health.
- The program covers lots of topics including healthy eating and physical activity.
- Basic exercises are included and are suitable for beginners.
- No prior knowledge or exercise experience is required!

*Aboriginal people aged 45+ years can register.

What's included in the program?

Healthy and Active for Life Online participants receive:

- A Healthy and Active for Life Online account
- Access to weekly online healthy lifestyle modules
- Access to two online basic exercise programs for beginners
- Weekly handouts and an exercise manual
- Telephone support to keep you motivated
- Ongoing access to your account for 12 months

Join Healthy and Active for Life Online now to get started!

Visit the website: **https://www.activeandhealthy.nsw.gov.au/home/healthy-and-active-for-life-online**

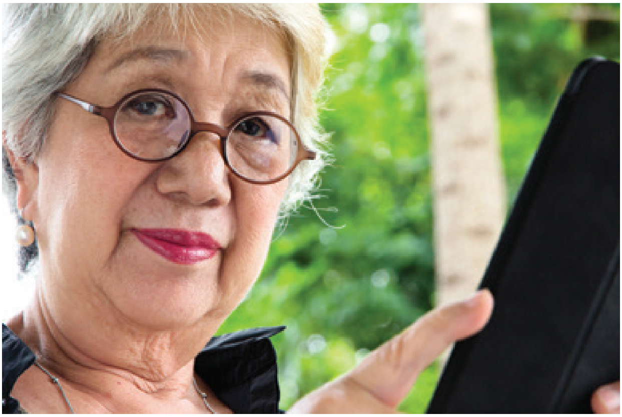

NHS Fitness Studio Exercise Videos

Take your pick from 23 instructor-led videos across our aerobics exercise, strength and resistance, and pilates and yoga categories.

These workouts have been created by fitness experts InstructorLive and range from 10 to 45 minutes.

There are exercises to tone your abs, raise your heart rate, and tone your upper arms. You will also find workouts suitable for new mums, such as postnatal yoga, or health problems, such as pilates for back pain.

Plus, there's the Wake up! workout, Vinyasa flow yoga, and Belly dancing for beginners to get you moving.

For more **FREE** exercise ideas, check out our Strength and Flex exercise plan.

Visit the website: **https://www.nhs.uk/conditions/nhs-fitness-studio**

NIKE Training Club – Free App

Reach your fitness goals with the Nike Training Club App (NTC). With access to 200+ **FREE** workouts and targeted training programmes, the NTC App will support you every step of the way. From invigorating yoga classes to bodyweight sessions with and without equipment, you'll find the right workout for you.

Follow our Nike Master Trainers through varied classes, ranging from 5–50 minutes. Do them at home, in the gym, or outdoors – whenever and wherever you want. Plus, our **FREE** video library is constantly growing, so expect new videos to flow in.

No matter what fitness level you're at, NTC will give you the tools you need to succeed.

Visit the website: **https://www.nike.com/au/ntc-app**

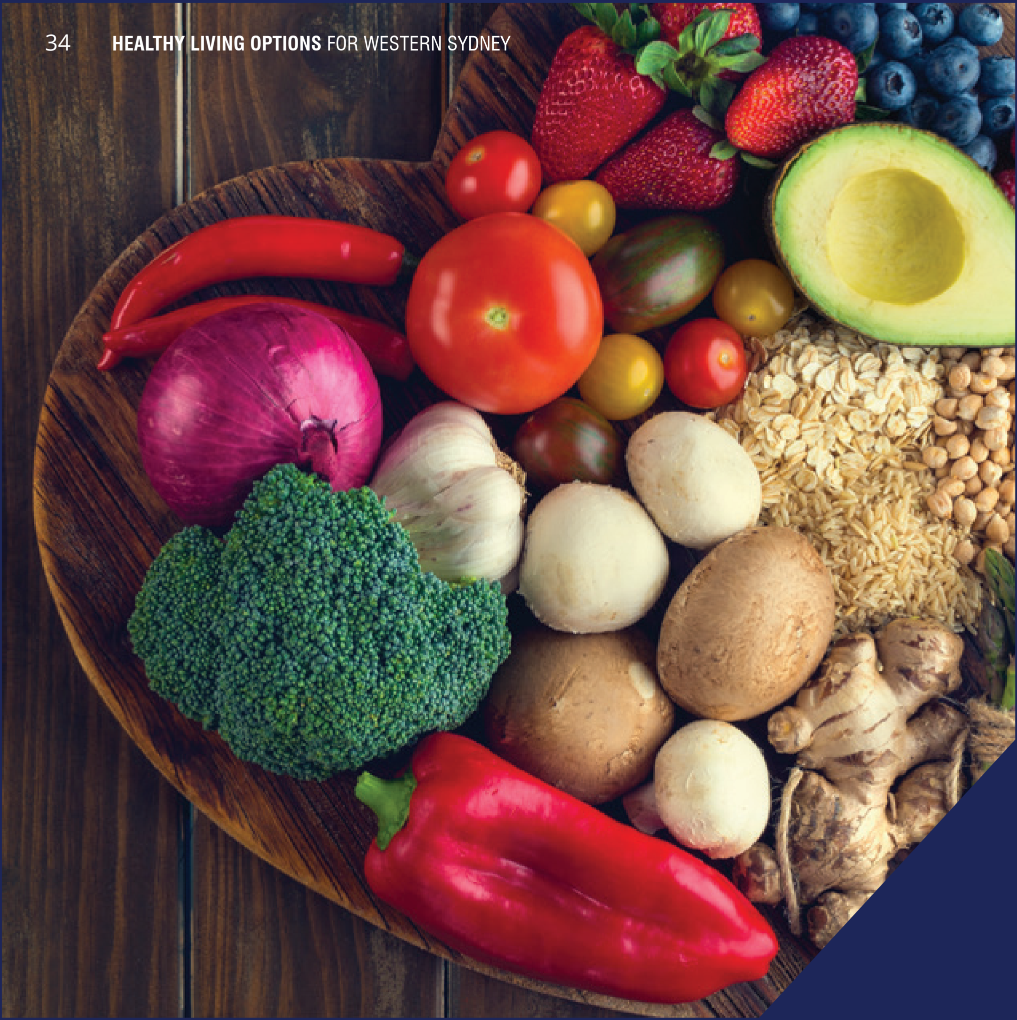

3 DIETARY ASSISTANCE

Box Divvy – Social Enterprise Business

Address: Food Hubs all across NSW and ACT

Cut your food bill in half! Box Divvy is the supermarket alternative where you can buy fresh food and groceries, local and Australian, up to 30% less than online supermarkets because it's direct from growers and food wholesalers.

The community owns the food system whilst providing easily accessible, good quality, inexpensive food to families, without the storage, food miles, packaging and food waste.

You can join your neighbourhood Box Divvy Food Hub where you order and pay online and either pick up from your Hubster, the person who runs it, or have your order home delivered if they offer it.

Want to be actively involved? It's a great side-gig so maybe run a Box Divvy Food Hub.

Earn some income from home, for your sporting group, for your community centre – there are endless possibilities. It's like running your own enterprise without the hassles. No investment required but you do need a space to pack, plus 4-5 hours a week (depends on Hub size) to pack orders and love people so you communicate with your members. You will also be given a Box Divvy Facebook page.

Interested? Either register to Join a Hub or Run a Hub.

Visit the website: **www.boxdivvy.com**

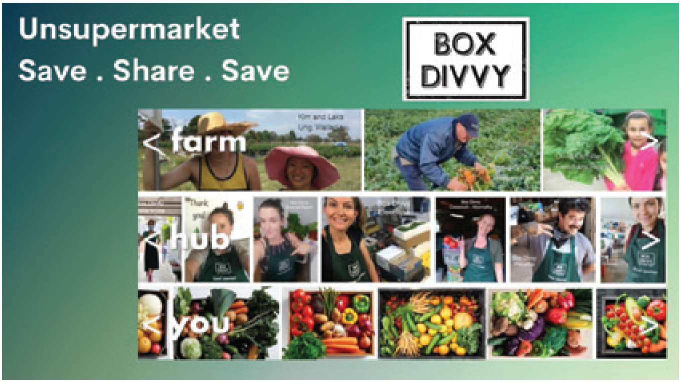

Cancer Council Free Resources

Healthy Lunch Box Website

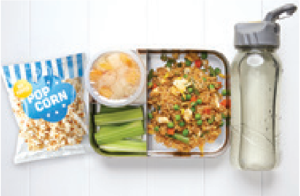

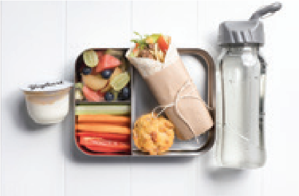

Cancer Council's website **healthylunchbox.com.au** is a one-stop-shop for everything you need to know about packing a healthy lunch box. The website features an interactive and educational, Healthy Lunch Box Builder for adults and kids. On the website you will find quick and easy recipes for every meal. There is also snack ideas, videos and blogs. You can subscribe to receive new recipes and nutrition information delivered straight to your inbox.

Visit the website: **https://healthylunchbox.com.au**

CSIRO Low-Carb Diet

The CSIRO Low-Carb Diet is a prescriptive, nutritionally complete eating and lifestyle plan, which is lower in carbohydrates and higher in proportions of protein and healthy fats – combined with a tailored home-based exercise program.

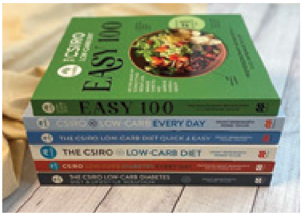

Clinical research demonstrates this diet and lifestyle approach is highly effective in achieving sustained long-term weight loss and compared to a traditional higher carbohydrate, low fat diet is more effective in reducing risk factors of type 2 diabetes. This includes controlling blood sugar levels and reducing risk factors for heart disease and diabetes medication requirements.

This evidence-based diet plan has been translated into The CSIRO Low-Carb book series featuring nutritious low-carb, high-protein and healthy fats recipes, with weekly meal and exercise plans, shopping lists and creative meal builder ideas. This plan will help you to prevent and/or manage type 2 diabetes, reach your weight-loss goals and improve your overall health and wellbeing.

Books can be purchased at department and bookstores or visit the website: **https://www.csiro.au/lcd**

No Money No Time

Looking to improve your diet? Have no money for fancy ingredients and no time to cook? This website will have you covered. Take the Healthy Eating Quiz to see where you can improve your diet and then find personalised **FREE** recipes and diet facts from leading experts in nutrition and dietetics from the University of Newcastle, Australia.

This website will help you cook healthy, easy recipes. If it's not tasty, fast, cheap and packed with good stuff, you won't find it here. The recipes are constantly updated, so you can keep coming back for more inspiration. They include useful information for each recipe including the time needed to make it, the number of ingredients and the cost per person. They even outline what equipment you will need and give you tweaks that you may like to try to cater for your own taste. Along with all this, they even provide some information on why this recipe is good for you. A great resource for everyone who is busy and careful with their budget.

Visit the website: **https://nomoneynotime.com.au**

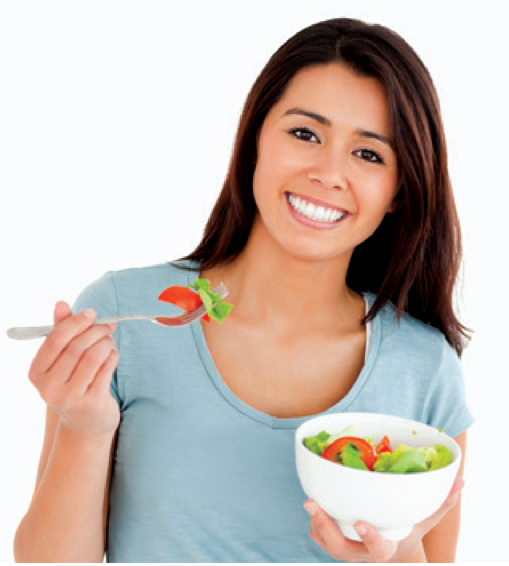

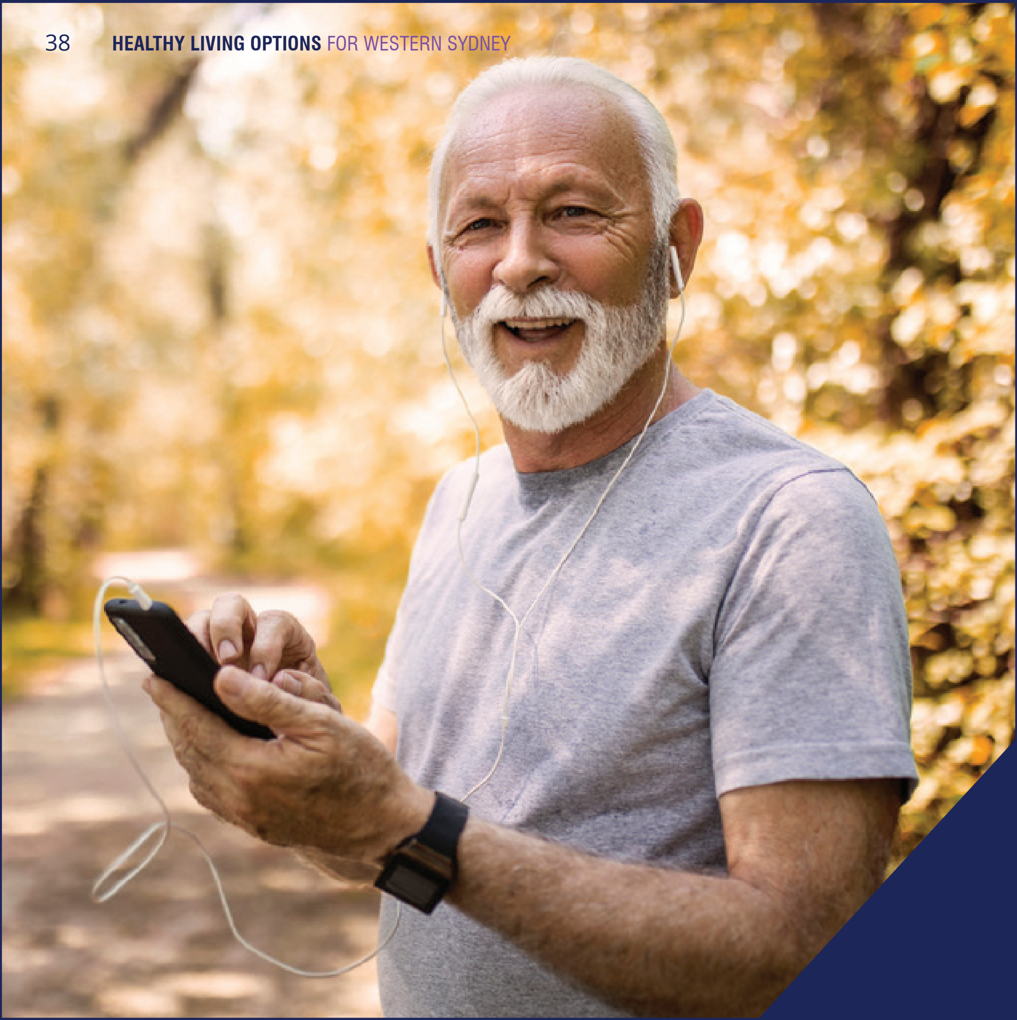

4 PHONE-BASED PROGRAMS

Get Healthy Information and Coaching Service

The Get Healthy Information and Coaching Service is a **FREE**, confidential telephone-based service which helps people make lifestyle changes regarding healthy eating, being physically active and achieving and maintaining a healthy weight.
The Service is open to anyone aged 16 years and over. You will get your own university qualified personal health coach to help you make healthy lifestyle changes.

Your health coach could help you to:

- Eat healthily
- Get active
- Reduce alcohol intake
- Achieve and maintain a healthy weight
- Gain or maintain a healthy amount of weight during pregnancy

What's included?

- Your own personal university-qualified, health coach
- 10 -13 confidential coaching calls over six or more months – all at a time and day that suits you
- Motivation and support to set your own healthy lifestyle goals
- Motivational emails and/or texts
- Support to help you track your goals and actions
- Help to overcome anything stopping you from reaching your goals
- Option to re-enrol for coaching or book coaching sessions online for **FREE**.

How do I get started?

Starting your Get Healthy Journey has never been easier. Complete our simple online form today or call 1300 806 258.

Visit the website: **www.gethealthynsw.com.au**

The COACH Program

Individual Telephone Health Coaching

Integrated and Community Health Directorate, Integrated Care Program
Tel: 1800 113 644. Email: WSLHD-IntegratedChronicCareProgram@health.nsw.gov.au

About the Program

The COACH Program, run by Western Sydney Local Health District, helps people with a chronic disease, such as diabetes, heart and lung disease, to manage their risk factors while working in partnership with their usual doctor/s.

Each patient with a chronic disease is allocated their personal coach. The coach will work with the patient, either on the telephone or by mailing out information in a series of regular coaching sessions over 6 months. The coach helps the patient to maintain their health by setting and achieving health goals for themselves.

Visit the website: **www.thecoachprogram.com**

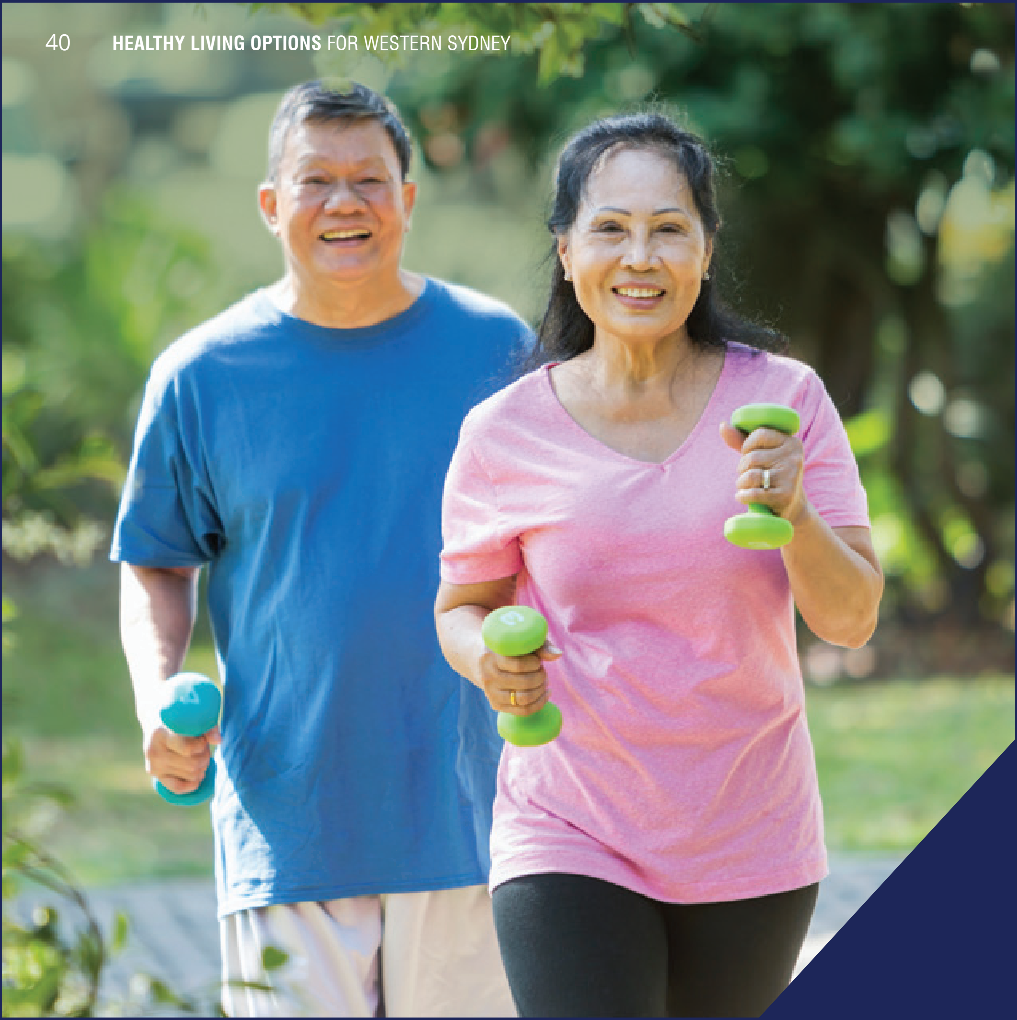

5 FACE TO FACE PROGRAMS

Bilingual Community Education

The Bilingual Community Education (BCE) program was developed to improve the health of individuals from Cultural and Linguistic Diverse backgrounds (CALD).

All programs are specifically designed for CALD individuals, and conducted by Bilingual Community Educators in the participant's own language to provide a safe environment that encourages sharing of experiences and supports the participant's ability to develop support networks.

Programs include:

- Diabetes Awareness... in Your Community
- New Healthy Women
- Women Growing Older
- Healthy Eating and Physical Activity
- Living Well in the Middle Years
- Healthy LifeStyle
- 'Bone Up' Osteoporosis Awareness Program

Organisations contact: Anoop Johar, BCE Officer, Multicultural Health WSLHD Tel: (02) 9840 3907. Email: anoop.johar@health.nsw.gov.au

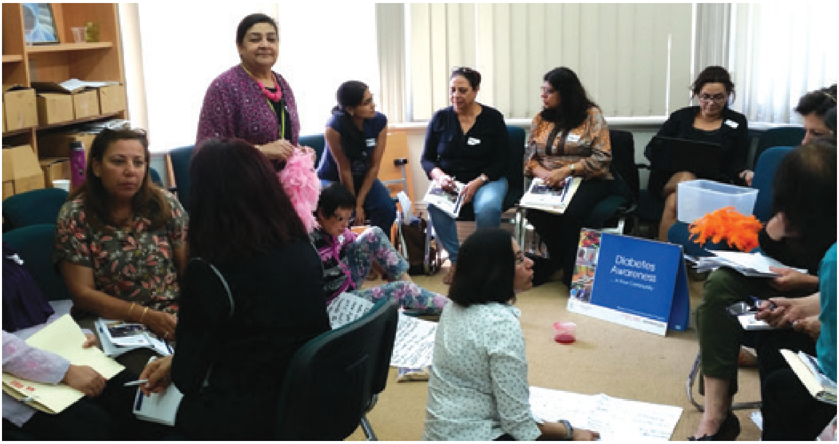

Blacktown Older Women's Network

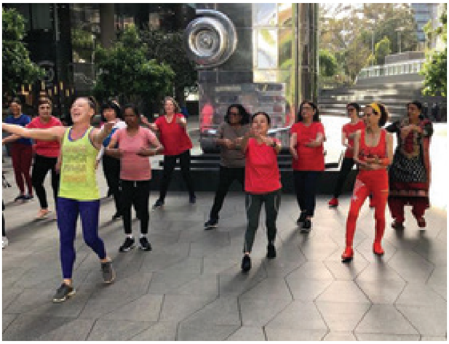

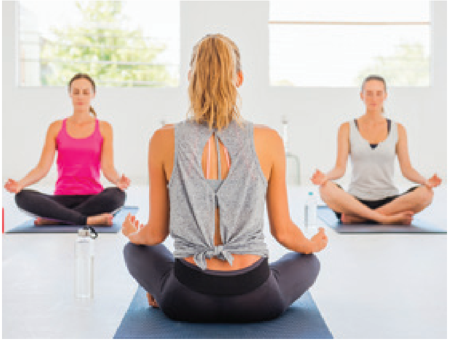

Address: 32 Rooty Hill Rd Sth, Rooty Hill 2766
Tel: 0418 447 815
Coordinator: Robyn Atkins via email: ownblacktown2766@gmail.com
We meet each Tues, Wed and Fri

There's something to suit all levels of fitness and all tastes:

- Gentle Exercise
- Heart Moves
- Line Dancing
- Meditation

All activities are designed to empower you as an individual, while at the same time, increase and maintain your health and wellbeing.

Visit the website: **https://ownnsw.org.au/get-involved/join-group/blacktown-wellness**

Blacktown Women's and Girl's Health Centre

Address: 6 Prince Street, Blacktown
Tel (02) 9831 2070
Email: info@womensandgirls.org.au

Blacktown Women's and Girl's Health Centre is a community-based, non-profit women's health centre which provides accessible health and wellbeing services for women and girls in Blacktown regardless of their income.

The centre provides health services to women and girls in Blacktown **FREE** of cost or at very low cost including:

- OzHarvest
- Foodbank
- Gentle Exercise
- Women's Walking Group
- Dietetics, Naturopathy and Nutrition
- Information workshops

Visit the website: **http://womensandgirls.org.au/contact-us**

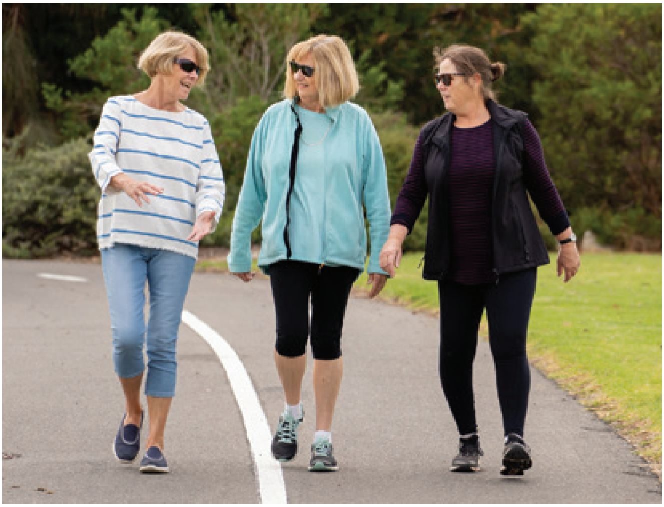

Chronic Disease Self-Management Program

Integrated and Community Health Directorate, Integrated Care Program.
Tel: 1800 113 644. (For non-English speakers call TIS National on 131 450)

The Stanford Chronic Disease Self-Management Program, run by Western Sydney Local Health District, is for people in the local community who want to:

- Make better choices for their health and lifestyle
- Gain a better understanding of their chronic disease.

This is a **FREE** 6-week program offered in a number of languages and held in local community venues. Morning tea is provided.

Who can attend

The program is for anyone over the age of 16 who has one or more of the following chronic diseases:

- Diabetes
- Heart failure
- Heart disease
- High blood pressure
- Chronic obstructive pulmonary disease (lung disease).

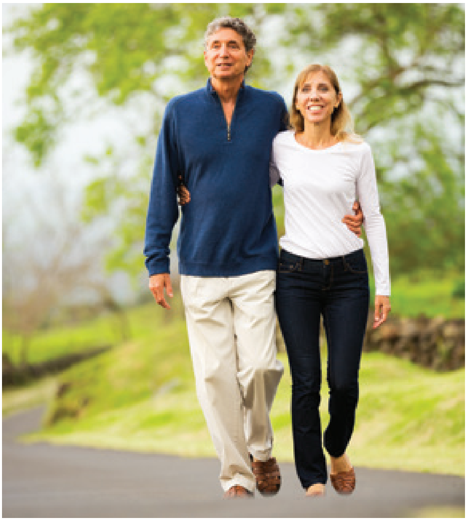

Collective Leisure

Contact: David Burns, CEO
Tel: 0451 441 434
Email: davidb@collectiveleisure.com.au

Collective Leisure is Australia's first social enterprise leisure management company, certified through the regulator Social Traders. A social enterprise is a 'business for good'.

Their mission is to reduce health inequity within our communities. This is summarised through their purpose statement 'enabling well-being without boundaries'.

Collective Leisure was founded in 2019 from a deep sense of social justice, the awareness that global health and well-being are tracking poorly, and the desire to create a sustainable social enterprise.

Selection of Programs

- Well-Being Initiative – Bespoke well-being programs
- Smash Tennis Mental Well-being Initiative – Supporting people in their mental health recovery
- Sportability Clubs – Weekly multi sports for people with disability
- Adaptive Sport School Programs – For children with disability
- Employment and training – Pathways for marginalised groups in the leisure industry
- Sports Mentoring Program – Youth-led program for underserved CALD young people using sport

*Active Kids registered *NDIS funding can be used

Collective Leisure works on the ground with community but is the initiator and leader of a systems change project.

WSYD Moving – A whole of system initiative to reduce physical inactivity in Western Sydney. The WSYD Moving Community of Practise meets every 8 weeks on a Thursday – 9:30am to 10:30am. We are a group of people who share a passion for reducing physical inactivity in Western Sydney. This is a platform for sharing, learning and collaboration by members and invited stakeholders.

Visit the website: **https://www.collectiveleisure.com.au**

Diabetes Australia

Education Programs

Diabetes Australia offers education programs in Western Sydney that are **FREE** for anyone registered with the National Diabetes Service Scheme (NDSS). These include:

- **Shop Smart** – will help you to understand how to read food labels, and what to look for when choosing healthy options for yourself and your family. You will also receive a handy wallet card for quick reference when shopping.
- **Carb Smart** – will help you enjoy quality carbohydrates in a way that suits you and your diabetes.
- **Beat It** – Want help to reach your fitness goals? Beat It is an 8-week group exercise and lifestyle program to help you better manage your diabetes and improve your general health. This program involves moderate intensity aerobic, strength and balance-based exercises as well as education sessions on healthier living.
- **Ready Set Go, Let's Move** – Exercise, of course we all know it's good for health, especially if you have diabetes. But knowing where to start and how to stay motivated can be tough. This is where Ready Set Go... Let's Move comes in. Get support and be empowered to take the first step in making exercise a part of your routine, or perhaps increasing the amount you are already doing.
- **DESMOND** – Do you feel confident managing your type 2 diabetes? There is a lot to consider when you have diabetes, so if you ever feel unsure, don't worry, you're not alone. The DESMOND program recognises that there is no 'one size fits all' approach to diabetes management. It is intended to provide you with a welcoming and non-judgmental space where you can plan how you would like to manage your diabetes.
- **Living Well Events** – Changing your lifestyle isn't easy, especially on your own. The Living Well program gives you the motivation and support to make and maintain positive changes and live a healthier and more active lifestyle. Whether you were diagnosed recently or have lived with diabetes for some time, this is your chance to chat, ask questions, learn about the latest information on diabetes, and get new tips to help make life a little easier. Learn from diabetes experts about a range of topics, such as food and nutrition, physical activity, diabetes devices and technology and mental health. Access services and programs on managing diabetes. Connect with health professionals and other people with diabetes.

Many of these NDSS programs are also offered in an online environment. For a full list of events near you visit **https://events.ndss.com.au/** or phone the **NDSS Helpline 1800 637 700**.

In partnership with your Primary Health Network we are also proud to offer in language programs such as:

- **Looking After Your Health:** workshops aim to address the higher rates of chronic conditions and health disparities experienced by many people from CALD communities, compared to the general population. Available in the following languages; Korean, Hindi, Tagalog, Mandarin, Cantonese, Arabic, Turkish and Punjabi.

Please contact Diabetes Australia for more information 1800 177 055.

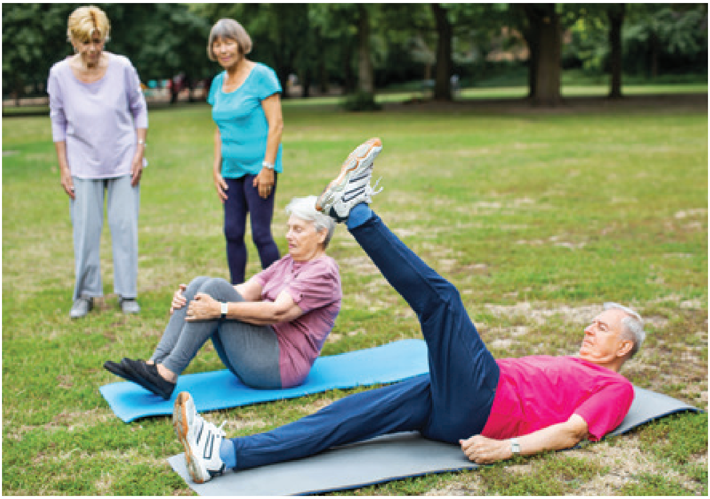

Go4Fun

Go4Fun is a 10-week healthy lifestyle program for kids aged 7 to 13 years who are above a healthy weight. The program focuses on improving eating habits, fitness and confidence.

Go4Fun programs are led by trained qualified health professionals and take place after school, once a week, running parallel with school terms. Children and their families have fun and become fitter, healthier and happier.

The program aims to improve the health of the child through the development of healthy lifestyle behaviours, as well as educating and positively affecting kids' attitude to food and exercise. The program includes:

Nutritional info:

- Food serving sizes
- Label reading
- Menu planning and recipes
- Guidelines for eating out and parties

Personal improvement:

- Physical activity
- Self-esteem

Support and advice:

- Talking about weight
- Goals and rewards
- Role modelling
- Problem solving

Light, fun games:

- Being active
- Movement skills

The Go4Fun program is highly effective in supporting healthy lifestyle changes for children and their families. The program has been running in NSW since 2009, with more than 13,000 families participating.

Western Sydney Go4Fun programs run in locations across Blacktown, The Hills, Cumberland and Parramatta Local Government Areas. We are now taking registrations for Go4Fun programs starting next term. Go4Fun is a **FREE** and fun program to help your family get more active and fitter.

To register for the program today, please call **1800 780 900** or call the local team on **02 9840 3603**.

Visit the website: **https://go4fun.com.au/locations/western-sydney**

New Edge Performance

Locations: Mt Druitt, Parramatta, Blacktown, Doonside, Schofields, Marayong
Tel (Mount Druitt Medical Centre): 1300 487921
Email: admin@newedgeperformance.com.au

Diabetes Lifestyle Education Program

This organisation runs a lifestyle self-management program for people with type 2 diabetes.

About the program:

- Education-based program allowing for self-management of condition.
- The Diabetes Lifestyle Education Program was developed by and is run by experienced Exercise Physiologists (EP) and Dietitians
- 72% of patients that attended all sessions of this program improved their BMI (weight loss) and HbA1c levels (improved sugar control).
- Initial assessment is completed by the EP
- 8 group based education sessions with Medicare rebates available for those with Type 2 diabetes
- Individual follow-ups and exercise maintenance program may also be available after or during program completion.

** Program is also suitable if you have pre-diabetes but no Medicare rebates available

Some of the topics covered are:

- Exercise and diet to optimise your diabetes
- Exercise and diet to aid weight loss
- Portion control for diet
- How much carbohydrates should you have
- Healthy food choices
- Food label reading
- Introduction to exercise to optimise diabetes
- How much exercise to do
- Tips to help you stick to an exercise routine
- Strength training for sugar control.

New Edge Performance (continued)

What you need to do to start the program:

1. Speak to your local GP to see if you are eligible for the diabetes lifestyle education program.
2. If eligible your GP needs to prepare a referral form for the group sessions. This form is called "Allied Health Group Services under Medicare for patients with Type II Diabetes" and if possible please include your latest and previous HbA1c results.
3. Once you have a referral you then need to book an assessment with the Exercise Physiologist.
4. After you complete the assessment you will receive a letter with the dates for the group sessions.

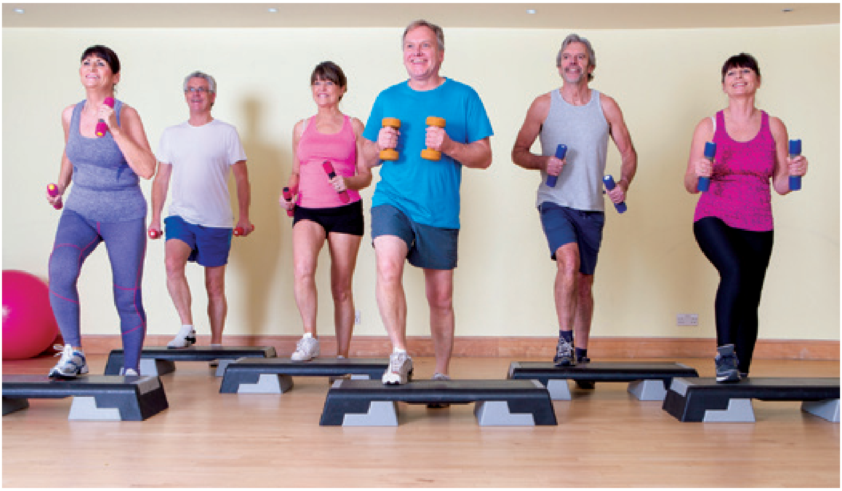

Optimum Health Solutions

Type 2 Diabetes and exercise program at Optimum Health Solutions

Address: Suite 2, 114 Main Street, Blacktown
Tel: (02) 8599 6275
Email: optimumenquiries@opt.net.au

An Allied Health team with Accredited Dietitians as well as Accredited Exercise Physiologists run an appointment-based treatment that allows success, with individuals held accountable for their lifestyle change.

What does the program involve?

- An individual assessment to review a patient's overall medical history, medications, physical capacities and goal setting
- Developing an appropriate exercise program to be performed at the studio in Blacktown
- Supervised gymnasium/hydrotherapy sessions, which are undertaken in a group environment (up to 12 people)
- These sessions are performed twice per week under the supervision of Optimum's Exercise Physiologists over four weeks
- A home program is also provided to perform independently.

Why use exercise to manage Type 2 diabetes:

- An individualised exercise program has been shown to improve insulin sensitivity and insulin dependent glucose uptake
- It can reduce body fat and improve lean muscle mass, which can translate to improvements in physical functioning and independence
- It can reduce other health co-morbidities such as hypertension, cardiovascular disease and obesity.

The benefits of a hydrotherapy pool in early stages of functional rehabilitation include: helping relieve pain, promoting relaxation, mobilising joints, strengthening muscles, and developing balance and coordination in a low weight bearing environment. There is also a studio in Werrington for people who live closer to St Marys.

Visit the website: **http://www.opt.net.au**

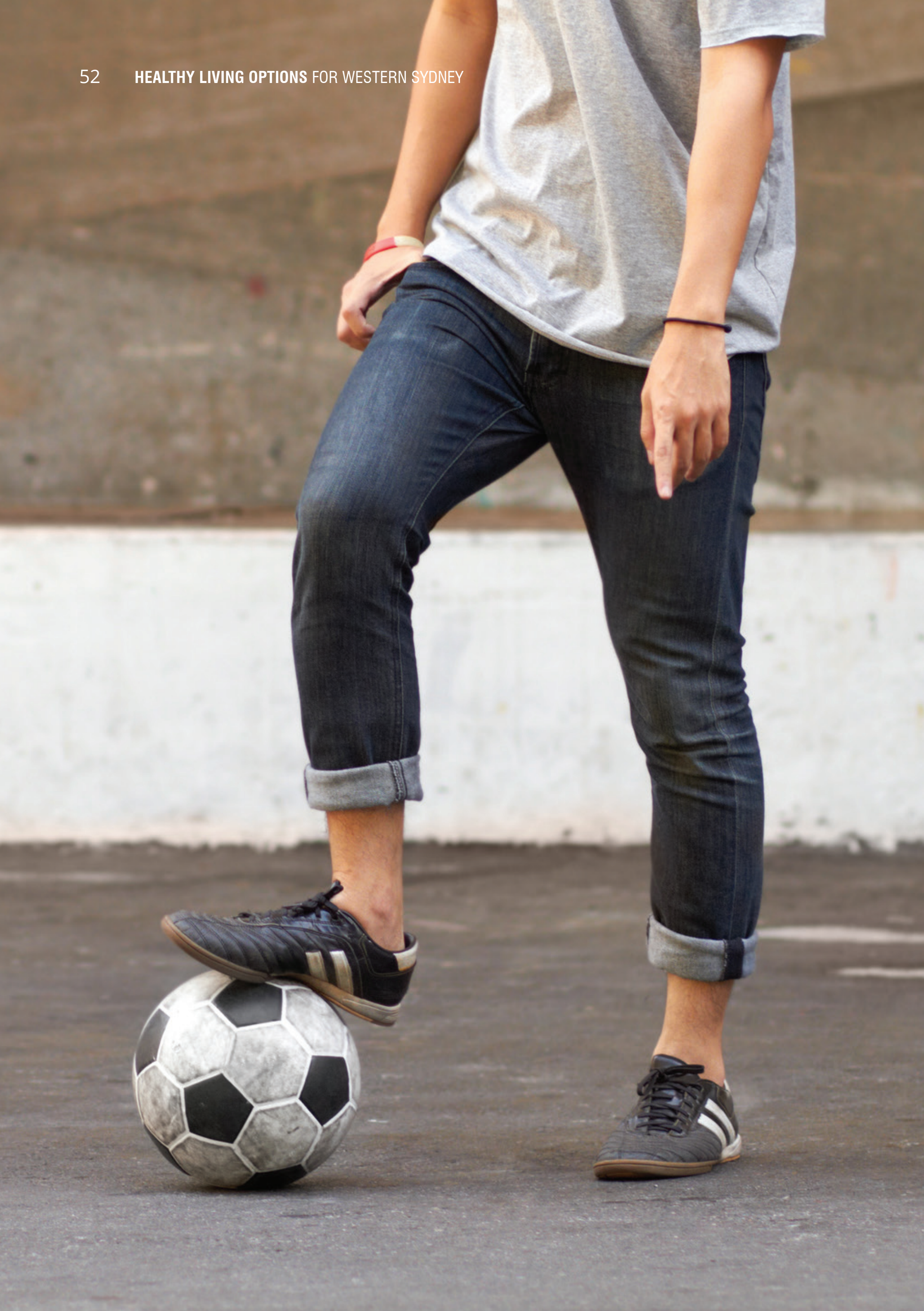

Street Soccer

Kicking a ball helps people feel good

The Big Issue's Community Street Soccer Program uses the power of sport to promote social inclusion and personal change for homeless, marginalised and disadvantaged people.

How does it work?

- It's **FREE**
- Males and females 16 years and above
- Once a week. The weekly sessions are fun, safe and provide a great opportunity to make new friends.
- Coaches encourage players to not only improve their soccer skills and fitness but most importantly to improve their lives. They act as role models, linking players to appropriate services to address their individual needs.
- Soccer activities and matches, special events and initiatives.

Benefits for street soccer participants include:

- Social inclusion and improved links with the community
- Re-connection with family
- Improved confidence and self-esteem
- Improved mental health
- Improved health and fitness
- A sense of belonging.

How can you help?

- Refer clients to Street Soccer and incorporate the program into support structure
- Bring participants to Street Soccer for them to see how it is run
- Coordinate transport for participants to attend Street Soccer or give directions

Parramatta Street Soccer Thursday 3.30pm – 5.30pm, Parramatta Park – West Domain, near corners of Park Avenue & Railway Parade, Parramatta

Contact the Coordinator: **soccerparramatta@bigissue.org.au**

Mount Druitt Street Soccer Tuesday 1:00pm – 3:00pm, Mount Druitt Town Centre Reserve, Ralph Place, Mount Druitt. Contact the Coordinator: **soccermountdruitt@bigissue.org.au** or just turn up.

Visit the website: **www.thebigissue.org.au**

Sydney West Sports Medicine

Address: Sydney Gymnastic and Aquatic Centre,
Rooty Hill RSL Club Complex, 12 North Parade Rooty Hill
Tel (02) 9851 5959
Email: reception@swsm.com.au

At Sydney West Sports Medicine, we're dedicated to providing exceptional healthcare resources to enhance the overall health and wellbeing of individuals in the Western Sydney area. We're heavily committed to assisting individuals in managing chronic diseases such as Type 2 Diabetes and providing programs that will improve the patient's health.

At Sydney West Sports Medicine, we offer Diabetes group education & exercise programs consisting of weekly exercise classes led by an Accredited Exercise Physiologist. The program aims to give people with diabetes the skills & knowledge to maintain a healthy and active lifestyle. These specifically designed Diabetes classes are held at our Rooty Hill clinic and are fully bulk billed by Medicare upon appropriate referral from your GP. Alternatively, they can be attended as a private patient for $25 per class. Beat-It programs are also provided under NDSS.

The group Diabetes exercise classes are enjoyable and include a combination of both aerobic and resistance training utilising Thera Bands (resistance bands) and dumbbells. Diabetes exercise classes may also involve land or pool based exercises to maintain diversity, motivation and ensure continual improvement.

- Assist with weight loss and improved body composition
- Improve Glycaemic control – Reduction in HbA1c levels reducing the risk of Diabetes-related complications.
- Increase exercise tolerance, strength and muscular endurance.
- Reduce systolic blood pressure and increase knowledge and awareness of the condition.

Visit the website: **https://www.swsm.com.au/sports-rehabilitation/diabetes**

Workers Blacktown Get Healthy Classes

Address: 55 Campbell Street Blacktown
Tel: (02) 9830 0600
Email: bwc@bwcl.com.au

Cooking demonstrations

Members of Workers Blacktown can enrol in **FREE** Healthy Cooking Demos which are held monthly in the Club. These classes run for 90 minutes and participants get the chance to learn all about nutrition along with how to prepare healthy meals and snacks that are easy, nutritious and delicious. There is plenty of time for discussion and questions. Make new friends, new skills and learn budget friendly recipes that you and your family will love.

Line dancing

Free beginner line dancing classes are available at the Club on a weekly basis. These classes are run for an 8 week term and are conducted for 45 minutes.
For bookings or more information contact: **janine.dawson@health.nsw.gov.au**

Visit the website: **https://workerslifestylegroup.com.au**

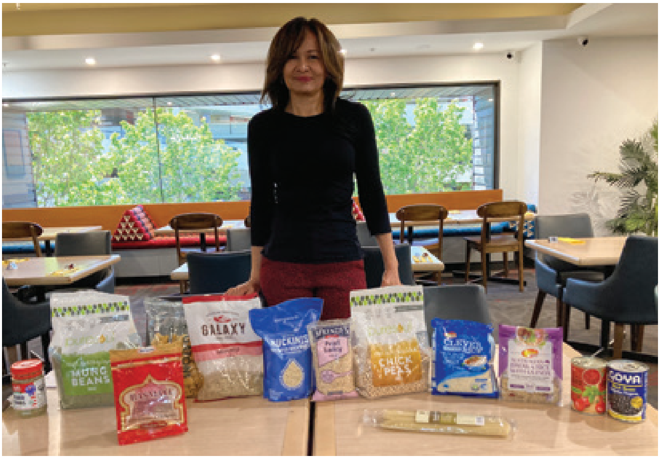

Western Sydney Diabetes
Beating Diabetes Together

Janine Dawson
Diabetes Prevention Manager

Telephone: (02) 8670 0015
Mobile: 0419 239179
Fax: (02) 9851 6146
Email: janine.dawson@health.nsw.gov.au
Web: www.westernsydneydiabetes.com.au
Follow us on LinkedIn

© July 2023

phn WESTERN SYDNEY
An Australian Government Initiative

diabetes australia

NSW GOVERNMENT
